# The Mouse Solitary Odorant Receptor Gene Promoters as Models for the Study of Odorant Receptor Gene Choice

**DOI:** 10.1371/journal.pone.0144698

**Published:** 2016-01-21

**Authors:** Andrea Degl'Innocenti, Marta Parrilla, Bettina Harr, Meike Teschke

**Affiliations:** 1 Max-Planck-Institut für Biophysik, Frankfurt am Main, Germany; 2 Unità di Biologia Cellulare e dello Sviluppo, Dipartimento di Biologia, Università di Pisa, Pisa, Italy; 3 Abteilung Evolutionsgenetik, Max-Planck-Institut für Evolutionsbiologie, Plön, Germany; Monell Chemical Senses Center, UNITED STATES

## Abstract

**Background:**

In vertebrates, several anatomical regions located within the nasal cavity mediate olfaction. Among these, the main olfactory epithelium detects most conventional odorants. Olfactory sensory neurons, provided with cilia exposed to the air, detect volatile chemicals via an extremely large family of seven-transmembrane chemoreceptors named odorant receptors. Their genes are expressed in a monogenic and monoallelic fashion: a single allele of a single odorant receptor gene is transcribed in a given mature neuron, through a still uncharacterized molecular mechanism known as *odorant receptor gene choice*.

**Aim:**

Odorant receptor genes are typically arranged in genomic clusters, but a few are isolated (we call them *solitary*) from the others within a region broader than 1 Mb upstream and downstream with respect to their transcript's coordinates. The study of clustered genes is problematic, because of redundancy and ambiguities in their regulatory elements: we propose to use the solitary genes as simplified models to understand odorant receptor gene choice.

**Procedures:**

Here we define number and identity of the solitary genes in the mouse genome (C57BL/6J), and assess the conservation of the solitary status in some mammalian orthologs. Furthermore, we locate their putative promoters, predict their homeodomain binding sites (commonly present in the promoters of odorant receptor genes) and compare candidate promoter sequences with those of wild-caught mice. We also provide expression data from histological sections.

**Results:**

In the mouse genome there are eight intact solitary genes: *Olfr19* (*M12*), *Olfr49*, *Olfr266*, *Olfr267*, *Olfr370*, *Olfr371*, *Olfr466*, *Olfr1402*; five are conserved as solitary in rat. These genes are all expressed in the main olfactory epithelium of three-day-old mice. The C57BL/6J candidate promoter of *Olfr370* has considerably varied compared to its wild-type counterpart. Within the putative promoter for *Olfr266* a homeodomain binding site is predicted. As a whole, our findings favor *Olfr266* as a model gene to investigate odorant receptor gene choice.

## Introduction

In the mouse, the detection of chemicals for the sense of smell relies on four anatomically distinct regions: the main olfactory epithelium (MOE), the septal organ, the vomeronasal organ and the Grüneberg ganglion. In these structures are located sensory neurons that express specific G-protein-coupled receptors, transmembrane proteins implicated in the signal transduction event that leads to the generation of smell perception [1][2][3][4][5][6].

Regarding the MOE, these receptors belong to a large gene superfamily comprising roughly 1100 intact genes [7], the odorant receptors (ORs) [1]. Remarkably, they are expressed in a monogenic and monoallelic manner, meaning that each mature olfactory sensory neuron (OSN) expresses one [8][9][10] out of approximately 2200 OR alleles [1][7], following a rule called *one-neuron one-receptor hypothesis*. The molecular mechanism underlying this property, generally referred to as *OR gene choice*, is still largely unknown. It is thought that (see *stochastic model* [11][12][13]) some random choice event must occur among promoters that are, to some extent, functionally equivalent. This would lead to the so-called *punctate* expression of OR genes: within their expression domains in the MOE, OSNs expressing the same OR gene are usually not contiguous. As of today, common motives discovered in OR gene promoters are homeodomain (HD) and olfactory/early cell B (O/E) transcription factor binding sites (TFBSs) [14][15][16][17][18][19], and it is believed that epigenetic information drives the expression of OR genes [20]. Indeed, the silenced OR *loci* are both tightly heterochromatized and packed in a handful of nuclear *foci* [21]. No genomic rearrangement was found so far to be involved in OR gene regulation [22][23].

Another notable characteristic of most OR genes is their genomic organization: they are found in clusters [11] distributed in around 50 *loci*, with an average intergenic distance of about 25 Kb [24][25]. Only a few OR genes are isolated from the others [26][25] within a region that spans over 1 Mb both upstream and downstream in respect to their transcript's start and end. We term these genes *solitary*.

The genomic clustering of OR genes represents an additional level of complexity in their transcriptional regulation. For instance, it was suggested that some regulatory elements (such as the H [27], P [28] and Lipsi [29] enhancers) control the probability of gene choice for some of the OR genes in the cluster they are proximal to [30][31][29]; those elements also possess TFBSs for HD and O/E proteins [32][28][29]. However, it has also been proposed that elements orchestrate OR gene expression, in addition to their critical *in cis* action modality, via extensive interchromosomal interactions [29]. In addition, the proximity of OR genes in the same cluster could provide some degree of redundancy in the sequences responsible for their regulation.

Our goal is to define and characterize the solitary genes as simplified models for the study of OR gene choice: the information density among regulatory sequences of solitary genes could be higher than the average OR gene, and possible ambiguities in assessing a given regulatory element to a specific OR gene would be avoided.

## Materials and Methods

### Ethics statement

Permissions to catch mouse specimens from the wild were not required at the time of sampling (2005 and 2006). Wild-caught mice were kept in pairs in standard laboratory cages (Type II and III, Bioscape, Germany). In addition to standard bedding (Rettenmaier, Germany) we provided some cage enrichment, i.e. paper stripes, wood wool, a cardboard box and a spinning wheel (Plexx, Netherland). Food (Standard Diet 1324, Altromin, Germany) and water were provided *ad libitum*. Offspring from each mating were weaned at 21 days and transferred to cages according to gender; specimens required for genome sequencing were sacrificed by CO_2_ asphyxiation followed by cervical dislocation. These experimental procedures received approval from the veterinary office of Kreis Plön, Germany. Animal protocol: 1401-144/PLÖ-004697.

Two laboratory mouse strains were used for this study: C57BL/6J (Jackson Laboratory) and *OMP*-*GFP* (see section "5' RACE analyses"). These mice were maintained and bred in our animal facilities in standard laboratory cages (Green Line IVC Sealsafe PLUS Mouse, Italy) with bedding (Nestpak, Grade 6), enrichment items (nesting material and plastic igloos) and different fodder for breeding (Ssniff, V1124-727, M-Z) and maintenance (Ssniff, V1534-727, R/M-H). Food and water were always freely available. Experiments on laboratory mice were approved by the veterinary office of Frankfurt am Main, Germany. For histological experiments, mice were deeply anesthetized with ketamin/xylazin (via intraperitoneal injection) before perfusion (see also section "In situ hybridizations and cell counts"). For 5' RACE experiments, prior to tissue collection mice were euthanized via cervical dislocation. Animal protocols: *Molekulargenetische Untersuchungen des olfaktorischen Systems der Maus*, file F105/Anz.07(36); Perfusion, file F105/Anz.12.

Animal research followed the ethical guidelines of the Max Planck Society, the German animal welfare law (*Tierschutzgesetz* § 11) and the rules of the European directive 2010/63/EU (regulating the protection of animals used for scientific purposes). All mouse handling was carefully performed by qualified personnel in accordance to FELASA guidelines, and all efforts were made to minimize suffering.

### Finding solitary genes and their orthologs

We retrieved a list of *Mus musculus* (house mouse, strain C57BL/6J) GRCm38.p1 genomic coordinates for all OR genes from BioMart [33] at Ensembl [34], and further formatted the data with Galaxy [35] and BEDTools [36]. Then, we defined number and identity of the solitary genes via local scripting. In accordance to our definition, an OR gene was classified as solitary if isolated from other OR genes for more than 1 Mb in respect to their transcripts' genomic coordinates, both upstream and downstream.

Analogous analyses were performed for three other selected Ensembl genomes: *Rattus norvegicus* (brown rat, strain Brown Norway) RGSC 3.4, *Cavia porcellus* (guinea pig, strain 2N) cavPor3 high coverage 6.79X assembly first release, *Homo sapiens* (human) GRCh37. The aim was to understand the conservation of the solitary state among mammalian species. The resulting lists of non-mouse solitary genes were screened for mouse solitary genes orthologs, using orthologous groups found at Inparanoid [37]. We also examined previously described orthologous gene groups (OGGs) of OR genes [38][39][7]: we retrieved all rat, guinea pig and human orthologs from each OGG containing a mouse solitary gene; we BLATed [40] their protein sequence against their appropriate genome (at Ensembl) in order to determine their genomic coordinates, and infer whether they were as well solitary or not.

Chromosome ideograms displaying solitary genes for mouse and rat were obtained by modifying output images from Idiographica [41].

### *In situ* hybridizations and cell counts

The expression of the solitary genes was evaluated by fluorescent *in situ* hybridization (ISH), according to standard laboratory protocol: briefly, C57BL/6J mice were anesthetized via intraperitoneal injection of ketamine HCl and xylazine (respectively, 150 mg/kg body weight and 10 mg/kg body weight), and then transcardially perfused with ice-cold 4% paraformaldehyde in 1% phosphate buffered saline (PBS); their heads were dissected, post-fixed with 4% paraformaldehyde in 1% PBS overnight at 4°C, decalcified overnight with 500 mM EDTA pH 8.0 in PBS (only for adult specimens), cryoprotected first in 15% sucrose and then in 30% sucrose, embedded in optimal cutting temperature compound and stored at -80°C. Coronal 12 μm sections of MOE were produced in a Leica CM3050 S cryostat. Further sample processing and following fluorescent single-color ISHs were performed as previously described [42]. Labelled antisense RNA probes were designed against the 3' part of the coding region of the mRNA of each solitary gene, reaching sometimes the beginning of its 3' UTR; a maximum of 75% of homology with non-solitary OR genes was allowed. GRCm38.p3 genomic coordinates of riboprobes are: chr16:16673002–16673626 for *Olfr19*, chr14:54282002–54282513 for *Olfr49*, chr3:106821836–106822352 for *Olfr266*, chr4:58784988–58785570 for *Olfr267*, chr8:83541574–83542185 for *Olfr370*, chr8:85230725–85231225 for *Olfr371*, chr13:65152575–65153040 for *Olfr466*, chr3:97410406–97411081 for *Olfr1402*. ISH experiments and cell counts were performed on three-day-old mice (n = 3), counting the number of neurons that were expressing every solitary gene in the MOE. Cell counts, meant to provide an indication of the magnitude of the number of neurons expressing each solitary gene in newborn mice, were performed every eighth section on a Zeiss LSM 710 confocal microscope, and the total number of cells was estimated using Abercrombie's method [43]. For those solitary genes not already investigated in Khan *et al*. [44], expression in ten-week-old mice was confirmed by ISH (n = 2).

Single stack images were taken with the same confocal microscope. Adjustments of the original images in size, brightness and contrast and posterior collation and labelling of the final images were done using Adobe Photoshop CS5.1.

### 5' RACE analyses

In order to define transcription start sites (TSSs) and 5'-end splicing of the solitary genes, we performed 5' RACE analyses. Total RNA was extracted as described in Khan *et al*. [31]. Two different types of RNA preparations were used, aiming to maximize either sensibility or specificity in the amplification of mRNA variants for the solitary genes. Accordingly, total RNA sources were either directly the whole olfactory mucosa (WOM), scratched from three-week-old C57BL/6J mice, or purified OSNs. These were obtained via fluorescence-activated cell sorting of WOM extracted from three-week-old specimens of the targeted strain *OMP-GFP*^*+/-*^, whereby an allele of *OMP* (a classic marker gene for mature OSNs) is replaced by the reporter *GFP* [45]. Heterozygote mice for *GFP* were used because the deletion of *OMP* is known to cause alterations in the physiology of the OSN [46]. Mice were euthanized by cervical dislocation and then decapitated. The WOM was exposed by splitting heads in two halves along the medial sagittal plan, and manually scratched out. If used for fluorescence-activated cell sorting, it was further dissected and triturated in a dish filled with Hanks' Balanced Salt Solution (Invitrogen) placed on ice, washed several times with ice-cooled Hanks' Balanced Salt Solution, then threated with dispase/collagenase (Invitrogen) and DNAseI (Roche) under the conditions recommended by the providers; the material was then centrifuged (150 RPM, 20 min, 37°C), the obtained pellet was gently re-suspended in PBS and filtered (pore size 70 μm). To differentially stain damaged cells, propidium iodide (Sigma-Aldrich) was added to the resulting cell suspension. Undamaged, GFP-positive neurons were then sorted on a fluorescence-activated cell sorter (JSAN Desktop Cell Sorter) and immediately used for RNA extraction.

Coordinates of TSSs were determined by 5' RACE (Clontech's SMARTer^TM^ RACE Kit): 5' RACE-ready cDNA was obtained from RNA samples (10–140 ng), and DNA libraries were generated via nested PCR with gene-specific reverse primers followed by electrophoresis on 1% agarose gel and recombination of the 5' RACE-gel purified bands with pGEM^®^-T Easy Vector (Promega). Primer sequences are reported in Table 1. DNA libraries were transformed into chemically competent DH5α *Escherichia coli*, and plasmid DNA from some of the resulting colonies was purified and Sanger-sequenced. The obtained sequences were polished from vector/primer backbone via local scripting and aligned against their genomic reference (GRCm38.p1) on MacVector [47].

**Table 1:** Gene-specific 5' RACE primers.

Gene	PCR primer	Nested PCR primer
***Olfr6* (*M50*)**	CCAGCCAGCATCTTAGGGACAGTGAC, GCCCGAGGTCGGACATACATGAAGA	TAGGCTTGTGCAGTGACCCAGTGACC
***Olfr19* (*M12*)**	CAGATGTCTGCAAAGGACAGGTTGGA	TGTCTGCAAAGGACAGGTTGGAAAGGA
***Olfr49***	GGGGAAGATGACAGAGGTGAACCAGA	GCATGGCAAAGTTGCGGAGGAAGTAA
***Olfr266***	GCAGACCTCCAGAAAGGACAGGTTCA	TGTGAAGAGCATGGTTGCACAGCACT
***Olfr267***	GGACAGGTACATCTGCACAGCACACC	TTTGAAGGCGTGAATCCAGGACAGTG
***Olfr370***	AGGGCTCTGTCTAGCACAATGGTGGA	CAGCCAGAGACGAGAAGCCAAGGAAG
***Olfr371***	CAGGATGATGAGCAGGTTCCCAAAGA	CAAGAACAGCCCAAAGAGAACGGACTG
***Olfr466***	GACAGCATTCACTGCACCCCATGAA	TCATGCCAAAATTCCCCACCACTG
***Olfr1402***	TGTGTTCCCCAGGAGAGTTGTCAGGT	CCCTGTAGGTTGGGGTGGTTGGAAAA

Gene-specific primers (5'-3') used for 5' RACE experiments. PCR primer: gene-specific primers used for the generation of the initial 5' RACE amplicons. Nested PCR primer: gene-specific primers used for the amplification of the final 5' RACE fragments. For *Olfr6* (*M50*) two different PCR primers were tested.

Similarly to previous studies, e.g. Vassalli *et al*. [17], we arbitrary define a putative minimal promoter as a sequence of 301 bp centered on the most upstream TSS of each solitary gene. Positions of the TSSs were compared to previous reports in literature [19][48][18]. We used as a control the non-solitary OR gene *Olfr6*, also known as *M50*, chosen for its characterized gene structure [16].

### Prediction of TFBSs on putative promoters

In order to determine sequence conservation, the newly defined promoter regions were compared via T-Coffee [49] at MacVector. Also, we looked for conserved stretches over candidate promoter sequences among Mammalia Eutheria, using the annotation track “36 Eutherian Mammals EPO Low Coverage” [50][51] found at Ensembl.

Using the software MEME [52] from the MEME Suite [53], a position-specific weight matrix (PSWMs) for HD transcription factors was derived from the TFBSs found on the P and H elements based on Vassalli *et al*. [54]. TFBS provided by Vassalli *et al*. were extended, and their orientation was checked, via EMBOSS [55] Palindrome. For the obtained PSWM, occurrences of TFBSs on the putative promoters were predicted using MEME Suite’s FIMO [56].

### Comparison of putative promoters with their wild-type counterparts

We compared the C57BL/6J sequence of the putative promoter of a solitary gene to sequences obtained from natural populations of mice from the subspecies *M*. *m*. *domesticus*. The goal was to understand whether the C57BL/6J inbred strain could display, in lack of selective pressure, sequence variations among the defined putative promoters.

Specimens were collected in the wild in three different locations in Germany, France and Iran, employing a sampling regime that eliminates the collection of related animals in one location. Individuals from France were collected in 2005, while samples from Germany and Iran were collected in 2006. Every location was covered by not less than eight trapping sites, each one at least 300 m apart. Trap sites in France, Germany and Iran were located, respectively, within a 100 Km radius around the *commune* of Sévérac-le-Château (44.322743°N, 3.072680°E), the city of Bonn (50.73350°N, 7.08687°E) and the city of Ahvaz (31.38097°N, 48.68116°E). More precisely, individual geographic coordinates of capture sites are: 44.341545°N, 3.035541°E; 43.315587°N, 3.138231°E; 44.345711°N, 3.060376°E; 44.345711°N, 3.060376°E; 44.342103°N, 3.034077°E; 44.311592°N, 2.999890°E; 44.345711°N, 3.060376°E; 50.43211°N, 6.55282°E; 50.40954°N, 6.56990°E; 50.36671°N, 7.02719°E; 50.36693°N, 7.05283°E; 50.39384°N, 7.07288°E; 50.631867°N, 7.154070°E; 50.645037°N, 7.191881°E; 50.748106°N, 6.869173°E; 50.651406°N, 6.984778°E; 50.841892°N, 6.732841°E; 50.47599°N, 6.52153°E; 31.52739094°N, 49.74733679°E; 31.53741427°N, 49.70954086°E; 31.50942081°N, 49.84584385°E; 31.38692616°N, 49.84893333°E; 31.23319452°N, 49.24533119°E; 31.38096973°N, 48.68116080°E; for one site in France and three in Iran detailed coordinates were not recorded. In order to set up mating pairs, we attempted to catch one male and one female mouse at each trap site. For some trap sites this was not possible: in these cases, we set up mating pairs by selecting two individuals of opposite sex from nearby trap sites. Single F1 males from most mating pairs were chosen as samples for sequencing. In rare occasions, we directly used a wild caught mouse. Sample mice were euthanized by CO_2_ asphyxiation followed by cervical dislocation at age nine-twelve weeks. In total, each location (France, Germany and Iran) was represented by eight individuals for whole genome sequencing.

For whole genome sequencing we fragmented 1 μg of DNA of each of the 24 individuals with sonication technology (Bioruptor, Diagenode, Liège, Belgium). The fragments were end-repaired and adaptor-ligated, including incorporation of sample index barcodes. The products were then purified and amplified (10 PCR cycles) to create the final libraries. The TruSeq^®^ DNA LT Sample Prep Kit v2 was used for all steps. After validation (Agilent 2200 TapeStation), all libraries were quantified using the Peqlab KAPA Library Quantification Kit and the Applied Biosystems 7900HT Sequence Detection System. One library was loaded on two lanes of a Hiseq2000 sequencer and sequenced with a 2x100 bp v3 protocol.

We used BWA-MEM [57] to align raw reads to the mouse reference genome (GRCm38.p3) and extracted reads that overlapped the solitary gene *loci* from the resulting BAM file. Those reads were then realigned to reference and manually inspected in MacVector, first all at the same time and then separated according to provenience. Putative promoter consensus sequences from France, Germany, Iran and all locations together were obtained, using a 51% consensus calculation threshold. Those sequences were then multi-aligned with ClustalW [58] at MacVector, together with their counterparts in C57BL/6J and in 36 additional laboratory strains provided by the Wellcome Trust Sanger Institute [59][60][61][62][63][64][65]. We here call *mutant* a C57BL/6J putative promoter if all its wild-type homologous sequences are locally different from the C57BL/6J variant; any other variation, in wild populations and/or in laboratory strains, would qualify a putative promoter as *polymorphic*.

Finally, to provide a global measure of conservation for every putative promoter, we built a neighbor-joining tree (using MacVector) for each multi-alignment. An average pairwise distance representing variation was assigned to every candidate promoter; such quantity was calculated as the mean Tamura-Nei genetic distance [66] between every possible couple of aligned sequences, found on the distance matrix of each phylogenetic tree.

## Results and Discussion

### Solitary genes and their orthologs

**Fig 1:**
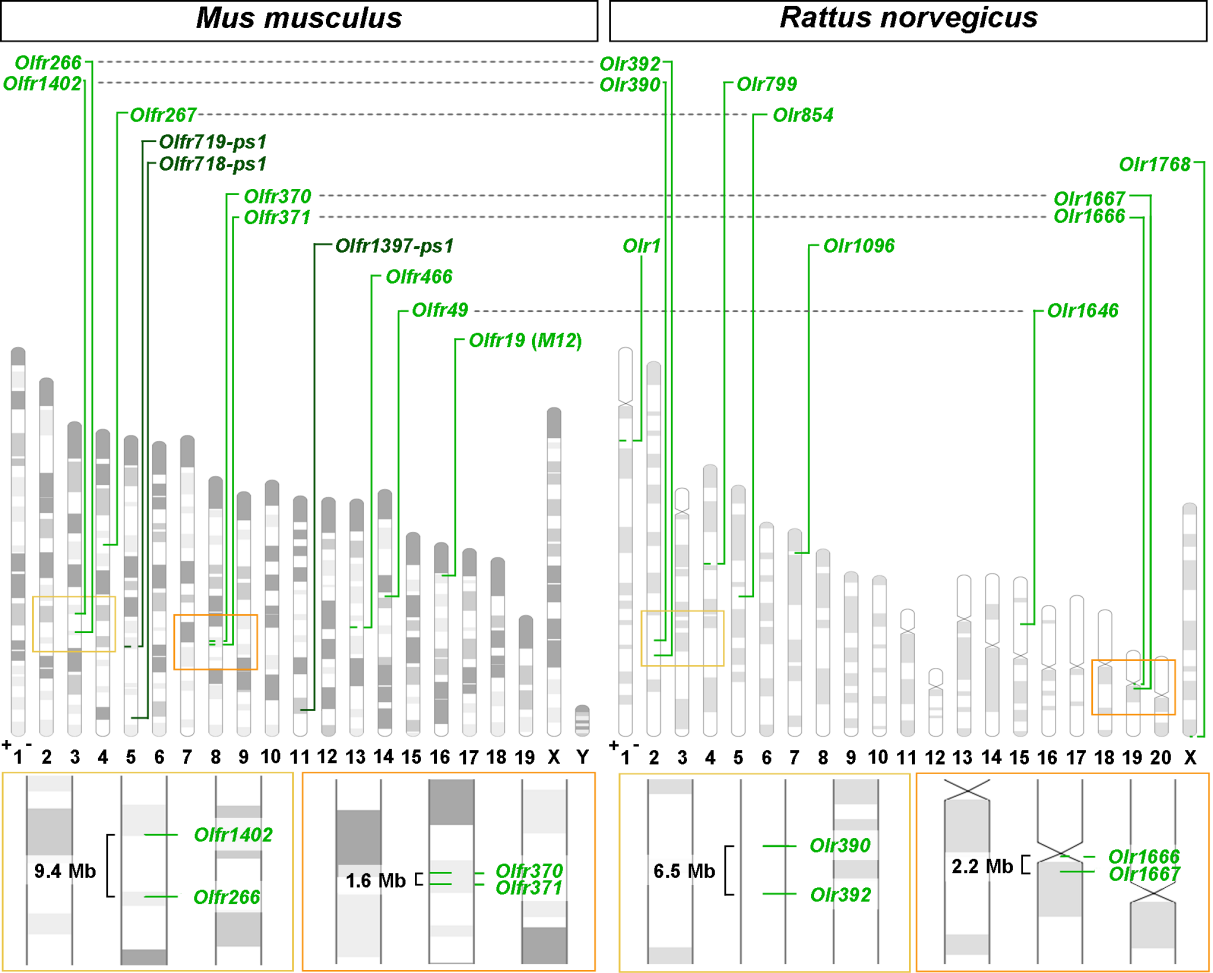
Chromosomal distribution of the solitary genes in Muridae. Solitary genes for *Mus musculus* (GRCm38.p1) and *Rattus norvegicus* (RGSC 3.4) are indicated in light green if intact, in dark green if pseudogenized. Grey dashed lines connect each solitary gene with its ortholog. Yellow and orange squares, amplified below the chromosomal diagram of the corresponding species, show pairs of solitary genes that (based on annotation) retain a conserved synteny in Muridae (while a non-annotated, neighboring odorant receptor gene is present for *Olr390*). The distance (measured in Mb) between genes of each gene pair is annotated. Chromosome bands represent Giemsa staining.

The Ensembl genomes used for comparison with C57BL/6J were chosen according to their annotation state and phylogenetic distance from the mouse: the rat from the same family (Muridae), the guinea pig from the same order (non-Muridae Rodentia) and the human from the same class (non-Rodentia Mammalia). For all the investigated genomes, the bioinformatic work resulted in the identification of solitary genes (Table 2 and Fig 1). There are eight intact solitary genes in the mouse genome: *Olfr19* (*M12*), *Olfr49*, *Olfr266*, *Olfr267*, *Olfr370*, *Olfr371*, *Olfr466*, *Olfr1402* (Table 2 and Fig 1). In addition, there are three solitary pseudogenes: *Olfr718-ps1*, *Olfr719-ps1*, *Olfr1397-ps1*. Since our work is focused on the definition of a suitable model gene for the study of the OR gene choice, we did not further investigate pseudogenes. Curiously, four out of the total eight solitary genes are genomically arranged as pairs: *Olfr266* neighbors *Olfr1402* (distance 9.4 Mb), and *Olfr370* neighbors *Olfr371* (distance 1.6 Mb). The most detached gene is *Olfr466*, isolated upstream and downstream in respect to the genomic coordinates of its transcript within a genomic interval of 43.4 Mb.

**Table 2:** Solitary genes in four mammalian species.

Species	Gene	MDAS
***Mus musculus***	*Olfr19 (M12)*	2.6
	*Olfr49*	1.7
	*Olfr266*	9.4
	*Olfr267*	5.8
	*Olfr370*	1.6
	*Olfr371*	1.6
	***Olfr466***	43.4
	*Olfr1402*	9.4
***Rattus norvegicus***	*Olr1*	1.5
	*Olr390*	6.5
	*Olr392*	6.5
	*Olr799*	2.0
	*Olr854*	4.4
	*Olr1096*	2.0
	*Olr1646*	3.1
	*Olr1666*	1.6
	*Olr1667*	1.6
	***Olr1768***	10.0
***Cavia porcellus***	*OR1I1*	Alone in genomic scaffold
	*OR1M1*	Alone in genomic scaffold
	*OR1N1*	Alone in genomic scaffold
	*OR2AT4*	3.5
	*OR2C1*	Alone in genomic scaffold
	*OR2K2*	Alone in genomic scaffold
	*OR2T11*	1.0
	*OR2Y1*	Alone in genomic scaffold
	*OR2Z1*	Alone in genomic scaffold
	*OR4B1*	Alone in genomic scaffold
	*OR4C5*	1.1
	*OR4C6*	1.3
	*OR4C11*	Alone in genomic scaffold
	*OR4C12*	Alone in genomic scaffold
	*OR4D1*	Alone in genomic scaffold
	*OR4X2*	Alone in genomic scaffold
	*OR5AS1*	Alone in genomic scaffold
	*OR5D16*	Alone in genomic scaffold
	*OR5I1*	1.4
	*OR5K4*	Alone in genomic scaffold
	*OR8B8*	1.0
	*OR10AG1*	Alone in genomic scaffold
	*OR13A1*	Alone in genomic scaffold
	*OR51G11*	3.6
	*OR52B2*	1.7
***Homo sapiens***	*OR2A4*	38.9
	*OR2AE1*	38.9
	*OR2K2*	6.6
	*OR4F17*	8.7
	*OR4F21*	6.9
	***OR11H1***	Alone in chromosome 22

Intact (according to Ensembl Biomart) solitary genes. MDAS: maximum distance as solitary (measured in Mb), the genomic interval in which the odorant receptor gene is isolated, both upstream and downstream in respect to its transcript coordinates, from neighboring odorant receptor genes. For *Cavia porcellus*, the average genomic scaffold length is 8.9 Mb. Most isolated genes in each species analyzed have gene names highlighted in bold.

Following BioMart annotation, in the rat genome we identified ten solitary genes (Table 2 and Fig 1). For the guinea pig, we found as many as 25 solitary genes (Table 2); we believe that, due to the relatively poor annotation status of cavPor3 (compared to the other genomes studied) and the segmentation of the genome in scaffolds rather than in chromosomes, such number is likely to be overestimated. The investigation of OGGs led to the identification of a non-annotated neighboring OR gene in the rat for *Olr390*, which therefore is not solitary, and to the finding of an additional, unnamed intact solitary gene for the guinea pig (S1 Table).

The OR gene repertoire of the human genome is strongly pseudogenized [67][68]; however, we could still locate six intact solitary genes (Table 2). Notably, one of them (*OR11H1*) is the only OR gene located on chromosome 22.

When we compared the conservation of the solitary status among the genomes studied (Table 3 and Fig 1, see also S1 Table), we found that as much as five out of eight mouse solitary genes have solitary orthologs in rat. In addition, a non-annotated ortholog of *Olfr19*, despite not being strictly solitary, is still remarkably isolated (0.6 Mb). Furthermore, the genomic organization of the pair *Olfr370*/*Olfr371* is retained: the correspondent pair of solitary orthologous genes is *Olr1667*/*Olr1666*. Conservation drops outside of the Muridae lineage: in both guinea pig and human, three out of the eight solitary genes of the mouse have solitary orthologs. The solitary status of *Olfr266* and *Olfr267* is noteworthy; in fact, both genes belong to OGGs made by a single, solitary gene in each of all selected species.

**Table 3:** Conservation of the solitary status in orthologous genes of the mouse solitary genes.

*Mus musculus*		*Rattus norvegicus*		*Cavia porcellus*		*Homo sapiens*	
Gene	MDAS	Ortholog	MDAS	Ortholog	MDAS	Ortholog	MDAS
*Olfr19* (*M12*)	2.6	RanoOR12.2.1P^a^	0.6^b^	-	-	*OR7A19P*	38.1
*Olfr49*	1.7	***Olr1646***	3.1	-	-	-	-
*Olfr266*	9.4	***Olr392***	6.5	**ENSCPOG00000023753**	10.1	*OR11I1P*	36.5
*Olfr267*	5.8	***Olr854***	4.4	***OR2K2***	Alone in scaffold_93^c^	***OR2K2***	6.6
*Olfr370*	1.6	***Olr1667***	2.1	-	-	-	-
*Olfr371*	1.6	***Olr1666***	2.1	-	-	-	-
*Olfr466*	43.4	-	-	-	-	-	-
*Olfr1402*	9.4	-	-	CapoORs5.1P^a^	Alone in scaffold_5^c^	-	-

The solitary genes and their solitary orthologs in selected mammalian species. For each solitary gene in *Mus musculus*, the table lists all orthologous genes (following Niimura *et al*.) that are found to be solitary in the other studied species. In bold are reported those genes recognized as orthologs also by Inparanoid. Ortholog: solitary orthologous gene. MDAS: maximum distance as solitary (measured in Mb), the genomic interval in which the odorant receptor gene is isolated, both upstream and downstream in respect to its transcript coordinates, from neighboring odorant receptor genes.

^a^ In absence of both an official name and an Ensembl gene identifier, we reported the identifier provided by Niimura *et al*. [7].

^b^ While reported for convenience, RanoOR12.2.1P is not strictly solitary according to our 1 Mb threshold criterion.

^c^ For *Cavia porcellus*, the average genomic scaffold length in the cavPor3 genome assembly is 8.9 Mb.

### Expression of the solitary genes in the MOE

A deep characterization of the expression pattern of the solitary genes is beyond the scope of our study. Here we investigated the expression of the solitary genes in the MOE for three-day-old mice, and we provided as well an overview of the number of OSNs expressing each solitary gene. To understand whether the solitary genes could recapitulate the basic features of OR genes, we aimed also to supply evidence of their punctate expression in the MOE, hallmark of OR gene choice. In a previous report [44], all solitary genes but *Olfr1402* were investigated for mRNA expression by NanoString analysis and showed expression in the MOE of 2, 6, 12, 18 or 31-month-old C57BL/6 mice: in three-week-old mice mRNA from sorted OSNs for *Olfr19*, *Olfr49*, *Olfr466* and *Olfr370* is relatively well represented; *Olfr266*, *Olfr267* and *Olfr371* possess very low amounts of mRNA. As the mouse ages, expression levels for the solitary genes either drop (*Olfr19*, *Olfr49*, *Olfr370*) or are substantially maintained (*Olfr266*, *Olfr267*, *Olfr371*, *Olfr466*). Moreover, *Olfr19* is expressed in the dorsal MOE [69], *Olfr49* is expressed within a patch-like pattern [70], and *Olfr370* is expressed in the medial MOE and in the septal organ [71].

**Fig 2:**
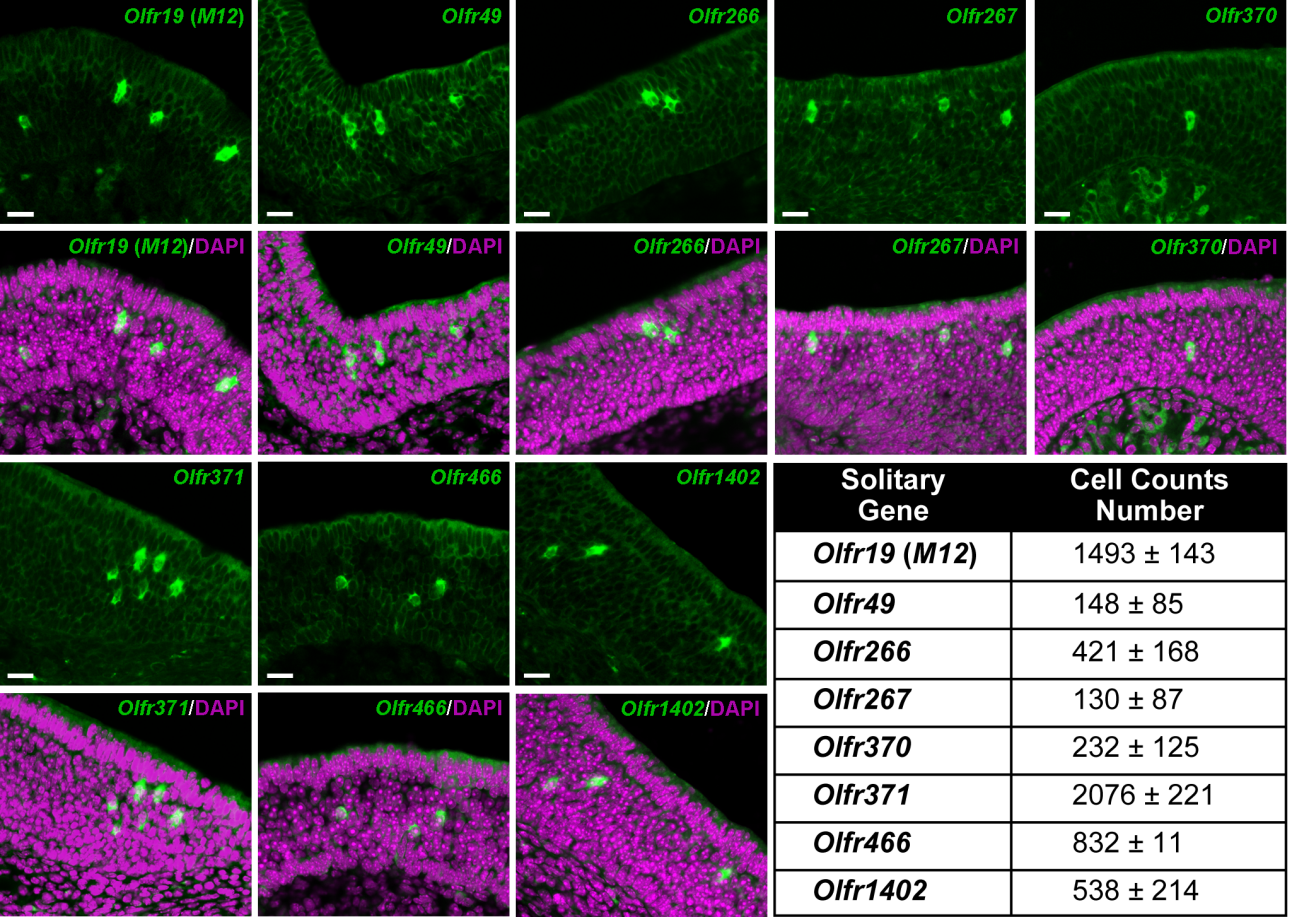
Solitary genes expressed in the mouse main olfactory epithelium. *In situ* hybridization of the main olfactory epithelium of three-day-old mice (n = 3), showing the expression of the eight mouse solitary genes: *Olfr19* (*M12*), *Olfr49*, *Olfr266*, *Olfr267*, *Olfr370*, *Olfr371*, *Olfr466* and *Olfr1402* (green). Nuclear counterstaining is with DAPI (magenta). The table on the right reports the average number of olfactory sensory neurons (± a standard deviation) for each solitary gene. Scale bars: 20 μm.

Our ISH experiments showed that in the MOE of three-day-old mice all solitary genes are already expressed, and in the punctate fashion typical of OR genes. For *Olfr1402*, expression was confirmed in ten-week-old mice (S1 Fig). *Olfr19* and *Olfr371*, in respect to the other solitary genes, are expressed in the highest numbers of OSNs in newborn mice; the lowest numbers are found for *Olfr266*, *Olfr370*, *Olfr1402*, and especially *Olfr49* and *Olfr267*. The average cell count value for *Olfr466* is intermediate. Fig 2 shows DIG-labeled OSNs expressing the solitary genes and reports their cell counts. To evaluate dispersion of our cell counts, we calculated the coefficient of variation (CV), defined as the ratio between a standard deviation over its arithmetic mean. We found values ranging from 0.669 (for *Olfr267*) to 0.013 (for *Olfr466*), and a negative correlation (r = -0.8145, p = 0.013926) between average and CV (S2 Fig). For comparison, another study [72] counted OSNs expressing the non-solitary OR gene *Olfr17* (also known as *P2*) in 3.5-day-old mice, counting in each section. They reported a mean of 1474, with a CV that can be estimated as 0.1. Similarly, averages for *Olfr19* and *Olfr371* amount respectively to 1493 and 2076; their CVs are correspondingly equal to 0.096 and 0.106.

A thorough analysis of OSN counts for several OR genes in three-week-old mice [73] recently showed how sampling methods can affect standard deviation. However, they found no correlation between average and CV: whether this disagreement reflects a peculiarity of solitary genes or not remains to be investigated.

### Putative promoters for the solitary genes

**Fig 3:**
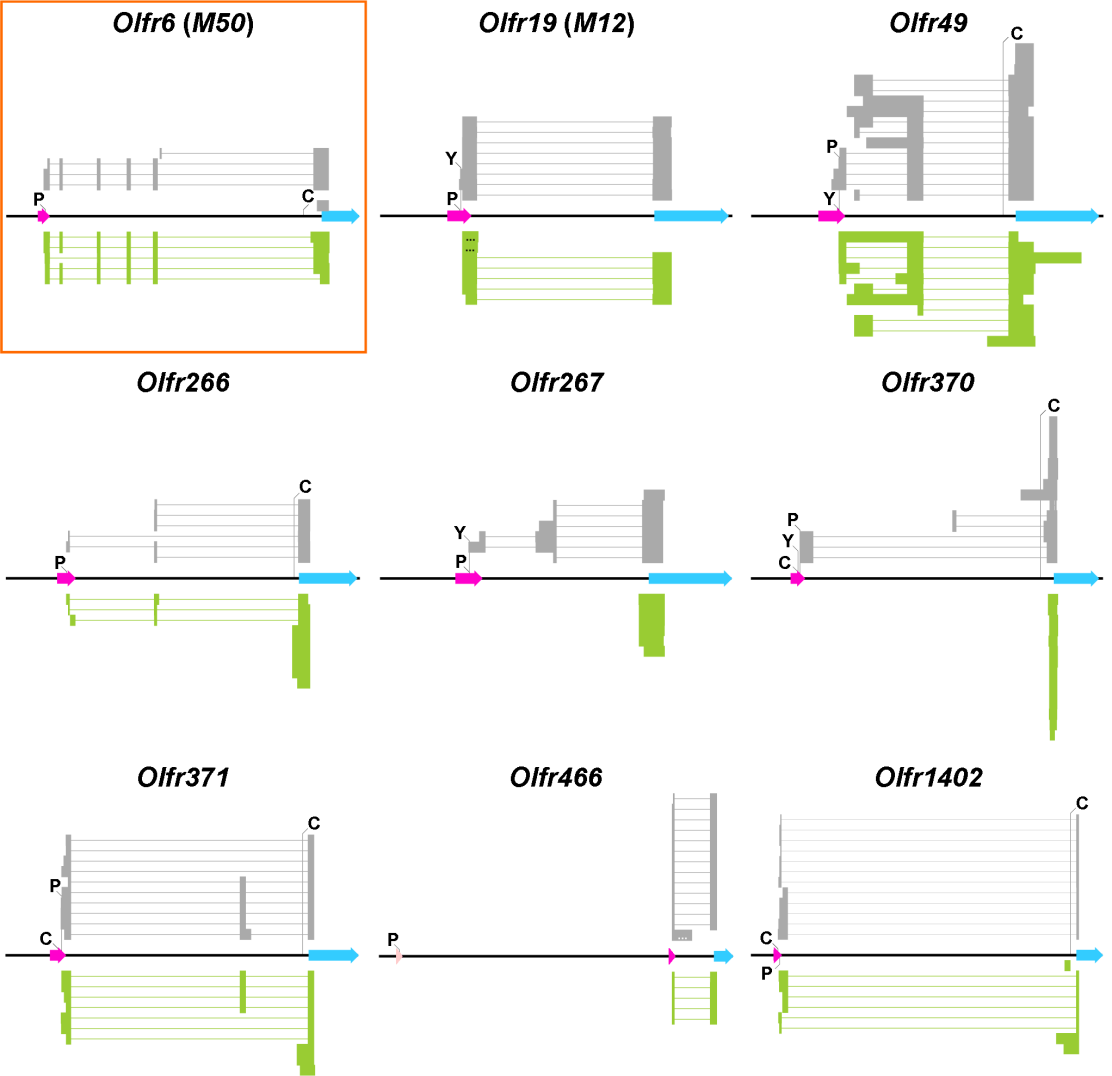
5' RACE clones aligned against their genomic references. For each gene, middle black line represents the genomic *locus* (GRCm38.p1). Transcription start sites derived from literature are labelled with capital letters (C for Clowney *et al*., P for Plessy *et al*., Y for Young *et al*.). Light blue arrows depict coding sequences as they appear on Ensembl. Fuchsia arrows represent sequences selected as putative promoters. On *Olfr466*, a pink arrow renders an alternative putative promoter derived from Plessy *et al*. All arrows point towards 3'-end. Above genomic *loci*, grey stacked lines represent spliced 5' RACE clones obtained from whole olfactory mucosa; similarly, in green below the genomic reference, clones derived from sorted olfactory sensory neurons are depicted. Thin (grey or green) lines in 5' RACE clones represent introns. Three dots (…) on a stacked line indicate that the clone is not sequenced until its 3'-end. Shortest clones are omitted for graphical reasons. The control, non-solitary, odorant receptor gene *Olfr6* (*M50*) is boxed in orange.

Overall, 5’ RACE analyses (S1 File) corroborated literature data (Fig 3): from both WOM and sorted OSNs, TSSs positions were substantially confirmed for the reference gene *Olfr6* and the solitary genes *Olfr19*, *Olfr49*, *Olfr266*, *Olfr371*, *Olfr1402*; for *Olfr266* and *Olfr371* we could confirm TSSs positions only when amplifying cDNA from WOM. *Olfr466* has a TSS shifted approximately 13 Kb downstream in respect to Plessy *et al*. [19]. In addition, more recent RNA-Seq data on WOM and sorted OSNs [74][75][76] provide further terms of comparison. For instance, detailed analyses on the dataset from Ibarra-Soria *et al*. highlighted very good accordance with our findings: all most 5'-located TSSs are essentially confirmed but for *Olfr266*, which has a TSS also found by us but more 3'-located, and *Olfr370*, for whom a TSS is not provided. Generally, gene structures are in close accordance too.

The non-solitary OR gene *Olfr6*, as previously described [16][74], displays mostly a six-exon structure, which is unusual for an OR gene [15][16][75]. We also found spliced variants that skip exon 2, and a clone with a TSS located after exon 5. *Olfr19* and *Olfr1402* have a simple two-exon structure. *Olfr49* and *Olfr371* have clones with three exons and clones with two exons: in the second case, the middle exon is skipped (*Olfr49*, *Olfr371*), or the first intron in respect to three-exon clones is retained (*Olfr49*); moreover, the position of TSSs for *Olfr49* is variable within a few hundred of base pairs. *Olfr266*, *Olfr267* and *Olfr370* have either a three-exon or a two-exon structure, the latter skipping the second exon (*Olfr266*, *Olfr370*) or the first one (*Olfr266*, *Olfr267*, *Olfr370*); this asset is compatible with the presence of two alternative promoters. For *Olfr370*, we found a clone with a short intron (21 bp, starting exactly from the start codon) on the coding sequence, a situation already described for a few other OR genes [74]. *Olfr466* displays a two-exon structure but, as mentioned above, we could not confirm the TSS proposed by Plessy *et al*. On the other hand, both WOM and sorted OSNs-derived clones, together with the data from Ibarra-Soria *et al*., sharply identify a different TSS. We decided to consider for further analyses both the 301 bp putative promoter centered on the 5’ RACE-derived TSS and the one centered on Plessy *et al*.'s.

Our work focused on the splicing of 5'-located genic regions. However, the vast majority of OR genes do not possess 3' UTR introns [15][75].

### A candidate HD binding site on *Olfr266* putative promoter

**Fig 4:**
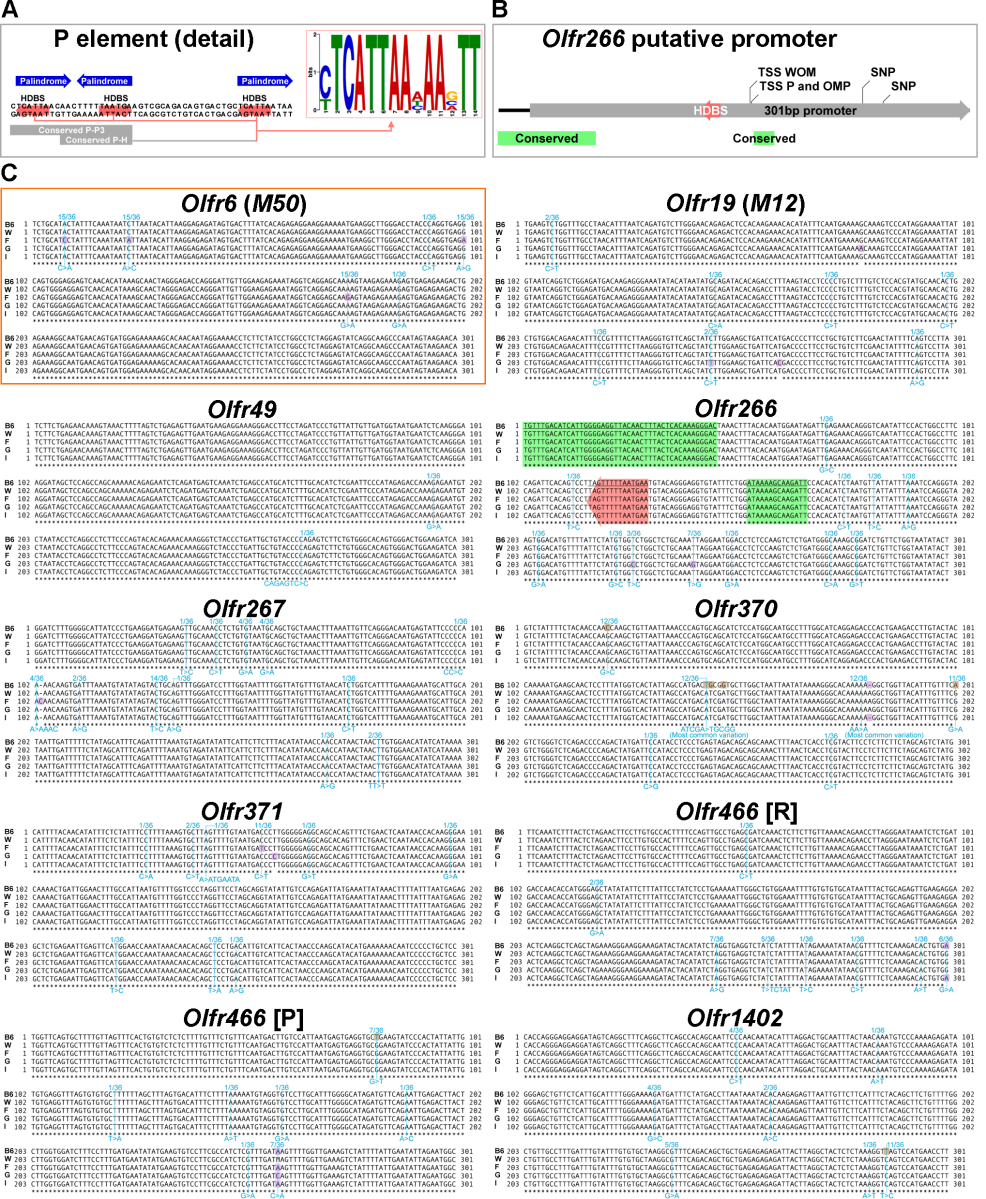
Candidate promoter sequences of the solitary genes. A: detail of the P element showing the sequences used to generate the position-specific weight matrix for homeodomain factor binding sites; on the main sequence, 3'-pointing arrows in salmon show position and orientation of homeodomain factor binding sites as annotated by Vassalli *et al*.; blue arrows, also 3'-oriented, show emipalindromes identified by EMBOSS Palindrome and used to extend sequences to be added to the matrix; grey boxes mark sequence stretches conserved between the P element and the neighboring *Olfr713* (*P3*) promoter (Conserved P-P3) or between the P and the H element (Conserved P-H); a matrix (depicted on the right, top) was obtained aligning the three extended binding sites on P element and the single extended binding site on H element (identical to the central binding site on P element). B: on the putative promoter of *Olfr266* (3'-pointing grey arrow) a homeodomain factor binding site (3'-pointing salmon arrow labelled as HDBS) is predicted; also, sequences conserved in Mammalia Eutheria are found (green boxes); more towards the 3'-end of the putative promoter, SNPs that are present in the wild for *Mus musculus domesticus* are found in two positions. C: multi-alignments of candidate promoter sequences (5'-3') from different mouse populations for the solitary genes plus (orange box) the non-solitary, reference odorant receptor gene *Olfr6* (*M50*); for *Olfr466* two different sequences are presented, one 5' RACE-derived (tagged with [R]) and one based on Plessy *et al*. (labelled with [P]); B6 stands for C57BL/6J, W stands for consensus of all wild-caught populations, F stands for French population, G stands for German population and I stands for Iranian population; in brown are marked mutant C57BL/6J bases; in violet are highlighted bases differing from consensus; thin vertical lines (light blue) on sequence alignment mark the position of variations in at least some of the 36 non-C57BL/6J laboratory strains, reporting also the fraction of strains carrying the variation (above) and the variation details (below); for *Olfr266*, sequences boxed in green are conserved in Mammalia Eutheria, and bases in arrowed salmon box (arrow points towards 3') are part of the predicted homeodomain factor binding site.

The multi-alignment of the defined putative promoters did not highlight any obvious sequence conservation (data not shown). We obtained a PSWM from the extension of TFBS for HD transcription factors on regulatory elements (Fig 4A). The FIMO search for TFBSs revealed a statistically supported HD binding site on *Olfr266* putative promoter, which is also found to be conserved in 36 Mammalia Eutheria species; more in detail, there are two conserved stretches, a longer one (45 bp) spanning across the 5'-end of the promoter and reaching further 5', and a shorter one (14 bp) located just a few base pairs 3' from the TSS (Fig 4B). Provided that possible follow-up promoter studies would be at the same time expensive and time-consuming, our TFBS searches were strictly designed to control the false positive rate, rather than false negative rate: a single PSWM is likely to lack information for the systematic identification of single TFBS occurrences, and the false discovery rates implemented by FIMO are context-dependent [56]; in addition, our PSWM was solely designed based on elements known to regulate *in cis* multiple OR genes. Therefore, our strategy specifically selects for TFBSs with remarkable sequence similarity with those found on P and H elements.

### Variations in putative promoters among inbred and wild-caught mice

The olfactory system has probably a reduced importance for the fitness of the mouse in the laboratory environment: even though the sense of smell as a whole is required for the life of laboratory mice [77], there is no need to think that their OR gene repertoire evolves exactly as if they were in the wild. Indeed, in contrast with captive colonies, mortality in wild populations is often a consequence of predation and starvation, cf. [78]. This would further support the fact that negative selection still concurs substantially in shaping the evolutionary dynamics of OR genes in the wild [79][7]. Consequently, OR alleles that are normally deleterious in a wild habitat might be fixed at least with comparatively higher frequencies by genetic drift during the process of inbreeding in a mouse facility. Only those OR *loci* that an inbred line shares with its wild ancestor represent variants relevantly exposed to purifying selection. We chose to investigate wild populations of *M*. *m*. *domesticus* because this subspecies is the main contributor to the creation of the domestic mouse [80].

The use of inbred lines for animal research, in spite of numerous advantages, presents indeed some limitations [81], especially when features with likely reduced importance for the life of captive individuals are investigated. For instance, the model OR gene *Olfr151* (better known as *M71*) is pseudogenized in C57BL/6J, cf. Feinstein *et al*. [82]. Hence, evaluating sequence conservation in wild-caught mice could contribute to the definition of a suitable OR gene for the study of OR gene expression: polymorphisms on wild-type putative promoters may hint to a reduced functional relevance, whilst mutations on C57BL/6J rise concerns about the possibility of accidental fixation of a null allele. Therefore, genes with mutant C57BL/6J putative promoters would be considered worse candidates for the study of OR gene choice: in other words, investigating allelic variants is a way to validate functional integrity of promoters and their parts: a sequence that is present in wild populations has higher chances to be normal and, consequently, becomes more desirable as a tool to understand how OR gene choice is achieved; ideal models should at least have one wild-type sequence locally identical to the C57BL/6J allele. Minor polymorphisms, especially among wild populations, would be taken on the contrary as an additional value, as they might provide information concerning locally decreased functional importance of the sequence. Finally, the incorporation of 36 additional laboratory strains in the study helps us gaining a more complete insight of sequence variation. For instance, a mutant C57BL/6J variant would be considered as more tolerable if present in most laboratory strains.

The multi-alignment of wild-type and laboratory sequences (S2 File) highlighted some variations (Fig 4C). According to our definitions, all putative promoters display some degree of polymorphism, and eight (all but those of *Olfr49* and *Olfr1402*) are polymorphic in wild-caught mice too; three of them are also mutant in C57BL/6J: these are the 5' RACE-derived candidate promoters for *Olfr370* (with as much as 5 bp conserved in wild mice but not in C57BL/6J) and *Olfr1402*, as well as the putative promoter for *Olfr466* obtained from Plessy *et al*. The candidate promoter of *Olfr49* is identical for all wild populations, and is also entirely conserved in all laboratory strains (including C57BL/6J) except SPRET/EiJ. The conserved stretches and the putative HD binding site on the *Olfr266* sequence are also intact in both wild-caught mice and laboratory strains. To measure overall conservation we evaluated the average pairwise distance. Ranking the studied genes according to crescent conservation of their putative promoter sequences results in the following list: *Olfr370*, *Olfr6*, *Olfr1402*, *Olfr267*, *Olfr266*, *Olfr466* (derived from Plessy *et al*.), *Olfr466* (5' RACE-derived), *Olfr371*, *Olfr19* and *Olfr49*. This further confirms the candidate promoter for *Olfr370* as the most varied, and the one for *Olfr49* as the most conserved.

Generally, the studied sequences from *M*. *m*. *domesticus* from Iran proved to be the most similar to their homologous C57BL/6J references (totally, 7 bp mismatch). Given the mixed Asian origin of the C57BL line [83], this is not surprising. Instead, the German population is the one with the highest number of variations from C57BL/6J (16 bp are mismatched in total).

## Conclusions

There are complications in studying the regulation of expression of OR genes that belong to clusters. As robustly showed in transgenic mice [84][17][85], sequences proximal to a given OR gene can also have a role in the regulation of nearby or even distant members of its cluster. Ultimately it may not be possible to study unambiguously the regulatory sequences of a single OR gene that belongs to a cluster. For instance, some redundancy in their regulatory sequences has been supported by the fact that if the promoter of the clustered model OR gene *Olfr151* is altered, the observed phenotype is milder in respect of the very same mutation on the promoter of an *Olfr151* transgene. The presence of multiple regulatory sequences would reinforce the clustering of OR genes by providing some functional redundancy [85]. We thus recognize in the long run the need for new and simpler model systems for the study of OR gene expression. Theoretically, it is still arguable that interchromosomal interactions could provide regulatory features to a solitary gene; in fact, based on cytogenetic studies regarding chromatin organization in OSNs, the H element was proposed to regulate *in trans* OR genes [86]. Later on the idea was debated: gene expression analyses showed that only OR genes residing *in cis* with the H element are affected by the targeted deletion of the enhancer [30][31]. However, further cytogenetic and molecular evidence suggested that the *in trans* action of OR gene-regulating elements is probably present but in a highly redundant fashion [29], meaning that a chromatin bundle of enhancers as a whole is required for the expression of OR genes. The *in trans* function of a single enhancer on OR gene regulation would be therefore mostly dispensable. On the other hand, whether solitary or not, each single OR gene has to retain a battery of nearby sequences allowing for choice and expression. For OR genes residing in genomic clusters this set is likely shared, at least partially, between cluster members; but solitary genes must possess unambiguously dedicated regulatory features, capable of driving the punctate expression distinctive of OR genes. Research with the current model systems has yielded valuable insights but, eventually, the identified regulatory sequences cannot be linked unequivocally to a single OR gene. It is somewhat surprising that there has been no attention thus far directed to the solitary genes.

With this work we located the mouse solitary genes and analyzed them in order to define the best candidate for studies aiming to address the molecular bases of the OR gene choice. By evaluating all different aspects together we can now select the best model genes (Table 4).

**Table 4:** Measurable features of the solitary genes.

Gene	Cell counts	MDAS	Solitary ortholog(s) MDAS	Inbred putative promoter	Predicted HDBS
***Olfr19* (*M12*)**	1493 ± 143	2.6	0.6^a^ [Rn], 38.1 [Hs]	Polymorphic	No
***Olfr49***	148 ± 85	1.7	3.1 [Rn]	Polymorphic	No
***Olfr266***	421 ± 168	9.4	6.5 [Rn], 10.1 [Cp], 36.5 [Hs]	Polymorphic	Yes
***Olfr267***	130 ± 87	5.8	4.4 [Rn], Alone in scaffold_93^b^ [Cp], 6.6 [Hs]	Polymorphic	No
***Olfr370***	232 ± 125	1.6	2.1 [Rn]	Mutant	No
***Olfr371***	2076 ± 221	1.6	2.1 [Rn]	Polymorphic	No
***Olfr466***	832 ± 11	43.4	-	Polymorphic [R], Mutant [P]	No
***Olfr1402***	538 ± 214	9.4	Alone in scaffold_5^b^ [Cp]	Mutant	No

Summary of the general quantifiable aspects of the solitary genes considered in the present work. MDAS: maximum distance as solitary (measured in Mb), the genomic interval in which the odorant receptor gene is isolated, both upstream and downstream in respect to its transcript coordinates, from neighboring odorant receptor genes; the MDAS is reported also for those orthologs of the mouse solitary genes that are solitary as well, for *Rattus norvegicus* ([Rn]), *Cavia porcellus* ([Cp]) and *Homo sapiens* ([Hs]). HDBS: homeodomain factor binding site. Cell counts (for three-day-old mice, n = 3) are reported as average ± a standard deviation. For *Olfr466* two different candidate promoters are presented, a 5' RACE-derived, polymorphic one (labelled with [R]) and a second, mutant sequence based on Plessy *et al*. (labelled with [P]).

^a^ While reported for convenience, the most isolated orthologous gene of *Olfr19* in *Rattus norvegicus* is not strictly solitary according to our 1 Mb threshold criterion.

^b^ For *Cavia porcellus*, the average genomic scaffold length in the cavPor3 genome assembly is 8.9 Mb.

Based on already published [44] and current expression data, *Olfr466* seems to have the most desirable expression features: its cell counts in newborn mice are acceptably high, and it is well expressed in adult mice (a feature shared with *Olfr19*, *Olfr49* and *Olfr370*); its expression level does not decrease with aging (an advantage also presented by *Olfr266*, *Olfr267* and *Olfr371*). *Olfr19* and *Olfr371* both have high cell counts in three-day-old mice. The expression level of *Olfr19* fades significantly only in ten-week-old mice, so that the gene might still also be considered as a model; on the other hand, the mRNA levels of *Olfr371* are already drastically low at three weeks of age.

Although all solitary genes are by definition dethatched from neighboring OR genes, some are more isolated then others. As mentioned, the most isolated OR gene of the mouse genome is *Olfr466*, followed by *Olfr266* and *Olfr1402*, neighboring each other at a distance of 9.4 Mb.

Regarding the conservation of the solitary status among mammalian species, interesting characteristics are displayed by both *Olfr266* and *Olfr267*, which have a single solitary ortholog in all studied *taxa*. In addition, the pair *Olfr370*/*Olfr371* is noteworthy for having a conserved chromosomal organization in rat, with the orthologous pair of solitary genes *Olr1667*/*Olr1666*.

The data provided by 5' RACE analyses favor above all *Olfr19*, *Olfr371* and *Olfr1402*, having at the same time a simple splicing structure and a well-defined TSS, supported by both our work and literature. *Olfr49*, *Olfr266*, *Olfr267* and *Olfr370* present minor obstacles, related to the possible existence of alternative promoters, less defined TSSs or more complex splicing. The position of the promoter for *Olfr466* varies between our 5' RACE (sharply identifying, in agreement with Ibarra-Soria *et al*., a single TSS at about 2 Kb from the start codon) and Plessy *et al*., placing the TSS for the gene about 13 Kb more upstream. The gene might possess two distant promoters. At present, such discordance makes it less attractive as a model.

Our stringent search for TFBSs for HD factors on putative promoter sequences yielded a single occurrence, on *Olfr266*. Moreover, on the same sequence we found stretches conserved among Mammalia Eutheria. Concerning sequence variation, six out of the nine investigated putative promoters for the solitary genes display the desirable feature of being mildly polymorphic without having a single mutation on the C57BL/6J allele that is unrepresented in wild populations (*Olfr19*, *Olfr49*, *Olfr266*, *Olfr267*, *Olfr371* and *Olfr466* 5' RACE-derived). For four of them (the exception is *Olfr466*) the wild-derived consensus sequence matches the inbred variant without exceptions. The two *M*. *m*. *domesticus* SNPs found on the candidate promoter of *Olfr266* are found outside both the predicted TFBS and the conserved sequences. Finally, the putative promoter of *Olfr370*, with both mutations and a SNP with no match with the wild-derived consensus sequence, may represent an uncommon variant potentially unsuitable to model the physiological functioning of a typical OR gene. To a lesser extent, similar flaws potentially affect the putative promoters for *Olfr466* (the one we derived from Plessy *et al*.) and *Olfr1402*.

Taken everything into account, despite its low expression levels, we currently consider *Olfr266* as the most convenient solitary gene for the investigation of the OR gene choice.

## Supporting Information

S1 Fig Expression of *Olfr1402* in the main olfactory epithelium of adult mice. *In situ* hybridization of the main olfactory epithelium in a ten-week-old mouse, showing (n = 2) punctate expression on olfactory sensory neurons (green) in a coronal emisection of the main olfactory epithelium (left panel, with dorsal side on top); a detail (boxed in white) is magnified on right panels, with or without the addition of DAPI nuclear counterstaining (magenta). Scale bars: 200 μm (whole emisection), 20 μm (detail).
(TIF)

S2 Fig Negative correlation between average cell counts and coefficients of variation. Scatter plot with regression line (magenta) showing negative correlation (r = -0.8145, p = 0.013926) between cell counts for olfactory sensory neurons expressing each solitary gene (Average) and their coefficients of variation (CV) in three-day-old mice.
(TIF)

S1 File Sequences of 5' RACE clones in FASTA format. Each FASTA entry contains the whole polished sequence of a single clone (or at least its 5'-end) derived via 5' RACE. Each numerical header is unique among clones obtained for a single gene from the same kind of biological sample (WOM, meaning whole olfactory mucosa, or OSNs, meaning sorted olfactory sensory neurons). The description field reports the kind of biological sample followed by the gene name. Sequences are invariably presented on genomic plus strand.
(PDF)

S2 File Putative promoters variants in FASTA format. FASTA entries are provided for candidate promoters of all solitary genes plus *Olfr6* (*M50*); in addition to the standard C57BL/6J version, allelic variants are also provided for wild populations of *Mus musculus domesticus* (France, Germany and Iran), for their consensus sequence (described as Wild_consensus) and for 36 additional laboratory strains. Sequences are invariably presented on genomic plus strand.
(PDF)

S1 Table Orthologous gene groups for the mouse solitary genes. The table represents a more detailed version of Table 3. It reports, for each orthologous gene group (OGG) containing a solitary gene in *Mus musculus*, all orthologs found in the studied species. Gene names follow the nomenclature proposed by Niimura *et al*. For solitary or nearly solitary genes (highlighted in bold), we reported the maximum distance as solitary (MDAS), expressed in Mb and defined as the genomic interval in which the odorant receptor gene is isolated, both upstream and downstream in respect to its transcript coordinates, from neighboring odorant receptor genes; when present, we also reported official gene names, or at least Ensembl gene identifiers. Those genes that were recognized as orthologs of a *M*. *musculus* solitary genes also by Inparanoid have underlined gene names. In magenta, we highlighted gene names for those few genes of which the solitary status could not be correctly determined based solely on BioMart annotation: *Olr390* has a non-annotated neighboring odorant receptor gene, and *Olfr266* has a non-annotated solitary ortholog in *Cavia porcellus*.
(XLSX)

## Abbreviations

CV: coefficient of variation
HD: homeodomain
HDBS: homeodomain factor binding site
ISH: *in situ* hybridization
MDAS: maximum distance as solitary
MOE: main olfactory epithelium
O/E: olfactory/early cell B
OGG: orthologous gene group
OR: odorant receptor
OSN: olfactory sensory neuron
PBS: phosphate buffered saline
PSWM: position-specific weight matrix
TFBS: transcription factor binding site
TSS: transcription start site
WOM: whole olfactory mucosa
