# Supplementary figures and images for: The Mouse Solitary Odorant Receptor Gene Promoters as Models for the Study of Odorant Receptor Gene Choice
Source: PLoS One. 2016 Jan 21;11(1):e0144698. doi: 10.1371/journal.pone.0144698 (PMC4721658; doi:10.1371/journal.pone.0144698)

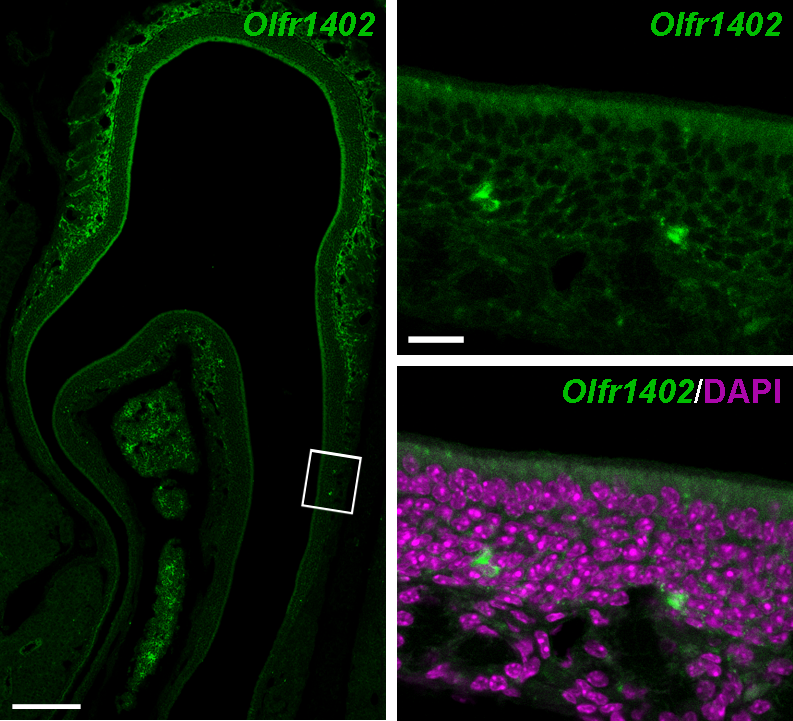

Supplement: S1 Fig — In situ hybridization of the main olfactory epithelium in a ten-week-old mouse, showing (n = 2) punctate expression on olfactory sensory neurons (green) in a coronal emisection of the main olfactory epithelium (left panel, with dorsal side on top); a detail (boxed in white) is magnified on right panels, with or without the addition of DAPI nuclear counterstaining (magenta). Scale bars: 200 μm (whole emisection), 20 μm (detail). (TIF) [file pone.0144698.s001.tif]

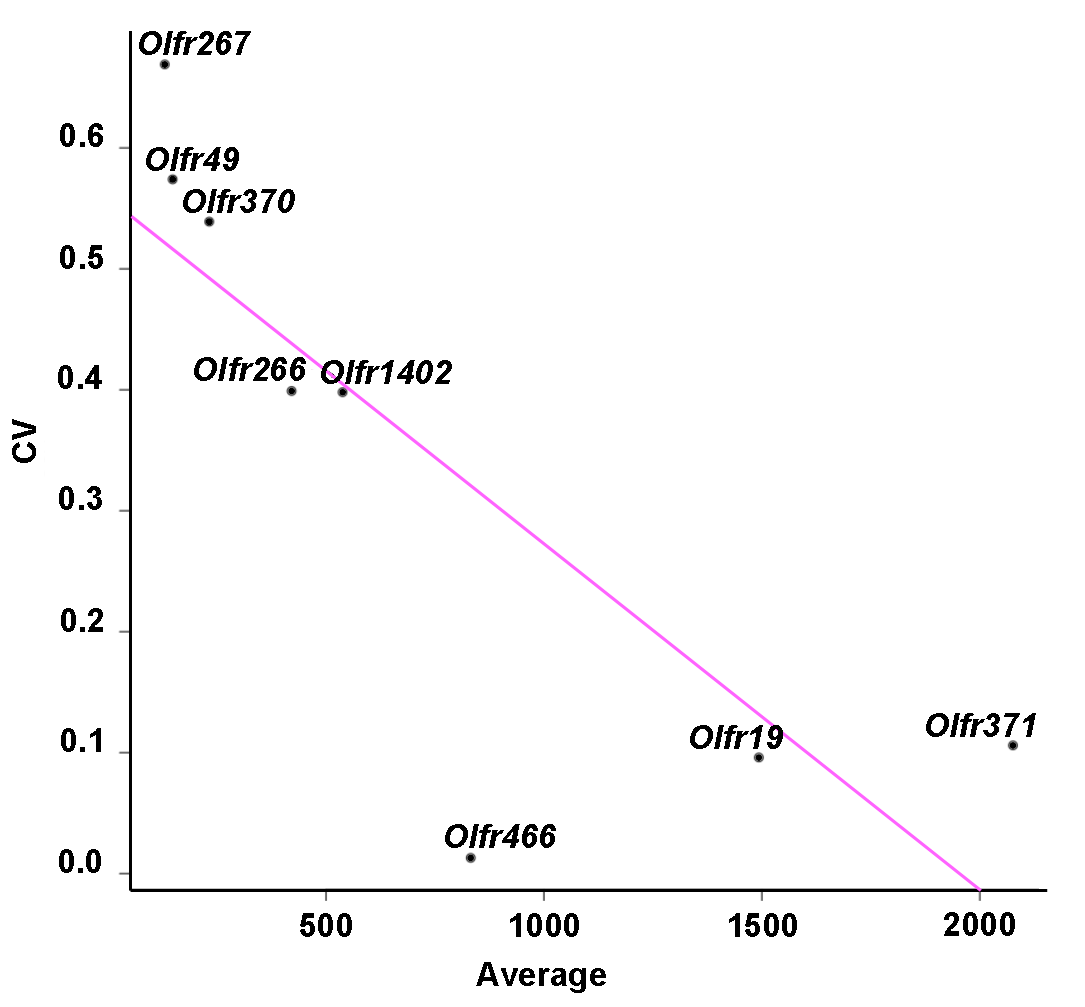

Supplement: S2 Fig — Scatter plot with regression line (magenta) showing negative correlation (r = -0.8145, p = 0.013926) between cell counts for olfactory sensory neurons expressing each solitary gene (Average) and their coefficients of variation (CV) in three-day-old mice. (TIF) [file pone.0144698.s002.tif]
