# Supplementary material for: The Mouse Solitary Odorant Receptor Gene Promoters as Models for the Study of Odorant Receptor Gene Choice
Source: PLoS One. 2016 Jan 21;11(1):e0144698. doi: 10.1371/journal.pone.0144698 (PMC4721658; doi:10.1371/journal.pone.0144698)
Supplement: S1 File — Each FASTA entry contains the whole polished sequence of a single clone (or at least its 5'-end) derived via 5' RACE. Each numerical header is unique among clones obtained for a single gene from the same kind of biological sample (WOM, meaning whole olfactory mucosa, or OSNs, meaning sorted olfactory sensory neurons). The description field reports the kind of biological sample followed by the gene name. Sequences are invariably presented on genomic plus strand. (PDF) [file pone.0144698.s003.pdf]

Sequences of 5' RACE clones in FASTA format

```
>1 WOM_Olfr6
GTGCAGTGACCCAGTGACTCATACAGTAAGAATGATGACCAGATTTTCCAAAAGCACAA
ACACATAAGTTATGAAGAAGATGGAGAAAAGCAGAATTTGCAGCCATGGAGCAGTAGGG
AAGCCCATGAGG
>2 WOM_Olfr6
TAGGCTTGTGCAGTGACCCAGTGACCCATACAGTAAGAATGATGACCAGATTTTCCAAA
AGCACAAACACATAAGTTATGAAGAAGATGGAGAAAAGCAGAATTTGCAGCCATGGAGC
AGTAGGGAAGCCCATG
>3 WOM_Olfr6
TAGGCTTGTGCAGTGACCCAGTGACCCATACAGTAAGAATGATGACCAGATTTTCCAAA
AGCACAAACACATAAGTTATGAAGAAGATGGAGAAAAGCAGAATTTGCAGCCATGGAGC
AGTAGGGAAGCCCATG
>4 WOM_Olfr6
TAGGCTTGTGCAGTGACCCAGTGACCCATACAGTAAGAATGATGACCAGATTTTCCAAA
AGCACAAACACATAAGTTATGAAGAAGATGGAGAAAAGCAGAATTTGCAGCCATGGAGC
AGTAGGGAAGCCCATG
>5 WOM_Olfr6
TAGGCTTGTGCAGTGACCCAGTGACCCATACAGTAAGAATGATGACCAGATTTTCCAAA
AGCACAAACACATAAGTTATGAAGAAGATGGAGAAAAGCAGAATTTGCA
>6 WOM_Olfr6
TAGGCTTGTGCAGTGACCCAGTGACCCATACAGTAAGAATGATGACCAGATTTTCCAAA
AGCACAAACACATAAGTTATGAAGAAGGTGGAGAAAAGCAGAATTTGCAGCCATGGAGC
AGTAGGGAAGCCCATGAGGATGAACTCACTGATGTTGGTGATGTTTTCCATCCACAAAG
TTGGATCACCAAATGACAAAAGATTGGATGCTAATATC
>7 WOM_Olfr6
TAGGCTTGTGCAGTGACCCAGTGACCCATACAGTAAGAATGATGACCAGATTTTCCAAA
AGCACAAACACATAAGTTATGAAGAAGATGGAGAAAAGCAGAATTTGCAGCCATGGAGC
AGTAGGGAAGCCCATG
>8 WOM_Olfr6
TAGGCTTGTGCAGTGACCCAGTGACCCATNCAGTAAGAATGATGACCAGATTTTCCAAA
AGCACAAACACATAAGTTATGAAGAAGATGGAGAAAAGCAGAATTTGCAGCCATGGAGC
AGTAGGGAAGCCCATGAGGATGAACTCACTGATGTTGGTGATGTTTTCCATCCACAAAG
TTGGATCACCAAATGACAAAAGATTGGATGCTAATATCAGAAAATTTAAGAATAAGATC
CATGAGAGTACCATGGAACATTAGGCTGAAGATTAGTTTTATTATTATAAAAATCTTAC
TCTTGATTCCTCATTTACCATGAGACTGATGTGGGAAACAAGAGAGGCCATAAGATGAG
TCCAGAAAAATAGAGACCTCTTACTTTATCCTGCTTTGTATTTAGCTCTGTAGTTGGAA
GCAGTTGAGAATCACCAAACATTGCAGTTACTTAGCAAAGGGACTAGAAAAAGAAAGAG
CCATCTTGTTGAACATTCACCAAATTTTTATATCTTGCATTTGTGATCTTCATTCTTCA
TGGCAGCTTTTTGTTACTCCAAGATCATGTAATCTGATAAGTCAGAAAAAGAAAAATAA
ACAACTGATGTAGCTGACCCAGTAGATGCTCCAAGTAAGAAGTAGGTTTTCAGACTGAT
GGGCAGGTGTCTTTGAGAACAAAAACTTTGCCCCATATGAATCTTTGTCTTTGCATTAT
TGGGCTTGCCTGATACTCCTAGAGGCCAGGATAGAAGAGGTTTTCCTATTGTTGTGCTT
TTTCTCCATCACTGTTCATTGCCTTTCTCAGTCTTCTCTCACTCTTTCTCTTACTTTTG
CTCCTGACCTATTTCTCTTCCC
>9 WOM_Olfr6
```

TAGGCTTGTGCAGTGACCCAGTGACCCATACAGTAAGAATGATGACCAGATTTTCCAAA
AGCACAAACACATAAGTTATGAAGAAGATGGAGAAAAGCAGAATTTGCAGCCATGGAGC
AGTAGGGAAGCCCATGAGGATGAACTCACTGATGTTGGTGATGTTTTCCATCCACAAAG
TTGGATCACCAAATGACAAAAGATTGGATGCTA
>10 WOM_Olfr6
TAGGCTTGTGCAGTGACCCAGTGACCCATACAGTAAGAATGATGACCAGATTTTCCAAA
AGCACAAACACATAAGTTATGAAGAAGATGGAGAAAAGCAGAATTTGCAGCCATGGAGC
AGTAGGGAAGCCCATG
>11 WOM_Olfr6
TAGGCTTGTGCAGTGACCCAGTGACCCATACAGTAAGAATGATGACCAGATTTTCCAAA
AGCACAAACACATAA
>12 WOM_Olfr6
TAGGCTTGTGCAGTGACCCAGTGACCCATACAGTAAGAATGATGACCAGATTTTCCAAA
AGCACAAACACATAAGTTATGAAGAAGATGGAGAAAAGCAGAATTTGCAGCCATGGAGC
AGTAGGGAAGCCCATG
>13 WOM_Olfr6
TAGGCTTGTGCAGTGACCCAGTGACCCATACAGTAAGAATGATGACCAGATTTTCCAAA
AGCACAAACACATAAGTTATGAAGAAGATGGAGAAAAGCAGAATTTGCAGCCATGGAGC
AGTAGGGAAG
>14 WOM_Olfr6
TAGGCTTGTGCAGTGACCCAGTGACCCATACAGTAAGAATGATGACCAGATTTTCCAAA
AGCACAAACACATAAGTTATGAAGA
>15 WOM_Olfr6
TAGGCTTGTGCAGTGACCCAGTGACCCATACAGTAAGAATGATGACCAGATTTTCCAAA
AGCACAAACACATAAGTTATGAAGAAGATGGAGAAAAGCAGAATTTGCAGCCATGGAGC
AGTAGGGAAGCCCATGAG
>16 WOM_Olfr6
TAGGCTTGTGCAGTGACCCAGTGACCCATACAGTAAGAATGATGACCAGATTTTCCAAA
AGCACAAACACATAAGTTATGAAGAAGATGGAGAAAAGCAGAATTTGCAGCCATGGAGC
AGTAGGGAAGCCCATG
>17 WOM_Olfr6
TAGGCTTGTGCAGTGACCCAGTGACCCATACAGTAAGAATGATGACCAGATTTTCCAAA
AGCACAAACACATAAGTTATGAAGAAGATGGAGAAAAGCAGAATTTGCA
>18 WOM_Olfr6
TAGGCTTGTGCAGTGACCCAGTGACCCATACAGTAAGAATGATGACCAGATTTTCCAAA
AGCACAAACACATAAGTTATGAAGAAGATGGAGAAAAGCAGAATTTGCAGCCATGGAGC
AGTAGGGAAGCCCATG
>19 WOM_Olfr6
TAGGCTTGTGCAGTGACCCAGTGACCCATACAGTAAGAATGATGACCAGATTTTCCAAA
AGCACAAACGCATAAGTTATGAAGAAGATGGAGAAAAGCAGAATTTGCAGCCATGGAGC
AGTAGGGAAGCCCATG
>20 WOM_Olfr6
TAGGCTTGTGCAGTGACCCAGTGACCCATACAGTAAGAATGATGACCAGATTTTCCAAA
AGCACAAACACATAAGTTATGAAGAAGATGGAGAAAAGCAGAATTTGTAGCCATGGAGC
AGTAGGGAAGCCCATGAGGATGAACTCACTGATGTTGGTGATGTTTTCCATCCACAAAG
TTGGATCACCAAATGACAAAAGATTGGATGCTAATATC
>21 WOM_Olfr6

TAGGCTTGTGCAGTGACCCAGTGACCCATACAGTAAGAATGATGACCAGATTTTCCAAA
AGCACAAACACATAAGTTATGAAGAAGATGGAGAAAAGCAGAATTTGCAGCCATGGAGC
AGTAGGGAAGCCCATGAGGATGAACTCACTGATGTTGGTGATGTTTTCCATCCACAAAG
TTGGATCACCAAATGACAAAAGATTGGATGCTAATATCAGAAAATTTAAGAATAAGATC
CATGAGAGTACCATGGAACATTAGGCTGAAGATTAGTTTTATTATTATAAAAATCTTAC
TCTTGATTCCTCATTTACCATGAGACTGATGTGGGAAACAAGAGAGGCCATAAGATGAG
TCCAGAAAAATAGAGACCTCTTACTTTATCCTGCTTTGTATTTAGCTCTGTAGTTGGAA
GCAGTTGAGAATCACCAAACATTGCAGTTACTTAGCAAAGGGACTAGAAAAAGAAAGAG
CCATCTTGTTGAACATTCACCAAATTTTTATATCTTGCATTTGTGATCTTCATTCTTCA
TGGCAGCTTTTTGTTACTCCAAGATCATGTAATCTGATAAGTCAGAAAAAGAAAAATAA
ACAACTGATGTAGCTGACCCAGTAGATGCTCCAAGTAAGAAGTAGGTTTTCAGACTGAT
GGGCAGGTGTCTTTGAGAACAAANACTTTGCCCCATATGAATCTTTGTCTTTGCATTAT
TGGGCTTGCCTGATACTCCTAGAGGCCAGGATAGAAGAGGTTTTCCTATTGTTGTGCTT
TTTCTCCATCACTGTTCATTGCCTTTCTCAGTCTTCTCTCACTCTTTCTCTTACTTTTG
CTCCTGACCTATTTCTCTTCCC
>22 WOM_Olfr6
NTAGGCTTGTGCAGTGACCCAGTGACCCATACAGTAAGAATGATGACCAGATTTTCCAA
AAGCACAAACACATAAGTTATGAAGAAGATGGAGAAAAGCAGAATTTGCAGCCATGGAG
CAGTAGGGAAGCCCATGAGGATGAACTCACTGATGTTGGTGATGTTTTCCATCCACAAA
GTTGGATCACCAAATGACAAAAGATTGGATGCTAATATCAGAAAATTTAAGAATAAGAT
CCATGAGAGTACCATGGAACATTAGGCTGAAGATTAGTTTTATTATTATAAAAATCTTA
CTCTTGATTCCTCATTTACCATGAGACTGATGTGGGAAACAAGAGAGGCCATAAGATGA
GTCCAGAAAAATAGAGACCTCTTACTTTATCCTGCTTTGTATTTAGCTCTGTAGTTGGA
AGCAGTTGAGAATCACCAAACATTGCAGTTACTTAGCAAAGGGACTAGAAAAAGAAAGA
GCCATCTTGTTGAACATTCACCAAATTTTTATATCTTGCATTTGTGATCTTCATTCTTC
ATGGCAGCTTTTTGTTACTCCAAGATCATGTAATCTGATAAGTCAGAAAAAGAAAAATA
AACAACTGATGTAGCTGACCCAGTAGATGCTCCAAGTAAGAAGTAGGTTTTCAGACTGA
TGGGCAGGTGTCTTTGAGAACAAAAACTTTGCCCCATATGAATCTTTGTCTTTGCATTA
TTGGGCTTGCCTGATACTCCTAGAGGCCAGGATAGAAGAGGT
>23 WOM_Olfr6
TAGGCTTGTGCAGTGACCCAGTGACCCATACAGTAAGAATGATGACCAGATTTTCCAAA
AGCACAAACACATAAGTTATGAAGA
>24 WOM_Olfr6
AGGCTTGTGCAGTGACCCAGTGACCCATACAGTAAGAATGATGACCAGATTTTCCAAAA
GCACAAACACATAAGTTATGAAGAAGATGGAGAAAAGCAGAATTTGCAGCCATGGAGCA
GTAGGGAAGCCCATG
>25 WOM_Olfr6
TAGGCTTGTGCAGTGACCCAGTGACCCATACAGTAAGAATGATGACCAGATTTTCCAAA
AGCACAAACACATAAGTTATGAAGAAGATGGAGAAAAGCAGAATTTGCAGCCATGGAGC
AGTAGGGAAGCCCATG
>26 WOM_Olfr6
TAGGCTTGTGCAGTGACCCAGTGACCCATACAGTAAGAATGATGACCAGATTTTCCAAA
AGCACAAACACATAAGTTATGAAGAAGATGGAGAAAAGCAGAATTTGCAGCCATGGAGC
AGTAGGGAAGCCCATG
>27 WOM_Olfr6
TAGGCTTGTGCAGTGACCCAGTGACCCATACAGTAAGAATGATGACCAGATTTTCCAAA
AGCACAAACACATAAGTTATGAAGAAGATGGAGAAAAGCAGAATTTGCAGCCATGGAGC

AGTAGGGAAGCCCATGAGGATGAACTCACTGATGTTGGTGATGTTTTCCATCCACAAAG
TTGGATCACCAAATGACAAAAGATTGGATGCTAATATCAGAAAATTTAAGAATAAGATC
CATGAGAGTACCATGGAACATTAGGCTGAAGATTAGTTTTATTATTATAAAAATCTTAC
TCTTGATTCCTCATTTACCATGAGACTGATGTGGGAAACAAGAGAGGCCATAAGATGAG
TCCAGAAAAATAGAGACCTGTAACCACAGCACTCAGCAAATAAGGCAGAGACC
>28 WOM_Olfr6
TAGGCTTGTGCAGTGACCCAGTGACCCATACAGTAAGAATGATGACCAGATTTTCCAAA
AGCACAAACACATAAGTTATGAAGAAGATGGAGAAAAGCAGAATTTGCAGCCATGGAGC
AGTAGGGAAGCCCATGAGGATGAACTCACTGATGTTGGTGATGTTTTCCATCCACAAAG
TTGGATCACCAAATGACAAAAGATTGGATGCTAATATCAGAAAATTTAAGAATAAGATC
CATGAGAGTACCATGGAACATTAGGCTGAAGATTAGTTTTATTATTATAA
>29 WOM_Olfr6
TAGGCTTGTGCAGTGACCCAGTGACCCATACAGTAAGAATGATGACCAGATTTTCCAAA
AGCACAAACACATAAGTTATGAAGAAGATGGAGAAAAGCAGAATTTGCAGCCATGGAGC
AGTAGGGAAGCCCATG
>30 WOM_Olfr6
TAGGCTTGTGCAGTGACCCAGTGACCCATACAGTAAGAATGATGACCAGATTTTCCAAA
AGCACAAACACATAAGTTATGAAGAAGATGGAGAAAAGCAGAATTTGCAGCCATGGAGC
AGTAGGGAAGCCCATG
>31 WOM_Olfr6
TAGGCTTGTGCAGTGACCCAGTGACCCATACAGTAAGAATGATGACCAGATTTTCCAAA
AGCACAAACACATAAGTTATGGAGAAGATGGAGAAAAGCAGAATTTGCAGCCATGGAGC
AGTAGGGAAGCCCATG
>32 WOM_Olfr6
TTGTGCAGTGACCCAGTGACCCATACAGTAAGAATGATGACC
>33 WOM_Olfr6
TAGGCTTGTGCAGTGACCCAGTGACCCATACAGTAAGAATGATGACCAGATTTTCCAAA
AGCACAAACACATAAGTTATGAAGAAGATGGAGAAAAGCAGAATTTGCAGCCATGGAGC
AGTAGGGAAGCCCATGAGGATG
>34 WOM_Olfr6
TAGGCTTGTGCAGTGACCCAGTGACCCATACAGTAAGAATGATGACCAGATTTTCCAAA
AGCACAAACACATAAGTTATGAAGAAGATGGAGAAAAGCAGAATTTGCAGCCATGGAGC
AGTAGGGAAGCCCATGAGGATGAACTCACTGATGTTGGTGATGTTTTCCATCCACAAAG
TTGGATCACCAAATGACAAAAGATTGGATGCTAATATCAGAAAATTTAAGAATAAGATC
CATGAGAGTACCATGGAACATTAGGCTGAAGATTAGTTTTATTATTATAA
>35 WOM_Olfr6
TAGGCTTGTGCAGTGACCCAGTGACCCATACAGTAAGAATGATGACCAGATTTTCCAAA
AGCACAAACACATAAGTTATGAAGAAGATGGAGAAAAGCAGAATTTGCAGCCATGGAGC
AGTAGGGAAGCCCATG
>36 WOM_Olfr6
TAGGCTTGTGCAGTGACCCAGTGACCCATACAGTAAGAATGATGACCAGATTTTCCAAA
AGCACAAACACATAAGTTATGAAGAAGATGGAGAAAAGCAGAATTTGCAGCCATGGAGC
AGTAGGGAAGCCCATG

>1 OSNs_Olfr6
GGCCGCGGGAATTCGATTTAGGCTTGTGCAGTGACCCAGTGACCCATACAGTAAGAATG
ATGACCAGATTTTCCAAAAGCACAAACACATAAGTTATGAAGAAGATGGAGAAAAGCAG

AATTTGCAGCCATGGAGCAGTAGGGAAGCCCATGAGGATGAACTCACTGATGTTGGTGA
TGTTTTCCATCCACAAAGTTCGATCACCAAATGACAAAAGATTGGATGCTAATATCAGA
AAATTTAAGAATAAGATCCATGAGAGTACCATGGAACATTAGGCTGAAGATTAGTTTTA
TTATTATAAAAATCTTACTCTTGATTCCTCATTTACCATGAGACTGATGTGGGAAACAA
GAGAGGCCATAAGATGAGTCCAGAAAAATAGAGACCTCTTACTTTATCCTGCTTTGTAT
TTAGCTCTGTAGTTGGAAGCAGTTGAGAATCACCAAACATTGCAGTTACTTAGCAAAGG
GACTAGAAAAAGAAAGAGCCATCTTGTTGAACATTCACCAAATTTTTATATCTTGCATT
TGTGATCTTCATTCTTCATGGCAGCTTTTTGTTACTCCAAGATCATGTAATCTGATAAG
TCAGAAAAAGAAAAATAAACAACTGATGTAGCTGACCCAGTAGATGCTCCAAGTAAGAA
GTAGGTTTTCAGACTGATGGGCAGGTGTCTTTGAGAACAAAAACTTTGCCCCATATGAA
TCTTTGTCTTTGCATTATTGGGCTTGCCTGATACTCCTAGAGGCCAGGATAGAAGAGGT
TTTCCTATTGTTGTGCTTTTTCTCCATCACTGTTCATTGCCTTTCTCAGTCTTCTCTCA
CTCTTTCTCTTACTCTTGCTCCTGACCTATTTCTCTTCCC
>2 OSNs_Olfr6
GGCCGCGGGAATTCGATTTAGGCTTGTGCAGTGACCCAGTGACCCATACAGTAAGAATG
ATGACCAGATTTTCCAAAAGCACAAACACATAAGTTATGAAGAAGATGGAGAAAAGCAG
AATTTGCAGCCATGGAGCAGTAGGGAAGCCCATGAGGATGAACTCACTGATGTTGGTGA
TGTTTTCCATCCACAAAGTTCGATCACCAAATGACAAAAGATTGGATGCTAATATCAGA
AAATTTAAGAATAAGATCCATGAGAGTACCATGGAACATTAGGCTGAAGATTAGTTTTA
TTATTATAAAAATCTTACTCTTGATTCCTCATTTACCATGAGACTGATGTGGGAAACAA
GAGAGGCCATAAGATGAGTCCAAAAAAATAGAGACCTCTTACTTTCTCCTGCTTTGTAT
TTAGCTCTGTAGTTGGAAGCAGTTGAGAACCACCAAACATTGCAGTTACTTAGCAAAGG
GACTAGAAAAAGAAAGAGCCATCTTGTTGAACATTCACCAAATTTTTATATCTTGCATT
TGTGATCTTCATTCTTCATGGCAGCTTTTTGTTACTCCAAGATCATGTAATCTGATAAG
TCAGAAAAAGAAAAATAAACAACTGATGTAGCTGACCCAGTAGATGCTCCAAGTAAGAT
GTAGGTTTTTATTGGGCTTGCCTGATACTCCTAGAGGCCAGGATAGAAGAGGTTTTCCT
ATTGTTGTGCTTTTTCTCCATCACTGTTCATTGCCTTTCTCAGTCTTCTCTCACTCTTT
CTCTTACTCTTGCTCCTGACCTATTTCTCTTCCC
>3 OSNs_Olfr6
GGCCGCGGGAATTCGATTTAGGCTTGTGCAGTGACCCAGTGACCCATACAGTAAGAATG
ATGACCAGATTTTCCAAAAGCACAAACACATAAGTTATGAAGAAGATGGAGAAAAGCAG
AATTTGCAGCCATGGAGCAGTAGGGAAGCCCATGAGGATGAACTCACTGATGTTGGTGA
TGTTTTCCATCCACAAAGTTGGATCACCAAATGACAAAAGATTGGATGCTCTTACTTTA
TCCTGCTTTGTATTTAGCTCTGTAGTTGGAAGCAGTTGAGAATCACCAAACATTGCAGT
TACTTAGCAAAGGGACTAGAAAAAGAAAGAGCCATCTTGTTGAACATTCACCAAATTTT
TATATCTTGCATTTGTGATCTTCATTCTTCATGGCAGCTTTTTGTTACTCCAAGATCAT
GTAATCTGATAAGTCAGAAAAAGAAAAATAAACAACTGATGTAGCTGACCCAGTAGATG
CTCCAAGTAAGAAGTAGGTTTTCAGACTGATGGGCAGGTGTCTTTGAGAACAAAAACTT
TGCCCCATATGAATCTTTGTCTTTGCATTATTGGGCTTGCCTGATACTCCTAGAGGCCA
GGATAGAAGAGGTTTTCCTATTGTTGTGCTTTTTCTCCATCACTGTTCATTGCCTTTCT
CAGTCTTCTCTCACTCC
>4 OSNs_Olfr6
GGCCGCGGGAATTCGATTTAGGCTTGTGCAGTGACCCAGTGACCCATACAGTAAGAATG
ATGACCAGATTTTCCAAAAGCACAAACACATAAGTTATGAAGAAGATGGAGAAAAGCAG
AATTTGCAGCCATGGAGCAGTAGGGAAG
>5 OSNs_Olfr6

TAGGCTTGTGCAGTGACCCAGTGACCCATACAGTAAGAATGATGACCAGATTTTCCAAA
AGCACAAACACATAAGTTATGAAGAAGATGGAGAAAAGCAGAATTTGCAGCCATGGAGC
AGTAGGGAAGCCCATGAGGATGAACTCACTGATGTTGGTGATGTTTTCCATCCACAAAG
TTGGATCACCAAATGACAAAAGATTGGATGCTAATATCAGAAAATTTAAGAATAAGATC
CATAAGAGTACCATGGAACATTAGGCTGAAGATTAGTTTTATTATTATAAAAATCTTAC
TCTTGATTCCTCATTTACCATGAGACTGATGTGGGAAACAAGAGAGGCCATAAGATGAG
TCCAGAAAAATAGAGACCTCTTACTTTATCCTGCTTTGTATTTAGCTCTGTAGTTGGAA
GCAGTTGAGAATCACCAAACATTGCAGTTACTTAGCAAAGGGACTAGAAAAAGAAAGAG
CCATCTTGTTGAACATTCACCAAATTTTTATATCTTGCATTTGTGATCTTCATTCTTCA
TGGCAGCTTTTTGTTACTCCAAGATCATGTAATCTGATAAGTCAGAAAAAGAAAAATAA
ACAACTGATGTAGCTGACCCAGTAGATGCTCCAAGTAAGAAGTAGGTTTTCAGACTGAT
GGGCAGGTGTCTTTGAGAACAAAAACTTTGCCCCATATGAATCTTTGTCTTTGCATTAT
TGGGCTTGCCTGATACTCCTAGAGGCCAGGATAGAAGAGGTTTTCCTATTGTTGTGCTT
TTTCTCCATCACTGTTCATTGCCTTTCTCC

>6 OSNs_Olfr6

TAGGCTTGTGCAGTGACCCAGTGACCCATACAGTAAGAATGATGACCAGATTTTCCAAA
AGCACAAACACATAAGTTATGAAGAAGATGGAGAAAAGCAGAATTTGCAGCCATGGAGC
AGTAGGGAAGCCCATGAGGATGAACTCACTGATGTTGGTGATGTTTTCCATCCACAAAG
TTNGATCACCAAATGACAAAAGATTGGATGCTAATATCAGAAAATTTAAGAATAAGATC
CATGAGAGTACCATGGAACATTAGGCTGAAGATTAGTTTTATTATTATAAAAATCTTAC
TCTTGATTCCTCATTTACCATGAGACTGATGTGGGAAACAAGAGAGGCCATAAGATGAG
TCCAAAAAAATAGAGACCTAAAACAAAGAACACAGCCAAGAATGTTGAAACAACATTAT
AAGGCCATATTTAAGGTTTAGGTCTTTGGGT

>7 OSNs_Olfr6

TAGGCTTGTGCAGTGACCCAGTGACCCATACAGTAAGAATGATGACCAGATTTTCCAAA
AGCACAAACACATAAGTTATGAAGAAGATGGAGAAAAGCAGAATTTGCAGCCATGGAGC
AGTAGGGAAGCCCATGAGGATGAACTCACTGATGTTGGTGATGTTTTCCATCCACAAAG
TTCGATCACCAAATGACAAAAGATTGGATGCTAATATCAGAAAATTTAAGAATAAGATC
CATGAGAGTACCATGGAAC

>8 OSNs_Olfr6

GGCCGCGGGAATTCGATTTAGGCTTGTGCAGTGACCCAGTGACCCATACAGTAAGAATG
ATGACCAGATTTTCCAAAAGCACAAAC

>9 OSNs_Olfr6

TAGGCTTGTGCAGTGACCCAGTGACCCATACAGTAAGAATGATGACCAGATTTTCCAAA
AGCACAAACACATAAGTTATGAAGAAGATGGAGAAAAGCAGAATTTGCAGCCATGGAGC
AGTAGGGAAGCCCATGAGGATGAACTCACTGATGTTGGTGATGTTTTCCATCCACAAAG
TTCGATCACCAAATGACAAAAGATTGGATGCTAATATCAGAAAATTTAAGAATAAGATC
CATGAGAGTACCATGGAAC

>10 OSNs_Olfr6

GGCCGCGGGAATTCGATTTAGGCTTGTGCAGTGACCCAGTGACCCATACAGTAAGAATG
ATGACCAGATTTTCCAAAAGCACAAACACATAAGTTATGAAGAAGATGGAGAAAAGCAG
AATTTGCAGCCATGGAGCAGTAGGGAAGCCCATGAGGATGAACTCACTGATGTTGGTGA
TGTTTTCCATCCACAAAGTTGGATCACCAAATGACAAAAGATTGGATGCTAATATCAGA
AAATTTAAGAATAAGATCCATGAGAGTACCATGGAACATTAGGCTGAAGATTAGTTTTA
TTATTATAAAAATCTTACTCTTGATTCCTCATTTACCATGAGACTGATGTGGGAAACAA
GAGAGGCCATAAGATGAGTCCAGAAAAATAGAGACCTCTTACTTTATCCTGCTTTGTAT
TTAGCTCTGTAGTTGGAAGCAGTTGAGAATCACCAAACATTGCAGTTACTTAGCAAAGG

GACTAGAAAAAGAAAGAGCCATCTTGTTGAACATTCACCAAATTTTTATATCTTGCATT
TGTGATCTTCATTCTTCATGGCAGCTTTTTGTTACTCCAAGATCATGTAATCTGATAAG
TCAGAAAAAGAAAAATAAACAACTGATGTAGCTGACCCAGTAGATGCTCCAAGTAAGAA
GTAGGTTTTCAGACTGATGGGCAGGTGTCTTTGAGAACAAAAACTTTGCCCCATATGAA
TCTTTGTCTTTGCATTATTGGGCTTGCCTGATACTCCTAGAGGCCAGGATAGAAGAGGT
TTTCCTATTGTTGTGCTTTTTCTCCATCACTGTTCATTGCCTTTCTCAGTCTTCTCTCA
CTCT
>11 OSNs_Olfr6
AGGCTTGTGCAGTGACCCAGTGACCCATACAGTAAGAATGATGACCAGATNTTCCAAAA
GCACAAACACATAAGTTATGAAGAAGATGGAGAAAAGCAGAATTTGCAGCCATGGAGCA
GTAGGGAAGCCCATGAGGATGAACTCACTGATGTTGGTGATGTTTTCCATCCACAAAGT
TCGATCACCAAATGACAAAAGATTGGATGCTAATATCAGAAAATTTAAGAATAAGATCC
ATGAGAGTACCATGGAACATTAGGCTGAAGATTAGTTTTATTATTATAAAAATCTTACT
CTTGATTCCTCATTTACCATGAGACTGATGTGGGAAACAAGAGAGGCCATAAGATGAGT
CCAAAAAAATAGAGACCTCTTACTTTCTCCTGCTTTGTATTTAGCTCTGTAGTTGGAAG
CAGTTGAGAACCACCAAACATTGCAGTTACTTAGCAAAGGGACTAGAAAAAGAAAGAGC
CATCTTGTTGAACATTCACCAAATTTTTATATCTTGCATTTGTGATCTTCATTCTTCAT
GGCAGCTTTTTGTTACTCCAAGATCATGTAATCTGATAAGTCAGAAAAAGAAAAATAAA
CAACTGATGTAGCTGACCCAGTAGATGCTCCAAGTAAGATGTAGGTTTTTATTGGGCTT
GCCTGATACTCCTAGAGGCCAGGATAGAAGAGGTTTTCCTATTGTTGTGCTTTTTCTCC
ATCACTGTTCATTGCCTTTCTCAGTCTTCTCTCACTCTTTCTCTT

>1 WOM_Olfr19
CCCAATATNACNNTTTCNTTTNANNTAATATAATGATATNGATACCACAGCTT
>2 WOM_Olfr19
TGTCTGCAAAGGACAGGTTGGAAAGGAAGAAGTACATGGGTGTGTGAAGATGGGAATCT
GTAATGGTGGCAATGATAATGAGAAGGTTCCCAAGCAGAGTGACCAAGTACATGAACAA
AAATAGTCCAAAAAGGAAAGGTTGCCATAGAGGATCCTCTGAAATTCCCAGGAGAATAA
ATTTTGAAATTTGTGTGTCATTTTTCAACTCCATATAGCTAATAAACCTGGATAGGGCT
GCTGGATGAAAAGACCCTTGTTGGAAATCTAATCTTTCCCCTGGATAGTTGAATATAAG
GACTGAAAATAGTTCGAAGACAGCAGGAAGGGGTCATGAATCAGCTTCCAAGATAGCTG
AACACCCTTAAGAAAACGGAAATGTTCTGTCCACAGCAGTGTTGCATACC
>3 WOM_Olfr19
AAGGACAGGTTGGAAAGGAAGAAGTACATGGGTGTGTGAAGATGGGAATCTGTAATGGT
GGCAATGATAATGAGAAGGTTCCCAAGCAGAGTGACCAAGTACATGAACAAAAATAGTC
CAAAAAGGAAAGGTTGCCATAGAGGATCCTCTGAAATTCCCAGGAGAATAAATTTTGAA
ATTTGTGTGTCATTTTTCAACTCCATATAGCTAATAAACAGGTTGAGCTGGATAGGGCT
GCTGGATGAAAAGACCCTTGTTGGAAATCTAATCTTTCCCCTGGATAGTTGAATATAAG
GACTGAAAATAGTTCGAAGACAGCAGGAAGGGGTCATGAATCAGCTTCCAAGATAGCTG
AACACCCTTAAGAAAACGGAAATGTTCTGTCCACAGCAGTGTTGCATC
>4 WOM_Olfr19
CAGATGTCTGCAAAGGACAGGTTGGAAAGGAAGAAGTACATGGGTGTGTGAAGATGGGA
ATCTGTAATGGTGGCAATGATAATGAGAAGGTTCCCAAGCAGAGTGACCAAGTACATGA
ACAAAAATAGTCCAAAAAGGAAAGGTTGCCATAGAGGATCCTCTGAAATTCCCAGGAGA
ATAAATTTTGAAATTTGTGTGTCATTTTTCAACTCCATATAGCTAATAAACAGGTTGAG
CTGGATAGGGCTGCTGGATGAAAAGACCCTTGTTGGAAATCTAATCTTTCCCCTGGATA
GTTGAATATAAGGACTGAAAATAGTTCGAAGACAGCAGGAAGGGGTCATGAATCAGCTT

CCAAGATAGCTGAACACCCTTAAGAAAACGGAAATGTTCTGTCCACAGCAGTGTTGCAT
C
>5 WOM_Olfr19
TGTCTGCAAAGGACAGGTTGGAAAGGAAGAAGTACATGGGTGTGTGAAGATGGGAATCT
GTAATGGTGGCAATGATAATGAGAAGGTTCCCAAGCAGAGTGACCAAGTACATGAACAA
AAATAGTCCAAAAAGGAAAGGTTGCCATAGAGGATCCTCTGAAATTCCCAGGAGAGTAA
ATTTTGAAATTTGTGTGTCATTTTTCAACTCCATGTAGCTAATAAACAGGTTGAGCTGG
ATAGGGCTGCTGGATGAAAAGACCCTTGTTGGAAATCTAATCTTTCCCCTGGATAGTTG
AATATAAGGACTGAAAATAGTTCGAAGACAGCAGGAAGGGGTCATGAATCAGCTTCCAA
GATAGCTGAACACCCTTAAGAAAACGGAAATGTTCTGTCCACAGCAGTGTTGCATACGT
GGAGACAAAGACAGGGGAGGTACTTAAAGGTCTGTGTC
>6 WOM_Olfr19
TGTCTGCAAAGGACAGGTTGGAAAGGAAGAAGTACATGGGTGTGTGAAGATGGGAATCT
GTAATGGTGGCAATGATAATGAGAAGGTTCCCAAGCAGAGTGACCAAGTACATGAACAA
AAATAGTCCAAAAAGGAAAGGTTGCCATAGAGGATCCTCTGAAATTCCCAGGAGAATAA
ATTTTGAAATTTGTGTGTCATTTTTCAACTCCATATAGCTAATAAACCTGGATAGGGCT
GCTGGATGAAAAGACCCTTGTTGGAAATCTAATCTTTCCCCTGGATAGTTGAATATAAG
GACTGAAAATAGTTCGAAGACAGCAGGAAGGGGTCATGAATCAGCTTCCAAGATAGCTG
AACACCCTTAAGAAAACGGAAATGTTCTGTCCACAGCAGTGTTGCATACC
>7 WOM_Olfr19
TGTCTGCAAAGGACAGGTTGGAAAGGAAGAAGTACATGGGTGTGTGAAGATGGGAATCT
GTAATGGTGGCAATGATAATGAGAAGGTTCCCAAGCAGAGTGACCAAGTACATGAACAA
AAATAGTCCAAAAAGGAAAGGTTGCCATAGAGGATCCTCTGAAATTCCCAGGAGAATAA
ATTTTGAAATTTGTGTGTCATTTTTCAACTCCATATAGCTAATAAACCTGGATAGGGCT
GCTGGATGAAAAGACCCTTGTTGGAAATCTAATCTTTCCCCTGGATAGTTGAATATAAG
GACTGAAAATAGTTCGAAGACAGCAGGAAGGGGTCATGAATCAGCTTCCAAGATAGCTG
AACACCCTTAAGAAAACGGAAATGTTCTGTCCACAGCAGTGTTGCAT
>8 WOM_Olfr19
TGTCTGCAAAGGACAGGTTGGAAAGGAAGAAGTACATGGGTGTGTGAAGATGGGAATCT
GTAATGGTGGCAATGATAATGAGAAGGTTCCCAAGCAGAGTGACCAAGTACATGAACAA
AAATAGTCCAAAAAGGAAAGGTTGCCATAGAGGATCCTCTGAAATTCCCAGGAGAATAA
ATTTTGAAATTTGTGTGTCATTTTTCAACTCCATATAGCTAATAAACCTGGATAGGGCT
GCTGGATGAAAAGACCCTTGTTGGAAATCTAATCTTTCCCCTGGATAGTTGAATATAAG
GACTGAAAATAGTTCGAAGACAGCAGGAAGGGGTCATGAATCAGCTTCCAAGATAGCTG
AACACCCTTAAGAAAACGGAAATGTTCTGTCCACAGCAGTGTTGCATACGTGGAGACAA
AGACAGGGGAGGTACC
>9 WOM_Olfr19
TGTCTGCAAAGGACAGGTTGGAAAGGAAGAAGTACATGGGTGTGTGAAGATGGGAATCT
GTAATGGTGGCAATGATAATGAGAAGGTTCCCAAGCAGAGTGACCAAGTACATGAACAA
AAATAGTCCAAAAAGGAAAGGTTGCCATAGAGGATCCTCTGAAATTCCCAGGAGAATAA
ATTTTGAAATTTGTGTGTCATTTTTCAACTCCATATAGCTAATAAACCTGGATAGGGCT
GCTGGATGAAAAGACCCTTGTTGGAAATCTAATCTTTCCCCTGGATAGTTGAATATAAG
GACTGAAAATAGTTCGAAGACAGCAGGAAGGGGTCATGAATCAGCTTCCAAGATAGCTG
AACACCCTTAAGAAAACGGAAATGTTCTGTCCACAGCAGTGTTGCATACC

>1 OSNs_Olfr19

```
TGTCTGCAAAGGACAGGTTGGAAAGGAAGAAGTACATGGGTGTGTGAAGATGGGAATCT
GTAATGGTGGCAATGATAATGAGAAGGTTCCCAAGCAGAGTGACCAAGTACATGAACAA
AAATAGTCCAAAAAGGAAAGGTTGCCATAGAGGATCCTCTGAAATTCCCAGGAGAATAA
ATTTTGAAATTTGTGTGTCATTTTTCAACTCCATATAGCTAATAAACAGGTTGAGCTGG
ATAGGGCTGCTGGATGAAAAGACCCTTGTTGGAAATCTAATCTTTCCCCTGGATAGTTG
AATATAAGGACTGAAAATAGTTCGAAGACAGCAGGAAGGGGTCATGAATCAGCTTCCAA
GATAGCTGAACACCCTTAAGAAAACGGAAATGTTCTGTCCACAGCAGTGTTGCATACC
>2 OSNs_Olfr19
TGTCTGCAAAGGACAGGTTGGAAAGGAAGAAGTACATGGGTGTGTGAAGATGGGAATCT
GTAATGGTGGCAATGATAATGAGAAGATTCCCAAGCAGAGTGACCAAGTACATGAACAA
AAATAGTCCAAAAAGGAAAGGTTGCCATAGAGGATCCTCTGAAATTCCCAGGAGAATAA
ATTTTGAAATTTGTGTGTCATTTTTCAACTCCATATAGCTAATAAACAGGTTGAGCTGG
ATAGGGCTGCTGGATGAAAAGACCCTTGTTGGAAATCTAATCTTTCCCCTGGATAGTTG
AATATAAGGACTGAAAATAGTTCGAAGACAGCAGGAAGGGGTCATGAATCAGCTTCCAA
GATAGCTGAACACCCTTAAGAAAACGGAAATGTTCTGTCCACAGCAGTGTTGCATAC
>3 OSNs_Olfr19
TGTCTGCAAAGGACAGGTTGGAAAGGAAGAAGTACATGGGTGTGTGAAGATGGGAATCT
GTAATGGTGGCAATGATAATGAGAAGGTTCCCAAGCAGAGTGACCAAGTACATGAACAA
AAATAGTCCAAAAAGGAAAGGTTGCCATAGAGGATCCTCTGAAATTCCCAGGAGAATAA
ATTTTGAAATTTGTGTGTCATTTTTCAACTCCATATAGCTAATAAACAGGTTGAGCTGG
ATAGGGCTGCTGGATGAAAAGACCCTTGTTGGAAATCTAATCTTTCCCCTGGATAGTTG
AATATAAGGACTGAAAATAGTTCGAAGACAGCAGGAAGGGGTCATGAATCAGCTTCCAA
GATAGCTGAACACCC
>4 OSNs_Olfr19
CGGGGAGANCTGGATAGGGCTGCTGGATGAAAAGACCCTTGTTGGAAATCTAATCTTTC
CCCTGGATAGTTGAATATAAGGACTGAAAATAGTTCGAAGACAGCAGGAAGGGGTCATG
AATCAGCTTCCAAGATAGCTGAACACCCTTAAGAAAACGGAAATGTTCTGTCCACAGCA
GTGTTGCATACC
>5 OSNs_Olfr19
TGTCTGCAAAGGACAGGTTGGAAAGGAAGAAGTACATGGGTGTGTGAAGATGGGAATCT
GTAATGGTGGCAATGATAATGAGAAGGTTCCCAAGCAGAGTGACCAAGTACATGAACAA
AAATAGTCCAAAAAGGAAAGGTTGCCATAGAGGATCCTCTGAAATTCCCAGGAGAATAA
ATTTTGAAATTTGTGTGTCATTTTTCAACTCCATATAGCTAATAAACAGGTTGAGCTGG
ATAGGGCTGCTGGATGAAAAGACCCTTGTTGGAAATCTAATCTTTCCCCTGGATAGTTG
AATATAAGGACTGAAAATAGTTCGAAGACAGCAGGAAGGGGTCATGAATCAGCTTCCAA
GATAGCTGAACACCCTTAAGAAAACGGAAATGTTCTGTCCACAGCAGTGTTGCATACC
>6 OSNs_Olfr19
TGTCTGCAAAGGACAGGTTGGAAAGGAAGAAGTACATGGGTGTGTGAAGATGGGAATCT
GTAATGGTGGCAATGATAATGAGAAGGTTCCCAAGCAGAGTGACCAAGTACATGAACAA
AAATAGTCCAAAAAGGAAAGGTTGCCATAGAGGATCCTCTGAAATTCCCAGGAGAATAA
ATTTTGAAATTTGTGTGTCATTTTTCAACTCCATATAGCTAATAAACCTGGATAGGGCT
GCTGGATGAAAAGACCCTTGTTGGAAATCTAATCTTTCCCCTGGATAGTTGAATATAAG
GACTGAAAATAGTTCGAAGACAGCAGGAAGGGGTCATGAATCAGCTTCCAAGATAGCTG
AACACCCTTAAGAAAACGGAAATGTTCTGTCCACAGCAGTGTTGCATACC
>7 OSNs_Olfr19
CANAAAACTAATAAANCTGGATAGGGCTGCTGGATGAAAAGACCCTTGTTGGAAATCTA
ATCTTTCCCCTGGATAGTTGAATATAAGGACTGAAAATAGTTCGAAGACAGCAGGAAGG
```

GGTCATGAATCAGCTTCCAAGATAGCTGAACACCCTTAAGAAAACGGAAATGTTCTGTC
CACAGCAGTGTTGCATACC

>1 WOM_Olfr49
GCATGGCAAAGTTGCGGAGGAAGTAATACATGGGGGTGTAAAGGCGCCTGTCCACAAGG
GTGATGAAGATGATGAGGAAGTTTCCCAGCAGAATGAGGAAGTAGGTCAGGAGAAAGCC
CAGGAATATGAGCACCTGCAGCTCACAGGCATCTGACAGCCCCAGCAAAATAAACTCAG
TAACAGTAGTGCTGTTCGCCATGGCTCCTCCCACCTCTGCTGGGGATGGTCATGGACCC
TGCTGAGCATCAGAGCCTGTGAGCTCTGAGGACAACACAAAGCTGTGTGTATATGTCTG
GGTGAGTGCAGGTGTGTGATGTGAGTGCAGATGCCCTCAGTAACCAGCAGTACTGGATC
CCCTGGAGCTGAAGTTGCCCGACACGGAGGCTGGGAGTTGAAGTCGGTCCTCTGCTGGA
GCAGTATGTGCTCCTAGCTTCTTAACCATCTCTTCATTCCCAGTCTGTGAAGATTTTGA
TGCAGGGCTGAGAGGTAGCTCAGCGGCACACCCCTTGTCTGGTGTGCGTGAGGCTGGTT
TCAAACACCACAGTTCTGTTTTCTCTTTCTCTGTTGCGTTTTCTATCGTTATTTAATCT
ACATATTGGTTTATACAGATTATGCATTTTACCCTTCTTCTTTTAAGATAGTAATAAAA
TAAAATGACATTATTATAAACTTTTTGTAATTATTTGGCTACTATTTTTGAAGACTTCA
GGTACACGAAACTGATACATAGATAAGAAAGCATGCATTTTTATCATGTTTAGCTTTTG
TATAACATCCTCAATAATCAACACAAATAACCACTCAGAGTCATCCATGGATTCAATCT
CAATTGTTCAGATTATGTTTTTACCTTAAGGTCTACCGTATGTTCTGCAGATCACAATC
AGGCAGCTCTTGTCCCATTACACTTCACTAAACCT
>2 WOM_Olfr49
GCATGGCAAAGTTGCGGAGGAAGTAATACATGGGGGTGTAAAGGCGCCTGTCCACAAGG
GTGATGAAGATGATGAGGAAGTTTCCCAGCAGAATGAGGAAGTAGGTCAGGAGAAAGCC
CAGGAATATGAGCACCTGCAGCTCACAGGCATCTGACAGCCCCAGCAAAATAAACTCAG
TAACAGTAGTGCTGTTCGCCATGGCTCCTCCCACCTCTGCTGGGGATGGTCATGGACCC
TGCTGAGCATCAGAGCCTGTGAGCTCTGAGGACAACACAAAGCTGTGTGTATATGTCTG
GGTGAGTGCAGGTGTGTGATGTGAGTGCAGATGCCCTCAGTAACCAGCAGTACTGGATC
CCCTGGAGCTGAAGTTGCCCGACACGGAGGCTGGGAGTTGAAGTCGGTCCTCTGCTGGA
GCAGTATGTGCTCCTAGCTTCTTAACCATCTCTTCATTCCCAGTCTGTGAAGATTTTGA
TGCAGGGCTGAGAGGTAGCTCAGCGGCACACCCCTTGTCTGGTGTGCGTGAGGCTGGTT
TCAAACACCACAGTTCTGTTTTCTCTTTCTCTGTTGCGTTTTCTATCGTTATTTAATCT
ACATATTGGTTTATACAGATTATGCATTTTACCCTTCTTCTTTTAAGATAGTAATAAAA
TAAAATGACATTATTATAAACTTTTTGTAATTATTTGGCTACTATTTTTGAAGACTTCA
GGTACACGAAACTGATACATAGATAAGAAAGCATGCATTTTTATCATGTTTAGCTTTTG
TATAACATCCTCAATAATCAACACAAATAACCACTCAGAGTCATCCATGGATTCAATCT
CAATTGTTCAGATTATGTTTTTACCTTAAGGTCTACCGTATGTTCTGCAGATCACAATC
AGGCAGCTCTTGTCCCATTACACTTCACTAAACCTTCAGTTTTTCCAGCGACCCACCTC
CCTTGCAATACTC
>3 WOM_Olfr49
GCATGGCAAAGTTGCGGAGGAAGTAATACATGGGGGTGTAAAGGCGCCTGTCCACAAGG
GTGATGAAGATGATGAGGAAGTTTCCCAGCAGAATGAGGAAGTAGGTCAGGAGAAAGCC
CAGGAATATGAGCACCTGCAGCTCACAGGCATCTGACAGCCCCAGCAAAATAAACTCAG
TAACAGTAGTGCTGTTCGCCATGGCTCCTCCCACCTCTGCTGGGGATGGTCATGGACCC
TGCTGAGCATCAGAGCCTGTGAGCTCTGAGGACAACACAAAGCTTAAGGTCTACCGTAT
GTTCTGCAGATCACAATCAGGCAGCTCTTGTCCCATTACACTTCACTAAACCTTCAGTT
TTTCCGGCGACCCACCTCCCTTGCAATACTCCCTCCCTCGGTCCTCTCAGAAGTGAGTT

GTCTCTGGGTACTTACTAGAATTGGGATTACCAACTCATTGACTGCTAGGGCTCCTAAC
CCCAAGTGTCAC
>4 WOM_Olfr49
GCATGGCAAAGTTGCGGAGGAAGTAATACATGGGGGTGTAAAGGCGCCTGTCCACAAGG
GTGATGAAGATGATGAGGAAGTTTCCCAGCAGAATGAGGAAGTAGGTCAGGAGAAAGCC
CAGGAATATGAGCACCTGCAGCTCACAGGCATCTGACAGCCCCAGCAAAATAAACTCAG
TAACAGTAGTGCTGTTCGCCATGGCTCCTCCCACCTCTGCTGGGGATGGTCATGGACCC
TGCTGAGCATCAGAGCCTGTGAGCTCTGAGGACAACACAAAGCTGTGTGTATATGTCTG
GGTGAGTGCAGGTGTGTGATGTGAGTGCAGATGCCCTCAGTAACCAGCAGTACTGGATC
CCCTGGAGCTGAAGTTGCCCGACACGGAGGCTGGGAGTTGAAGTCGGTCCTCTGCTGGA
GCAGTATGTGCTCCTAGCTTCTTAACCATCTCTTCATTCCCAGTCTTAAGGTCTACTGT
ATGTTCTGCAGATCACAATCAGGCAGCTCTTGTCCCATTACACTTCACTAAACCTTCAG
TTTTTCCAGCGACCCACCTCCCTTGCAATACTCCCTCCCTCGGTCCTCTCAGAAGTGAG
TTGTCTCTGGGTACTTACTAGAATTGGGATTACCAACTCATTGACTGCTAGGGCTCCTA
ACCCCAAGTGTCACC
>5 WOM_Olfr49
GCATGGCAAAGTTGCGGAGGAAGTAATACATGGGGGTGTAAAGGC
>6 WOM_Olfr49
GCATGGCAAAGTTGCGGAGGAAGTAATACATGGGGGTGTAAAGGC
>7 WOM_Olfr49
ATGAAGATGATGAGGAAGTTTCCCAGCAGAATGAGGAAGTAGGTCAGGAGAAAGCCCAG
GAATATGAGCACCTGCAGNTNNNAGGCANNTGACAGCCCCAGCAAAATAANNNCAGTAA
CAGTAGTGCTGTTCGCCATGACTCCTNCCACCTCTGCTGGGGATGGTCATGGACCCTGC
TGAGCATCAGAGCCTGTGAGCTCTGAGGACAACACAAAGCTGTGTGTATATGTCTGGGT
GAGTGCAGGTGTGTGATGTGAGTGCAGATGCCCTCAGTAACCAGCAGTACTGGATCCCC
TGGAGCTGAAGTTGCCCGACACGGAGGCTGGGAGTTGAAGTCGGTCCTCTGCTGGAGCA
GTATGTGCTCCTAGCTTCTTAACCATCTCTTCATTCCCAGTCTGTGAAGATTTTGATGC
AGGGCTGAGAGGTAGCTCAGCGGCACACCCCTTGTCTGGTGTGCGTGAGGCTGGTTTCA
AACACCACAGTTCTGTTTTCTCTTTCTCTGTTGCGTTTTCTATCGTTATTTAATCTACA
TATTGGTTTATACAGATTATGCATTTTACCCTTCTTCTTTTAAGATAGTAATAAAATAA
AATGACATTATTATAAACTTTTTGTAATTATTTGGCTACTATTTTTGAAGACTTCAGGT
ACACGAAACTGATACATAGATAAGAAAGCATGCATTTTTATCATGTTTAGCTTTTGTAT
AACATCCTCAATAATCAACACAAATAACCACTCAGAGTCATCCATGGATTCAATCTCAA
TTGTTCAGATTATGTTTTTACCTTAAGGTCTACCGTATGTTCTGCAGATCACAATCAGG
CAGCTCTTGTCCCATTACACTTCACTAAACCTTCAGTTTTTCCAGCGACCCACCTCCCT
TGCAATACTCCCTCCCTCGGTCCTCTCAGAAGTGAGTTGTCTCTGGGTACTTACTAGAA
TTGGGATTACCAACTCATTGACTGCTAGGGCTCCTAACCCCAAGTGTCACAGTTGGGAC
CCTTGCCTGAGAAAGTTAAATATACATCTATAGTTCCTGTAGTAGAGAGACAGACATCA
ATGGATGACTTGTGTC
>8 WOM_Olfr49
GCATGGCAAAGTTGCGGAGGAAGTAATACATGGGGGTGTAAAGGCGCCTGTCCACAAGG
GTGATGAAGATGATGAGGAAGTTTCCCAGCAGAATGAGGAAGTAGGTCAGGAGAAAGCC
CAGGAATATGAGCACCTGCAGCTCACAGGCATCTGACAGCCCCAGCAAAATAAACTCAG
TAACAGTAGTGCTGTTCGCCATGGCTC
>9 WOM_Olfr49
GCATGGCAAAGTTGCGGAGGAAGTAATACATGGGGGTGTAAAGGCGCCTGTCCACAAGG
GTGATGAAGATGATGAGGAAGTTTCCCAGCAGAATGAGGAAGTAGGTCAGGAGAAAGCC

CAGGAATATGAGCACCTGCAGCTCACAGGCATCTGACAGCCCCAGCAAAATAAACTCAG
TAACAGTAGTGCTGTTCGCCATGGCTCCTCCCACCTCTGCTGGGGATGGTCATGGACCC
TGCTGAGCATCAGAGCCTGTGAGCTCTGAGGACAACACAAAGCTGTGTGTATATGTCTG
GGTGAGTGCAGGTGTGTGATGTGAGTGCAGATGCCCTCAGTAACCAGCAGTACTGGATC
CCCTGGAGCTGAAGTTGCCCGACACGGAGGCTGGGAGTTGAAGTCGGTCCTCTGCTGGA
GCAGTATGTGCTCCTAGCTTCTTAACCATCTCTTCATTCCCAGTATGCTGCTCGTGATC
TTCCAGTCCCACTGTGCCCACAGAAGACTCTGGGGTACAGCAATCAGGGTAGACCCTTT
GTTTCTGTGTACTGGGAAGAGGCCTGAGGTATTAGACATTCTCTTTGGTCTCTATGGGA
ATTCC
>10 WOM_Olfr49
GCATGGCAAAGTTGCGGAGGAAGTAATACATGGGGGTGTAAAGGCGCCTGTCCACAAGG
GTGATGAAGATGATGAGGAAGTTTCCCAGCAGAATGAGGAAGTAGGTCAGGAGAAAGCC
CAGGAATATGAGCACCTGCAGCTCACAGGCATCTGACAGCCCCAGCAAAATAAACTCAG
TAACAGTAGTGCTGTTCGCCATGGCTCCTCCCACCTCTGCTGGGGATGGTCATGGACCC
TGCTGAGCATCAGAGCCTGTGAGCTCTGAGGACAACACAAAGCTGTGTGTATATGTCTG
GGTGAGTGCAGGTGTGTGATGTGAGTGCAGATGCCCTCAGTAACCAGCAGTACTGGATC
CCCTGGAGCTGAAGTTGCCCGACACGGAGGCTGGGAGTTGAAGTCGGTCCTCTGCTGGA
GCAGTATGTGCTCCTAGCTTCTTAACCATCTCTTCATTCCCAGTATGCTGCTCGTGATC
TTCCAGTCCCACTGTGCCCACAGAAGACTCTGGGGTACAGCAATCAGGGTAGACCCTTT
GTTTCTC
>11 WOM_Olfr49
GCATGGCAAAGTTGCGGAGGAAGTAATACATGGGGGTGTAAAGGCGCCTGTCCACAAGG
GTGATGAAGATGATGAGGAAGTTTCCCAGCAGAATGAGGAAGTAGGTCAGGAGAAAGCC
CAGGAATATGAGCACCTGCAGCTCACAGGCATCTGACAGCCCCAGCAAAATAAACTCAG
TAACAGTAGTGCTGTTCGCCATGGCTCCTCCCACCTCTGCTGGGGATGGTCATGGACCC
TGCTGAGCATCAGAGCCTGTGAGCTCTGAGGACAACACAAAGCTGTGTGTATATGTCTG
GGTGAGTGCAGGTGTGTGATGTGAGTGCAGATGCCCTCAGTAACCAGCAGTACTGGATC
CCCTGGAGCTGAAGTTGCCCGACACGGAGGCTGGGAGTTGAAGTCGGTCCTCTGCTGGA
GCAGTATGTGCTCCTAGCTTCTTAACCATCTCTTCATTCCCAGTTAGAATTGGGATTAC
CAACTCATTGGCTGCTAGGGCTCCTAACCCCAAGTGTCACC
>12 WOM_Olfr49
GCATGGCAAAGTTGCGGAGGAAGTAATACATGGGGGTGTAAAGGCGCCTGTCCACAAGG
GTGATGAAGATGATGAGGAAGTTTCCCAGCAGAATGAGGAAGTAGGTCAGGAGAAAGCC
CAGGAATATGAGCACCTGCAGCTCACAGGCATCTGACAGCCCCAGCAAAATAAACTCAG
TAACAGTAGTGCTGTTCGCCATGGCTCCCCATGTC
>13 WOM_Olfr49
GCATGGCAAAGTTGCGGAGGAAGTAATACATGGGGGTGTAAAGGCGCCTGTCCACAAGG
GTGATGAAGATGATGAGGAAGTTTCCCAGCAGAATGAGGAAGTAGGTCAGGAGAAAGCC
CAGGAATATGAGCACCTGCAGCTCACAGGCATCTGACAGCCCCAGCAAAATAAACTCAG
TAACAGTAGTGCTGTTCGCCATGGCTC
>14 WOM_Olfr49
GCATGGCAAAGTTGCGGAGGAAGTAATACATGGGGGTGTAAAGGCGCCTGTCCACAAGG
GTGATGAAGATGATGAGGAAGTTTCCCAGCAGAATGAGGAAGTAGGTCAGGAGAAGGCC
CAGGAATATGAGCACCTGCAGCTCACAGGCATCTGACAGCCCCAGCAAAATAAACTCAG
TAACAGTAGTGCTGTTCGCCATGGCTCCTCCCACCTCTGCTGGGGATGGTCATGGACCC
TGCTGAGCATCAGAGCCTGTGAGCTCTGAGGACAACACAAAGCTTAAGGTCTACCGTAT
GTTCTGCAGATCACAATCAGGCAGCTCTTGTCCCATTACACTTCACTAAACCTTCAGTT

TTTCCAGCGACCCACCTCCCTTGCAATACTCCCTCCCTCGGCCCTCTCAGAAGTGAGTT
GTCTCTGGGTACTTACTAGAATTGGGATTACCAACTCATTGACTGCTAGGGCTCCTAAC
CCCAAGTGTCACC
>15 WOM_Olfr49
GCATGGCAAAGTTGCGGAGGAAGTAATACATGGGGGTGTAAAGGCGCCTGTCCACAAGG
GTGATGAAGATGATGAGGAAGTTTCCCAGCAGAATGAGGAAGTAGGTCAGGAGAAAGCC
CAGGAATATGAGCACCTGCAGCTCACAGGCATCTGACAGCCCCAGCAAAATAAACTCAG
TAACAGTAGTGCTGTTCGCCATGGCTCCTCCCACCTCTGCTGGGGATGGTCATGGACCC
TGCTGAGCATCAGAGCCTGTGAGCTCTGAGGACAACACAAAGCTGTGTGTATATGTCTG
GGTGAGTGCAGGTGTGTGATGTGAGTGCAGATGCCCTCAGTAACCAGCAGTACTGGATC
CCCTGGAGCTGAAGTTGCCCGACACGGAGGCTGGGAGTTGAAGTCGGTCCTCTGCTGGA
GCAGTATGTGCTCCTAGCTTCTTAACCATCTCTTCATTCCCAGTATGCTGCTCGTGATC
TTCCAGTCCCACTGTGCCCACAGAAGACTCTGGGGTACAGCAATCAGGGTAGACCCTTT
GTTTCTGTGCACTGGGAAGAGGCCTGAGGTATTAGACATTCTCTTTGGTCTCTATGGGA
ATTCGAGATGTGCAAAGATGCATGGCTCC
>16 WOM_Olfr49
GCATGGCAAAGTTGCGGAGGAAGTAATACATGGGGGTGTAAAGGCGCCTGTCCACAAGG
GTGATGAAGATGATGAGGAAGTTTCCCAGCAGAATGAGGAAGTAGGTCAGGAGAAAGCC
CAGGAATATGAGCACCTGCAGCTCACAGGCATCTGACAGCCCCAGCAAAATAAACTCAG
TAACAGTAGTGCTGTTCGCCATGGCTCCTCCCACCTCTGCTGGGGATGGTCATGGACCC
TGCTGAGCATCAGAGCCTGTGAGCTCTGAGGACAACACAAAGCTGTGTGTATATGTCTG
GGTGAGTGCAGGTGTGTGATGTGAGTGCAGATGCCCTCAGTAACCAGCAGTACTGGATC
CCCTGGAGCTGAAGTTGCCCGACACGGAGGCTGGGAGTTGAAGTCGGTCCTCTGCTGGA
GCAGTATGTGCTCCTAGCTTCTTAACCATCTCTTCATTCCCAGTATGCTGCTCGTGATC
TTCCAGTCCCACTGTGCCCACAGAAGACTCTGGGGTACAGCAATCAGGGTAGACCC
>17 WOM_Olfr49
GCATGGCAAAGTTGCGGAGGAAGTAATACATGGGGGTGTAAAGGCGCCTGTCCACAAGG
GTGATGAAGATGATGAGGAAGTTTCCCAGCAGAATGAGGAAGTAGGTCAGGAGAAAGCC
CAGGAATATGAGCACCTGCAGCTCACAGGCATCTGACAGCCCCAGCAAAATAAACTCAG
TAACAGTAGTGCTGTTCGCCATGGCTCCTCCCACCTCTGCTGGGGATGGTCATGGACCC
TGCTGAGCATCAGAGCCTGTGAGCTCTGAGGACAACACAAAGCTGTGTGTATATGTCTG
GGTGAGTGCAGGTGTGTGATGTGAGTGCAGATGCCCTCAGTAACCAGCAGTACTGGATC
CCCTGGAGCTGAAGTTGCCCGACACGGAGGCTGGGAGTTGAAGTCGGTCCTCTGCTGGA
GCAGTATGTGCTCCTAGCTTCTTAACCATCTCTTCATTCCCAGTTAGAATTGGGATTAC
CAACTCATTGACTGCTAGGGCTCCTAACCCCAAGCGTCACC

>1 OSNs_Olfr49
GCATGGCAAAGTTGCGGAGGAAGTAATACATGGGGGTGTAAAGGCGCCTGTCCACAAGG
GTGATGAAGATGATGAGGAAGTTTCCCAGCAGAATGAGGAAGTAGGTCAGGAGAAAGCC
CAGGAATATGAGCACCTGCAGCTCACAGGCATCTGACAGCCCCAGCAAAATAAACTCAG
TAACAGTAGTGCTGTTCGCCATGGCTCCTCCCACCTCTGCTGGGGATGGTCATGGACCC
TGCTGAGCATCAGAGCCTGTGAGCTCTGAGGACAACACAAAGCTGTGTGTATATGTCTG
GGTGAGTGCAGGTGTGTGATGTGAGTGCAGATGCCCTCAGTAAC
>2 OSNs_Olfr49
CCGCGAATTCACTAGTGATTGCATGGCAAAGTTGCGGAGGAAGTAATACATGGGGGTGT
AAAGGCGCCTGTCCACAAGGGTGATGAAGATGATGAGGAAGTTTCCCAGCAGAATGAGG
AAGTAGGTCAGGAGAAAGCCCAGGAATATGAACACCTGCAGCTCACAGGCATCTGACAG

CCCCAGCAAAATAAACTCAGTAACAGTAGTGCTGTTCGCCATGGCTCCTCCCACCTCTG
CTGGGGATGGTCATGGACCCTGCTGAGCATCAGAGCCTGTGAGCTCTGAGGACAACACA
AAGCTGTGTGTATATGTCTGGGTGAGTGCAGGTGTGTGATGTGAGTGCAGATGCCCTCA
GTAACCAGCAGTACTGGATCCCCTGGAGCTGAAGTTGCCCGACACGGAGGCTGGGAGTT
GAAGTCGGTCCTCTGCTGGAGCAGTATGTGCTCCTAGCTTCTTAACCATCTCTTCATTC
CCAGTTAGAATTGGGATTACCAACTCATTGACTGCTAGGGCTCCTAACCCCAAGTGTCA
CAGTTGGGACCCTTGCCTGAGAAAGTTAAATATACATCTATAGTTCCTGTAGTAGAGAG
ACAGACATCAATGGATGACTTGTGTGACTGGCTCTTACATGCTGCTCGTGATCTTCCAG
TCCCACTGTGCCCACAGAAGACTCTGGGGTACAGCAATCAGGGTAGACCCTTTGTTTCC
>3 OSNs_Olfr49
CAGGGGGGCGGAGCCTATGGAAAAACGCCAGCAACGCGGCCTTTTTACGGTTCCTGGCC
TTTTGCTGGCCTTTTGCTCACATGTTCTTTCCTGCGTTATCCCCTGATTCTGTGGATAA
CCGTATTACCGCCTTTGAGTGAGCTGATACCGCTCGCCGCAGCCGAACGACCGAGCGCA
GCGAGTCAGTGAGCGAGGAAGCGGAAGAGCGCCCAATACGCAAACCGCCTCTCCCCGCG
CGTTGGCCGATTCATTAATGCAGCTGGCACGACAGGTTTCCCGACTGGAAAGCGGGCAG
TGAGCGCAACGCAATTAATGTGAGTTAGCTCACTCATTAGGCACCCCAGGCTTTACACT
TTATGCTTCCGGCTCGTATGTTGTGTGGAATTGTGAGCGGATAACAATTTCACACAGGA
AACAGCTATGACCATGATTACGCCAAGCTATTTAGGTGACACTATAGAATACTCAAGCT
ATGCATCCAACGCGTTGGGAGCTCTCCCATATGGTCGACCTGCAGGCGGCCGCGAATTC
ACTAGTGATTGCATGGCAAAGTTGCGGAGGAAGTAATACATGGGGGTGTAAAGGCGCCT
GTCCACAAGGGTGATGAAGATGATGAGGAAGTTTCCCAGCAGAATGAGGAAGTAGGTCA
GGAGAAAGCCCAGGAATATGAGCACCTGCAGCTCACAGGCATCTGACAGCCCCAGCAAA
ATAAACTCAGTAACAGTAGTGCTGTTCGCCATGGCTCCTCCCACCTCTGCTGGGGATGG
TCATGGACCCTGCTGAGCATCAGAGCCTGTGAGCTCTGAGGACAACACAAAGCTGTGTG
TATATGTCTGGGTGAGTGCAGGTGTGTGATGTGAGTGCAGATGCCCTCAGTAACCAGCA
GTACTGGATCCCCTGGAGCTGAAGTTGCCCGACACGGAGGCTGGGAGTTGAAGTCGGTC
CTCTGCTGGAGCAGTATGTGCTCCTAGCTTCTTAACCATCTCTTCATTCCCAGTATGCT
GCTCGTGATCTTCCAGTCCCACTGTGCCCACAAAAGACTCTGGGGTACAGCAATCAGGG
TAGACCCTTTGTTTCTC
>4 OSNs_Olfr49
GCATGGCAAAGTTGCGGAGGAAGTAATACATGGGGGTGTAAAGGCGCCTGTCCACAAGG
GTGATGAAGATGATGAGGAAGTTTCCCAGCAGAATGAGGAAGTAGGTCAGGAGAAAGCC
CAGGAATGTGAGCACCTGCAGCTCACAGGCATCTGACAGCCCCAGCAAAATAAACTCAG
TAACAGTAGTGCTGTTCGCCATGGCTCCTCCCACCTCTGCTGGGGATGGTCATGGACCC
TGCTGAGCATCAGAGCCTGTGAGCTCTGAGGACAACACAAAGCTGTGTGTATATGTCTG
GGTGAGTGCAGGTGTGTGATGTGAGTGCAGATGCCCTCAGTAACCAGCAGTACTGGATC
CCCTGGAGCTGAAGTTGCCCGACACGGAGGCTGGGAGTTGAAGTCGGTCCTCTGCTGGA
GCAGTATGTGCTCCTAGCTTCTTAACCATCTCTTCATTCCCAGTCTGTGAAGATTTTGA
TGCAGGGCTGAGAGGTAGCTCAGCGGCACACCCCTTGTCTGGTGTGCGTGAGGCTGGTT
TCAAACACCCCAGTTCTGTTTTCTCTTTCTCTGTTGCGTTTTCTATCGTTATTTAATAT
GCTGCTCGTGATCTTCCAGTCCCACTGTGCCCACAGAAGACTCTGGGGTACAGCAATCA
GGGTAGACCCT
>5 OSNs_Olfr49
GCATGGCAAAGTTGCGGAGGAAGTAATACATGGGGGTGTAAAGGCGCCTGTCCACAAGG
GTGATGAAGATGATGAGGAAGTTTCCCAGCAGAATGAGGAAGTAGGTCAGGAGAAAGCC
CAGGAATATGAGCACCTGCAGCTCACAGGCATCTGACAGCCCCAGCAAAATAAACTCAG
TAACAGTAGTGCTGTTCGCCATGGCTCCTCCCACCTCTGCTGGGGATGGTCATGGACCC

TGCTGAGCATCAGAGCCTGTGAGCTCTGAGGACAACACAAAGCTTAAGGTCTACCGTAT
GTTCTGCAGATCACAATCAGGCAGCTCTTGTCCCATTACACTTCACTAAACCTTCAGTT
TTTCCAGCGACCCACCTCCCTTGCAATACTCCCTCCCTCGGTCCTCTCAGAAGTGAGTT
GTCTCTGGGTACTTACTAGAATTGGGATTACCAACTCATTGACTGCTAGGGCTCCTAAC
CCCAAGTGTCAC
>6 OSNs_Olfr49
GCATGGCAAAGTTGCGGAGGAAGTAATACATGGGGGTGTAAAGGCGCCTGTCCACAAGG
GTGATGAAGATGATGAGGAAGTTTCCCAGCAGAATGAGGAAGTAGGTCAGGAGAAAGCC
CAGGAATATGAGCACCTGCAGCTCACAGGCATCTGACAGCCCCAGCAAAATAAACTCAG
TAACAGTAGTGCTGTTCGCCATGGCTCCTCCCACCTCTGCTGGGGATGGTCATGGACCC
TGCTGAGCATCAGAGCCTGTGAGCTCTGAGGACAACACAAAGCTGTGTGTATATGTCTG
GGTGAGTGCAGGTGTGTGATGTGAGTGCAGATGCCCTCAGTAACCAGCAGTACTGGATC
CCCTGGAGCTGAAGTTGCCCGACACGGAGGCTGGGAGTTGAAGTCGGTCCTCTGCTGGA
GCAGTATGTGCTCCTAGCTTCTTAACCATCTCTTCATTCCCAGTATGCTGCTCGTGATC
TTCCAGTCCCACTGTGCCCACAGAAGACTCTGGGGTACAGCAATCAGGGTAGACCCTTT
GTTTCTC
>7 OSNs_Olfr49
GCATGGCAAAGTTGCGGAGGAAGTAATACATGGGGGTGTAAAGGCGCCTGTCCACAAGG
GTGATGAAGATGATGAGGAAGTTTCCCAGCAGAATGAGGAAGTAGGTCAGGAGAAAGCC
CAGGAATATGAGCACCTGCAGCTCACAGGCATCTGACAGCCCCAGCAAAATAAACTCAG
TAACAGTAGTGCTGTTCGCCATGGCTCCTCCCACCTCTGCTGGGGATGGTCATGGACCC
TGCTGAGCATCAGAGCCTGTGAGCTCTGAGGACAACACAAAGCTGTGTGTATATGTCTG
GGTGAGTGCAGGTGTGTGATGTGAGTGCAGATGCCCTCAGTAACCAGCAGTACTGGATC
CCCTGGAGCTGAAGTTGCCCGACACGGAGGCTGGGAGTTGAAGTCGGTCCTCTGCTGGA
GCAGTATGTGCTCCTAGCTTCTTAACCATCTCTTCATTCCCAGTCTTAAGGTCTACCGT
ATGTTCTGCAGATCACAATCAGGCAGCTCTTGTCCCATTACACTTCACTAAACCTTCAG
TTTTTCCCGCGACCCACCTCCCTTGCAATACTCCCTCCCTCGGTCCTCTCAGAAGTGAG
TTGTCTCTGGGTACTTACTAGAATTGGGATTACCAACTCATTGACTGCTAGGGCTCCTA
ACCCCAAGTGTCAC
>8 OSNs_Olfr49
CAGTAACCAGTAGTGCTGTTCGCCCATGGCTCCTTCCNNCCTTTNGCTGGGGATGGTCA
TGGACCCTGCTGAGCATCAGAGCCTGTGAGCTCTGAGGACAACACAAAAGCTGTGTGTA
TATGTCTGGGTGAGTGCAGGTGTGTGATGTGAGTGCAGATGCCCTCAGTAACCAGCAGT
ACTGGATCCCCTGGAGCTGAAGTTGCCCGACACGGAGGCTGGGAGTTGAAGTCGGTCCT
CTGCTGGAGCAGTATGTGCTCCTAGCTTCTTAACCATCTCTTCATTCCCAGTCTGTGAA
GATTTTGATGCAGGGCTGAGAGGTAGCTCAGCGGCACACCCCTTGTCTGGTGTGCGTGA
GGCTGGTTTCAAACACCACAGTTCTGTTTTCTCTTTCTCTGTTGCGTTTTCTATCGTTA
TTTAATCTACATATTGGTTTATACAGATTATGCATTTTACCCTTCTTCTTTTAAGATAG
TAATAAAATAAAATGACATTATTATAAACTTTTTGTAATTATTTGGCTACTATTTTTGA
AGACTTCAGGTACACGAAACTGATACATAGATAAGAAAGCATGCATTTTTATCATGTTT
AGCTTTTGTATAACATCCTCAATAATCAACACAAATAACCACTCAGAGTCATCCATGGA
TTCAATCTCAATTGTTCAGATTATGTTTTTACCTTAAGGTCTACCGTATGTTCTGCAGA
TCACAATCAGGCAGCTCTTGTCCCATTACACTTCACTAAACCTTCAGTTTTTCCAGCGA
CCCACCTCCCTTGCAATACTCCCTCCCTCGGTCCTCTCAGAAGTGAGTTGTCTCTGGGT
ACTTACTAGAATTGGGATTACCAACTCATTGACTGCTAGGGCTCCTAACCCCAAGTGTC
ACAGTTGGGACCCTTGCCTGAGAAAGTTAAATATACGTCTATAGTTCCTGTAGTAGAGA
GACAGACATCAATGGATGACTTGTGTGACTGGCTCTTACATGCTGCTCGTGATCTTCCA

GTCCCACTGTGCCCACAGAAGACTCTGGGGTACAGCAATCAGGGTAGACCCTTTGTTTC
TC
>9 OSNs_Olfr49
GCATGGCAAAGTTGCGGAGGAAGTAATACATGGGGGTGTAAAGGCGCCTGTCCACAAGG
GTGATGAAGATGATGAGGAAGTTTCCCAGCAGAATGAGGAAGTAGGTCAGGAGAAAGCC
CAGGAATATGAGCACCTGCAGCTCACAGGCATCTGACAGCCCCAGCAAAATAAACTCAG
TAACAGTAGTGCTGTTCGCCATGGCTCCTCCCACCTCTGCTGGGGATGGTCATGGACCC
TGCTGAGCATCAGAGCCTGTGAGCTCTGAGGACAACACAAAGCTTAAGGTCTACCGTAT
GTTCTGCAGATCACAATCAGGCAGCTCTTGTCCCATTACACTTCACTAAACCTTCAGTT
TTTCCAGCGACCCACCTCCCTTGCAATACTCCCTCCCTCGGTCCTCTCAGAAGTGAGTT
GTCTCTGGGTACTTACTAGAATTGGGATTACCAACTCATTGACTGCTAGGGCTCCTAAC
CCCAAGTGTCACC
>10 OSNs_Olfr49
GGCCGCGGGAATTCGATTGCATGGCAAAGTTGCGGAGGAAGTAATACATGGGGGTGTAA
AGGCGCCTGTCCACAAGGGTGATGAAGATGATGAGGAAGTTTCCCAGCAGAATGAGGAA
GTAGGTCAGGAGAAAGCCCAGGAATATGAGCACCTGCAGCTCACAGGCATCTGACAGCC
CCAGCAAAATAAACTCAGTAACAGTAGTGCTGTTCGCCATGGCTCCTCCCACCTCTGCT
GGGGATGGTCATGGACCCTGCTGAGCATCAGAGCCTGTGAGCTCTGAGGACAACACAAA
GCTGGAAATACAGAGGAATGAGTATTACACTTTCAGCTGGCGGAGTCCTTTAGAGGTTT
CATCTCCTTAGCGTCCTGTTCATCAGGCTGGTGAGTAAGATGTCTGGCTGAGTCAACAG
CATATCATAAACGTTGACATCACTTTACAAAATTCGGGGTAATTCTCTACCCCAACTGA
TTTTAAACAACTTTGTATGTGACCTATTTTTTTCTGCTAGGATTTTTTTATTATTATTT
TAGATCAG
>11 OSNs_Olfr49
AGGAATNTGAGNNCCTGCAGNNCNCAGGCATCTGACAGCCCCAGCAAAATAANNTCAGT
ANCAGTAGTGCTGTTCGCCATGGCTCCTCCCACCTNTGCTGGGGATGGTCATGGACCCT
GCTGAGCATCAGAGCCTGTGAGCTCTGAGGACAACACAAAGCTGTGTGTATATGTCTGG
GTGAGTGCAGGTGTGTGATGTGAGTGCAGATGCCCTCAGTAACCAGCAGTACTGGATCC
CCTGGAGCTGAAGTTGCCCGACACGGAGGCTGGGAGTTGAAGTCGGTCCTCTGCTGGAG
CAGTATGTGCTCCTAGCTTCTTAACCATCTCTTCATCCCCAGTCTGTGAAGATTNTGAT
GCAGGGCTGAGAGGTAGCTCAGCGGCACACCCCTTGTCTGGTGTGCGTGAGGCTGGTTT
CAAACACCACAGTTCTGTTTTCTCTTTCTCTGTTGCGTTTTCTATCGTTATTTAATCTA
CATATTGGTTTATACAGATTATGCATTTTACCCTTCTTCTTTTAAGATAGTAATAAAAT
AAAATGACATTATTATAAACTTTTTGTAATTATTTGGCTACTATTTTTGAAGACTTCAG
GTACACGAAACTGATACATAGATAAGAAAGCATGTATTTTTATCATGTTTAGCTTTTGT
ATAACATCCTCAATAATCAACACAAATAACCACTCAGAGTCATCCATGGATTCAATCTC
AATTGTTCAGATTATGTTTTTACCTTAAGGTCTACCGTATGTTCTGCAGATCACAATCA
GGCAGCTCTTGTCCCATTACACTTCACTAAACCTTCAGTTTTTCCAGCGACCCACCTCC
CTTGCAATACTCCCTCCCTCGGTCCTCTCAGAAGTGAGTTGTCTCTGGGTACTTACTAG
AATTGGGATTACCAACTCATTGACTGCTAGGGCTCCTAACCCCAAGTGTCACAGTTGGG
ACCCTTGCCTGAGAAAGTTAAATATACATCTATAGTTCCTGTAGTAGAGAGACAGACAT
CAATGGATGACTTGTGTC

>1 WOM_Olfr266
GAAGAGCATGGTTGCACAGCACTAGAAAAACAATAAGGATGTTTCCAGAAATGGTTAGG
ATGTAGAAGATGCAAAACATTATAAAGAGGGAAATATTCAGCTGTAAGATGGAAGAGAA

TCCTCGAAGAACAAACTCCACTATCACCGTTTGATTTTCCCAGGATAAGGTCATCATAC
TTTGGTTTCCTGGATTTAAATAATC
>2 WOM_Olfr266
TGTGAAGAGCATGGTTGCACAGCACTAGAAAAACAATAAGGATGTTTCCAGAAATGGTT
AGGATGTAGAAGATGCAAAACATTATAAAGAGGGAAATATTCAGCTGTAAGATGGAAGA
GAATCCTCGAAGAACAAACTCCACTATCACCGTTTGATTTTCCCAGGATAAGGTCATCA
TACTTTGGTTTTCAGGGTTGCTTCATACCCTGGATATAGCACAGAGAATCCCTGGATTT
AAATAATAACATTAGATGTGTGGAATCTTGCTTTTATCCAC
>3 WOM_Olfr266
TGTGAAGAGCATGGTTGCACAGCACTAGAAAAACAATAAGGATGTTTCCAGAAATGGTT
AGGATGTAGAAGATGCAAAACATTATAAAGAGGGAAATATTCAGCTGTAAGATGGAGGA
GAGTCCTCGAAGAACAAACTCCACTATCACCGTTTGATTTTCCCAGGATAAGGTCATCA
TACTTTGGTTTTCAGGGTTGCTTCATACCCTGGATATAGCACAGAGAAT
>4 WOM_Olfr266
TGTGAAGAGCATGGTTGCACAGCACTAGAAAAACAATAAGGATGTTTCCAGAAATGGTT
AGGATGTAGAAGATGCAAAACATTATAAAGAGGGAAATATTCAGCTGTAAGATGGAAGA
GAATCCTCGAAGAACAAACTCCACTATCACCGTTTGATTTTCCCAGGATAAGGTTATCA
TACTTTGGTTTTCAGGGTTGCTTCATACCCTGGATATAGCACAGAGAAT
>5 WOM_Olfr266
TGTGAAGAGCATGGTTGCACAGCACTAGAAAAACAATAAGGATGTTTCCAGAAATGGTT
AGGATGTAGAAGATGCAAAACATTATAAAGAGGGAAATATTCAGCTGTAAGATGGAAGA
GAATCCTCGAAGAACAAACTCCACTATCACCGTTTGATTTTCCCAGGATAAGGTCATCA
TACTTTGGTTTTCAGGGTTGCTTCATACCCTGGATATAGCA
>6 WOM_Olfr266
TGTGAAGAGCATGGTTGCACAGCACTAGAAAAACAATAAGGATGTTTCCAGAAATGGTT
AGGATGTAGAAGATGCAAAACATTATAAAGAGGGAAATATTCAGCTGTAAGATGGAAGA
GAATCCTCGAAGAACAAACTCCACTATCACCGTTTGATTTTCCCAGGATAAGGTCATCA
TACTTTGGTTTTCAGGGTTGCTTCATACCCTGGATATAGCACAGAGAAT

>1 OSNs_Olfr266
TGTGAAGAGCATGGTTGCACAGCACTAGAAAAACAATAAGGATGTTTCCAGAAATGGTT
AGGATGTAGAAGATGCAAAACATTATAAAGAGGGAAATATTCAGCTGTAAGATGGAAGA
GAATCCTCGAAGAACAAACTCCACTATCACCGTTTGATTTTCCCAGGATAAGGTCATCA
TACTTTGGTTTTCAGGGTTGCTTCATACCCTGGATATAGCACAGAGAATCCAGAACAGA
TCCGCTTTGCCCATCAGAGACTTGGAGAGGTCCATTCCTAATTTGCAGAGCCAGACCAC
ATAGAATC
>2 OSNs_Olfr266
TGTGAAGAGCATGGTTGCACAGCACTAGAAAAACAATAAGGATGTTTCCAGAAATGGTT
AGGATGTAGAAGATGCAAAACATTATAAAGAGGGAAATATTCAGCTGTAAGATGGAAGA
GAATCCTCGAAGAACAAACTCCACTATCACCGTTTGATTTTCCCAGGATAAGGTCATCA
TACTTTGGTTTCTGTGAAGGACACGATTATAAAAGCATACATAATATAGCTGTTTCTAC
TTTATGCAAGAATGTGAACAGAATGGTTTTTATTCTATGCTCTCTCTACT
>3 OSNs_Olfr266
AATAAGGATGTTTCCAGAAATGGTTAGGATGTAGAAGATGCAAAACATTATAAAGAGGG
AAATATTCAGCTGTAAGATGGAAGAGAATCCTCGAAGAACAAACTCCACTATCACCGTT
TGATTTTCCCAGGATAAGGTCATCATACTTTGGTTTTCTTTCCCCTCTACCAAGTGATT

GCCCCACTTACTCAGGGTTGCTTCATACCCTGGATATAGCACAGAGAATCCCTGGATTT
AAATAATAACATTAGATGTGTGGAATCTTGCTTTTATCCC
>4 OSNs_Olfr266
TGTGAAGAGCATGGTTGCACAGCACTAGAAAAACAATAAGGATGTTTCCAGAAATGGTT
AGGATGTAGAAGATGCAAAACATTATAAAGAGGGAAATATTCAGCTGTAAGATGGAAGA
GAATCCTCGAAGAACAAACTCCACTATCACCGTTTGATTTTCCCAGGATAAGGTCATCA
TACTTTGGTTTCTGTGAAGGACACGATTATAAAAGCATACATAATATAGCTGTTTCTAC
TTTATGCAAGAATGTGAACAGAATGGTTTTTATTCTATGCTCTCTCTAC
>5 OSNs_Olfr266
TGTGAAGAGCATGGTTGCACAGCACTAGAAAAACAATAAGGATGTTTCCAGAAATGGTT
AGGATGTAGAAGATGCAAAACATTATAAAGAGGGAAATATTCAGCTGTAAGATGGAAGA
GAATCCTCGAAGAACAAACTCCACTATCACCGTTTGATTTTCCCAGGATAAGGTCATCA
TACTTTGGTTTCTGTGAAGGACACGATTATAAAAGCATACATAATATAGCTGTTTCTAC
TTTATGCAAGAATGTGAACAGAATGGTTTTTATTCTATGCTCTCTCTAC
>6 OSNs_Olfr266
TGTGAAGAGCATGGTTGCACAGCACTAGAAAAACAATAAGGATGTTTCCAGAAATGGTT
AGGATGTAGAAGATGCAAAACATTATAAAGAGGGAAATATTCAGCTGTAAGATGGAAGA
GAATCCTCGAAGAACAAACTCCACTATCACCGTTTGATTTTCCCAGGATAAGGTCATCA
TACTTTGGTTTCTAACCCAGAAGCTC
>7 OSNs_Olfr266
TGTGAAGAGCATGGTTGCACAGCACTAGAAAAACAATAAGGATGTTTCCAGAAATGGTT
AGGATGTAGAAGATGCAAAACATTATAAAGAGGGAAATATTCAGCTGTAAGATGGAAGA
GAATCCTCGAAGAACAAACTCCACTATCGCCGTTTGATTTTCCCAGGATAAGGTCATCA
TACTTTGGTTTTCAGGGTTGCTTCATACCCTGGATATAGCACAGAGAATCCCTGGATTT
AAATAATC
>8 OSNs_Olfr266
TGTGAAGAGCATGGTTGCACAGCACTAGAAAAACAATAAGGATGTTTCCAGAAATGGTT
AGGATGTAGAAGATGTAAAACATTATAAAGAGGGAAATATTCAGCTGTAAGATGGAAGA
GAATCCTCGAAGAACAAACTCCACTATCACCGTTTGATTTTCCCAGGATAAGGTCATCA
TACTTTGGTTTCTGTGAAGGACACGATTATAAAAGCATACATAATATAGCTGTTTCTAC
TTTATGCAAGAATGTGAACAGAATGGTTTTTATTCTATGCTCTCTCTACT
>9 OSNs_Olfr266
TGTGAAGAGCATGGTTGCACAGCACTAGAAAAACAATAAGGATGTTTCCAGAAATGGTT
AGGATGTAGAAGATGCAAAACATTATAAAGAGGGAAATATTCAGCTGTAAGATGGAAGA
GAATCCTCGAAGAACAAACTCCACTATCACCGTTTGATTTTCCCAGGATAAGGTCATCA
TACTTTGGTTTCTGTGAAGGACACGATTATAAAAGCATACATAATATAGCTGTTTCTAC
TTTATGCAAGAATGTGAACAGAATGGTTTTTATTCTATGCTCTCTCTACT

>1 WOM_Olfr267
CGTGAATCCAGGACAGTGATTAAGATAAGAGTACAGTTACCCAGGATGGTAACCAGATA
CATGACAAGGCTGAAGACAAACCGAACAATCTCTAACCTC
>2 WOM_Olfr267
TTTGAAGGCGTGAATCCAGGACAGTGATTAAGATAAGAGTACAGTTACCCAGGATGGTA
ACCAGATACATGACAAGGCTGAAGACAAACAGAACAATCTCTAACCTCGGGTACCGGGA
AAATCCCTCTAGGAAAAAAAGGCTCCAAACAGTGACATTTTCTCCTGGCATTTCCTCAC
GAGCACCACCAGCACCAACAGCTATTCTAGAGAGATTCTCTTCATTTCATATTGCTCAT
TACCACTGTCAAACCTTTTCTTT

>3 WOM_Olfr267
TTTGAAGGCGTGAATCCAGGACAGTGATTAAGATAAGAGTACAGTTACCCAGGATGGTA
ACCAGATACATGACAAGGCTGAAGACAAACAGAACAATCTCTAACCTC
>4 WOM_Olfr267
TTTGAAGGCGTGAATCCAGGACAGTGATTAAGATAAGAGTACAGTTACCCAGGATGGTA
ACCAGATACATGACAAGGCTGAAGACAAACAGAACAATCTCTAACCTCGGGTACCGGGA
AAATCCCTCTAGGAAAAAAAGGCTCCAAACAGTGACATTTTCTCCTGGCATTTCCTCAC
GAGCACCACCAGCACCAACAGCTATTCTAGAGAGATTCTCTTCATTTCATATTGCTCAT
TACCACTGTCAAACCTTTTCTTTCCCTTCTGATTCTCTCCAGACTTTCTTCAAACACAT
ATGCTCTCAAATATTCCTTCCACTGGCATCCTGCCATGTTGCCACTGTCTCTGCTTGCT
TTCTTAGAAAGATCCACCGGTCAGATGGTCCATGTCACAGTACTTCAGCCTTTGTTTCT
GTTTCTTGCTTCCAACCAGTCAGTCTTCTCCACATTCTCTGAATCCCATGCTATAAACT
CCAAATAAAAATGTTTTATGATATGTTCCACAAGTTAGTTATGGTTGGTTATATGTAAA
GAAGAATGAATATATCTACATTTAAAATCTGAAATGCTATAGAAAAATCAATTATGCAA
TGCATTTCTTTCAAAATGACCAGATGTTACAAACATAACCAAAATTC
>5 WOM_Olfr267
TTTGAAGGCGTGAATCCAGGACAGTGATTAAGATAAGAGTACAGTTACCCAGGATGGTA
ACCAGATACATGACAAGGCTGAAGACAAACAGAACAATCTCTAACCTCGGGTACCGGGA
AAATCCCTCTAGGAAAAAAAGGCTCCAAACAGTGACATTTTCTCCTGGCATTTCCTCAC
GAGCACCACCAGCACCAACAGCTATTCTAGAGAGATTCTCTTCATTTCATATTGCTCAT
TACCACTGTCAAACCTTTTCTTTCCCTTCTGATTCTCTCCAGACTTTCTTCAAACACAT
ATGCTCTCAAATATTCCTTCCACTGGCATCCTGCCATGTTGCCACTGTCTCTGCTTGCT
TTCTTAGAAAGATCCACCGGTCAGATGGTCCATGTCACAGTACTTCAGCCTTTGTTTCT
GTT
>6 WOM_Olfr267
TTTGAAGGCGTGAATCCAGGACAGTGATTAAGATAAGAGTACAGTTACCCAGGATGGTA
ACCAGATACATGACAAGGCTGAAGACAAACAGAACAATCTCTAACCTCGGGTACCGGGA
AAATCCCTCTAGGAAAAAAAGGCTCCAAACAGTGACATTTTCTCCTGGCATTTCCTCAC
GAGCACCACCAGCACCAACAGCTATTCTAGAGAGATTCTCTTCATTTCATATTGCTCAT
TACCACTGTCAAACCTTTTCTTT
>7 WOM_Olfr267
TTTGAAGGCGTGAATCCAGGACAGTGATTAAGATAAGAGTACAGTTACCCAGGATGGTA
ACCAGATACATGACAAGGCTGAAGACAAACAGAACAATCTCTAACCTC
>8 WOM_Olfr267
TTTGAAGGCGTGAATCCAGGACAGTGATTAAGATAAGAGTACAGTTACCCAGGATGGTA
ACCAGATACATGACAAGGCTGAAGACAAACAGAACAATCTCTAACCTCGGGTACCGGGA
AAATCCCTCTAGGAAAAAAAAGGCTCCAAACAGTGACATTTTCTCCTGGCATTTCCTCA
CGAGCACCACCAGCACCAACAGCTATTCTAGAGAGATTCTCTTCATTTCATATTGCTCA
TTACCACTGTCAAACCTTTTCTTTCCCTTCTGATTCTCTCCAGACTTTCTTCAAACACA
TATGCTCTCAAATATTCCTTCCACTGGCATCCTGCCATGTTGCCACTGTCTCTGCTTGC
TTTCTTAGAAAGATCCACCGGTCAGATGGTCCGTGTCACAGTACTTCAGCCTTTGTTTC
TGTTTCTTGCTTCCAACCAGTCAGTCTTCTCCACATTCTCTGCCTGAATCCCATGCTAT
AAACTCCAAATAAAAATGTTTTATGATATGTTCCACAAGTTAGTTATGGTT
>9 WOM_Olfr267
TTTGAAGGCGTGAATCCAGGACAGTGATTAAGATAAGAGTACAGTTACCCAGGATGGTA
ACCAGATACATGACAAGGCTGAAGACAAACAGAACAATCTCTAACCTCGGGTACCGGGA
AAATCCCTCTAGGAAAAAAAGGCTCCAAACAGTGACATTTTCTCCTGGCATTTCCTCAC

GAGCACCACCAGCACCAACAGCTATTCTAGAGAGATTCTCTTCATTTCATATTGCTCAT
TACCACTGTCAAACCTTTTCTTT

>1 OSNs_Olfr267
TTGAAGGCGTGAATCCAGGACAGTGATTAAGATAAGAGTACAGTTACCCAGGATGGTAA
CCAGATACATGACAAGGCTGAAGACAAACAGAACAATCTCTAACCTCGGGTACCGGGAA
AATCCCTCTAGGAAAAAAAGGCTCCAAACAGTGACATTTTCTCCTGGCATTTCCTCACG
AGCACCACCAGCACCAACAGCTATTCTAGAGAGATTCTCTTCATTTCATATTGCTCATT
ACCACTGTCAAACCTTTTCTTTCCCTTCT
>2 OSNs_Olfr267
GGCCGCGGGAATTCGATTTTTGAAGGCGTGAATCCAGGACAGTGATTAAGATAAGAGTA
CAGTTACCCAGGATGGTAACCAGATACATGACAAGGCTGAAGACAAACAGAACAATCTC
TAACCTCGGGTACCGGGAAAATCCCTCTAGGAAAAAAAGGCTCCAAACAGTGACATTTT
CTCCTGGCATTTCCTCACGAGCACCACCAGCACCAACAGCTATTCTAGAGAGATTCTCT
TCATTTCATCTGAGGAGTATAAATACATATAATGTCCATTATGCTA
>3 OSNs_Olfr267
GGCCGCGGGAATTCGATTTTTGAAGGCGTGAATCCAGGACAGTGATTAAGATAAGAGTA
CAGTTACCCAGGATGGTAACCAGATACATGACAAGGCTGAAGACAAACAGAACAATCTC
TAACCTCGGGTACCGGGAAAATCCCTCTAGGAAAAAAAGGCTCCAAACAGTGACATTTT
CTCCTGGCATTTCCTCACGAGCACCACCAGCACCAACAGCTATTCTAGAGAGATTCTCT
TCATTTCATCTGAGGAGTATAAATACATATAATGTCCATTATGCTA
>4 OSNs_Olfr267
TTTGAAGGCGTGAATCCAGGACAGTGATTAAGATAAGAGTACAGTTACCCAGGATGGTA
ACCAGATACATGACAAGGCTGAAGACAAACAGAACAATCTCTAACCTCGGGTACCGGGA
A
>5 OSNs_Olfr267
GGCCGCGGGAATTCGATTTTGAAGGCGTGAATCCAGGACAGTGATTAAGATAAGAGTAC
AGTTACCCAGGATGGTAACCAGATACATGACAAGGCTGAAGACAAACAGAACAATCTCT
AACCTCGGGTACCGGGAAAATCCCTCTAGGAAAAAAAGGCTCCAAACAGTGACATTTTC
TCCTGGCATTTCCTCACGAGCACCACCAGCACCAACAGCTATTCTAGAGAGATTCTCTT
CATTTCGTCTGAGGAGTATAAATACATATAATGTCCATTATGCTA
>6 OSNs_Olfr267
GGCCGCGGGAATTCGATTTTTGAAGGCGTGAATCCAGGACAGTGACTAAGATAAGAGTA
CAGTTACCCAGGATGGTAACCAGATACATGACAAGGCTGAAGACAAACAGAACAATCTC
TAACCTCGGGTACCGGGAAAATCCCTCTAGGAAAAAAAGGCTCCAAACAGTGACATTTT
CTCCTGGCATTTCCTCACGAGCACCACCAGCACCAACAGCTATTCTAGAG
>7 OSNs_Olfr267
GGCCGCGGGAATTCGATTTTTGAAGGCGTGAATCCAGGACAGTGATTAAGATAAGAGTA
CAGTTACCCAGGATGGTAACCAGATACATGACAAGGCTGAAGACAAACAGAACAATCTC
TAACCTCGGGTACCGGGAAAATCCCTCTAGGAAAAAAAGGCTCCAAACAGTGACATTTT
CTCCTGGCATTTCCTCACGAGCACCACCAGCACCAACAGCTATTCTAGAGAGATTCTCT
TCATTTCATCTGAGGAGTATAAATACATATAATGTCCATTATGCTAGAA
>8 OSNs_Olfr267
GGCCGCGGAATTCGATTGCGTGAATGACAGTGATTAAGATAAGAGTACAGTACCCAGGA
TGGTAACCAGATACATGACAAGGCTGAAGACAAACAGAACAATCTCTAACCTCGGGTAC
CGGGAAAATCCCTCTAGGAAAAAAAGGCTCCAAACAGACATTTTCTCTGGCATTTCCTC

ACGAGCACCACCAGCACCAACAGCTATTCTAGAGAGATTCTCTTCATTTCATCTGAGGA
GTATAAATACATATAATGTCCATTATGCTA
>9 OSNs_Olfr267
GGCCGCGGGAATTCGATTTTTGAAGGCGTGAATCCAGGACAGTGATTAAGATAAGAGTA
CAGTTACCCAGGATGGTAACCAGATACATGACAAGGCTGAAGACAAACAGAACAATCTC
TAACCTCGGGTACCGGGAAAATCCCTCTAGGAAAAAAAGGCTCCAAACAGTGACATTTT
CTCCTGGCATTTCCTCACGAGCACCACCAGCACCAACAGCTATTCTAGAG

>1 WOM_Olfr370
GTACGGCTGTCAATCTCCTGTGCTTCTCGAGTGTGTAAGGATTTCCTCAGTGCCCTCTC
CCCATCTGGGGCTGATCTCTGATGACAGAGCCTCCATGGAGTGTGTAAATGACACTGTG
GTCAGAGAATTTGTCTTCCTTGGCTTCTCGTCTCTGGCTG
>2 WOM_Olfr370
GTACGGCTGTCAATCTCCTGTGCTTCTCGAGTGTGTAAGGATTTCCTCAGTGCCCTCTC
CCCATCTGGGGCTGATCTCTGATGACAGAGCCTCCATGGAGTGTGTAAATGACACTGTG
GTCAGAGAATTTGTCTTCCTTGGCTTCTCGTCTCTGGCTG
>3 WOM_Olfr370
ACTTGAGCGTCGATTTTTGTGATGCTCGTCAGGGGGGCGGAGCCTATGGAAAAACGCCA
GCAACGCGGCCTTTTTACGGTTCCTGGCCTTNTGCTGGCCTTTTGCTCACATGTTCTNT
CCTGCGTTATCCCCTGATTCTGTGGATAACCGTATTACCGCCTTTGAGTGAGCTGATAC
CGCTCGCCGCAGCCGAACGACCGAGCGCAGCGAGTCAGTGAGCGAGGAAGCGGAAGAGC
GCCCAATACGCAAACCGCCTCTCCCCGCGCGTTGGCCGATTCATTAATGCAGCTGGCAC
GACAGGTTTCCCGACTGGAAAGCGGGCAGTGAGCGCAACGCAATTAATGTGAGTTAGCT
CACTCATTAGGCACCCCAGGCTTTACACTTTATGCTTCCGGCTCGTATGTTGTGTGGAA
TTGTGAGCGGATAACAATTTCACACAGGAAACAGCTATGACCATGATTACGCCAAGCTA
TTTAGGTGACACTATAGAATACTCAAGCTATGCATCCAACGCGTTGGGAGCTCTCCCAT
ATGGTCGACCTGCAGGCGGCCGCGAATTCACTAGTGATTAAGCAGTGGTATCAACGCAG
AGTACATGCTGTACGGCTGTCAATCTCCTGTGCTTCTCGAGTGTGTAAGGATTTCCTCA
GTGCCCTCTCCCCATCTGGGGCTGATCTCTGATGACAGAGCCTCCATGGAGTGTGTAAA
TGACACTGTGGTCAGAGAATTTGTCTTCCTTGGCTTCTCGTCTCTGGCTG
>4 WOM_Olfr370
GGTTTCAGTCTGGGTCTCAGACCCCAGACTATGATTCCATACCTCCCTGAGTAGACAGC
AGCAAACTTTAACTCACCTCGTACTTCCTCTTCTTCTAGCAGTCTATGTGACTTCAGTC
CTGGTGCCTCTGTGTCACTGGGCTTTCTCAGGAACAGAAAAGAAGGAAGAGAAGCTCGT
CCATGGACAGCTGACATCTTCCTTCAGCTGACACCACTAACACTGGGGAATAAGATTAA
GCATCTACCTCCAACAACACATAGGTTTTCATATCTCAGGAAGGAGGAGAAATGGCAAG
TTTAGCAGGAGGGTCTGGGAGCACATGCTGTACGGCTGTCAATCTCCTGTGCTTCTCGA
GTGTGTAAGGATTTCCTCAGTGCCCTCCCCCCATCTGGGGCTGATCTCTGATGACAGAG
CCTCCATGGAGTGTGTAAATGACACTGTGGTCAGAGAATTTGTCTTCCTTGGCTTCTCG
TCTCTGGCTG
>5 WOM_Olfr370
AAACAAGCAGAGACACGGAAGCACAGCGGTCCTCTTTCCTGTCCTGTGGGATACACACT
TCAGAAGAGCATGGAAGGAGGAGAAATGGCAAGTTTAGCAGGAGGGTCTGGGAGCACAT
GCTGTACGGCTGTCAATCTCCTGTGCTTCTCGAGTGTGTAAGGATTTCCTCAGTGCCCT
CTCCCCATCTGGGGCTGATCTCTGATGACAGAGCCTCCATGGAGTGTGTAAATGACACT
GTGGTCAGAGAATTTGTCTTCCTTGGCTTCTCGTCTCTGGCTG
>6 WOM_Olfr370

GTACGGCTGTCAATCTCCTGTGCTTCTCGAGTGTGTAAGGATTTCCTCAGTGCCCTCTC
CCCATCTGGGGCTGATCTCTGATGACAGAGCCTCCATGGAGTGTGTAAATGACACTGTG
GTCAGAGAATTTGTCTTCCTTGGCTTCTCGTCTCTGGCTG
>7 WOM_Olfr370
AAACAAGCAGAGACACGGAAGCACAGCGGTCCTCTTTCCTGTCCTGTGGGATACACACT
TCAGAAGAGCATGAACTACTGCGGAGACAACGTCTGATGTACGACAGCTTGATCTGGAA
TTCTCGTTTCTTGGACAAACAGGAAGGAGGAGAAATGGCAAGTTTAGCAGGAGGGTCTG
GGAGCACATGCTGTACGGCTGTCAATCTCCTGTGCTTCTCGAGTGTGTAAGGATTTCCT
CAGTGCCCTCTCCCCATCTGGGGCTGATCTCTGATGACAGAGCCTCCATGGAGTGTGTA
AATGACACTGTGGTCAGAGAATTTGTCTTCCTTGGCTTCTCGTCTCTGGCTG
>8 WOM_Olfr370
GTACGGCTGTCAATCTCCTGTGCTTCTCGAGTGTGTAAGGATTTCCTCAGTGCCCTCTC
CCCATCTGGGGCTGATCTCTGATGACAGAGCCTCCATGGAGTGTGTAAATGACACTGTG
GTCAGAGAATTTGTCTTCCTTGGCTTCTCGTCTCTGGCTG
>9 WOM_Olfr370
TGGAGTGTGTAAATGACACTGTGGTCAGAGAATTCGTCTTCCTTGGCTTCTCGTCTCTG
GCTG
>10 WOM_Olfr370
GGTTTCAGTCTGGGTCTCAGACCCCAGACTATGATTCCATACCTCCCTGAGTAGACAGC
AGCAAACTTTAACTCACCTCGTACTTCCTCTTCTTCTAGCAGTCTATGTGACTTCAGTC
CTGGTGCCTCTGTGTCATTGGGCTTTCTCAGGAACAGAAAAGAAGGAAGAGGAGCTCGT
CCATGGACAGCTGACATCTTCCTTCAGCTGACACCACTAACACTGGGGAATAAGATTAA
GCATCTACCTCCAACAACACATAGGTTTTCATATCTCAGGAAGGAGGAGAAATGGCAAG
TTTAGCAGGAGGGTCTGGGAGCACATGCTGTATGGCTGTCAATCTCCTGTGCTTCTCGA
GTGTGTAAGGATTTCCTCAGTGCCCTCTCCCCATCTGGGGCTGATCTCTGATGACAGAG
CCTCCATGGAGTGTGTAAATGACACTGTGGTCAGAGAATTTGTCTTCCTTGGCTTCTCG
TCTCTGGCTG
>11 WOM_Olfr370
GGTTTCAGTCTGGGTCTCAGACCCCAGACTATGATTCCATACCTCCCTGAGTAGACAGC
AGCAAACTTTAACTCACCTCGTACTTCCTCTTCTTCTAGCAGTCTATGTGACTTCAGTC
CTGGTGCCTCTGTGTCACTGGGCTTTCTCAGGAACAGAAAAGAAGGAAGAGGAGCTCGT
CCATGGACAGCTGACATCTTCCTTCAGCTGACACCACTAACACTGGGGAATAAGATTAA
GCATCTACCTCCAACAACACATAGGTTTTCATATCTCAGAACTACTGCGGAGACAACGT
CTGATGTACGACAGCTTGATCTGGAATTCTCGTTTCTTGGACAAACAGGAAGGAGGAGA
AATGGCAAGTTTAGCAGGAGGGTCTGGGAGCACATGCTGTACGGCTGTCAATCTCCTGT
GCTTCTCGAGTGTGTAAGGATTTCCTCAGTGCCCTCTCCCCATCTGGGGCTGATCTCTG
ATGACAGAGCCTCCATGGAGTGTGTAAATGACACTGTGGTCAGAGAATTTGTCTTCCTT
GGCTTCTCGTCTCTGGCTG

>1 OSNs_Olfr370
GTTACCAGAACTACTGCGGAGACAACCTCTGATGTACGACAGCTTGATCTGCAATTCTC
GTTTCTTGGACAAACAGGAAGGAGGAGAAATGGCAAGTTTAGCAGGAAGGTCTGGGAGC
ACATGCTGTACGGCTGTCAGTCTCCTGTGCTTCTCGAGCGTGTAAGGATTTCCTCAGTG
CCCTCTCCCCATCTGGGGCTGATCTCTGATGACAGAGCCTCCATGGAGTGTGTAAATGA
CACTGTGGTCAGAGAGTTTGTCTTCCTTGGCTTCTCGTCTCTGGCTG
>2 OSNs_Olfr370

GTACGGCTGTCAGTCTCCTGTGCTTCTCGAGCGTGTAAGGATTTCCTCAGTGCCCTCTC
CCCATCTGGGGCTGATCTCTGATGACAGAGCCTCCATGGAGTGTGTAAATGACACTGTG
GTCAGAGAGTTTGTCTTCCTTGGCTTCTCGTCTCTGGCTG
>3 OSNs_Olfr370
GTACGGCTGTCAGTCTCCTGTGCTTCTCGAGCGTGTAAGGATTTCCTCAGTGCCCTCTC
CCCATCTGGGGCTGATCTCTGATGACAGAGCCTCCATGGAGTGTGTAAATGACACTGTG
GTCAGAGAGTTTGTCTTCCTTGGCTTCTCGTCTCTGGCTGAATCGAATTCCCGCGGCC
>4 OSNs_Olfr370
CAGGAAGGTCTGGGAGCACATGCTGTACGGCTGTCAGTCTCCTGTGCTTCTCGAGCGTG
TAAGGATTTCCTCAGTGCCCTCTCCCCATCTGGGGCTGATCTCTGATGACAGAGCCTCC
ATGGAGTGTGTAAATGACACTGTGGTCAGAGAGTTTGTCTTCCTTGGCTTCTCGTCTCT
GGCTGAATCGAATTCCCGCGGCC
>5 OSNs_Olfr370
CAGGAAGGTCTGGGAGCACATGCTGTACGGCTGTCAGTCTCCTGTGCTTCTCGAGCGTG
TAAGGATTTCCTCAGTGCCCTCTCCCCATCTGGGGCTGATCTCTGATGACAGAGCCTCC
ATGGAGTGTGTAAATGACACTGTGGTCAGAGAGTTTGTCTTCCTTGGCTTCTCGTCTCT
GGCTGAATCGAATTCCCGCGGCC
>6 OSNs_Olfr370
CAGGAAGGTCTGGGAGCACATGCTGTACGGCTGTCAGTCTCCTGTGCTTCTCGAGCGTG
TAAGGATTTCCTCAGTGCCCTCTCCCCATCTGGGGCTGATCTCTGATGACAGAGCCTCC
ATGGAGTGTGTAAATGACACTGTGGTCAGAGAGTTTGTCTTCCTTGGCTTCTCGTCTCT
GGCTGAATCGAATTCCCGCGGCC
>7 OSNs_Olfr370
GTACGGCTGTCAGTCTCCTGTGCTTCTCGAGCGTGTAAGGATTTCCTCAGTGCCCTCTC
CCCATCTGGGGCTGATCTCTGATGACAGAGCCTCCATGGAGTGTGTAAATGACACTGTG
GTCAGAGAGTTTGTCTTCCTTGGCTTCTCGTCTCTGGCTG
>8 OSNs_Olfr370
GTGTAAATGACACTGTGGTCAGAGAATTTGTCTTCCTTGGCTTCTCGTCTCTGGCTG
>9 OSNs_Olfr370
GTACGGCTGTCAGTCTCCTGTGCTTCTCGAGCGTGTAAGGATTTCCTCAGTGCCCTCTC
CCCATCTGGGGCTGATCTCTGATGACAGAGCCTCCATGGAGTGTGTAAATGACACTGTG
GTCAGAGAGTTTGTCTTCCTTGGCTTCTCGTCTCTGGCTGAATCGAATTCCCGCGGCC
>10 OSNs_Olfr370
CAGGAGGTCTGGGCACATACGTCCTCCTGCTCGAGCGTGTAAGGATTTCCTCAGCCCTC
TCCCCATCTGGGGCGATCTCTGATGACAGAGCCTCCATGGTGTAAATGACACTGTCAGA
GAGTTTGTCTTCCTTGGCTTCTCTTGGCTG
>11 OSNs_Olfr370
GTACGGCTGTCAATCTCCTGTGCTTCTCGAGTGTGTAAGGATTTCCTCAGTGCCCTCTC
CCCATCTGGGGCTGATCTCTGATGACAGAGCCTCCATGGAGTGTGTAAATGACACTGTG
GTCAGAGAATTTGTCTTCCTTGGCTTCTCGTCTCTGGCTG
>12 OSNs_Olfr370
GTACGGCTGTCAGTCTCCTGTGCTTCTCGAGCGTGTAAGGATTTCCTCAGTGCCCTCTC
CCCATCTGGGGCTGATCTCTGATGACAGAGCCTCCATGGAGTGTGTAAATGACACTGTG
GTCAGAGAGTTTGTCTTCCTTGGCTTCTCGTCTCAATCGAATTCCCGCGGCC
>13 OSNs_Olfr370

GGGAGCACATGCTGTACGGCTGTCAATCTCCTGTGCTTCTCGAGTGTGTAAGGATTTCC
TCAGTGCCCTCTCCCCATCTGGGGCTGATCTCTGATGACAGAGCCTCCATGGAGTGTGT
AAATGACACTGTGGTCAGAGAATTTGTCTTCCTTGGCTCGTCAATCGAACCCGCGGCC
>14 OSNs_Olfr370
GTACGGCTGTCAATCTCCTGTGCTTCTCGAGTGTGTAAGGATTTCCTCAGTGCCCTCTC
CCCATCTGGGGCTGATCTCTGATGACAGAGCCTCCATGGAGTGTGTAAATGACACTGTG
GTCAGAGAATTTGTCTTCCTTGGCTCTCGTCTCGAATCGAATTCCCGCGGCC
>15 OSNs_Olfr370
GCAGGAAGGTCTGGGAGCACATGCACGGGTCAGTCTCCTGTGCTTCTCGAGCGTGTAAG
GATTTCCTCAGTGCCCTCTCCCCATCTGGGGCTGATCTCTGATGACAGAGCCTCCATGG
TGTAAATGACACTGTGGTCAGAGAGTTTGTCTTCCTTGGCTTCTCGTCTCTGGCTG
>16 OSNs_Olfr370
CCTTCCGGCTTTGAGGGTAAGGATTTCCTCATGCCCTCTCCCCATGGGGATTGATGACA
GAGCCTGGTAAACGTGGTCAGATTTTC
>17 OSNs_Olfr370
CGGCTGTGCTGTGCTCTCGAGCGTGTAAGTTTCCTCAGTGCCCCCCCATCTGGGGCTGA
TCGATGAGAGCCAGTAAATGGTGGTCGAGTTTGTCTTCCTTGCTC
>18 OSNs_Olfr370
GTACGGCTGTCAATCTCCTGTGCTTCTCGAGTGTGTAAGGATTTCCTCAGTGCCCTCTC
CCCATCTGGGGCTGATCTCTGATGACAGAGCCTCCATGGAGTGTGTAAATGACACTGTG
GTCAGAGAATTTGTCTTCCTTGGCTTCTCGTCTCTGGCTG
>19 OSNs_Olfr370
CAGTCTCCTGTGCTTCTCGAGCGTGTAAGGATTTCCTCAGTGCCCTCTCCCCATCTGGG
GCTGATCTCTGATGACAGAGCCTCCATGGAGTGTGTAAATGACACTGTGGTCAGAGAGT
TTGTCTTCCTTGGCTTCTCGTCT

>1 WOM_Olfr371
GACTAACCCAAGCATACATGAAAAAACAATCCCCCTGCTCCAGAGAACATTGCTGTGGT
TCAGGAATCACTCTTTACCTCGTCTCACTGACCCTGCTGACTCATTAGCTCCACCAGTC
TCTTTTCTGAAAGACACTTCATTTACATGGAACCAGAAAACCACACAGGGATTCCAGAA
TTTTACCTGTTGGGACTTTCAGAGAATCCAGAGATTCAGTCCGTTCTCTTTGGGCTGTT
CTTG
>2 WOM_Olfr371
GTCACTGACCCTGCTGACTCATTAGCTCCACCAGTCTCTTTTCTGAAAGACCTGAACCT
CTCTCAGTCTTGGTGAGGAAAGCTTCTTTTTGCAGTGGGCAGCAGTCAGTGAGGACTCA
TGGCTGGTCAACATGCTGGGAATAACTGATTACACTTCATTTACATGGAACCAGAAAAC
CACACAGGGATTCCAGAATTTTACCTGTTGGGGCTTTCAGAGAATCCAGAGACTCAGTC
CGTTCTCTTTGGGCTGTTCTTG
>3 WOM_Olfr371
GAACATTGCTGTGGTTCAGGAATCACTCTTTACCTCGTCTCACTGACCCTGCTGACTCA
TTGGCTCCACCAGTCTCTTTTCTGAAAGACACTTCATTTACATGGAACCAGAAAACCAC
ACAGGGATTCCAGAATTTTACCTGTTGGGACTTTCAGAGAATCCAGAGATTCAGTCCGT
TCTCTTTGGGCTGTTCTTG
>4 WOM_Olfr371
ACCAAATAAACAACACAGCTCCTGACATTGTCATTCACTAACCCAAGCATACATGAAAA
AACAATCCCCCTGCTCCAGAGAACATTGCTGTGGTTCAGGAATCACTCTTTACCTCGTC
TCACTGACCCTGCTGACTCATTAGCTCCACCAGTCTCTTTTCTGAAAGACCTGAACCTC

TCTCAGTCTTGGTGAGGAAAGCTTCTTTTTGCAGTGGGCAGCAGTCAGTGAGGACTCAT
GGCTGGTCAACATGCTGGGAATAACTGATTACACTTCATTTACATGGAACCAGAAAACC
ACACAGGGATTCCAGAATTTTACCTGTTGGGACTTTCAGAGAATCCAGTGATTCAGTCC
GTTCTCTTTGGGCTGTTCTTG
>5 WOM_Olfr371
GAGAGAACATTGCTGTGGTTCAGGAATCACTCTTTACCTCGTCTCACTGACCCTGCTGA
CTCATTAGCTCCACCAGTCTCTTTTCTGAAAGACACTTCATTTACATGGAACCAGAAAA
CCACACAGGGATTCCAGAATTTTACCTGTTGGGACTTTCAGAGAATCCAGAGATTCAGT
CCGTTCTCTTTGGGCTGTTCTTG
>6 WOM_Olfr371
GATGGAACCAAATAAACAACACAGCTCCTGACATTGTCATTCACTAACCCAAGCATACA
TGAAAAAACAATCCCCCTGCTCCAGAGAACATTGCTGTGGTTCAGGAATCACTCTTTAC
CTCGTCTCACTGACCCTGCTGACTCATTAGCTCCACCAGTCTCTTTTCTGAAAGACACT
TCATTTACATGGAACCAGAAAACCACACAGGGATTCCAGAATTTTACCTGTTGGGACTT
TCAGAGAATCCAGAGATTCAGTCCGTTCTCTTTGGGCTGTTCTTG
>7 WOM_Olfr371
ATACATGAAAAAACAATCCCCCTGCTCCAGAGAACATTGCTGTGGTTCAGGAATCACTC
TTTACCTCGTCTCACTGACCCTGCTGACTCATTAGCTCCACCAGTCTCTTTTCTGAAAG
ACCTGAACCTCTCTCAGTCTTGGTGAGGAAAGCTTCTTTTTGCAGTGGGCAGCAGTCAG
TGAGGACTCATGGCTGGTCAACATGCTGGGAATAACTGATTGTGAGTGCTCAGCCCTAA
AGGAGACATCTTTATCACCTCTCGTGCTTTCAGGGCTCAGGGAACACTGTGTGAAGGAG
GCAGAGAGGATGTAAAGGACAGCAGACACTTCATTTACATGGAACCAGAAAACCACACA
GGGATTCCAGAATTTTACCTGTTGGGACTTTCAGAGAATCCAGAGATTCAGTCCGTTCT
CTTTGGGCTGTTCTTG
>8 WOM_Olfr371
GGAGAATTGAGTTCATGGAACCAAATAAACAACACAGCTCCTGACATTGTCATTCACTA
ACCCAAGCATACATGAAAAAACAATCCCCCTGCTCCAGAGAACATTGCTGTGGTTCAGG
AATCACTCTTTACCTCGTCTCACTGACCCTGCTGACTCATTAGCTCCACCAGTCTCTTT
TCTGAAAGACCTGAACCTCTCTCAGTCTTGGTGAGGAAAGCTTCTTTTTGCAGTGGGCA
GCAGTCAGTGAGGACTCATGGCTGGTCAACATGCTGGGAATAACTGATTACACTTCATT
TACATGGAACCAGAAAACCACACAGGGATTCCAGAATTTTACCTGTTGGGACTTTCAGA
GAATCCAGAGATTCAGTCCGTTCTCTTTGGGCTGTTCTTG
>9 WOM_Olfr371
GAATTGAGTTCATGGAACCAAATAAACAACACAGCTCCTGACATTGTCATTCACTAACC
CAAGCATACATGAAAAAACAATCCCCCTGNTCCAGANAACATTGCTNTGGTTCAGGAAT
CACTCTTTACCTCGTCTCACTGACCCTGCTGACTCATTAGCTCCACCAGTCTCTTTTCT
GAAAGACCTGAACCTCTCTCAGTCTTGGTGAGGAAAGCTTCTTTTTGCAGTGGGCAGCA
GTCAGTGAGGACTCNNGGCTGGTCAACATGCTGGGAATAACTGATTACACTTCATTTAC
ATGGAACCAGAAAACCACACAGGGATTCCAGAATTTTACCTGTTGGGACNTTCAGAGAA
TCCAGAGATTCAGTCCGTTCTCTTTGGGCTGTTCTTG
>10 WOM_Olfr371
GAGAATTGAGTTCATGGAACCAAATAAACAACACAGCTCCTGACATTGTCATTCACTAA
CCCAAGCATACATGAAAAAACAATCCCCCTGCTCCAGAGAACATTGCTGTGGTTCAGGA
ATCACTCTTTACCTCGTCTCACTGACCCTGCTGACTCATTAGCTCCACCAGTCTCTTTT
CTGAAAGACCTGAACCTCTCTCAGTCTTGGTGAGGAAAGCTTCTTTTTGCAGTGGGCAG
CAGTCAGTGAGGACTCATGGCTGGTCAACATGCTGGGAATAACTGATTACACTTCATTT

ACATGGAACCAGAAAACCACACAGGGATTCCAGAATTTTACCTGTTGGGACTTTCAGAG
AATCCAGAGATTCAGTCCGTTCTCTTTGGGCTGTTCTTG

>1 OSNs_Olfr371
GGAACATTGCTGTGGTTCAGGAATCACTCTTTACCTCGTCTCACTGACCCTGCTGACTC
ATTAGCTCCACCAGTCTCTTTTCTGAAAGACCTGAACCTCTCTCAGTCTTGGTGAGGAA
AGCTTCTTTTTGCAGTGGGCAGCAGTCAGTGAGGACTCATGGCTGGTCAACATGCTGGG
AATAACTGATTACACTTCATTTACATGGAACCAGAAAACCACACAGGGATTCCAGAATT
TTACCTGTTGGGACTTTCAGAGAATCCAGAGATTCAGTCCGTTCTCTTTGGGCTGTTCT
TG
>2 OSNs_Olfr371
GATGGAACCAAATAAACAACACAGCTCCTGACATTGTCATTCACTAACCCAAGCATACA
TGAAAAAACAATCCCCCTGCTCCAGAGAACATTGCTGTGGTTCAGGAATCACTCTTTAC
CTCGTCTCACTGACCCTGCTGACTCATTAGCTCCACCAGTCTCTTTTCTGAAAGACCTG
AACCTCTCTCAGTCTTGGTGAGGAAAGCTTCTTTTTGCAGTGGGCAGCAGTCAGTGAGG
ACTCATGGCTGGTCAACATGCTGGGAATAACTGATTACACTTCATTTACATGGAACCAG
AAAACCACACAGGGATTCCAGAATTTTACCTGTTGGGACTTTCAGAGAATCCAGAGATT
CAGTCCGTTCTCTTTGGGCTGTTCTTG
>3 OSNs_Olfr371
GATTGCTGTGGTTCAGGAATCACTCTTTACCTCGTCTCACTGACCCTGCTGACTCATTA
GCTCCACCAGTCTCTTTTCTGAAAGACACTTCATTTACATGGAACCAGAAAACCACACA
GGGATTCCAGAATTTTACCTGTTGGGACTTTCAGAGAATCCAGAGATTCAGTCCGTTCT
CTTTGGGCTGTTCTTG
>4 OSNs_Olfr371
GGTTCATGGAACCAAATAAACAACGCAGCTCCTGACATTGTCATTCACTAACCCAAGCA
TACATGAAAAAACAATCCCCCTGCTCCAGAAAACATTGCTGTGGTTCAGGAATCACTCT
TTACCTCGTCTCACTGACCCTGCTGACTCATTAGCTCCACCAGTCTCTTTTCTGAAAGA
CACTTCATTTACATGGAACCAGAAAACCACACAGGGATTCCAGAATTTTACCTGTTGGG
ACTTTCAGAGAATCCAGAGATTCAGTCCGTTCTCTTTGGGCTGTTCTTG
>5 OSNs_Olfr371
GATGGAACCAAATAAACAACACAGCTCCTGACATTGTCATTCACTAACCCAAGCATACA
TGAAAAAACAATCCCCCTGCTCCAGAGAACATTGCTGTGGTTCAGGAATCACTCTTTAC
CTCGTCTCACTGACCCTGCTGACTCATTAGCTCCACCAGTCTCTTTTCTGAAAGACCTG
AACCTCTCTCAGTCTTGGTGAGGAAAGCTTCTTTTTGCAGTGGGCAGCAGTCAGTGAGG
ACTCATGGCTGGTCAACATGCTGGGAATAACTGATTACACTTCATTTACATGGAACCAG
AAAACCACACAGGGATTCCAGAATTTTACCTGTTGGGACTTTCAGAGAATCCAGAGATT
CAGTCCGTTCTCTTTGGGCTGTTCTTG
>6 OSNs_Olfr371
GAACTTAGAAACCTCAGCCTCTAAAGAGAAGCAAGCACTAAGCCAACACATCATGTCAC
CAATGTGTGGAGTCTGAGTTAAGGCAGATGCACTCCGCTATATTTGTTTCCTCTTAGTT
CAGCCATCCTGTTTCTTCTCTTTTATAGACACTTCATTTACATGGAACCAGAAAACCAC
ACAGGGATTCCAGAATTTTACCTGTTGGGACTTTCAGAGAATCCAGAGATTCAGTCCGT
TCTCTTTGGGCTGTTCTTGAATCGAATTCCCGCGGCC
>7 OSNs_Olfr371
ATCTGAATACTACATATAAGTGCTATAGCTAGGAGTCCTTCACCCACAGGAGAGTATGG
CTGAACTTAGAAACCTCAGCCTCTAAAGAGAAGCAAGCACTAAGCCAACACATCATGTC
ACCAATGTGTGGAGTCTGAGTTAAGGCAGATGCACTCCGCTATATTTGTTTCCTCTTAG

TTCAGCCATCCTGTTTCTTCTCTTTTATAGACACTTCATTTACATGGAACCAGAAAACC
ACACAGGGATTCCAGAATTTTACCTGTTGGGACTTTCAGAGAATCCAGAGATTCAGTCC
GTTCTCTTTGGGCTGTTCTTG
>8 OSNs_Olfr371
AAGCATACATGAAAAAACAATCCCCCTGCTCCAGAGAACATTGCTGTGGTTCAGGAATC
ACTCTTTACCTCGTCTCACTGACCCTGCTGACTCATTAGCTCCACCAGTCTCTTTTCTG
AAAGACCTGAACCTCTCTCAGTCTTGGTGAGGAAAGCTTCTTTTTGCAGTGGGCAGCAG
TCAGTGAGGACTCATGGCTGGTCAACATGCTGGGAATAACTGATTACACTTCATTTACA
TGGAACCAGAAAACCACACAGGGATTCCAGAATTTTACCTGTTGGGACTTTCAGAGAAT
CCAGAGATTCAGTCCGTTCTCTTTGGGCTGTTCTTG
>9 OSNs_Olfr371
GGAATTGAGTTCATGGAACCAAATAAACAACACAGCTCCTGACATTGTCATTCACTAAC
CCAAGCATACATGAAAAAACAATCCCCCTGCTCCAGAGAGCGTTGCTGTGGTTCAGGAA
TCACTCTTTACCTCGTCTCACTGACCCTGCTGACTCATTAGCTCCACCAGTCTCTTTTC
TGAAAGACACTTCATTTACATGGAACCAGAAAACCACACAGGGATTCCAGAATTTTACC
TGTTGGGACTTTCGGAGAATCCAGAGACTCAGTCCGTTCTCTTTGGGCTGTTCTTG
>10 OSNs_Olfr371
ATCTGAATACTACATATAAGTGCTATAGCTAGGAGTCCTTCACCCACAGGAGAGTATGG
CTGAACTTAGAAACCTCAGCCTCTAAAGAGAAGCAAGCACTAAGCCAACACATCATGTC
ACCAATGTGTGGAGTCTGAGTTAAGGCAGATGCACTCCGCTATATTTGTTTCCTCTTAG
TTCAGCCATCCTGTTTCTTCTCTTTTATAGACACTTCATTTACATGGAACCAGAAAACC
ACACAGGGATTCCAGAATTTTACCTGTTGGGACTTTCAGAGAATCCAGAGATTCAGTCC
GTTCTCTTTGGGCTGTTCTTG

>1 WOM_Olfr466
GGGCTGTGGAAATTTTGTGTGTGCATAATTTACTGCAGAGTTGAAGAGGAACTCAAGGC
TCAGCTAGAAAGGGAAGGAAAGATACTACATATCTAGCTGTACTTATTCCAATTTTTCA
TGGCATATGGAACACAGTAGACCAATGTTATAAACAGAATGATTCTAATGCTATACAAA
AGTCTATAATTTAACTTAATCCTGTAGGTAGTAAAGTGTGAAATTTTTCTTCTTCTTGT
ATATCCTCTTTCTTTTCCATCCTCTCTGGTTATAAAATGACAAATTTTACACGTGTCTC
AGAGTTTATTCTACTTGGATTCAGAGGAGGTCCTGGTATACAAATGTTGCTATTTCTGA
TTTTTTTATTTTTGTATGTTATAGCAGTGGTGGGGAATTTTGGCATGA
>2 WOM_Olfr466
GGGGCTGTGGAAATTTTGTGTGTGCATAATTTACTGCAGAGTTGAAGAGGAACTCAAGG
CTCAGCTAGAAAGGGAAGGAAAGATACTACATATCTAGCTGTACTTATTCCAATTTTTC
ATGGCATATGGAACACGGTAGACCAATGTTATAAACAGAATGATTCTAATGCTATACAA
AAGTCTATAATTTAACTTAATCCTGTAGGTAGTAAAGTGTGAAATTTTTCTTCTTCTTG
TATATCCTCTTTCTTTTCCATCCTCTCTGGTTATAAAATGACAAATTTTACACGTGTCT
CAGAGTTTATTCTACTTGGATTCAGAGGAGGTCCTGGTATACAAATGTTGCTATTTCTG
ATTTTTTTATTTTTGTATGTTATAGCAGTGGTGGGGAATTTTGGCATGA
>3 WOM_Olfr466
GGGGCTGTGGAAATTTTGTGTGTGCATAATTTACTGCAGAGTTGAAGAGGAACTCAAGG
CTCAGCTAGAAAGGGAAGGAAAGATACTACATATCTAGCTGTACTTATTCCAATTTTTC
ATGGCATATGGAACACAGTAGACCAATGTTATAAACAGAGTGATTCTAATGCTATACAA
AAGTCTATAATTTAACTTAATCCTGTAGGTAGTAAAGTGTGAAATTTTTCTTCTTCTTG
TATATCCTCTTTCTTTTCCATCCTCTCTGGTTATAAAATGACAAATTTTACACGTGTCT

CAGAGTTTATTCTACTTGGATTCAGAGGAGGTCCTGGTATACAAATGTTGCTATTTCTG
ATTTTTTTATTTTTGTATGTTATAGCAGTGGTGGGGAATTTTGGCATGA
>4 WOM_Olfr466
GGGGAAGGCTCAGCTAGAAAGGGAAGGAAAGATACTACATATCTAGCTGTACTTATTCC
AATTTTTCATGGCATATGGAACACAGTAGACCAATGTTATAAACAGAATGATTCTAATG
CTATACAAAAGTCTATAATTTAACTTAATCCTGTAGGTAGTAAAGTGTGAAATTTTTCT
TCTTCTTGTATATCCTCTTTCTTTTCCATCCTCTCTGGTTATAAAATGACAAATTTTAC
ACGTGTCTCAGAGTTTATTCTACTTGGATTCAGAGGAGGTCCTGGTATACAAATGTTGC
TATTTCTGATTTTTTTATTTTTGTATGTTATAGCAGTGGTGGGGAATTTTGGCATGA
>5 WOM_Olfr466
GTGGGCTGTGGAAATTTTGTGTGTGCATAATTTACTGCAGAGTTGAAGAGGAACTCAAG
GCTCAGCTAGAAAGGGAAGGAAAGATACTACATATCTAGCTGTACTTATTCCAATTTTT
CATGGCATATGGAACACAGTAGACCAATGTTATAAACAGAATGATTCTAATGCTATACA
AAAGTCTATAATTTAACTTAATCCTGTAGGTAGTAAAGTGTGAAATTTTTCTTCTTCTT
GTATATCCTCTTTCTTTTCCATCCTCTCTGGTTATAAAATGACAAATTTTACACGTGTC
TCAGAGTTTATTCTACTTGGATTCAGAGGAGGTCCTGGTATACAAATGTTGCTATTTCT
GATTTTTTTATTTTTGTATGTTATAGCAGTGGTGGGGAATTTTGGCATGA
>6 WOM_Olfr466
GGGGCTGTGGAAATTTTGTGTGTGCATAATTTACTGCAGAGTTGAAGAGGAACTCAAGG
CTCAGCTAGAAAGGGAAGGAAAGATACTACATATCTAGCTGTACTTATTCCAATTTTTC
ATGGCATATGGAACACAGTAGACCAATGTTATAAACAGAATGATTCTAATGCTATACAA
AAGTCTATAATTTAACTTAATCCTGTAGGTAGTAAAGTGTGAAATTTTTCTTCTTCTTG
TATATCCTCTTTCTTTTCCATCCTCTCTGGTTATAAAATGACAAATTTTACACGTGTCT
CAGAGTTTATTCTACTTGGATTCAGAGGAGGTCCTGGTATACAAATGTTGCTATTTCTG
ATTTTTTTATTTTTGTATGTTATAGCAGTGGTGGGGAATTTTGGCATGA
>7 WOM_Olfr466
TGGGCTGTGGAAATTTTGTGTGTGCATAATTTACTGCAGAGTTGAAGAGGAACTCAAGG
CTCAGCTAGAAAGGGAAGGAAAGATACTACATATCTAGCTGTACTTATTCCAATTTTTC
ATGGCATATGGAGCACAGTAGACCAATGTTATAAACAGAATGATTCTAATGCTATACAA
AAGTCTATAATTTAACTTAATCCTGTAGGTAGTAAAGTGTGAAATTTTTCTTCTTCTTG
TATATCCTCTTTCTTTTCCATCCTCTCTGGTTATAAAATGACAAATTTTACACGTGTCT
CAGAGTTTATTCTACTTGGATTCAGAGGAGGTCCTGGTATACAAATGTTGCTATTTCTG
ATTTTTTTATTTTTGTATGTTATAGCAGTGGTGGGGAATTTTGGCATGA
>8 WOM_Olfr466
GGGGCTGTGGAAATTTTGTGTGTGCATAATTTACTGCAGAGTTGAAGAGGAACTCAAGG
CTCAGCTAGAAAGGGAAGGAAAGATACTACATATCTAGCTGTACTTATTCCAATTTTTC
ATGGCATATGGAACACAGTAGACCAATGTTATAAACAGAATGATTCTAATGCTATACAA
AAGTCTATAATTTAACTTAATCCTGTAGGTAGTAAAGTGTGAAATTTTTCTTCTTCTTG
TATATCCTCTTTCTTTTCCATCCTCTCTGGTTATAAAATGACAAATTTTACACGTGTCT
CAGAGTTTATTCTACTTGGATTCAGAGGAGGTCCTGGTATACAAATGTTGCTATTTCTG
ATTTTTTTATTTTTGTATGTTATAGCAGTGGTGGGGAATTTTGGCATGA
>9 WOM_Olfr466
GGGGCTGTGGAAATTTTGTGTGTGCATAATTTACTGCAGAGTTGAAGAGGAACTCAAGG
CTCAGCTAGAAAGGGAAGGAAAGGTACTACATATCTAGCTGTACTTATTCCAATTTTTC
ATGGCATATGGAACACAGTAGACCAATGTTATAAACAGAATGATTCTACTGCTATACAA
AAGTCTATAATTTAACTTAATCCTGTAGGTAGTAAAGTGTGAAATTTTTCTTCTTCTTG
TATATCCTCTTTCTTTTCCATCCTCTCTGGTTATAAAATGACAAATTTTACACGTGTCT

CAGAGTTTATTCTACTTGGATTCAGAGGAGGTCCTGGTATACAAATGTTGCTATTTCTG
ATTTTTTTATTTTTGTATGTTATAGCAGTGGTGGGGAATTTTGGCATGA
>10 WOM_Olfr466
GGGGCTGTGGAAATTTTGTGTGTGCATAATTTACTGCAGAGTTGAAGAGGAACTCAAGG
CTCAGCTAGAAAGGGAAGGAAAGATACTACATATCTAGCTGTACTTATTCCAATTTTTC
ATGGCATATGGAACACAGTAGACCAATGTTATAAACAGAATGATTCTAATGCTATACAA
AAGTCTATAATTTAACTTAATCCTGTAGGTAGTAAAGTGTGAAATTTTTCTTCTTCTTG
TATATCCTCTTTCTTTTCCATCCTCTCTGGTTATAAAATGACAAATTTTACACGTGTCT
CAGAGTTTATTCTACTTGGATTCAGAGGAGGTCCTGGTATACAAATGTTGCTATTTCTG
ATTTTTTTATTTTTGTATGTTATAGCAGTGGTGGGGAATTTTGGCATGA
>11 WOM_Olfr466
GGGGCTGTGGAAATTTTGTGTGTGCATAATTTACTGCAGAGTTGAAGAGGAACTCAAGG
CTCAGCTAGAAAGGGAAGGAAAGATACTACATATCTAGCTGTACTTATTCCAATTTTTC
ATGGCATATGGAACACAGTAGACCAATGTTATAAACAGAATGATTCTAATGCTATACAA
AAGTCTATAATTTAACTTAATCCTGTAGGTAGTAAAGTGTGAAATTTTTCTTCTTCTTG
TATATCCTCTTTCTTTTCCATCCTCTCTGGTTATAAAATGACAAATTTTACACGTGTCT
CAGAGTTTATTCTACTTGGATTCAGAGGAGGTCCTGGTATACAAATGTTGCTATTTCTG
ATTTTTTTATTTTTGTATGTTATAGCAGTGGTGGGGAATTTTGGCATGA
>12 WOM_Olfr466
TGGGCTGTGGAAATTTTGTGTGTGCATAATTTACTGCAGAGTTGAAGAGGAACTCAAGG
CTCAGCTAGAAAGGGAAGGAAAGATACTACATATCTAGCTGTACTTATTCCAATTTTTC
ATGGCATATGGAACACAGTAGACCAATGTTATAAACAGAATGATTCTAATGCTATACAA
AAGTCTATAATTTAACTTAATCCTGTAGGTAGTAAAGTGTGAAATTTTTCTTCTTCTTG
TATATCCTCTTTCTTTTCCATCCTCTCTGGTTATAAAATGACAAATTTTACACGTGTCT
CAGAGTTTATTCTACTTGGATTCAGAGGAGGTCCTGGTATACAAATGTTGCTATTTCTG
ATTTTTTTATTTTTGTATGTTATAGCAGTGGTGGGGAATTTTGGCATGA
>13 WOM_Olfr466
GGGGCTGTGGAAATTTTGTGTGTGCATAATTTACTGCAGAGTTGAAGAGGAACTCAAGG
CTCAGCTAGAAAGGGAAGGAAAGATACTACATATCTAGGTGAGGTCTATCTATTTTATA
GAAAATATAACGTTTTCTCAAAGACACTGTGACCAATTATATTAAGTATATTTGAATTT
TAGTTAAATAGATGAAGATTCTGGATCAATCATACTTTTCTGAGAATAACAATTGATTC
ATGAACATAACAGGCATCAATCCAAATTCATTGGAGAAGCAATGTACCCTAAACTATTA
ATAACTATAAAACTGAGATTGTGCTTACTATATGCCAGGAATCTGCACAAAACTTTCAC
CTATAAAATGCTTTAGACTTTACAGTGAACCATCACTTTTAATATCCACACCCAACAGG
TTAAGCTACCAGGAAAGTGGTCATATTAAGGCCAGGGTTTTTGACACCAGGGCCTTATT
ATACCAGATATTAGTACACTGAACTCTTTCTCTGTTTCATATCACTACTTTGAATATTA
GAGATTCCTAGTAATAAGCAAATTGGAAAACAATAGCCAAAATTTTTCAAAATTGTAAA
TACCCCATGCATACAAATACCTACCTCATAAATGCACAGCTTCATCACTTATTGTTTTA
AAATTTGGTATCTCACTATACAACAAAATCTCAATTTACTGAAGTACAAGTGTTCCTTT
AGAGAGGATACTAAATTANATTAAGTTTTATGTGGTATTTGTATAATAGCTCATGAAGA
AGTGTGTATCAGAGATAGAANAAGTAAGAGAANAATAATTTAACAAGAGAGCACATAAA
TTACAATAAAAGATCTAATTTTATAAGAAGAATATATTACTTGATTTATTTTGTCTTAT
AGCTTAACAAAAAATTCATTAGTTTGTCTAGAACGTATCCAATAAATTTTTT
>14 WOM_Olfr466
GGGGCTGTGGAAATTTTGTGTGTGCATAATTTACTGCAGAGTTGAAGAGGAATTCAAGG
CTCAGCTAGAAAGGGAAGGAAAGATACTACATATCTAGCTGTACTTATTCCAATTTTTC
ATGGCATATGGAACACAGTAGACCAATGTTATAAACAGAATGATTCTAATGCTATACAA

AAGTCTATAATTTAACTTAATCCTGTAGGTAGTAAAGTGTGAAATTTTTCTTCTTCTTG
TATATCCTCTTTCTTTTCCATCCTCTCTGGTTATAAAATGACAAATTTTACACGTGTCT
CAGAGTTTATTCTACTTGGATTCAGAGGAGGTCCTGGTATGCAAATGTTGCTATTTCTG
ATTTTTTTATTTTTGTATGTTATAGCAGTGGTGGGGAATTTTGGCATGA

>1 OSNs_Olfr466
AGCAGTGGTGGGGAATTTTGGCATGA
>2 OSNs_Olfr466
GGGGCTGTGGAAATTTTGTGTGTGCATAATTTACTGCAGAGTTGAAGAGGAACTCAAGG
CTCAGCTAGAAAGGGAAGGAAAGATACTACATATCTAGCTGTACTTATTCCAATTTTTC
ATGGCATATGGAACACAGTAGACCAATGTTATAAACAGAATGATTCTAATGCTATACAA
AAGTCTATAATTTAACTTAATCCTGTAGGTAGTAAAGTGTGAAATTTTTCTTCTTCTTG
TATATCCTCTTTCTTTTCCATCCTCTCTGGTTATAAAATGACAAATTTTACACGTGTCT
CAGAGTTTATTCTACTTGGATTCAGAGGAGGTCCTGGTATACAAATGTTGCTATTTCTG
ATTTTTTTATTTTTGTATGTTATAGCAGTGGTGGGGAATTTTGGCATGA
>3 OSNs_Olfr466
AGCAGTGGTGGGGAATTTTGGCATGA
>4 OSNs_Olfr466
GGGGCTGTGGAAATTTTGTGTGTGCATAATTTACTGCAGAGTTGAAGAGGAACTCAAGG
CTCAGCTAGAAAGGGAAGGAAAGATACTACATATCTAGCTGTACTTATTCCAATTTTTC
ATGGCATATGGAACACAGTAGACCAATGTTATAAACAGAATGATTCTAATGCTATACAA
AAGTCTATAATTTAACTTAATCCTGTAGGTAGTAAAGTGTGAAATTTTTCTTCTTCTTG
TATATCCTCTTTCTTTTCCATCCTCTCTGGTTATAAAATGACAAATTTTACACGTGTCT
CAGAGTTTATTCTACTTGGATTCAGAGGAGGTCCTGGTATACAAATGTTGCTATTTCTG
ATTTTTTTATTTTTGTATGTTATAGCAGTGGTGGGGAATTTTGGCATGA
>5 OSNs_Olfr466
AAGCAGTGGTGGGGAATTTTGGCATGA
>6 OSNs_Olfr466
AAGCAGTGGTGGGGAATTTTGGCATGA
>7 OSNs_Olfr466
AAGCAGTGGTGGGGAATTTTGGCATGA
>8 OSNs_Olfr466
AAGCAGTGGTGGGGAATTTTGGCATGA
>9 OSNs_Olfr466
AAGCAGTGGTGGGGAATTTTGGCATGA
>10 OSNs_Olfr466
AAGCAGTGGTGGGGAATTTTGGCATGA
>11 OSNs_Olfr466
GCAGTGGTGGGGAATTTTGGCATGA
>12 OSNs_Olfr466
GGGCTGTGGAAATTTTGTGTGTGCATAATTTACTGCAGAGTTGAAGAGGAACTCAAGGC
TCAGCTAGAAAGGGAAGGAAAGATACTACATATCTAGCTGTACTTATTCCAATTTTTCA
TGGCATATGGAACACAGTAGACCAATGTTATAAACAGAATGATTCTAATGCTATACAAA
AGTCTATAATTTAACTTAATCCTGTAGGTAGTAAAGTGTGAAATTTTTCTTCTTCTTGT
ATATCCTCTTTCTTTTCCATCCTCTCTGGTTATAAAATGACAAATTTTACACGTGTCTC
AGAGTTTATTCTACTTGGATTCAGAGGAGGTCCTGGTATACAAATGTTGCTATTTCTGA
TTTTTTTATTTTTGTATGTTATAGCAGTGGTGGGGAATTTTGGCATGA

```
>13 OSNs_Olfr466
AAGCAGTGGTGGGGAATTTTGGCATGA
>14 OSNs_Olfr466
AAGCAGTGGTGGGGAATTTTGGCATGA
>15 OSNs_Olfr466
AAGCAGTGGTGGGGAATTTTGGCATGA
>16 OSNs_Olfr466
GGGGCTGTGGAAATTTTGTGTGTGCATAATTTACTGCAGAGTTGAAGAGGAACTCAAGG
CTCAGCTAGAAAGGGAAGGAAAGATACTACATATCTAGCTGTACTTATTCCAATTTTTC
ATGGCATATGGAACACAGTAGACCAATGTTATAAACAGAATGATTCTAATGCTATACAA
AAGTCTATAATTTAACTTAATCCTGTAGGTAGTAAAGTGTGAAATTTTTCTTCTTCTTG
TATATCCTCTTTCTTTTCCATCCTCTCTGGTTATAAAATGACAAATTTTACACGTGTCT
CAGAGTTTATTCTACTTGGATTCAGAGGAGGTCCTGGTATACAAATGTTGCTATTTCTG
ATTTTTTTATTTTTGTATGTTATAGCAGTGGTGGGGAATTTTGGCATGA
>17 OSNs_Olfr466
GGGGCTGTGGAAATTTTGTGTAACTGCGTTGAAGAGGAACTAGGCTCAGAGAAAGGGAA
GGAAAGATACTACATATCTAGCTACTTATTCCAATTTTTATGGCATATGGACACAGTAG
ACCAATAACAGAATGATTCTAATGCTATACAAAAGTCTATAATTTAACTTAATCCTGTA
GGTAGTAAAGAAATTTTTCTTCTTCTTGTATATCCTCTTTCTTTTCCATCCTCTCTGGT
TATAAAATGACAAATTTTACACGTGTCTCAGAGTTTATTCTACTTGGATTCAGAGGAGG
TCCTGGTATACAAATGTTGCTATTTCTGATTTTTTTATTTTTGTATGTTATAGCAGTGG
TGGGGAATTTTGGCATGA

>1 WOM_Olfr1402
CCCTGTAGGTTGGGGTGGTTGGAAAATCCCAGGAAGATGAATTCTGTGACACGTGTGTG
ATTGGTCATTTCAAATGGAGTCATCGCACTGACATCTGTTGGAAGCCCTGTGTCGCTGG
CTTTGGTGTGAGTGGGGATCAGTGTCCTGCACAGCACTGGACATTGGTAGAATTAGCTG
TTGACCTTCATGAATCATGATGACCTGCCATCCTGAGAGCTGCCTCTTACACACTCAAT
ACTTCACAGAGGAGTCAAATCCTGTGGTTTCTCCATAGTTCACTTTTCTGGGATGACAA
TTATAAAGGTTCATGGACTAACCTTTAGAGAGTAGCCTAAGTAATCTCTCTCTCAGCAG
CTGTCTGAAACGCCTTAGCACACAAATACAAATCC
>2 WOM_Olfr1402
CCCTGTAGGTTGGGGTGGTTGGAAAATCCCAGGAAGATGAATTCTGTGACACGTGTGTG
ATTGGTCATTTCAAATGGAGTCATTGCTTTAGAGAGTAGCCTAAGTAATCTCTCTCTCA
GCAGCTGTCTGAAACGCCTTAGCACACAAATACAAATCC
>3 WOM_Olfr1402
CCCTGTAGGTTGGGGTGGTTGGAAAATCCCAGGAAGATGAATTCTGTGACACGTGTGTG
ATTGGTCATTTCAAATGGAGTCATTGCACTGACATCTGTTGGAAGCCCTGTGTCGCTGG
CTTTGGTGTGAGTGGGGATCAGTGTCCTGCACAGCACTGGACATTGGTAGAATTAGCTG
TTGACCTTCATGAATCATGATGACCTGCCATCCTGAGAGCTGCCTCTTACACACTCAAT
ACTTCACAGAGGAGTCAAATCCTGTGGTTTCT
>4 WOM_Olfr1402
CCCTGTAGGTTGGGGTGGTTGGAAAATCCCAGGAAGATGAATTCTGTGACACGTGTGTG
ATTGGTCATTTCAAATGGAGTCATTGCTTTAGAGAGTAGCCTAAGTAATCTCTCTCTCA
GCAGCTGTCTGAAACGCCTTAGCACACAAATACAAATCC
>5 WOM_Olfr1402
```

CCCTGTAGGTTGGGGTGGTTGGAAAATCCCAGGAAGATGAATTCTGTGACACGTGTGTG
ATTGGTCATTTCAAATGGAGTCATTGCACTGACATCTGTTGGAAGCCCTGTGTCGCTGG
CTTTGGTGTGAGTGGGGATCAGTGTCCTGCACAGCACTGGACATTGGTAGAATTAGCTG
TTGACCTTCATGAATCATGATGACCTGCCATCCTGAGAGCTGCCTCTTACACACTCAAT
ACTTCACAGAGGAGTCAAATCCTGTGGTTTCTCCATAGTTCACTTTTCTGGGATGACAA
TTATAAAGGTTCATGGACTAACCTTTAGAGAGTAGCCTAAGTAATCTCTCTCTCAGCAG
CTGTCTGAAACGCCTTAGCACACAAATACAAATCAAAGGCAACAGCCAAAACAGAAGCT
GTAGAAATGAAGAACAATTAACTCTCT
>6 WOM_Olfr1402
CCCTGTAGGTTGGGGTGGTTGGAAAATCCCAGGAAGATGAATTCTGTGACACGTGTGTG
ATTGGTCATTTCAAATGGAGCCATTGCTTTAGAGAGTAGCCTAAGTAATCTCTCTCTCA
GCC
>7 WOM_Olfr1402
CCCTGTAGGTTGGGGTGGTTGGAAAATCCCAGGAAGATGAATTCTGTGACACGTGTGTG
ATTGGTCATTTCAAATGGAGTCATTGCTTTAGAGAGTAGCCTAAGTAATCTCTCTCTCA
GCAGCTGTCTGAAACGCCTTAGCACACAAATACAAATCC
>8 WOM_Olfr1402
CCCTGTAGGTTGGGGTGGTTGGAAAATCCCAGGAAGATGAATTCTGTGACACGTGTGTG
ATTGGTCATTTCAAATGGAGTCATTGCACTGACATCTGTTGGAAGCCCTGTGTCGCTGG
CTTTGGTGTGAGTGGGGATCAGTGTCCTGCACAGCACTGGACATTGGTAGAATTAGCTG
TTGACCTTCATGAATCATGATGACCTGCCATCCTGAGAGCTGCCTCTTACACACTCAAT
ACTTCACAGAGGAGTCAAATCCTGTGGTTTCTCCATAGTTCACTTTTCTGGGATGACAA
TTATAAAGGTTCATGGACTAACCTTTAGAGAGTAGCCTAAGTAATCTCTCTCTCAGCAG
CTGTCTGAAACGCCTTAGCACACAAATACAAATCAAAGGCAACAGCCAAAACAGAAGCT
GTAGAAATC
>9 WOM_Olfr1402
CCCTGTAGGTTGGGGTGGTTGGAAAATCCCAGGAAGATGAATTCTGTGACACGTGTGTG
ATTGGTCATTTCAAATGGAGTCATTGCTTTAGAGAGTAACCTAAGTAATCTCTCTCTCA
GCAGCTGTCTGAAACGCCTTAGCACACAAATACAAATCAAAGGCCCCCATGTACTCTGC
GTTGATACCAC
>10 WOM_Olfr1402
CCCTGTAGGTTGGGGTGGTTGGAAAATCCCAGGAAGATGAATTCTGTGACACGTGTGTG
ATTGGTCATTTCAAATGGAGCCATTGCTTTAGAGAGTAGCCTAAGTAATCTCTCTCTCA
GCC
>11 WOM_Olfr1402
CCCTGTAGGTTGGGGTGGTTGGAAAATCCCAGGAAGATGAATTCTGTGACACGTGTGTG
ATTGGTCATTTCAAATGGAGTCATTGCACTGACATCTGTTGGAAGCCCTGTGTCGCTGG
CTTTGGTGTGAGTGGGGATCAGTGTCCTGCACAGCACTGGACATTGGTAGAATTAGCTG
TTGACCTTCATGAATCATGATGACCTGCCATCCTGAGAGCTGCCTCTTACACACTCAAT
ACTTCACAGAGGAGTCAAATCCTGTGGTTTCTCCATAGTTCACTTTTCTGGGATGACAA
TTATAAAGGTTCATGGACTAACCTTTAGAGAGTAGCCTAAGTAATCTCTCTCTCAGCAG
CTGTCTGAAACGCCTTAGCACACAAATACAAATC
>12 WOM_Olfr1402
CCCTGTAGGTTGGGGTGGTTGGAAAATCACAGGAAGATGAATTCTGTGACACGTGTGTG
ATTGGTCATTTCAAATGGAGTCATTGCTTTAGAGAGTAGCCTAAGTAATCTCTCTCTCA
GCAGCTGTCTGAAACGCCTTAGCACACAAATACAAATCC

>1 OSNs_Olfr1402
CCCTGTAGGTTGGGGTGGTTGGAAAATCCCAGGAAGATGAATTCTGTGACACGTGTGTG
ATTGGTCATTTCAAATGGAGTCATTGCACTGACATCTGTTGGAAGCCCTGTGTCGCTGG
CTTTGGTGTGAGTGGGGATCAGTGTCCTGCACAGCACTGGACATTGGTAGAATTAGCTG
TTGACCTTCATGAATCATGATGACCTGCCATCCTGAGAGCTGCCTCTTACACACTCAAT
ACTTCACAGAGGAGTCAAATCCTGTGGTTTCTCCATAGTTCACTTTTCTGGGATGACAA
TTATAAAGGTTCATGGACTAACCTTTAGAGAGTAGCCTAAGTAATCTCTCTCTCAGCAG
CTGTCTGAAACGCCTTAGCACACAAATACAAATCC
>2 OSNs_Olfr1402
CCCTGTAGGTTGGGGTGGTTGGAAAATCCCAGGAAGATGAATTCTGTGACACGTGTGTG
ATTGGTCATTTCAAATGGAGTCATTGCTACAGAGAACAAAGGATCGAGAGTAAATATGT
ACAGAAGATACTGACAAACATGGTTCTGAATTACCAGGGTCATGTCCAGTAAAGAGTCA
TAAAGGTATCTGGATAGTGGTCACCTGCTGGGCTAGCCTTCATATGATCAGTATTTTAG
GCCAAGCCAACTTTGGACCCCAGGATTTTCCACAAACCTGCATGGCTGATAAGGGGTGA
GAATAAATGGAAACACAGTCACAGCCAACACTAAGCCCTCAATGCTTACATTACTATAA
ACTTGTTGTTTCCTCGTCTCAATGCTATCTCTCTCTTATTATCCTCCTTTTTTGAAAAC
ATTTAATTTTTCTCTAAAAGACATAAGTTTTCATTAAAAAAAAAAGCAGAAAGAAAATCA
AATGAATTCAGTCACCTGCAGATCATATCAGTACTACCTCTCAGAGCATTGCACTTTAG
GGCATTTTCTCTCCCATTGTGCACTTAAGGGGTGTGTGTGTGCATGTACACACATGTTC
ATGGGTAGGTGTAATGTGCACATATTTGTATATGTACATGGAAACCACAGGTCAATCTT
TGGTGTAGTTTCTTTGGAACTATTCACCTTATTTTTTTGAGGCAGGGTCTTTCACTGGG
ACCTTAGGGTGGCCAAATAGGCTATACTGGCTCTCCTATGAGCTCCAAGGAGCCTTCTG
TGTCTGCTTACCCAGTACTGGGATTACAAGTGTTTGCCAACAATACCTG
>3 OSNs_Olfr1402
CCCTGTAGGTTGGGGTGGTTGGAAAATCCCAGGAAGATGAATTCTGTGACACGTGTGTG
ATTGGTCATTTCAAATGGAGTCATTGCACTGACATCTGTTGGAAGCCCTGTGTCGCTGG
CTTTGGTGTGAGTGGGGATCAGTGTCCTGCACAGCACTGGACATTGGTAGAATTAGCTG
TTGACCTTCATGAATCATGATGACCTGCCATCCTGAGAGCTGCCTCTTACACACTCAAT
ACTTCACAGAGGAGTCAAATCCTGTGGTTTCTCCATC
>4 OSNs_Olfr1402
CCCTGTAGGTTGGGGTGGTTGGAAAATCCCAGGAAGATGAATTCTGTGACACGTGTGTG
ATTGGTCATTTCAAATGGAGTCATTGCTACAGAGAACAAAGGATCGAGAGTAAATATGT
ACAGAAGATACTGACAAACATGGTTCTGAATTACCAGGGTCATGTCCAGTAAAGAGTCA
TAAAGGTATCTGGATAGTGGTCACCTGCTGGGCTAGCCTTCATATGATCAGTATTTTAG
GCCAAGCCAACTTTGGACCCCAGGATTTTCCACAAACCTGCATGGCTGATAAGGGGTGA
GAATAAATGGGAACACAGTCACAGCCAACACTAAGCCCTCAATGCTTACATTACTATAA
ACTTGTTGTTTCCTCGTCTCAATGCTATCTCTCTCTTATTATCCTCCTTTTTTGAAAAC
ATTTAATTTTTCTCTAAAAGACGTAAGTTTTCATTAAAAAAAAAAGCAGAAAGAAAATCA
AATGAATTCAGTCACCTGCAGATCATATCAGTACTACCTCTCAGAGCATTGCACTTTAG
GGCATTTTCTCT
>5 OSNs_Olfr1402
CCCTGTAGGTTGGGGTGGTTGGAAAATCCCAGGAAGATGAATTCTGTGACACGTGTGTG
ATTGGTCATTTCAAATGGAGTCATTGCACTGACATCTGTTGGAAGCCCTGTGTCGCTGG
CTTTGGTGTGAGTGGGGATCAGTGTCCTGCACAGCACTGGACATTGGTAGAATTAGCTG
TTGACCTTCATGAATCATGATGACCTGCCATCCTGAGAGCTGCCTCTTACACACTCAAT
ACTTCACAGAGGAGTCAAATCCTGTGGTTTCTCC
>6 OSNs_Olfr1402

CCCTGTAGGTTGGGGTGGTTGGAAAATCCCGGGAAGATGAATTCTGTGACACGTGTGTG
ATTGGTCATTTCAAATGGAGTCATTGCTTTAGAGAGTAGCCTAAGTAATCTCTCTCTCA
GCAGCTGTCTGAAACGCCTTAGCACACAAATACC
>7 OSNs_Olfr1402
CACTAGTGATTCACTAAGCCCTCAATGCTTACATTACTATAAACTTGTTGTTTCCTCGT
CTCAATGCTATCTCTCTCTTATTATCCTCCTTTTTTGAAAACATTTAATTTTTCTCTAA
AAGACATAAGTTTTCATTAAAAAAAAAGCAGAAAGAAAATCAAATGAATTCAGTCACCT
GCAGATCATATCAGTACTACC
>8 OSNs_Olfr1402
CCCTGTAGGTTGGGGTGGTTGGAAAATCCCAGGAAGATGAATTCTGTGACACGTGTGTG
ATTGGTCATTTCAAATGGAGTCATTGCACTGACATCTGTTGGAAGCCCTGTGTCGCTGG
CTTTGGTGTGAGTGGGGATCAGTGTCCTGCACAGCACTGGACATTGGTAGAATTAGCTG
TTGACCTTCATGAATCATGATGACCTGCCATCCTGAGAGCTGCCTCTTACACACTCAAT
ACTTCACAGAGGAGTCAAATCCTGTGGTTTCTCCATAGTTCACTTTTCTGGGATGACAA
TTATAAAGGTTCATGGACTAACCTTTAGAGAGTAGCCTAAGTAATCTCTCTCTCAGCAG
CTGTCTGAAACGCCTTAGCACACAAATACAAATCAAAGGCAACAGCCAAAACC
>9 OSNs_Olfr1402
CCCTGTAGGTTGGGGTGGTTGGAAAATCCCAGGAAGATGAATTCTGTGACACGCGTGTG
ATTGGTCATTTCAAATGGAGTCATTGCTTTAGAGAGTAGCCTAGGTAATCTCTCTCTCA
GCAGCTGTCTGAAACGCCTTAGCACACAAATACAAATCAAAGGCAACAGCCAAAACAGA
AGCTGTAGAAAT
