# Supplementary material for: The Mouse Solitary Odorant Receptor Gene Promoters as Models for the Study of Odorant Receptor Gene Choice
Source: PLoS One. 2016 Jan 21;11(1):e0144698. doi: 10.1371/journal.pone.0144698 (PMC4721658; doi:10.1371/journal.pone.0144698)
Supplement: S2 File — FASTA entries are provided for candidate promoters of all solitary genes plus Olfr6 (M50); in addition to the standard C57BL/6J version, allelic variants are also provided for wild populations of Mus musculus domesticus (France, Germany and Iran), for their consensus sequence (described as Wild_consensus) and for 36 additional laboratory strains. Sequences are invariably presented on genomic plus strand. (PDF) [file pone.0144698.s004.pdf]

Putative promoters variants in FASTA format

```
>Olfr6 C57BL/6J
TCTGCATACTATTTCAAATAATCTTAATACATTAAGGAGAGATAGTGACTTTATCAC
AGAGAGGAAGGAAAAATGAAGGCTTGGGACCTACCCAGGTGAGGCAGTGGGAGGAGT
CAACACATAAAGCAACTAGGGAGACCAGGGATTGTTGGAAGAGAAATAGGTCAGGAG
CAAAAGTAAGAGAAAGAGTGAGAGAAGACTGAGAAAGGCAATGAACAGTGATGGAGA
AAAAGCACAACAATAGGAAAACCTCTTCTATCCTGGCCTCTAGGAGTATCAGGCAAG
CCCAATAGTAAGAACA
>Olfr6 Wild_consensus
TCTGCATACTATTTCAAATAATCTTAATACATTAAGGAGAGATAGTGACTTTATCAC
AGAGAGGAAGGAAAAATGAAGGCTTGGGACCTACCCAGGTGAGGCAGTGGGAGGAGT
CAACACATAAAGCAACTAGGGAGACCAGGGATTGTTGGAAGAGAAATAGGTCAGGAG
CAAAAGTAAGAGAAAGAGTGAGAGAAGACTGAGAAAGGCAATGAACAGTGATGGAGA
AAAAGCACAACAATAGGAAAACCTCTTCTATCCTGGCCTCTAGGAGTATCAGGCAAG
CCCAATAGTAAGAACA
>Olfr6 France
TCTGCATCCTATTTCAAATAATATTAATACATTAAGGAGAGATAGTGACTTTATCAC
AGAGAGGAAGGAAAAATGAAGGCTTGGGACCTACCCAGGTGAGACAGTGGGAGGAGT
CAACACATAAAGCAACTAGGGAGACCAGGGATTGTTGGAAGAGAAATAGGTCAGGAG
CAAGAGTAAGAGAAAGAGTGAGAGAAGACTGAGAAAGGCAATGAACAGTGATGGAGA
AAAAGCACAACAATAGGAAAACCTCTTCTATCCTGGCCTCTAGGAGTATCAGGCAAG
CCCAATAGTAAGAACA
>Olfr6 Germany
TCTGCATACTATTTCAAATAATCTTAATACATTAAGGAGAGATAGTGACTTTATCAC
AGAGAGGAAGGAAAAATGAAGGCTTGGGACCTACCCAGGTGAGGCAGTGGGAGGAGT
CAACACATAAAGCAACTAGGGAGACCAGGGATTGTTGGAAGAGAAATAGGTCAGGAG
CAAAAGTAAGAGAAAGAGTGAGAGAAGACTGAGAAAGGCAATGAACAGTGATGGAGA
AAAAGCACAACAATAGGAAAACCTCTTCTATCCTGGCCTCTAGGAGTATCAGGCAAG
CCCAATAGTAAGAACA
>Olfr6 Iran
TCTGCATACTATTTCAAATAATCTTAATACATTAAGGAGAGATAGTGACTTTATCAC
AGAGAGGAAGGAAAAATGAAGGCTTGGGACCTACCCAGGTGAGGCAGTGGGAGGAGT
CAACACATAAAGCAACTAGGGAGACCAGGGATTGTTGGAAGAGAAATAGGTCAGGAG
CAAAAGTAAGAGAAAGAGTGAGAGAAGACTGAGAAAGGCAATGAACAGTGATGGAGA
AAAAGCACAACAATAGGAAAACCTCTTCTATCCTGGCCTCTAGGAGTATCAGGCAAG
CCCAATAGTAAGAACA
>Olfr6 129P2/OlaHsd
TCTGCATCCTATTTCAAATAATATTAATACATTAAGGAGAGATAGTGACTTTATCAC
AGAGAGGAAGGAAAAATGAAGGCTTGGGACCTACCCAGGTGAGACAGTGGGAGGAGT
CAACACATAAAGCAACTAGGGAGACCAGGGATTGTTGGAAGAGAAATAGGTCAGGAG
CAAGAGTAAGAGAAAGAGTGAGAGAAGACTGAGAAAGGCAATGAACAGTGATGGAGA
AAAAGCACAACAATAGGAAAACCTCTTCTATCCTGGCCTCTAGGAGTATCAGGCAAG
CCCAATAGTAAGAACA
>Olfr6 129S1/SvImJ
TCTGCATCCTATTTCAAATAATATTAATACATTAAGGAGAGATAGTGACTTTATCAC
AGAGAGGAAGGAAAAATGAAGGCTTGGGACCTACCCAGGTGAGACAGTGGGAGGAGT
CAACACATAAAGCAACTAGGGAGACCAGGGATTGTTGGAAGAGAAATAGGTCAGGAG
CAAGAGTAAGAGAAAGAGTGAGAGAAGACTGAGAAAGGCAATGAACAGTGATGGAGA
AAAAGCACAACAATAGGAAAACCTCTTCTATCCTGGCCTCTAGGAGTATCAGGCAAG
CCCAATAGTAAGAACA
```

>Olfr6 129S5SvEvBrd
TCTGCATCCTATTTCAAATAATATTAATACATTAAGGAGAGATAGTGACTTTATCAC
AGAGAGGAAGGAAAAATGAAGGCTTGGGACCTACCCAGGTGAGACAGTGGGAGGAGT
CAACACATAAAGCAACTAGGGAGACCAGGGATTGTTGGAAGAGAAATAGGTCAGGAG
CAAGAGTAAGAGAAAGAGTGAGAGAAGACTGAGAAAGGCAATGAACAGTGATGGAGA
AAAAGCACAACAATAGGAAAACCTCTTCTATCCTGGCCTCTAGGAGTATCAGGCAAG
CCCAATAGTAAGAACA
>Olfr6 A/J
TCTGCATCCTATTTCAAATAATATTAATACATTAAGGAGAGATAGTGACTTTATCAC
AGAGAGGAAGGAAAAATGAAGGCTTGGGACCTACCCAGGTGAGACAGTGGGAGGAGT
CAACACATAAAGCAACTAGGGAGACCAGGGATTGTTGGAAGAGAAATAGGTCAGGAG
CAAGAGTAAGAGAAAGAGTGAGAGAAGACTGAGAAAGGCAATGAACAGTGATGGAGA
AAAAGCACAACAATAGGAAAACCTCTTCTATCCTGGCCTCTAGGAGTATCAGGCAAG
CCCAATAGTAAGAACA
>Olfr6 AKR/J
TCTGCATCCTATTTCAAATAATATTAATACATTAAGGAGAGATAGTGACTTTATCAC
AGAGAGGAAGGAAAAATGAAGGCTTGGGACCTACCCAGGTGAGACAGTGGGAGGAGT
CAACACATAAAGCAACTAGGGAGACCAGGGATTGTTGGAAGAGAAATAGGTCAGGAG
CAAGAGTAAGAGAAAGAGTGAGAGAAGACTGAGAAAGGCAATGAACAGTGATGGAGA
AAAAGCACAACAATAGGAAAACCTCTTCTATCCTGGCCTCTAGGAGTATCAGGCAAG
CCCAATAGTAAGAACA
>Olfr6 BALB/cJ
TCTGCATCCTATTTCAAATAATATTAATACATTAAGGAGAGATAGTGACTTTATCAC
AGAGAGGAAGGAAAAATGAAGGCTTGGGACCTACCCAGGTGAGACAGTGGGAGGAGT
CAACACATAAAGCAACTAGGGAGACCAGGGATTGTTGGAAGAGAAATAGGTCAGGAG
CAAGAGTAAGAGAAAGAGTGAGAGAAGACTGAGAAAGGCAATGAACAGTGATGGAGA
AAAAGCACAACAATAGGAAAACCTCTTCTATCCTGGCCTCTAGGAGTATCAGGCAAG
CCCAATAGTAAGAACA
>Olfr6 BTBR/T_+_Itpr3tf/J
TCTGCATCCTATTTCAAATAATATTAATACATTAAGGAGAGATAGTGACTTTATCAC
AGAGAGGAAGGAAAAATGAAGGCTTGGGACCTACCCAGGTGAGACAGTGGGAGGAGT
CAACACATAAAGCAACTAGGGAGACCAGGGATTGTTGGAAGAGAAATAGGTCAGGAG
CAAGAGTAAGAGAAAGAGTGAGAGAAGACTGAGAAAGGCAATGAACAGTGATGGAGA
AAAAGCACAACAATAGGAAAACCTCTTCTATCCTGGCCTCTAGGAGTATCAGGCAAG
CCCAATAGTAAGAACA
>Olfr6 BUB/BnJ
TCTGCATACTATTTCAAATAATCTTAATACATTAAGGAGAGATAGTGACTTTATCAC
AGAGAGGAAGGAAAAATGAAGGCTTGGGACCTACCCAGGTGAGGCAGTGGGAGGAGT
CAACACATAAAGCAACTAGGGAGACCAGGGATTGTTGGAAGAGAAATAGGTCAGGAG
CAAAAGTAAGAGAAAGAGTGAGAGAAGACTGAGAAAGGCAATGAACAGTGATGGAGA
AAAAGCACAACAATAGGAAAACCTCTTCTATCCTGGCCTCTAGGAGTATCAGGCAAG
CCCAATAGTAAGAACA
>Olfr6 C3H/HeH
TCTGCATCCTATTTCAAATAATATTAATACATTAAGGAGAGATAGTGACTTTATCAC
AGAGAGGAAGGAAAAATGAAGGCTTGGGACCTACCCAGGTGAGACAGTGGGAGGAGT
CAACACATAAAGCAACTAGGGAGACCAGGGATTGTTGGAAGAGAAATAGGTCAGGAG
CAAGAGTAAGAGAAAGAGTGAGAGAAGACTGAGAAAGGCAATGAACAGTGATGGAGA
AAAAGCACAACAATAGGAAAACCTCTTCTATCCTGGCCTCTAGGAGTATCAGGCAAG
CCCAATAGTAAGAACA
>Olfr6 C3H/HeJ
TCTGCATCCTATTTCAAATAATATTAATACATTAAGGAGAGATAGTGACTTTATCAC

AGAGAGGAAGGAAAAATGAAGGCTTGGGACCTACCCAGGTGAGACAGTGGGAGGAGT
CAACACATAAAGCAACTAGGGAGACCAGGGATTGTTGGAAGAGAAATAGGTCAGGAG
CAAGAGTAAGAGAAAGAGTGAGAGAAGACTGAGAAAGGCAATGAACAGTGATGGAGA
AAAAGCACAACAATAGGAAAACCTCTTCTATCCTGGCCTCTAGGAGTATCAGGCAAG
CCCAATAGTAAGAACA
>Olfr6 C57BL/10J
TCTGCATACTATTTCAAATAATCTTAATACATTAAGGAGAGATAGTGACTTTATCAC
AGAGAGGAAGGAAAAATGAAGGCTTGGGACCTACCCAGGTGAGGCAGTGGGAGGAGT
CAACACATAAAGCAACTAGGGAGACCAGGGATTGTTGGAAGAGAAATAGGTCAGGAG
CAAAAGTAAGAGAAAGAGTGAGAGAAGACTGAGAAAGGCAATGAACAGTGATGGAGA
AAAAGCACAACAATAGGAAAACCTCTTCTATCCTGGCCTCTAGGAGTATCAGGCAAG
CCCAATAGTAAGAACA
>Olfr6 C57BL/6NJ
TCTGCATACTATTTCAAATAATCTTAATACATTAAGGAGAGATAGTGACTTTATCAC
AGAGAGGAAGGAAAAATGAAGGCTTGGGACCTACCCAGGTGAGGCAGTGGGAGGAGT
CAACACATAAAGCAACTAGGGAGACCAGGGATTGTTGGAAGAGAAATAGGTCAGGAG
CAAAAGTAAGAGAAAGAGTGAGAGAAGACTGAGAAAGGCAATGAACAGTGATGGAGA
AAAAGCACAACAATAGGAAAACCTCTTCTATCCTGGCCTCTAGGAGTATCAGGCAAG
CCCAATAGTAAGAACA
>Olfr6 C57BR/cdJ
TCTGCATACTATTTCAAATAATCTTAATACATTAAGGAGAGATAGTGACTTTATCAC
AGAGAGGAAGGAAAAATGAAGGCTTGGGACCTACCCAGGTGAGGCAGTGGGAGGAGT
CAACACATAAAGCAACTAGGGAGACCAGGGATTGTTGGAAGAGAAATAGGTCAGGAG
CAAAAGTAAGAGAAAGAGTGAGAGAAGACTGAGAAAGGCAATGAACAGTGATGGAGA
AAAAGCACAACAATAGGAAAACCTCTTCTATCCTGGCCTCTAGGAGTATCAGGCAAG
CCCAATAGTAAGAACA
>Olfr6 C57L/J
TCTGCATACTATTTCAAATAATCTTAATACATTAAGGAGAGATAGTGACTTTATCAC
AGAGAGGAAGGAAAAATGAAGGCTTGGGACCTACCCAGGTGAGGCAGTGGGAGGAGT
CAACACATAAAGCAACTAGGGAGACCAGGGATTGTTGGAAGAGAAATAGGTCAGGAG
CAAAAGTAAGAGAAAGAGTGAGAGAAGACTGAGAAAGGCAATGAACAGTGATGGAGA
AAAAGCACAACAATAGGAAAACCTCTTCTATCCTGGCCTCTAGGAGTATCAGGCAAG
CCCAATAGTAAGAACA
>Olfr6 C58/J
TCTGCATACTATTTCAAATAATCTTAATACATTAAGGAGAGATAGTGACTTTATCAC
AGAGAGGAAGGAAAAATGAAGGCTTGGGACCTACCCAGGTGAGGCAGTGGGAGGAGT
CAACACATAAAGCAACTAGGGAGACCAGGGATTGTTGGAAGAGAAATAGGTCAGGAG
CAAAAGTAAGAGAAAGAGTGAGAGAAGACTGAGAAAGGCAATGAACAGTGATGGAGA
AAAAGCACAACAATAGGAAAACCTCTTCTATCCTGGCCTCTAGGAGTATCAGGCAAG
CCCAATAGTAAGAACA
>Olfr6 CAST/EiJ
TCTGCATACTATTTCAAATAATCTTAATACATTAAGGAGAGATAGTGACTTTATCAC
AGAGAGGAAGGAAAAATGAAGGCTTGGGACCTACCCAGGTGAGGCAGTGGGAGGAGT
CAACACATAAAGCAACTAGGGAGACCAGGGATTGTTGGAAGAGAAATAGGTCAGGAG
CAAAAGTAAGAGAAAGAGTGAGAGAAGACTGAGAAAGGCAATGAACAGTGATGGAGA
AAAAGCACAACAATAGGAAAACCTCTTCTATCCTGGCCTCTAGGAGTATCAGGCAAG
CCCAATAGTAAGAACA
>Olfr6 CBA/J
TCTGCATCCTATTTCAAATAATATTAATACATTAAGGAGAGATAGTGACTTTATCAC
AGAGAGGAAGGAAAAATGAAGGCTTGGGACCTACCCAGGTGAGACAGTGGGAGGAGT
CAACACATAAAGCAACTAGGGAGACCAGGGATTGTTGGAAGAGAAATAGGTCAGGAG

CAAGAGTAAGAGAAAGAGTGAGAGAAGACTGAGAAAGGCAATGAACAGTGATGGAGA
AAAAGCACAACAATAGGAAAACCTCTTCTATCCTGGCCTCTAGGAGTATCAGGCAAG
CCCAATAGTAAGAACA
>Olfr6 DBA/1J
TCTGCATCCTATTTCAAATAATATTAATACATTAAGGAGAGATAGTGACTTTATCAC
AGAGAGGAAGGAAAAATGAAGGCTTGGGACCTACCCAGGTGAGACAGTGGGAGGAGT
CAACACATAAAGCAACTAGGGAGACCAGGGATTGTTGGAAGAGAAATAGGTCAGGAG
CAAGAGTAAGAGAAAGAGTGAGAGAAGACTGAGAAAGGCAATGAACAGTGATGGAGA
AAAAGCACAACAATAGGAAAACCTCTTCTATCCTGGCCTCTAGGAGTATCAGGCAAG
CCCAATAGTAAGAACA
>Olfr6 DBA/2J
TCTGCATCCTATTTCAAATAATATTAATACATTAAGGAGAGATAGTGACTTTATCAC
AGAGAGGAAGGAAAAATGAAGGCTTGGGACCTACCCAGGTGAGACAGTGGGAGGAGT
CAACACATAAAGCAACTAGGGAGACCAGGGATTGTTGGAAGAGAAATAGGTCAGGAG
CAAGAGTAAGAGAAAGAGTGAGAGAAGACTGAGAAAGGCAATGAACAGTGATGGAGA
AAAAGCACAACAATAGGAAAACCTCTTCTATCCTGGCCTCTAGGAGTATCAGGCAAG
CCCAATAGTAAGAACA
>Olfr6 FVB/NJ
TCTGCATACTATTTCAAATAATCTTAATACATTAAGGAGAGATAGTGACTTTATCAC
AGAGAGGAAGGAAAAATGAAGGCTTGGGACCTACCCAGGTGAGGCAGTGGGAGGAGT
CAACACATAAAGCAACTAGGGAGACCAGGGATTGTTGGAAGAGAAATAGGTCAGGAG
CAAAAGTAAGAGAAAGAGTGAGAGAAGACTGAGAAAGGCAATGAACAGTGATGGAGA
AAAAGCACAACAATAGGAAAACCTCTTCTATCCTGGCCTCTAGGAGTATCAGGCAAG
CCCAATAGTAAGAACA
>Olfr6 I/LnJ
TCTGCATCCTATTTCAAATAATATTAATACATTAAGGAGAGATAGTGACTTTATCAC
AGAGAGGAAGGAAAAATGAAGGCTTGGGACCTACCCAGGTGAGACAGTGGGAGGAGT
CAACACATAAAGCAACTAGGGAGACCAGGGATTGTTGGAAGAGAAATAGGTCAGGAG
CAAGAGTAAGAGAAAGAGTGAGAGAAGACTGAGAAAGGCAATGAACAGTGATGGAGA
AAAAGCACAACAATAGGAAAACCTCTTCTATCCTGGCCTCTAGGAGTATCAGGCAAG
CCCAATAGTAAGAACA
>Olfr6 KK/HiJ
TCTGCATACTATTTCAAATAATCTTAATACATTAAGGAGAGATAGTGACTTTATCAC
AGAGAGGAAGGAAAAATGAAGGCTTGGGACCTACCCAGGTGAGGCAGTGGGAGGAGT
CAACACATAAAGCAACTAGGGAGACCAGGGATTGTTGGAAGAGAAATAGGTCAGGAG
CAAAAGTAAGAGAAAGAGTGAGAGAAGACTGAGAAAGGCAATGAACAGTGATGGAGA
AAAAGCACAACAATAGGAAAACCTCTTCTATCCTGGCCTCTAGGAGTATCAGGCAAG
CCCAATAGTAAGAACA
>Olfr6 LEWES/EiJ
TCTGCATACTATTTCAAATAATCTTAATACATTAAGGAGAGATAGTGACTTTATCAC
AGAGAGGAAGGAAAAATGAAGGCTTGGGACCTACCCAGGTGAGGCAGTGGGAGGAGT
CAACACATAAAGCAACTAGGGAGACCAGGGATTGTTGGAAGAGAAATAGGTCAGGAG
CAAAAGTAAGAGAAAGAGTGAGAGAAGACTGAGAAAGGCAATGAACAGTGATGGAGA
AAAAGCACAACAATAGGAAAACCTCTTCTATCCTGGCCTCTAGGAGTATCAGGCAAG
CCCAATAGTAAGAACA
>Olfr6 LP/J
TCTGCATCCTATTTCAAATAATATTAATACATTAAGGAGAGATAGTGACTTTATCAC
AGAGAGGAAGGAAAAATGAAGGCTTGGGACCTACCCAGGTGAGACAGTGGGAGGAGT
CAACACATAAAGCAACTAGGGAGACCAGGGATTGTTGGAAGAGAAATAGGTCAGGAG
CAAGAGTAAGAGAAAGAGTGAGAGAAGACTGAGAAAGGCAATGAACAGTGATGGAGA
AAAAGCACAACAATAGGAAAACCTCTTCTATCCTGGCCTCTAGGAGTATCAGGCAAG

CCCAATAGTAAGAACA
>Olfr6 MOLF/EiJ
TCTGCATCCTATTTCAAATAATATTAATACATTAAGGAGAGATAGTGACTTTATCAC
AGAGAGGAAGGAAAAATGAAGGCTTGGGACCTACCTAGGTGAGACAGTGGGAGGAGT
CAACACATAAAGCAACTAGGGAGACCAGGGATTGTTGGAAGAGAAATAGGTCAGGAG
CAAGAGTAAGAGAAAGAGTGAGAGAAGACTGAGAAAGGCAATGAACAGTGATGGAGA
AAAAGCACAACAATAGGAAAACCTCTTCTATCCTGGCCTCTAGGAGTATCAGGCAAG
CCCAATAGTAAGAACA
>Olfr6 NOD/ShiLtJ
TCTGCATCCTATTTCAAATAATATTAATACATTAAGGAGAGATAGTGACTTTATCAC
AGAGAGGAAGGAAAAATGAAGGCTTGGGACCTACCCAGGTGAGACAGTGGGAGGAGT
CAACACATAAAGCAACTAGGGAGACCAGGGATTGTTGGAAGAGAAATAGGTCAGGAG
CAAGAGTAAGAGAAAAAGTGAGAGAAGACTGAGAAAGGCAATGAACAGTGATGGAGA
AAAAGCACAACAATAGGAAAACCTCTTCTATCCTGGCCTCTAGGAGTATCAGGCAAG
CCCAATAGTAAGAACA
>Olfr6 NZB/B1NJ
TCTGCATCCTATTTCAAATAATATTAATACATTAAGGAGAGATAGTGACTTTATCAC
AGAGAGGAAGGAAAAATGAAGGCTTGGGACCTACCCAGGTGAGACAGTGGGAGGAGT
CAACACATAAAGCAACTAGGGAGACCAGGGATTGTTGGAAGAGAAATAGGTCAGGAG
CAAGAGTAAGAGAAAGAGTGAGAGAAGACTGAGAAAGGCAATGAACAGTGATGGAGA
AAAAGCACAACAATAGGAAAACCTCTTCTATCCTGGCCTCTAGGAGTATCAGGCAAG
CCCAATAGTAAGAACA
>Olfr6 NZO/HlLtJ
TCTGCATCCTATTTCAAATAATATTAATACATTAAGGAGAGATAGTGACTTTATCAC
AGAGAGGAAGGAAAAATGAAGGCTTGGGACCTACCCAGGTGAGACAGTGGGAGGAGT
CAACACATAAAGCAACTAGGGAGACCAGGGATTGTTGGAAGAGAAATAGGTCAGGAG
CAAGAGTAAGAGAAAGAGTGAGAGAAGACTGAGAAAGGCAATGAACAGTGATGGAGA
AAAAGCACAACAATAGGAAAACCTCTTCTATCCTGGCCTCTAGGAGTATCAGGCAAG
CCCAATAGTAAGAACA
>Olfr6 NZW/LacJ
TCTGCATCCTATTTCAAATAATATTAATACATTAAGGAGAGATAGTGACTTTATCAC
AGAGAGGAAGGAAAAATGAAGGCTTGGGACCTACCCAGGTGAGACAGTGGGAGGAGT
CAACACATAAAGCAACTAGGGAGACCAGGGATTGTTGGAAGAGAAATAGGTCAGGAG
CAAGAGTAAGAGAAAGAGTGAGAGAAGACTGAGAAAGGCAATGAACAGTGATGGAGA
AAAAGCACAACAATAGGAAAACCTCTTCTATCCTGGCCTCTAGGAGTATCAGGCAAG
CCCAATAGTAAGAACA
>Olfr6 PWK/PhJ
TCTGCATACTATTTCAAATAATCTTAATACATTAAGGAGAGATAGTGACTTTATCAC
AGAGAGGAAGGAAAAATGAAGGCTTGGGACCTACCCAGGTGAGGCAGTGGGAGGAGT
CAACACATAAAGCAACTAGGGAGACCAGGGATTGTTGGAAGAGAAATAGGTCAGGAG
CAAAAGTAAGAGAAAGAGTGAGAGAAGACTGAGAAAGGCAATGAACAGTGATGGAGA
AAAAGCACAACAATAGGAAAACCTCTTCTATCCTGGCCTCTAGGAGTATCAGGCAAG
CCCAATAGTAAGAACA
>Olfr6 RF/J
TCTGCATCCTATTTCAAATAATATTAATACATTAAGGAGAGATAGTGACTTTATCAC
AGAGAGGAAGGAAAAATGAAGGCTTGGGACCTACCCAGGTGAGACAGTGGGAGGAGT
CAACACATAAAGCAACTAGGGAGACCAGGGATTGTTGGAAGAGAAATAGGTCAGGAG
CAAGAGTAAGAGAAAGAGTGAGAGAAGACTGAGAAAGGCAATGAACAGTGATGGAGA
AAAAGCACAACAATAGGAAAACCTCTTCTATCCTGGCCTCTAGGAGTATCAGGCAAG
CCCAATAGTAAGAACA
>Olfr6 SEA/GnJ

TCTGCATCCTATTTCAAATAATATTAATACATTAAGGAGAGATAGTGACTTTATCAC
AGAGAGGAAGGAAAAATGAAGGCTTGGGACCTACCCAGGTGAGACAGTGGGAGGAGT
CAACACATAAAGCAACTAGGGAGACCAGGGATTGTTGGAAGAGAAATAGGTCAGGAG
CAAGAGTAAGAGAAAGAGTGAGAGAAGACTGAGAAAGGCAATGAACAGTGATGGAGA
AAAAGCACAACAATAGGAAAACCTCTTCTATCCTGGCCTCTAGGAGTATCAGGCAAG
CCCAATAGTAAGAACA
>Olfr6 SPRET/EiJ
TCTGCATACTATTTCAAATAATCTTAATACATTAAGGAGAGATAGTGACTTTATCAC
AGAGAGGAAGGAAAAATGAAGGCTTGGGACCTACCCAGGTGAGGCAGTGGGAGGAGT
CAACACATAAAGCAACTAGGGAGACCAGGGATTGTTGGAAGAGAAATAGGTCAGGAG
CAAAAGTAAGAGAAAGAGTGAGAGAAGACTGAGAAAGGCAATGAACAGTGATGGAGA
AAAAGCACAACAATAGGAAAACCTCTTCTATCCTGGCCTCTAGGAGTATCAGGCAAG
CCCAATAGTAAGAACA
>Olfr6 ST/bJ
TCTGCATACTATTTCAAATAATCTTAATACATTAAGGAGAGATAGTGACTTTATCAC
AGAGAGGAAGGAAAAATGAAGGCTTGGGACCTACCCAGGTGAGGCAGTGGGAGGAGT
CAACACATAAAGCAACTAGGGAGACCAGGGATTGTTGGAAGAGAAATAGGTCAGGAG
CAAAAGTAAGAGAAAGAGTGAGAGAAGACTGAGAAAGGCAATGAACAGTGATGGAGA
AAAAGCACAACAATAGGAAAACCTCTTCTATCCTGGCCTCTAGGAGTATCAGGCAAG
CCCAATAGTAAGAACA
>Olfr6 WSB/EiJ
TCTGCATACTATTTCAAATAATCTTAATACATTAAGGAGAGATAGTGACTTTATCAC
AGAGAGGAAGGAAAAATGAAGGCTTGGGACCTACCCAGGTGAGGCAGTGGGAGGAGT
CAACACATAAAGCAACTAGGGAGACCAGGGATTGTTGGAAGAGAAATAGGTCAGGAG
CAAAAGTAAGAGAAAGAGTGAGAGAAGACTGAGAAAGGCAATGAACAGTGATGGAGA
AAAAGCACAACAATAGGAAAACCTCTTCTATCCTGGCCTCTAGGAGTATCAGGCAAG
CCCAATAGTAAGAACA
>Olfr6 ZALENDE/EiJ
TCTGCATACTATTTCAAATAATCTTAATACATTAAGGAGAGATAGTGACTTTATCAC
AGAGAGGAAGGAAAAATGAAGGCTTGGGACCTACCCAGGTGAGGCAGTGGGAGGAGT
CAACACATAAAGCAACTAGGGAGACCAGGGATTGTTGGAAGAGAAATAGGTCAGGAG
CAAAAGTAAGAGAAAGAGTGAGAGAAGACTGAGAAAGGCAATGAACAGTGATGGAGA
AAAAGCACAACAATAGGAAAACCTCTTCTATCCTGGCCTCTAGGAGTATCAGGCAAG
CCCAATAGTAAGAACA

>Olfr19 C57BL/6J
TGAAGTCTGGTTTGCCTAACATTTAATCAGATGTCTTGGGAACAGAGACTCCACAAG
AAACACATATTTCAATGAAAAGCAAAGTCCCATAGGAAAATTATGTAATCAGGTCTG
GAGATGACAAGAGGGAAATATACATAATATGCAGATACACAGACCTTTAAGTACCTC
CCCTGTCTTTGTCTCCACGTATGCAACACTGCTGTGGACAGAACATTTCCGTTTTCT
TAAGGGTGTTCAGCTATCTTGGAAGCTGATTCATGACCCCTTCCTGCTGTCTTCGAA
CTATTTTCAGTCCTTA
>Olfr19 Wild_consensus
TGAAGTCTGGTTTGCCTAACATTTAATCAGATGTCTTGGGAACAGAGACTCCACAAG
AAACACATATTTCAATGAAAAGCAAAGTCCCATAGGAAAATTATGTAATCAGGTCTG
GAGATGACAAGAGGGAAATATACATAATATGCAGATACACAGACCTTTAAGTACCTC
CCCTGTCTTTGTCTCCACGTATGCAACACTGCTGTGGACAGAACATTTCCGTTTTCT
TAAGGGTGTTCAGCTATCTTGGAAGCTGATTCATGACCCCTTCCTGCTGTCTTCGAA
CTATTTTCAGTCCTTA
>Olfr19 France
TGAAGTCTGGTTTGCCTAACATTTAATCAGATGTCTTGGGAACAGAGACTCCACAAG

AAACACATATTTCAATGAAAAGCAAAGTCCCATAGGAAAATTATGTAATCAGGTCTG
GAGATGACAAGAGGGAAATATACATAATATGCAGATACACAGACCTTTAAGTACCTC
CCCTGTCTTTGTCTCCACGTATGCAACACTGCTGTGGACAGAACATTTCCGTTTTCT
TAAGGGTGTTCAGCTATCTTGGAAGCTGATTCATGACCCCTTCCTGCTGTCTTCGAA
CTATTTTCAGTCCTTA
>Olfr19 Germany
TGAAGTCTGGTTTGCCTAACATTTAATCAGATGTCTTGGGAACAGAGACTCCACAAG
AAACACATATTTCAATGAAAAACAAAGTCCCATAGGAAAATTATGTAATCAGGTCTG
GAGATGACAAGAGGGAAATATACATAATATGCAGATACACAGACCTTTAAGTACCTC
CCCTGTCTTTGTCTCCACGTATGCAACACTGCTGTGGACAGAACATTTCCGTTTTCT
TAAGGGTGTTCAGCTATTTTGGAAGCTGATTCACGACCCCTTCCTGCTGTCTTCGAA
CTATTTTCAGTCCTTA
>Olfr19 Iran
TGAAGTCTGGTTTGCCTAACATTTAATCAGATGTCTTGGGAACAGAGACTCCACAAG
AAACACATATTTCAATGAAAAGCAAAGTCCCATAGGAAAATTATGTAATCAGGTCTG
GAGATGACAAGAGGGAAATATACATAATATGCAGATACACAGACCTTTAAGTACCTC
CCCTGTCTTTGTCTCCACGTATGCAACACTGCTGTGGACAGAACATTTCCGTTTTCT
TAAGGGTGTTCAGCTATCTTGGAAGCTGATTCATGACCCCTTCCTGCTGTCTTCGAA
CTATTTTCAGTCCTTA
>Olfr19 129P2/OlaHsd
TGAAGTCTGGTTTGCCTAACATTTAATCAGATGTCTTGGGAACAGAGACTCCACAAG
AAACACATATTTCAATGAAAAGCAAAGTCCCATAGGAAAATTATGTAATCAGGTCTG
GAGATGACAAGAGGGAAATATACATAATATGCAGATACACAGACCTTTAAGTACCTC
CCCTGTCTTTGTCTCCACGTATGCAACACTGCTGTGGACAGAACATTTCCGTTTTCT
TAAGGGTGTTCAGCTATCTTGGAAGCTGATTCATGACCCCTTCCTGCTGTCTTCGAA
CTATTTTCAGTCCTTA
>Olfr19 129S1/SvImJ
TGAAGTCTGGTTTGCCTAACATTTAATCAGATGTCTTGGGAACAGAGACTCCACAAG
AAACACATATTTCAATGAAAAGCAAAGTCCCATAGGAAAATTATGTAATCAGGTCTG
GAGATGACAAGAGGGAAATATACATAATATGCAGATACACAGACCTTTAAGTACCTC
CCCTGTCTTTGTCTCCACGTATGCAACACTGCTGTGGACAGAACATTTCCGTTTTCT
TAAGGGTGTTCAGCTATCTTGGAAGCTGATTCATGACCCCTTCCTGCTGTCTTCGAA
CTATTTTCAGTCCTTA
>Olfr19 129S5SvEvBrd
TGAAGTCTGGTTTGCCTAACATTTAATCAGATGTCTTGGGAACAGAGACTCCACAAG
AAACACATATTTCAATGAAAAGCAAAGTCCCATAGGAAAATTATGTAATCAGGTCTG
GAGATGACAAGAGGGAAATATACATAATATGCAGATACACAGACCTTTAAGTACCTC
CCCTGTCTTTGTCTCCACGTATGCAACACTGCTGTGGACAGAACATTTCCGTTTTCT
TAAGGGTGTTCAGCTATCTTGGAAGCTGATTCATGACCCCTTCCTGCTGTCTTCGAA
CTATTTTCAGTCCTTA
>Olfr19 A/J
TGAAGTCTGGTTTGCCTAACATTTAATCAGATGTCTTGGGAACAGAGACTCCACAAG
AAACACATATTTCAATGAAAAGCAAAGTCCCATAGGAAAATTATGTAATCAGGTCTG
GAGATGACAAGAGGGAAATATACATAATATGCAGATACACAGACCTTTAAGTACCTC
CCCTGTCTTTGTCTCCACGTATGCAACACTGCTGTGGACAGAACATTTCCGTTTTCT
TAAGGGTGTTCAGCTATCTTGGAAGCTGATTCATGACCCCTTCCTGCTGTCTTCGAA
CTATTTTCAGTCCTTA
>Olfr19 AKR/J
TGAAGTCTGGTTTGCCTAACATTTAATCAGATGTCTTGGGAACAGAGACTCCACAAG
AAACACATATTTCAATGAAAAGCAAAGTCCCATAGGAAAATTATGTAATCAGGTCTG
GAGATGACAAGAGGGAAATATACATAATATGCAGATACACAGACCTTTAAGTACCTC

CCCTGTCTTTGTCTCCACGTATGCAACACTGCTGTGGACAGAACATTTCCGTTTTCT
TAAGGGTGTTCAGCTATCTTGGAAGCTGATTCATGACCCCTTCCTGCTGTCTTCGAA
CTATTTTCAGTCCTTA
>Olfr19 BALB/cJ
TGAAGTCTGGTTTGCCTAACATTTAATCAGATGTCTTGGGAACAGAGACTCCACAAG
AAACACATATTTCAATGAAAAGCAAAGTCCCATAGGAAAATTATGTAATCAGGTCTG
GAGATGACAAGAGGGAAATATACATAATATGCAGATACACAGACCTTTAAGTACCTC
CCCTGTCTTTGTCTCCACGTATGCAACACTGCTGTGGACAGAACATTTCCGTTTTCT
TAAGGGTGTTCAGCTATCTTGGAAGCTGATTCATGACCCCTTCCTGCTGTCTTCGAA
CTATTTTCAGTCCTTA
>Olfr19 BTBR/T_+_Itpr3tf/J
TGAAGTCTGGTTTGCCTAACATTTAATCAGATGTCTTGGGAACAGAGACTCCACAAG
AAACACATATTTCAATGAAAAGCAAAGTCCCATAGGAAAATTATGTAATCAGGTCTG
GAGATGACAAGAGGGAAATATACATAATATGCAGATACACAGACCTTTAAGTACCTC
CCCTGTCTTTGTCTCCACGTATGCAACACTGCTGTGGACAGAACATTTCCGTTTTCT
TAAGGGTGTTCAGCTATCTTGGAAGCTGATTCATGACCCCTTCCTGCTGTCTTCGAA
CTATTTTCAGTCCTTA
>Olfr19 BUB/BnJ
TGAAGTCTGGTTTGCCTAACATTTAATCAGATGTCTTGGGAACAGAGACTCCACAAG
AAACACATATTTCAATGAAAAGCAAAGTCCCATAGGAAAATTATGTAATCAGGTCTG
GAGATGACAAGAGGGAAATATACATAATATGCAGATACACAGACCTTTAAGTACCTC
CCCTGTCTTTGTCTCCACGTATGCAACACTGCTGTGGACAGAACATTTCCGTTTTCT
TAAGGGTGTTCAGCTATCTTGGAAGCTGATTCATGACCCCTTCCTGCTGTCTTCGAA
CTATTTTCAGTCCTTA
>Olfr19 C3H/HeH
TGAAGTCTGGTTTGCCTAACATTTAATCAGATGTCTTGGGAACAGAGACTCCACAAG
AAACACATATTTCAATGAAAAGCAAAGTCCCATAGGAAAATTATGTAATCAGGTCTG
GAGATGACAAGAGGGAAATATACATAATATGCAGATACACAGACCTTTAAGTACCTC
CCCTGTCTTTGTCTCCACGTATGCAACACTGCTGTGGACAGAACATTTCCGTTTTCT
TAAGGGTGTTCAGCTATCTTGGAAGCTGATTCATGACCCCTTCCTGCTGTCTTCGAA
CTATTTTCAGTCCTTA
>Olfr19 C3H/HeJ
TGAAGTCTGGTTTGCCTAACATTTAATCAGATGTCTTGGGAACAGAGACTCCACAAG
AAACACATATTTCAATGAAAAGCAAAGTCCCATAGGAAAATTATGTAATCAGGTCTG
GAGATGACAAGAGGGAAATATACATAATATGCAGATACACAGACCTTTAAGTACCTC
CCCTGTCTTTGTCTCCACGTATGCAACACTGCTGTGGACAGAACATTTCCGTTTTCT
TAAGGGTGTTCAGCTATCTTGGAAGCTGATTCATGACCCCTTCCTGCTGTCTTCGAA
CTATTTTCAGTCCTTA
>Olfr19 C57BL/10J
TGAAGTCTGGTTTGCCTAACATTTAATCAGATGTCTTGGGAACAGAGACTCCACAAG
AAACACATATTTCAATGAAAAGCAAAGTCCCATAGGAAAATTATGTAATCAGGTCTG
GAGATGACAAGAGGGAAATATACATAATATGCAGATACACAGACCTTTAAGTACCTC
CCCTGTCTTTGTCTCCACGTATGCAACACTGCTGTGGACAGAACATTTCCGTTTTCT
TAAGGGTGTTCAGCTATCTTGGAAGCTGATTCATGACCCCTTCCTGCTGTCTTCGAA
CTATTTTCAGTCCTTA
>Olfr19 C57BL/6NJ
TGAAGTCTGGTTTGCCTAACATTTAATCAGATGTCTTGGGAACAGAGACTCCACAAG
AAACACATATTTCAATGAAAAGCAAAGTCCCATAGGAAAATTATGTAATCAGGTCTG
GAGATGACAAGAGGGAAATATACATAATATGCAGATACACAGACCTTTAAGTACCTC
CCCTGTCTTTGTCTCCACGTATGCAACACTGCTGTGGACAGAACATTTCCGTTTTCT
TAAGGGTGTTCAGCTATCTTGGAAGCTGATTCATGACCCCTTCCTGCTGTCTTCGAA

CTATTTTCAGTCCTTA
>Olfr19 C57BR/cdJ
TGAAGTCTGGTTTGCCTAACATTTAATCAGATGTCTTGGGAACAGAGACTCCACAAG
AAACACATATTTCAATGAAAAGCAAAGTCCCATAGGAAAATTATGTAATCAGGTCTG
GAGATGACAAGAGGGAAATATACATAATATGCAGATACACAGACCTTTAAGTACCTC
CCCTGTCTTTGTCTCCACGTATGCAACACTGCTGTGGACAGAACATTTCCGTTTTCT
TAAGGGTGTTCAGCTATCTTGGAAGCTGATTCATGACCCCTTCCTGCTGTCTTCGAA
CTATTTTCAGTCCTTA
>Olfr19 C57L/J
TGAAGTCTGGTTTGCCTAACATTTAATCAGATGTCTTGGGAACAGAGACTCCACAAG
AAACACATATTTCAATGAAAAGCAAAGTCCCATAGGAAAATTATGTAATCAGGTCTG
GAGATGACAAGAGGGAAATATACATAATATGCAGATACACAGACCTTTAAGTACCTC
CCCTGTCTTTGTCTCCACGTATGCAACACTGCTGTGGACAGAACATTTCCGTTTTCT
TAAGGGTGTTCAGCTATCTTGGAAGCTGATTCATGACCCCTTCCTGCTGTCTTCGAA
CTATTTTCAGTCCTTA
>Olfr19 C58/J
TGAAGTCTGGTTTGCCTAACATTTAATCAGATGTCTTGGGAACAGAGACTCCACAAG
AAACACATATTTCAATGAAAAGCAAAGTCCCATAGGAAAATTATGTAATCAGGTCTG
GAGATGACAAGAGGGAAATATACATAATATGCAGATACACAGACCTTTAAGTACCTC
CCCTGTCTTTGTCTCCACGTATGCAACACTGCTGTGGACAGAACATTTCCGTTTTCT
TAAGGGTGTTCAGCTATCTTGGAAGCTGATTCATGACCCCTTCCTGCTGTCTTCGAA
CTATTTTCAGTCCTTA
>Olfr19 CAST/EiJ
TGAAGTCTGGTTTGCCTAACATTTAATCAGATGTCTTGGGAACAGAGACTCCACAAG
AAACACATATTTCAATGAAAAGCAAAGTCCCATAGGAAAATTATGTAATCAGGTCTG
GAGATGACAAGAGGGAAATATACATAATATGCAGATACACAGACCTTTAAGTACCTC
CTCTGTCTTTGTCTCCACGTATGCAACATTGCTGTGGACAGAACATTTCCGTTTTCT
TAAGGGTGTTCAGCTATCTTGGAAGCTGATTCATGACCCCTTCCTGCTGTCTTCGAA
CTATTTTCAGTCCTTA
>Olfr19 CBA/J
TGAAGTCTGGTTTGCCTAACATTTAATCAGATGTCTTGGGAACAGAGACTCCACAAG
AAACACATATTTCAATGAAAAGCAAAGTCCCATAGGAAAATTATGTAATCAGGTCTG
GAGATGACAAGAGGGAAATATACATAATATGCAGATACACAGACCTTTAAGTACCTC
CCCTGTCTTTGTCTCCACGTATGCAACACTGCTGTGGACAGAACATTTCCGTTTTCT
TAAGGGTGTTCAGCTATCTTGGAAGCTGATTCATGACCCCTTCCTGCTGTCTTCGAA
CTATTTTCAGTCCTTA
>Olfr19 DBA/1J
TGAAGTCTGGTTTGCCTAACATTTAATCAGATGTCTTGGGAACAGAGACTCCACAAG
AAACACATATTTCAATGAAAAGCAAAGTCCCATAGGAAAATTATGTAATCAGGTCTG
GAGATGACAAGAGGGAAATATACATAATATGCAGATACACAGACCTTTAAGTACCTC
CCCTGTCTTTGTCTCCACGTATGCAACACTGCTGTGGACAGAACATTTCCGTTTTCT
TAAGGGTGTTCAGCTATCTTGGAAGCTGATTCATGACCCCTTCCTGCTGTCTTCGAA
CTATTTTCAGTCCTTA
>Olfr19 DBA/2J
TGAAGTCTGGTTTGCCTAACATTTAATCAGATGTCTTGGGAACAGAGACTCCACAAG
AAACACATATTTCAATGAAAAGCAAAGTCCCATAGGAAAATTATGTAATCAGGTCTG
GAGATGACAAGAGGGAAATATACATAATATGCAGATACACAGACCTTTAAGTACCTC
CCCTGTCTTTGTCTCCACGTATGCAACACTGCTGTGGACAGAACATTTCCGTTTTCT
TAAGGGTGTTCAGCTATCTTGGAAGCTGATTCATGACCCCTTCCTGCTGTCTTCGAA
CTATTTTCAGTCCTTA
>Olfr19 FVB/NJ

TGAAGTCTGGTTTGCCTAACATTTAATCAGATGTCTTGGGAACAGAGACTCCACAAG
AAACACATATTTCAATGAAAAGCAAAGTCCCATAGGAAAATTATGTAATCAGGTCTG
GAGATGACAAGAGGGAAATATACATAATATGCAGATACACAGACCTTTAAGTACCTC
CCCTGTCTTTGTCTCCACGTATGCAACACTGCTGTGGACAGAACATTTCCGTTTTCT
TAAGGGTGTTCAGCTATCTTGGAAGCTGATTCATGACCCCTTCCTGCTGTCTTCGAA
CTATTTTCAGTCCTTA
>Olfr19 I/LnJ
TGAAGTCTGGTTTGCCTAACATTTAATCAGATGTCTTGGGAACAGAGACTCCACAAG
AAACACATATTTCAATGAAAAGCAAAGTCCCATAGGAAAATTATGTAATCAGGTCTG
GAGATGACAAGAGGGAAATATACATAATATGCAGATACACAGACCTTTAAGTACCTC
CCCTGTCTTTGTCTCCACGTATGCAACACTGCTGTGGACAGAACATTTCCGTTTTCT
TAAGGGTGTTCAGCTATCTTGGAAGCTGATTCATGACCCCTTCCTGCTGTCTTCGAA
CTATTTTCAGTCCTTA
>Olfr19 KK/HiJ
TGAAGTCTGGTTTGCCTAACATTTAATCAGATGTCTTGGGAACAGAGACTCCACAAG
AAACACATATTTCAATGAAAAGCAAAGTCCCATAGGAAAATTATGTAATCAGGTCTG
GAGATGACAAGAGGGAAATATACATAATATGCAGATACACAGACCTTTAAGTACCTC
CCCTGTCTTTGTCTCCACGTATGCAACACTGCTGTGGACAGAACATTTCCGTTTTCT
TAAGGGTGTTCAGCTATCTTGGAAGCTGATTCATGACCCCTTCCTGCTGTCTTCGAA
CTATTTTCAGTCCTTA
>Olfr19 LEWES/EiJ
TGAAGTCTGGTTTGCCTAACATTTAATCAGATGTCTTGGGAACAGAGACTCCACAAG
AAACACATATTTCAATGAAAAGCAAAGTCCCATAGGAAAATTATGTAATCAGGTCTG
GAGATGACAAGAGGGAAATATACATAATATGCAGATACACAGACCTTTAAGTACCTC
CCCTGTCTTTGTCTCCACGTATGCAACACTGCTGTGGACAGAACATTTCCGTTTTCT
TAAGGGTGTTCAGCTATCTTGGAAGCTGATTCATGACCCCTTCCTGCTGTCTTCGAA
CTATTTTCAGTCCTTA
>Olfr19 LP/J
TGAAGTCTGGTTTGCCTAACATTTAATCAGATGTCTTGGGAACAGAGACTCCACAAG
AAACACATATTTCAATGAAAAGCAAAGTCCCATAGGAAAATTATGTAATCAGGTCTG
GAGATGACAAGAGGGAAATATACATAATATGCAGATACACAGACCTTTAAGTACCTC
CCCTGTCTTTGTCTCCACGTATGCAACACTGCTGTGGACAGAACATTTCCGTTTTCT
TAAGGGTGTTCAGCTATCTTGGAAGCTGATTCATGACCCCTTCCTGCTGTCTTCGAA
CTATTTTCAGTCCTTA
>Olfr19 MOLF/EiJ
TGAAGTTTGGTTTGCCTAACATTTAATCAGATGTCTTGGGAACAGAGACTCCACAAG
AAACACATATTTCAATGAAAAGCAAAGTCCCATAGGAAAATTATGTAATCAGGTCTG
GAGATGACAAGAGGGAAATATACATAATATGCAGATACACAGACCTTTAAGTACCTC
CCCTGTCTTTGTCTCCACGTATGCAACACTGCTGTGGACAGAACATTTCCGTTTTCT
TAAGGGTGTTCAGCTATTTTGGAAGCTGATTCATGACCCCTTCCTGCTGTCTTCGAA
CTATTTTCAGTCCTTA
>Olfr19 NOD/ShiLtJ
TGAAGTCTGGTTTGCCTAACATTTAATCAGATGTCTTGGGAACAGAGACTCCACAAG
AAACACATATTTCAATGAAAAGCAAAGTCCCATAGGAAAATTATGTAATCAGGTCTG
GAGATGACAAGAGGGAAATATACATAATATGCAGATACACAGACCTTTAAGTACCTC
CCCTGTCTTTGTCTCCACGTATGCAACACTGCTGTGGACAGAACATTTCCGTTTTCT
TAAGGGTGTTCAGCTATCTTGGAAGCTGATTCATGACCCCTTCCTGCTGTCTTCGAA
CTATTTTCAGTCCTTA
>Olfr19 NZB/B1NJ
TGAAGTCTGGTTTGCCTAACATTTAATCAGATGTCTTGGGAACAGAGACTCCACAAG
AAACACATATTTCAATGAAAAGCAAAGTCCCATAGGAAAATTATGTAATCAGGTCTG

GAGATGACAAGAGGGAAATATACATAATATGCAGATACACAGACCTTTAAGTACCTC
CCCTGTCTTTGTCTCCACGTATGCAACACTGCTGTGGACAGAACATTTCCGTTTTCT
TAAGGGTGTTCAGCTATCTTGGAAGCTGATTCATGACCCCTTCCTGCTGTCTTCGAA
CTATTTTCAGTCCTTA
>Olfr19 NZO/HlLtJ
TGAAGTCTGGTTTGCCTAACATTTAATCAGATGTCTTGGGAACAGAGACTCCACAAG
AAACACATATTTCAATGAAAAGCAAAGTCCCATAGGAAAATTATGTAATCAGGTCTG
GAGATGACAAGAGGGAAATATACATAATATGCAGATACACAGACCTTTAAGTACCTC
CCCTGTCTTTGTCTCCACGTATGCAACACTGCTGTGGACAGAACATTTCCGTTTTCT
TAAGGGTGTTCAGCTATCTTGGAAGCTGATTCATGACCCCTTCCTGCTGTCTTCGAA
CTATTTTCAGTCCTTA
>Olfr19 NZW/LacJ
TGAAGTCTGGTTTGCCTAACATTTAATCAGATGTCTTGGGAACAGAGACTCCACAAG
AAACACATATTTCAATGAAAAGCAAAGTCCCATAGGAAAATTATGTAATCAGGTCTG
GAGATGACAAGAGGGAAATATACATAATATGCAGATACACAGACCTTTAAGTACCTC
CCCTGTCTTTGTCTCCACGTATGCAACACTGCTGTGGACAGAACATTTCCGTTTTCT
TAAGGGTGTTCAGCTATCTTGGAAGCTGATTCATGACCCCTTCCTGCTGTCTTCGAA
CTATTTTCAGTCCTTA
>Olfr19 PWK/PhJ
TGAAGTTTGGTTTGCCTAACATTTAATCAGATGTCTTGGGAACAGAGACTCCACAAG
AAACACATATTTCAATGAAAAGCAAAGTCCCATAGGAAAATTATGTAATCAGGTCTG
GAGATGACAAGAGGGAAATATACATAATATGCAGATACACAGACCTTTAAGTACCTC
CCCTGTCTTTGTCTCCACGTATGCAACACTGCTGTGGACAGAACATTTCCGTTTTCT
TAAGGGTGTTCAGCTATTTTGGAAGCTGATTCATGACCCCTTCCTGCTGTCTTCGAA
CTATTTTCAGTCCTTA
>Olfr19 RF/J
TGAAGTCTGGTTTGCCTAACATTTAATCAGATGTCTTGGGAACAGAGACTCCACAAG
AAACACATATTTCAATGAAAAGCAAAGTCCCATAGGAAAATTATGTAATCAGGTCTG
GAGATGACAAGAGGGAAATATACATAATATGCAGATACACAGACCTTTAAGTACCTC
CCCTGTCTTTGTCTCCACGTATGCAACACTGCTGTGGACAGAACATTTCCGTTTTCT
TAAGGGTGTTCAGCTATCTTGGAAGCTGATTCATGACCCCTTCCTGCTGTCTTCGAA
CTATTTTCAGTCCTTA
>Olfr19 SEA/GnJ
TGAAGTCTGGTTTGCCTAACATTTAATCAGATGTCTTGGGAACAGAGACTCCACAAG
AAACACATATTTCAATGAAAAGCAAAGTCCCATAGGAAAATTATGTAATCAGGTCTG
GAGATGACAAGAGGGAAATATACATAATATGCAGATACACAGACCTTTAAGTACCTC
CCCTGTCTTTGTCTCCACGTATGCAACACTGCTGTGGACAGAACATTTCCGTTTTCT
TAAGGGTGTTCAGCTATCTTGGAAGCTGATTCATGACCCCTTCCTGCTGTCTTCGAA
CTATTTTCAGTCCTTA
>Olfr19 SPRET/EiJ
TGAAGTCTGGTTTGCCTAACATTTAATCAGATGTCTTGGGAACAGAGACTCCACAAG
AAACACATATTTCAATGAAAAGCAAAGTCCCATAGGAAAATTATGTAATCAGGTCTG
GAGATGACAAGAGGGAAATATACATAATATGAAGATACACAGACCTTTAAGTACCTC
CCCTGTCTTTGTCTCCACGTATGCAACACTGCTGTGGACAGAACATTTTCGTTTTCT
TAAGGGTGTTCAGCTATCTTGGAAGCTGATTCATGACCCCTTCCTGCTGTCTTCGAA
CTATTTTCGGTCCTTA
>Olfr19 ST/bJ
TGAAGTCTGGTTTGCCTAACATTTAATCAGATGTCTTGGGAACAGAGACTCCACAAG
AAACACATATTTCAATGAAAAGCAAAGTCCCATAGGAAAATTATGTAATCAGGTCTG
GAGATGACAAGAGGGAAATATACATAATATGCAGATACACAGACCTTTAAGTACCTC
CCCTGTCTTTGTCTCCACGTATGCAACACTGCTGTGGACAGAACATTTCCGTTTTCT

TAAGGGTGTTCAGCTATCTTGGAAGCTGATTCATGACCCCTTCCTGCTGTCTTCGAA
CTATTTTCAGTCCTTA
>Olfr19 WSB/EiJ
TGAAGTCTGGTTTGCCTAACATTTAATCAGATGTCTTGGGAACAGAGACTCCACAAG
AAACACATATTTCAATGAAAAGCAAAGTCCCATAGGAAAATTATGTAATCAGGTCTG
GAGATGACAAGAGGGAAATATACATAATATGCAGATACACAGACCTTTAAGTACCTC
CCCTGTCTTTGTCTCCACGTATGCAACACTGCTGTGGACAGAACATTTCCGTTTTCT
TAAGGGTGTTCAGCTATCTTGGAAGCTGATTCATGACCCCTTCCTGCTGTCTTCGAA
CTATTTTCAGTCCTTA
>Olfr19 ZALENDE/EiJ
TGAAGTCTGGTTTGCCTAACATTTAATCAGATGTCTTGGGAACAGAGACTCCACAAG
AAACACATATTTCAATGAAAAGCAAAGTCCCATAGGAAAATTATGTAATCAGGTCTG
GAGATGACAAGAGGGAAATATACATAATATGCAGATACACAGACCTTTAAGTACCTC
CCCTGTCTTTGTCTCCACGTATGCAACACTGCTGTGGACAGAACATTTCCGTTTTCT
TAAGGGTGTTCAGCTATCTTGGAAGCTGATTCATGACCCCTTCCTGCTGTCTTCGAA
CTATTTTCAGTCCTTA

>Olfr49 C57BL/6J
TCTTCTGAGAACAAAGTAAACTTTTAGTCTGAGAGTTGAATGAAGAGGAAAGGGACC
TTCCTAGATCCCTGTTATTGTTGATGGTAATGAATCTCAAGGGAAGGATAGCTCCAG
CCAGCAAAAACAGAGAATCTCAGATGAGTCAAATCTGAGCCATGCATCTTTGCACAT
CTCGAATTCCCATAGAGACCAAAGAGAATGTCTAATACCTCAGGCCTCTTCCCAGTA
CACAGAAACAAAGGGTCTACCCTGATTGCTGTACCCCAGAGTCTTCTGTGGGCACAG
TGGGACTGGAAGATCA
>Olfr49 Wild_consensus
TCTTCTGAGAACAAAGTAAACTTTTAGTCTGAGAGTTGAATGAAGAGGAAAGGGACC
TTCCTAGATCCCTGTTATTGTTGATGGTAATGAATCTCAAGGGAAGGATAGCTCCAG
CCAGCAAAAACAGAGAATCTCAGATGAGTCAAATCTGAGCCATGCATCTTTGCACAT
CTCGAATTCCCATAGAGACCAAAGAGAATGTCTAATACCTCAGGCCTCTTCCCAGTA
CACAGAAACAAAGGGTCTACCCTGATTGCTGTACCCCAGAGTCTTCTGTGGGCACAG
TGGGACTGGAAGATCA
>Olfr49 France
TCTTCTGAGAACAAAGTAAACTTTTAGTCTGAGAGTTGAATGAAGAGGAAAGGGACC
TTCCTAGATCCCTGTTATTGTTGATGGTAATGAATCTCAAGGGAAGGATAGCTCCAG
CCAGCAAAAACAGAGAATCTCAGATGAGTCAAATCTGAGCCATGCATCTTTGCACAT
CTCGAATTCCCATAGAGACCAAAGAGAATGTCTAATACCTCAGGCCTCTTCCCAGTA
CACAGAAACAAAGGGTCTACCCTGATTGCTGTACCCCAGAGTCTTCTGTGGGCACAG
TGGGACTGGAAGATCA
>Olfr49 Germany
TCTTCTGAGAACAAAGTAAACTTTTAGTCTGAGAGTTGAATGAAGAGGAAAGGGACC
TTCCTAGATCCCTGTTATTGTTGATGGTAATGAATCTCAAGGGAAGGATAGCTCCAG
CCAGCAAAAACAGAGAATCTCAGATGAGTCAAATCTGAGCCATGCATCTTTGCACAT
CTCGAATTCCCATAGAGACCAAAGAGAATGTCTAATACCTCAGGCCTCTTCCCAGTA
CACAGAAACAAAGGGTCTACCCTGATTGCTGTACCCCAGAGTCTTCTGTGGGCACAG
TGGGACTGGAAGATCA
>Olfr49 Iran
TCTTCTGAGAACAAAGTAAACTTTTAGTCTGAGAGTTGAATGAAGAGGAAAGGGACC
TTCCTAGATCCCTGTTATTGTTGATGGTAATGAATCTCAAGGGAAGGATAGCTCCAG
CCAGCAAAAACAGAGAATCTCAGATGAGTCAAATCTGAGCCATGCATCTTTGCACAT
CTCGAATTCCCATAGAGACCAAAGAGAATGTCTAATACCTCAGGCCTCTTCCCAGTA
CACAGAAACAAAGGGTCTACCCTGATTGCTGTACCCCAGAGTCTTCTGTGGGCACAG

TGGGACTGGAAGATCA
>Olfr49 129P2/OlaHsd
TCTTCTGAGAACAAAGTAAACTTTTAGTCTGAGAGTTGAATGAAGAGGAAAGGGACC
TTCCTAGATCCCTGTTATTGTTGATGGTAATGAATCTCAAGGGAAGGATAGCTCCAG
CCAGCAAAAACAGAGAATCTCAGATGAGTCAAATCTGAGCCATGCATCTTTGCACAT
CTCGAATTCCCATAGAGACCAAAGAGAATGTCTAATACCTCAGGCCTCTTCCCAGTA
CACAGAAACAAAGGGTCTACCCTGATTGCTGTACCCCAGAGTCTTCTGTGGGCACAG
TGGGACTGGAAGATCA
>Olfr49 129S1/SvImJ
TCTTCTGAGAACAAAGTAAACTTTTAGTCTGAGAGTTGAATGAAGAGGAAAGGGACC
TTCCTAGATCCCTGTTATTGTTGATGGTAATGAATCTCAAGGGAAGGATAGCTCCAG
CCAGCAAAAACAGAGAATCTCAGATGAGTCAAATCTGAGCCATGCATCTTTGCACAT
CTCGAATTCCCATAGAGACCAAAGAGAATGTCTAATACCTCAGGCCTCTTCCCAGTA
CACAGAAACAAAGGGTCTACCCTGATTGCTGTACCCCAGAGTCTTCTGTGGGCACAG
TGGGACTGGAAGATCA
>Olfr49 129S5SvEvBrd
TCTTCTGAGAACAAAGTAAACTTTTAGTCTGAGAGTTGAATGAAGAGGAAAGGGACC
TTCCTAGATCCCTGTTATTGTTGATGGTAATGAATCTCAAGGGAAGGATAGCTCCAG
CCAGCAAAAACAGAGAATCTCAGATGAGTCAAATCTGAGCCATGCATCTTTGCACAT
CTCGAATTCCCATAGAGACCAAAGAGAATGTCTAATACCTCAGGCCTCTTCCCAGTA
CACAGAAACAAAGGGTCTACCCTGATTGCTGTACCCCAGAGTCTTCTGTGGGCACAG
TGGGACTGGAAGATCA
>Olfr49 A/J
TCTTCTGAGAACAAAGTAAACTTTTAGTCTGAGAGTTGAATGAAGAGGAAAGGGACC
TTCCTAGATCCCTGTTATTGTTGATGGTAATGAATCTCAAGGGAAGGATAGCTCCAG
CCAGCAAAAACAGAGAATCTCAGATGAGTCAAATCTGAGCCATGCATCTTTGCACAT
CTCGAATTCCCATAGAGACCAAAGAGAATGTCTAATACCTCAGGCCTCTTCCCAGTA
CACAGAAACAAAGGGTCTACCCTGATTGCTGTACCCCAGAGTCTTCTGTGGGCACAG
TGGGACTGGAAGATCA
>Olfr49 AKR/J
TCTTCTGAGAACAAAGTAAACTTTTAGTCTGAGAGTTGAATGAAGAGGAAAGGGACC
TTCCTAGATCCCTGTTATTGTTGATGGTAATGAATCTCAAGGGAAGGATAGCTCCAG
CCAGCAAAAACAGAGAATCTCAGATGAGTCAAATCTGAGCCATGCATCTTTGCACAT
CTCGAATTCCCATAGAGACCAAAGAGAATGTCTAATACCTCAGGCCTCTTCCCAGTA
CACAGAAACAAAGGGTCTACCCTGATTGCTGTACCCCAGAGTCTTCTGTGGGCACAG
TGGGACTGGAAGATCA
>Olfr49 BALB/cJ
TCTTCTGAGAACAAAGTAAACTTTTAGTCTGAGAGTTGAATGAAGAGGAAAGGGACC
TTCCTAGATCCCTGTTATTGTTGATGGTAATGAATCTCAAGGGAAGGATAGCTCCAG
CCAGCAAAAACAGAGAATCTCAGATGAGTCAAATCTGAGCCATGCATCTTTGCACAT
CTCGAATTCCCATAGAGACCAAAGAGAATGTCTAATACCTCAGGCCTCTTCCCAGTA
CACAGAAACAAAGGGTCTACCCTGATTGCTGTACCCCAGAGTCTTCTGTGGGCACAG
TGGGACTGGAAGATCA
>Olfr49 BTBR/T_+_Itpr3tf/J
TCTTCTGAGAACAAAGTAAACTTTTAGTCTGAGAGTTGAATGAAGAGGAAAGGGACC
TTCCTAGATCCCTGTTATTGTTGATGGTAATGAATCTCAAGGGAAGGATAGCTCCAG
CCAGCAAAAACAGAGAATCTCAGATGAGTCAAATCTGAGCCATGCATCTTTGCACAT
CTCGAATTCCCATAGAGACCAAAGAGAATGTCTAATACCTCAGGCCTCTTCCCAGTA
CACAGAAACAAAGGGTCTACCCTGATTGCTGTACCCCAGAGTCTTCTGTGGGCACAG
TGGGACTGGAAGATCA
>Olfr49 BUB/BnJ

TCTTCTGAGAACAAAGTAAACTTTTAGTCTGAGAGTTGAATGAAGAGGAAAGGGACC
TTCCTAGATCCCTGTTATTGTTGATGGTAATGAATCTCAAGGGAAGGATAGCTCCAG
CCAGCAAAAACAGAGAATCTCAGATGAGTCAAATCTGAGCCATGCATCTTTGCACAT
CTCGAATTCCCATAGAGACCAAAGAGAATGTCTAATACCTCAGGCCTCTTCCCAGTA
CACAGAAACAAAGGGTCTACCCTGATTGCTGTACCCCAGAGTCTTCTGTGGGCACAG
TGGGACTGGAAGATCA
>Olfr49 C3H/HeH
TCTTCTGAGAACAAAGTAAACTTTTAGTCTGAGAGTTGAATGAAGAGGAAAGGGACC
TTCCTAGATCCCTGTTATTGTTGATGGTAATGAATCTCAAGGGAAGGATAGCTCCAG
CCAGCAAAAACAGAGAATCTCAGATGAGTCAAATCTGAGCCATGCATCTTTGCACAT
CTCGAATTCCCATAGAGACCAAAGAGAATGTCTAATACCTCAGGCCTCTTCCCAGTA
CACAGAAACAAAGGGTCTACCCTGATTGCTGTACCCCAGAGTCTTCTGTGGGCACAG
TGGGACTGGAAGATCA
>Olfr49 C3H/HeJ
TCTTCTGAGAACAAAGTAAACTTTTAGTCTGAGAGTTGAATGAAGAGGAAAGGGACC
TTCCTAGATCCCTGTTATTGTTGATGGTAATGAATCTCAAGGGAAGGATAGCTCCAG
CCAGCAAAAACAGAGAATCTCAGATGAGTCAAATCTGAGCCATGCATCTTTGCACAT
CTCGAATTCCCATAGAGACCAAAGAGAATGTCTAATACCTCAGGCCTCTTCCCAGTA
CACAGAAACAAAGGGTCTACCCTGATTGCTGTACCCCAGAGTCTTCTGTGGGCACAG
TGGGACTGGAAGATCA
>Olfr49 C57BL/10J
TCTTCTGAGAACAAAGTAAACTTTTAGTCTGAGAGTTGAATGAAGAGGAAAGGGACC
TTCCTAGATCCCTGTTATTGTTGATGGTAATGAATCTCAAGGGAAGGATAGCTCCAG
CCAGCAAAAACAGAGAATCTCAGATGAGTCAAATCTGAGCCATGCATCTTTGCACAT
CTCGAATTCCCATAGAGACCAAAGAGAATGTCTAATACCTCAGGCCTCTTCCCAGTA
CACAGAAACAAAGGGTCTACCCTGATTGCTGTACCCCAGAGTCTTCTGTGGGCACAG
TGGGACTGGAAGATCA
>Olfr49 C57BL/6NJ
TCTTCTGAGAACAAAGTAAACTTTTAGTCTGAGAGTTGAATGAAGAGGAAAGGGACC
TTCCTAGATCCCTGTTATTGTTGATGGTAATGAATCTCAAGGGAAGGATAGCTCCAG
CCAGCAAAAACAGAGAATCTCAGATGAGTCAAATCTGAGCCATGCATCTTTGCACAT
CTCGAATTCCCATAGAGACCAAAGAGAATGTCTAATACCTCAGGCCTCTTCCCAGTA
CACAGAAACAAAGGGTCTACCCTGATTGCTGTACCCCAGAGTCTTCTGTGGGCACAG
TGGGACTGGAAGATCA
>Olfr49 C57BR/cdJ
TCTTCTGAGAACAAAGTAAACTTTTAGTCTGAGAGTTGAATGAAGAGGAAAGGGACC
TTCCTAGATCCCTGTTATTGTTGATGGTAATGAATCTCAAGGGAAGGATAGCTCCAG
CCAGCAAAAACAGAGAATCTCAGATGAGTCAAATCTGAGCCATGCATCTTTGCACAT
CTCGAATTCCCATAGAGACCAAAGAGAATGTCTAATACCTCAGGCCTCTTCCCAGTA
CACAGAAACAAAGGGTCTACCCTGATTGCTGTACCCCAGAGTCTTCTGTGGGCACAG
TGGGACTGGAAGATCA
>Olfr49 C57L/J
TCTTCTGAGAACAAAGTAAACTTTTAGTCTGAGAGTTGAATGAAGAGGAAAGGGACC
TTCCTAGATCCCTGTTATTGTTGATGGTAATGAATCTCAAGGGAAGGATAGCTCCAG
CCAGCAAAAACAGAGAATCTCAGATGAGTCAAATCTGAGCCATGCATCTTTGCACAT
CTCGAATTCCCATAGAGACCAAAGAGAATGTCTAATACCTCAGGCCTCTTCCCAGTA
CACAGAAACAAAGGGTCTACCCTGATTGCTGTACCCCAGAGTCTTCTGTGGGCACAG
TGGGACTGGAAGATCA
>Olfr49 C58/J
TCTTCTGAGAACAAAGTAAACTTTTAGTCTGAGAGTTGAATGAAGAGGAAAGGGACC
TTCCTAGATCCCTGTTATTGTTGATGGTAATGAATCTCAAGGGAAGGATAGCTCCAG

CCAGCAAAAACAGAGAATCTCAGATGAGTCAAATCTGAGCCATGCATCTTTGCACAT
CTCGAATTCCCATAGAGACCAAAGAGAATGTCTAATACCTCAGGCCTCTTCCCAGTA
CACAGAAACAAAGGGTCTACCCTGATTGCTGTACCCCAGAGTCTTCTGTGGGCACAG
TGGGACTGGAAGATCA
>Olfr49 CAST/EiJ
TCTTCTGAGAACAAAGTAAACTTTTAGTCTGAGAGTTGAATGAAGAGGAAAGGGACC
TTCCTAGATCCCTGTTATTGTTGATGGTAATGAATCTCAAGGGAAGGATAGCTCCAG
CCAGCAAAAACAGAGAATCTCAGATGAGTCAAATCTGAGCCATGCATCTTTGCACAT
CTCGAATTCCCATAGAGACCAAAGAGAATGTCTAATACCTCAGGCCTCTTCCCAGTA
CACAGAAACAAAGGGTCTACCCTGATTGCTGTACCCCAGAGTCTTCTGTGGGCACAG
TGGGACTGGAAGATCA
>Olfr49 CBA/J
TCTTCTGAGAACAAAGTAAACTTTTAGTCTGAGAGTTGAATGAAGAGGAAAGGGACC
TTCCTAGATCCCTGTTATTGTTGATGGTAATGAATCTCAAGGGAAGGATAGCTCCAG
CCAGCAAAAACAGAGAATCTCAGATGAGTCAAATCTGAGCCATGCATCTTTGCACAT
CTCGAATTCCCATAGAGACCAAAGAGAATGTCTAATACCTCAGGCCTCTTCCCAGTA
CACAGAAACAAAGGGTCTACCCTGATTGCTGTACCCCAGAGTCTTCTGTGGGCACAG
TGGGACTGGAAGATCA
>Olfr49 DBA/1J
TCTTCTGAGAACAAAGTAAACTTTTAGTCTGAGAGTTGAATGAAGAGGAAAGGGACC
TTCCTAGATCCCTGTTATTGTTGATGGTAATGAATCTCAAGGGAAGGATAGCTCCAG
CCAGCAAAAACAGAGAATCTCAGATGAGTCAAATCTGAGCCATGCATCTTTGCACAT
CTCGAATTCCCATAGAGACCAAAGAGAATGTCTAATACCTCAGGCCTCTTCCCAGTA
CACAGAAACAAAGGGTCTACCCTGATTGCTGTACCCCAGAGTCTTCTGTGGGCACAG
TGGGACTGGAAGATCA
>Olfr49 DBA/2J
TCTTCTGAGAACAAAGTAAACTTTTAGTCTGAGAGTTGAATGAAGAGGAAAGGGACC
TTCCTAGATCCCTGTTATTGTTGATGGTAATGAATCTCAAGGGAAGGATAGCTCCAG
CCAGCAAAAACAGAGAATCTCAGATGAGTCAAATCTGAGCCATGCATCTTTGCACAT
CTCGAATTCCCATAGAGACCAAAGAGAATGTCTAATACCTCAGGCCTCTTCCCAGTA
CACAGAAACAAAGGGTCTACCCTGATTGCTGTACCCCAGAGTCTTCTGTGGGCACAG
TGGGACTGGAAGATCA
>Olfr49 FVB/NJ
TCTTCTGAGAACAAAGTAAACTTTTAGTCTGAGAGTTGAATGAAGAGGAAAGGGACC
TTCCTAGATCCCTGTTATTGTTGATGGTAATGAATCTCAAGGGAAGGATAGCTCCAG
CCAGCAAAAACAGAGAATCTCAGATGAGTCAAATCTGAGCCATGCATCTTTGCACAT
CTCGAATTCCCATAGAGACCAAAGAGAATGTCTAATACCTCAGGCCTCTTCCCAGTA
CACAGAAACAAAGGGTCTACCCTGATTGCTGTACCCCAGAGTCTTCTGTGGGCACAG
TGGGACTGGAAGATCA
>Olfr49 I/LnJ
TCTTCTGAGAACAAAGTAAACTTTTAGTCTGAGAGTTGAATGAAGAGGAAAGGGACC
TTCCTAGATCCCTGTTATTGTTGATGGTAATGAATCTCAAGGGAAGGATAGCTCCAG
CCAGCAAAAACAGAGAATCTCAGATGAGTCAAATCTGAGCCATGCATCTTTGCACAT
CTCGAATTCCCATAGAGACCAAAGAGAATGTCTAATACCTCAGGCCTCTTCCCAGTA
CACAGAAACAAAGGGTCTACCCTGATTGCTGTACCCCAGAGTCTTCTGTGGGCACAG
TGGGACTGGAAGATCA
>Olfr49 KK/HiJ
TCTTCTGAGAACAAAGTAAACTTTTAGTCTGAGAGTTGAATGAAGAGGAAAGGGACC
TTCCTAGATCCCTGTTATTGTTGATGGTAATGAATCTCAAGGGAAGGATAGCTCCAG
CCAGCAAAAACAGAGAATCTCAGATGAGTCAAATCTGAGCCATGCATCTTTGCACAT
CTCGAATTCCCATAGAGACCAAAGAGAATGTCTAATACCTCAGGCCTCTTCCCAGTA

CACAGAAACAAAGGGTCTACCCTGATTGCTGTACCCCAGAGTCTTCTGTGGGCACAG
TGGGACTGGAAGATCA
>Olfr49 LEWES/EiJ
TCTTCTGAGAACAAAGTAAACTTTTAGTCTGAGAGTTGAATGAAGAGGAAAGGGACC
TTCCTAGATCCCTGTTATTGTTGATGGTAATGAATCTCAAGGGAAGGATAGCTCCAG
CCAGCAAAAACAGAGAATCTCAGATGAGTCAAATCTGAGCCATGCATCTTTGCACAT
CTCGAATTCCCATAGAGACCAAAGAGAATGTCTAATACCTCAGGCCTCTTCCCAGTA
CACAGAAACAAAGGGTCTACCCTGATTGCTGTACCCCAGAGTCTTCTGTGGGCACAG
TGGGACTGGAAGATCA
>Olfr49 LP/J
TCTTCTGAGAACAAAGTAAACTTTTAGTCTGAGAGTTGAATGAAGAGGAAAGGGACC
TTCCTAGATCCCTGTTATTGTTGATGGTAATGAATCTCAAGGGAAGGATAGCTCCAG
CCAGCAAAAACAGAGAATCTCAGATGAGTCAAATCTGAGCCATGCATCTTTGCACAT
CTCGAATTCCCATAGAGACCAAAGAGAATGTCTAATACCTCAGGCCTCTTCCCAGTA
CACAGAAACAAAGGGTCTACCCTGATTGCTGTACCCCAGAGTCTTCTGTGGGCACAG
TGGGACTGGAAGATCA
>Olfr49 MOLF/EiJ
TCTTCTGAGAACAAAGTAAACTTTTAGTCTGAGAGTTGAATGAAGAGGAAAGGGACC
TTCCTAGATCCCTGTTATTGTTGATGGTAATGAATCTCAAGGGAAGGATAGCTCCAG
CCAGCAAAAACAGAGAATCTCAGATGAGTCAAATCTGAGCCATGCATCTTTGCACAT
CTCGAATTCCCATAGAGACCAAAGAGAATGTCTAATACCTCAGGCCTCTTCCCAGTA
CACAGAAACAAAGGGTCTACCCTGATTGCTGTACCCCAGAGTCTTCTGTGGGCACAG
TGGGACTGGAAGATCA
>Olfr49 NOD/ShiLtJ
TCTTCTGAGAACAAAGTAAACTTTTAGTCTGAGAGTTGAATGAAGAGGAAAGGGACC
TTCCTAGATCCCTGTTATTGTTGATGGTAATGAATCTCAAGGGAAGGATAGCTCCAG
CCAGCAAAAACAGAGAATCTCAGATGAGTCAAATCTGAGCCATGCATCTTTGCACAT
CTCGAATTCCCATAGAGACCAAAGAGAATGTCTAATACCTCAGGCCTCTTCCCAGTA
CACAGAAACAAAGGGTCTACCCTGATTGCTGTACCCCAGAGTCTTCTGTGGGCACAG
TGGGACTGGAAGATCA
>Olfr49 NZB/B1NJ
TCTTCTGAGAACAAAGTAAACTTTTAGTCTGAGAGTTGAATGAAGAGGAAAGGGACC
TTCCTAGATCCCTGTTATTGTTGATGGTAATGAATCTCAAGGGAAGGATAGCTCCAG
CCAGCAAAAACAGAGAATCTCAGATGAGTCAAATCTGAGCCATGCATCTTTGCACAT
CTCGAATTCCCATAGAGACCAAAGAGAATGTCTAATACCTCAGGCCTCTTCCCAGTA
CACAGAAACAAAGGGTCTACCCTGATTGCTGTACCCCAGAGTCTTCTGTGGGCACAG
TGGGACTGGAAGATCA
>Olfr49 NZO/HlLtJ
TCTTCTGAGAACAAAGTAAACTTTTAGTCTGAGAGTTGAATGAAGAGGAAAGGGACC
TTCCTAGATCCCTGTTATTGTTGATGGTAATGAATCTCAAGGGAAGGATAGCTCCAG
CCAGCAAAAACAGAGAATCTCAGATGAGTCAAATCTGAGCCATGCATCTTTGCACAT
CTCGAATTCCCATAGAGACCAAAGAGAATGTCTAATACCTCAGGCCTCTTCCCAGTA
CACAGAAACAAAGGGTCTACCCTGATTGCTGTACCCCAGAGTCTTCTGTGGGCACAG
TGGGACTGGAAGATCA
>Olfr49 NZW/LacJ
TCTTCTGAGAACAAAGTAAACTTTTAGTCTGAGAGTTGAATGAAGAGGAAAGGGACC
TTCCTAGATCCCTGTTATTGTTGATGGTAATGAATCTCAAGGGAAGGATAGCTCCAG
CCAGCAAAAACAGAGAATCTCAGATGAGTCAAATCTGAGCCATGCATCTTTGCACAT
CTCGAATTCCCATAGAGACCAAAGAGAATGTCTAATACCTCAGGCCTCTTCCCAGTA
CACAGAAACAAAGGGTCTACCCTGATTGCTGTACCCCAGAGTCTTCTGTGGGCACAG
TGGGACTGGAAGATCA

>Olfr49 PWK/PhJ
TCTTCTGAGAACAAAGTAAACTTTTAGTCTGAGAGTTGAATGAAGAGGAAAGGGACC
TTCCTAGATCCCTGTTATTGTTGATGGTAATGAATCTCAAGGGAAGGATAGCTCCAG
CCAGCAAAAACAGAGAATCTCAGATGAGTCAAATCTGAGCCATGCATCTTTGCACAT
CTCGAATTCCCATAGAGACCAAAGAGAATGTCTAATACCTCAGGCCTCTTCCCAGTA
CACAGAAACAAAGGGTCTACCCTGATTGCTGTACCCCAGAGTCTTCTGTGGGCACAG
TGGGACTGGAAGATCA
>Olfr49 RF/J
TCTTCTGAGAACAAAGTAAACTTTTAGTCTGAGAGTTGAATGAAGAGGAAAGGGACC
TTCCTAGATCCCTGTTATTGTTGATGGTAATGAATCTCAAGGGAAGGATAGCTCCAG
CCAGCAAAAACAGAGAATCTCAGATGAGTCAAATCTGAGCCATGCATCTTTGCACAT
CTCGAATTCCCATAGAGACCAAAGAGAATGTCTAATACCTCAGGCCTCTTCCCAGTA
CACAGAAACAAAGGGTCTACCCTGATTGCTGTACCCCAGAGTCTTCTGTGGGCACAG
TGGGACTGGAAGATCA
>Olfr49 SEA/GnJ
TCTTCTGAGAACAAAGTAAACTTTTAGTCTGAGAGTTGAATGAAGAGGAAAGGGACC
TTCCTAGATCCCTGTTATTGTTGATGGTAATGAATCTCAAGGGAAGGATAGCTCCAG
CCAGCAAAAACAGAGAATCTCAGATGAGTCAAATCTGAGCCATGCATCTTTGCACAT
CTCGAATTCCCATAGAGACCAAAGAGAATGTCTAATACCTCAGGCCTCTTCCCAGTA
CACAGAAACAAAGGGTCTACCCTGATTGCTGTACCCCAGAGTCTTCTGTGGGCACAG
TGGGACTGGAAGATCA
>Olfr49 SPRET/EiJ
TCTTCTGAGAACAAAGTAAACTTTTAGTCTGAGAGTTGAATGAAGAGGAAAGGGACC
TTCCTAGATCCCTGTTATTGTTGATGGTAATGAATCTCAAGGGAAGGATAGCTCCAG
CCAGCAAAAACAGAGAATCTCAGATGAGTCAAATCTGAGCCATGCATCTTTGCACAT
CTCGAATTCCCATAGAGACCAAAAAGAATGTCTAATACCTCAGGCCTCTTCCCAGTA
CACAGAAACAAAGGGTCTACCCTGATTGCTGTACCCCTTCTGTGGGCACAGTGGGAC
TGGAAGATCA
>Olfr49 ST/bJ
TCTTCTGAGAACAAAGTAAACTTTTAGTCTGAGAGTTGAATGAAGAGGAAAGGGACC
TTCCTAGATCCCTGTTATTGTTGATGGTAATGAATCTCAAGGGAAGGATAGCTCCAG
CCAGCAAAAACAGAGAATCTCAGATGAGTCAAATCTGAGCCATGCATCTTTGCACAT
CTCGAATTCCCATAGAGACCAAAGAGAATGTCTAATACCTCAGGCCTCTTCCCAGTA
CACAGAAACAAAGGGTCTACCCTGATTGCTGTACCCCAGAGTCTTCTGTGGGCACAG
TGGGACTGGAAGATCA
>Olfr49 WSB/EiJ
TCTTCTGAGAACAAAGTAAACTTTTAGTCTGAGAGTTGAATGAAGAGGAAAGGGACC
TTCCTAGATCCCTGTTATTGTTGATGGTAATGAATCTCAAGGGAAGGATAGCTCCAG
CCAGCAAAAACAGAGAATCTCAGATGAGTCAAATCTGAGCCATGCATCTTTGCACAT
CTCGAATTCCCATAGAGACCAAAGAGAATGTCTAATACCTCAGGCCTCTTCCCAGTA
CACAGAAACAAAGGGTCTACCCTGATTGCTGTACCCCAGAGTCTTCTGTGGGCACAG
TGGGACTGGAAGATCA
>Olfr49 ZALENDE/EiJ
TCTTCTGAGAACAAAGTAAACTTTTAGTCTGAGAGTTGAATGAAGAGGAAAGGGACC
TTCCTAGATCCCTGTTATTGTTGATGGTAATGAATCTCAAGGGAAGGATAGCTCCAG
CCAGCAAAAACAGAGAATCTCAGATGAGTCAAATCTGAGCCATGCATCTTTGCACAT
CTCGAATTCCCATAGAGACCAAAGAGAATGTCTAATACCTCAGGCCTCTTCCCAGTA
CACAGAAACAAAGGGTCTACCCTGATTGCTGTACCCCAGAGTCTTCTGTGGGCACAG
TGGGACTGGAAGATCA

>Olfr266 C57BL/6J

TGTTTGACATCATTGGGGAGGTTACAACTTTACTCACAAAGGGACTAAACTTTACAC
AATGGAATAGATTGAGAAACAGGGTCAATATTCCACTGGCCTTCCAGATTCACAGTC
CTTAGTTTTTAATGAATGTACAGGGAGGTGTATTTCTGGATAAAAGCAAGATTCCAC
ACATCTAATGTTATTATTTAAATCCAGGGTAAGTGGACATGTTTTATTCTATGTGGT
CTGGCTCTGCAAATTAGGAATGGACCTCTCCAAGTCTCTGATGGGCAAAGCGGATCT
GTTCTGGTAATATACT
>Olfr266 Wild_consensus
TGTTTGACATCATTGGGGAGGTTACAACTTTACTCACAAAGGGACTAAACTTTACAC
AATGGAATAGATTGAGAAACAGGGTCAATATTCCACTGGCCTTCCAGATTCACAGTC
CTTAGTTTTTAATGAATGTACAGGGAGGTGTATTTCTGGATAAAAGCAAGATTCCAC
ACATCTAATGTTATTATTTAAATCCAGGGTAAGTGGACATGTTTTATTCTATGTGGT
CTGGCTCTGCAAATTAGGAATGGACCTCTCCAAGTCTCTGATGGGCAAAGCGGATCT
GTTCTGGTAATATACT
>Olfr266 France
TGTTTGACATCATTGGGGAGGTTACAACTTTACTCACAAAGGGACTAAACTTTACAC
AATGGAATAGATTGAGAAACAGGGTCAATATTCCACTGGCCTTCCAGATTCACAGTC
CTTAGTTTTTAATGAATGTACAGGGAGGTGTATTTCTGGATAAAAGCAAGATTCCAC
ACATCTAATGTTATTATTTAAATCCAGGGTAAGTGGACATGTTTTATTCTATGTGGT
CTGGCTCTGCAAATTAGGAATGGACCTCTCCAAGTCTCTGATGGGCAAAGCGGATCT
GTTCTGGTAATATACT
>Olfr266 Germany
TGTTTGACATCATTGGGGAGGTTACAACTTTACTCACAAAGGGACTAAACTTTACAC
AATGGAATAGATTGAGAAACAGGGTCAATATTCCACTGGCCTTCCAGATTCACAGTC
CTTAGTTTTTAATGAATGTACAGGGAGGTGTATTTCTGGATAAAAGCAAGATTCCAC
ACATCTAATGTTATTATTTAAATCCAGGGTAAGTGGACATGTTTTATTCTATGTGGC
CTGGCTCTGCAAAGTAGGAATGGACCTCTCCAAGTCTCTGATGGGCAAAGCGGATCT
GTTCTGGTAATATACT
>Olfr266 Iran
TGTTTGACATCATTGGGGAGGTTACAACTTTACTCACAAAGGGACTAAACTTTACAC
AATGGAATAGATTGAGAAACAGGGTCAATATTCCACTGGCCTTCCAGATTCACAGTC
CTTAGTTTTTAATGAATGTACAGGGAGGTGTATTTCTGGATAAAAGCAAGATTCCAC
ACATCTAATGTTATTATTTAAATCCAGGGTAAGTGGACATGTTTTATTCTATGTGGT
CTGGCTCTGCAAATTAGGAATGGACCTCTCCAAGTCTCTGATGGGCAAAGCGGATCT
GTTCTGGTAATATACT
>Olfr266 129P2/OlaHsd
TGTTTGACATCATTGGGGAGGTTACAACTTTACTCACAAAGGGACTAAACTTTACAC
AATGGAATAGATTGAGAAACAGGGTCAATATTCCACTGGCCTTCCAGATTCACAGTC
CTTAGTTTTTAATGAATGTACAGGGAGGTGTATTTCTGGATAAAAGCAAGATTCCAC
ACATCTAATGTTATTATTTAAATCCAGGGTAAGTGGACATGTTTTATTCTATGTGGT
CTGGCTCTGCAAATTAGGAATGGACCTCTCCAAGTCTCTGATGGGCAAAGCGGATCT
GTTCTGGTAATATACT
>Olfr266 129S1/SvImJ
TGTTTGACATCATTGGGGAGGTTACAACTTTACTCACAAAGGGACTAAACTTTACAC
AATGGAATAGATTGAGAAACAGGGTCAATATTCCACTGGCCTTCCAGATTCACAGTC
CTTAGTTTTTAATGAATGTACAGGGAGGTGTATTTCTGGATAAAAGCAAGATTCCAC
ACATCTAATGTTATTATTTAAATCCAGGGTAAGTGGACATGTTTTATTCTATGTGGT
CTGGCTCTGCAAATTAGGAATGGACCTCTCCAAGTCTCTGATGGGCAAAGCGGATCT
GTTCTGGTAATATACT
>Olfr266 129S5SvEvBrd
TGTTTGACATCATTGGGGAGGTTACAACTTTACTCACAAAGGGACTAAACTTTACAC
AATGGAATAGATTGAGAAACAGGGTCAATATTCCACTGGCCTTCCAGATTCACAGTC

CTTAGTTTTTAATGAATGTACAGGGAGGTGTATTTCTGGATAAAAGCAAGATTCCAC
ACATCTAATGTTATTATTTAAATCCAGGGTAAGTGGACATGTTTTATTCTATGTGGT
CTGGCTCTGCAAATTAGGAATGGACCTCTCCAAGTCTCTGATGGGCAAAGCGGATCT
GTTCTGGTAATATACT
>Olfr266 A/J
TGTTTGACATCATTGGGGAGGTTACAACTTTACTCACAAAGGGACTAAACTTTACAC
AATGGAATAGATTGAGAAACAGGGTCAATATTCCACTGGCCTTCCAGATTCACAGTC
CTTAGTTTTTAATGAATGTACAGGGAGGTGTATTTCTGGATAAAAGCAAGATTCCAC
ACATCTAATGTTATTATTTAAATCCAGGGTAAGTGGACATGTTTTATTCTATGTGGT
CTGGCTCTGCAAATTAGGAATGGACCTCTCCAAGTCTCTGATGGGCAAAGCGGATCT
GTTCTGGTAATATACT
>Olfr266 AKR/J
TGTTTGACATCATTGGGGAGGTTACAACTTTACTCACAAAGGGACTAAACTTTACAC
AATGGAATAGATTGAGAAACAGGGTCAATATTCCACTGGCCTTCCAGATTCACAGTC
CTTAGTTTTTAATGAATGTACAGGGAGGTGTATTTCTGGATAAAAGCAAGATTCCAC
ACATCTAATGTTATTATTTAAATCCAGGGTAAGTGGACATGTTTTATTCTATGTGGT
CTGGCTCTGCAAATTAGGAATGGACCTCTCCAAGTCTCTGATGGGCAAAGCGGATCT
GTTCTGGTAATATACT
>Olfr266 BALB/cJ
TGTTTGACATCATTGGGGAGGTTACAACTTTACTCACAAAGGGACTAAACTTTACAC
AATGGAATAGATTGAGAAACAGGGTCAATATTCCACTGGCCTTCCAGATTCACAGTC
CTTAGTTTTTAATGAATGTACAGGGAGGTGTATTTCTGGATAAAAGCAAGATTCCAC
ACATCTAATGTTATTATTTAAATCCAGGGTAAGTGGACATGTTTTATTCTATGTGGT
CTGGCTCTGCAAATTAGGAATGGACCTCTCCAAGTCTCTGATGGGCAAAGCGGATCT
GTTCTGGTAATATACT
>Olfr266 BTBR/T_+_Itpr3tf/J
TGTTTGACATCATTGGGGAGGTTACAACTTTACTCACAAAGGGACTAAACTTTACAC
AATGGAATAGATTGAGAAACAGGGTCAATATTCCACTGGCCTTCCAGATTCACAGTC
CTTAGTTTTTAATGAATGTACAGGGAGGTGTATTTCTGGATAAAAGCAAGATTCCAC
ACATCTAATGTTATTATTTAAATCCAGGGTAAGTGGACATGTTTTATTCTATGTGGT
CTGGCTCTGCAAATTAGGAATGGACCTCTCCAAGTCTCTGATGGGCAAAGCGGATCT
GTTCTGGTAATATACT
>Olfr266 BUB/BnJ
TGTTTGACATCATTGGGGAGGTTACAACTTTACTCACAAAGGGACTAAACTTTACAC
AATGGAATAGATTGAGAAACAGGGTCAATATTCCACTGGCCTTCCAGATTCACAGTC
CTTAGTTTTTAATGAATGTACAGGGAGGTGTATTTCTGGATAAAAGCAAGATTCCAC
ACATCTAATGTTATTATTTAAATCCAGGGTAAGTGGACATGTTTTATTCTATGTGGT
CTGGCTCTGCAAATTAGGAATGGACCTCTCCAAGTCTCTGATGGGCAAAGCGGATCT
GTTCTGGTAATATACT
>Olfr266 C3H/HeH
TGTTTGACATCATTGGGGAGGTTACAACTTTACTCACAAAGGGACTAAACTTTACAC
AATGGAATAGATTGAGAAACAGGGTCAATATTCCACTGGCCTTCCAGATTCACAGTC
CTTAGTTTTTAATGAATGTACAGGGAGGTGTATTTCTGGATAAAAGCAAGATTCCAC
ACATCTAATGTTATTATTTAAATCCAGGGTAAGTGGACATGTTTTATTCTATGTGGT
CTGGCTCTGCAAATTAGGAATGGACCTCTCCAAGTCTCTGATGGGCAAAGCGGATCT
GTTCTGGTAATATACT
>Olfr266 C3H/HeJ
TGTTTGACATCATTGGGGAGGTTACAACTTTACTCACAAAGGGACTAAACTTTACAC
AATGGAATAGATTGAGAAACAGGGTCAATATTCCACTGGCCTTCCAGATTCACAGTC
CTTAGTTTTTAATGAATGTACAGGGAGGTGTATTTCTGGATAAAAGCAAGATTCCAC
ACATCTAATGTTATTATTTAAATCCAGGGTAAGTGGACATGTTTTATTCTATGTGGT

CTGGCTCTGCAAATTAGGAATGGACCTCTCCAAGTCTCTGATGGGCAAAGCGGATCT
GTTCTGGTAATATACT
>Olfr266 C57BL/10J
TGTTTGACATCATTGGGGAGGTTACAACTTTACTCACAAAGGGACTAAACTTTACAC
AATGGAATAGATTGAGAAACAGGGTCAATATTCCACTGGCCTTCCAGATTCACAGTC
CTTAGTTTTTAATGAATGTACAGGGAGGTGTATTTCTGGATAAAAGCAAGATTCCAC
ACATCTAATGTTATTATTTAAATCCAGGGTAAGTGGACATGTTTTATTCTATGTGGT
CTGGCTCTGCAAATTAGGAATGGACCTCTCCAAGTCTCTGATGGGCAAAGCGGATCT
GTTCTGGTAATATACT
>Olfr266 C57BL/6NJ
TGTTTGACATCATTGGGGAGGTTACAACTTTACTCACAAAGGGACTAAACTTTACAC
AATGGAATAGATTGAGAAACAGGGTCAATATTCCACTGGCCTTCCAGATTCACAGTC
CTTAGTTTTTAATGAATGTACAGGGAGGTGTATTTCTGGATAAAAGCAAGATTCCAC
ACATCTAATGTTATTATTTAAATCCAGGGTAAGTGGACATGTTTTATTCTATGTGGT
CTGGCTCTGCAAATTAGGAATGGACCTCTCCAAGTCTCTGATGGGCAAAGCGGATCT
GTTCTGGTAATATACT
>Olfr266 C57BR/cdJ
TGTTTGACATCATTGGGGAGGTTACAACTTTACTCACAAAGGGACTAAACTTTACAC
AATGGAATAGATTGAGAAACAGGGTCAATATTCCACTGGCCTTCCAGATTCACAGTC
CTTAGTTTTTAATGAATGTACAGGGAGGTGTATTTCTGGATAAAAGCAAGATTCCAC
ACATCTAATGTTATTATTTAAATCCAGGGTAAGTGGACATGTTTTATTCTATGTGGT
CTGGCTCTGCAAATTAGGAATGGACCTCTCCAAGTCTCTGATGGGCAAAGCGGATCT
GTTCTGGTAATATACT
>Olfr266 C57L/J
TGTTTGACATCATTGGGGAGGTTACAACTTTACTCACAAAGGGACTAAACTTTACAC
AATGGAATAGATTGAGAAACAGGGTCAATATTCCACTGGCCTTCCAGATTCACAGTC
CTTAGTTTTTAATGAATGTACAGGGAGGTGTATTTCTGGATAAAAGCAAGATTCCAC
ACATCTAATGTTATTATTTAAATCCAGGGTAAGTGGACATGTTTTATTCTATGTGGT
CTGGCTCTGCAAATTAGGAATGGACCTCTCCAAGTCTCTGATGGGCAAAGCGGATCT
GTTCTGGTAATATACT
>Olfr266 C58/J
TGTTTGACATCATTGGGGAGGTTACAACTTTACTCACAAAGGGACTAAACTTTACAC
AATGGAATAGATTGAGAAACAGGGTCAATATTCCACTGGCCTTCCAGATTCACAGTC
CTTAGTTTTTAATGAATGTACAGGGAGGTGTATTTCTGGATAAAAGCAAGATTCCAC
ACATCTAATGTTATTATTTAAATCCAGGGTAAGTGGACATGTTTTATTCTATGTGGT
CTGGCTCTGCAAATTAGGAATGGACCTCTCCAAGTCTCTGATGGGCAAAGCGGATCT
GTTCTGGTAATATACT
>Olfr266 CAST/EiJ
TGTTTGACATCATTGGGGAGGTTACAACTTTACTCACAAAGGGACTAAACTTTACAC
AATGGAATAGATTGAGAAACAGGGTCAATATTCCACTGGCCTTCCAGATTCACAGTC
CTTAGTTTTTAATGAATGTACAGGGAGGTGTATTTCTGGATAAAAGCAAGATTCCAC
ACATCTAATGTTATTATTTAAATCCAGGGTAAGTGGACATGTTTTATTCTATGTGGT
CTGGCTCTGCAAATTAGGAATGGACCTCTCCAAGTCTCTGATGGGCAAAGCGGATCT
GTTCTGGTAATATACT
>Olfr266 CBA/J
TGTTTGACATCATTGGGGAGGTTACAACTTTACTCACAAAGGGACTAAACTTTACAC
AATGGAATAGATTGAGAAACAGGGTCAATATTCCACTGGCCTTCCAGATTCACAGTC
CTTAGTTTTTAATGAATGTACAGGGAGGTGTATTTCTGGATAAAAGCAAGATTCCAC
ACATCTAATGTTATTATTTAAATCCAGGGTAAGTGGACATGTTTTATTCTATGTGGT
CTGGCTCTGCAAATTAGGAATGGACCTCTCCAAGTCTCTGATGGGCAAAGCGGATCT
GTTCTGGTAATATACT

>Olfr266 DBA/1J
TGTTTGACATCATTGGGGAGGTTACAACTTTACTCACAAAGGGACTAAACTTTACAC
AATGGAATAGATTGAGAAACAGGGTCAATATTCCACTGGCCTTCCAGATTCACAGTC
CTTAGTTTTTAATGAATGTACAGGGAGGTGTATTTCTGGATAAAAGCAAGATTCCAC
ACATCTAATGTTATTATTTAAATCCAGGGTAAGTGGACATGTTTTATTCTATGTGGT
CTGGCTCTGCAAATTAGGAATGGACCTCTCCAAGTCTCTGATGGGCAAAGCGGATCT
GTTCTGGTAATATACT
>Olfr266 DBA/2J
TGTTTGACATCATTGGGGAGGTTACAACTTTACTCACAAAGGGACTAAACTTTACAC
AATGGAATAGATTGAGAAACAGGGTCAATATTCCACTGGCCTTCCAGATTCACAGTC
CTTAGTTTTTAATGAATGTACAGGGAGGTGTATTTCTGGATAAAAGCAAGATTCCAC
ACATCTAATGTTATTATTTAAATCCAGGGTAAGTGGACATGTTTTATTCTATGTGGT
CTGGCTCTGCAAATTAGGAATGGACCTCTCCAAGTCTCTGATGGGCAAAGCGGATCT
GTTCTGGTAATATACT
>Olfr266 FVB/NJ
TGTTTGACATCATTGGGGAGGTTACAACTTTACTCACAAAGGGACTAAACTTTACAC
AATGGAATAGATTGAGAAACAGGGTCAATATTCCACTGGCCTTCCAGATTCACAGTC
CTTAGTTTTTAATGAATGTACAGGGAGGTGTATTTCTGGATAAAAGCAAGATTCCAC
ACATCTAATGTTATTATTTAAATCCAGGGTAAGTGGACATGTTTTATTCTATGTGGT
CTGGCTCTGCAAATTAGGAATGGACCTCTCCAAGTCTCTGATGGGCAAAGCGGATCT
GTTCTGGTAATATACT
>Olfr266 I/LnJ
TGTTTGACATCATTGGGGAGGTTACAACTTTACTCACAAAGGGACTAAACTTTACAC
AATGGAATAGATTGAGAAACAGGGTCAATATTCCACTGGCCTTCCAGATTCACAGTC
CTTAGTTTTTAATGAATGTACAGGGAGGTGTATTTCTGGATAAAAGCAAGATTCCAC
ACATCTAATGTTATTATTTAAATCCAGGGTAAGTGGACATGTTTTATTCTATGTGGT
CTGGCTCTGCAAATTAGGAATGGACCTCTCCAAGTCTCTGATGGGCAAAGCGGATCT
GTTCTGGTAATATACT
>Olfr266 KK/HiJ
TGTTTGACATCATTGGGGAGGTTACAACTTTACTCACAAAGGGACTAAACTTTACAC
AATGGAATAGATTGAGAAACAGGGTCAATATTCCACTGGCCTTCCAGATTCACAGTC
CTTAGTTTTTAATGAATGTACAGGGAGGTGTATTTCTGGATAAAAGCAAGATTCCAC
ACATCTAATGTTATTATTTAAATCCAGGGTAAGTGGACATGTTTTATTCTATGTGGT
CTGGCTCTGCAAATTAGGAATGGACCTCTCCAAGTCTCTGATGGGCAAAGCGGATCT
GTTCTGGTAATATACT
>Olfr266 LEWES/EiJ
TGTTTGACATCATTGGGGAGGTTACAACTTTACTCACAAAGGGACTAAACTTTACAC
AATGGAATAGATTGAGAAACAGGGTCAATATTCCACTGGCCTTCCAGATTCACAGTC
CTTAGTTTTTAATGAATGTACAGGGAGGTGTATTTCTGGATAAAAGCAAGATTCCAC
ACATCTAATGTTATTATTTAAATCCAGGGTAAGTGGACATGTTTTATTCTATGTGGC
CTGGCTCTGCAAAGTAGGAATGGACCTCTCCAAGTCTCTGATGGGCAAAGCGGATCT
GTTCTGGTAATATACT
>Olfr266 LP/J
TGTTTGACATCATTGGGGAGGTTACAACTTTACTCACAAAGGGACTAAACTTTACAC
AATGGAATAGATTGAGAAACAGGGTCAATATTCCACTGGCCTTCCAGATTCACAGTC
CTTAGTTTTTAATGAATGTACAGGGAGGTGTATTTCTGGATAAAAGCAAGATTCCAC
ACATCTAATGTTATTATTTAAATCCAGGGTAAGTGGACATGTTTTATTCTATGTGGT
CTGGCTCTGCAAATTAGGAATGGACCTCTCCAAGTCTCTGATGGGCAAAGCGGATCT
GTTCTGGTAATATACT
>Olfr266 MOLF/EiJ
TGTTTGACATCATTGGGGAGGTTACAACTTTACTCACAAAGGGACTAAACTTTACAC

AATGGAATAGATTGAGAAACAGGGTCAATATTCCACTGGCCTTCCAGATTCACAGTC
CTTAGTTTTTAATGAATGTACAGGGAGGTGTATTTCTGGATAAAAGCAAGATTCCAC
ACATCTAATGTTATTATTTGAATCCAGGGTAAGTGGACATGTTTTATTCTATGTGGC
CTGGCTCTGCAAAGTAGGAATGAACCTCTCCAAGTCTCTGATGGGCAAAGCGGATCT
GTTCTGGTAATATACT
>Olfr266 NOD/ShiLtJ
TGTTTGACATCATTGGGGAGGTTACAACTTTACTCACAAAGGGACTAAACTTTACAC
AATGGAATAGATTGAGAAACAGGGTCAATATTCCACTGGCCTTCCAGATTCACAGTC
CTTAGTTTTTAATGAATGTACAGGGAGGTGTATTTCTGGATAAAAGCAAGATTCCAC
ACATCTAATGTTATTATTTAAATCCAGGGTAAGTGGACATGTTTTATTCTATGTGGT
CTGGCTCTGCAAATTAGGAATGGACCTCTCCAAGTCTCTGATGGGCAAAGCGGATCT
GTTCTGGTAATATACT
>Olfr266 NZB/B1NJ
TGTTTGACATCATTGGGGAGGTTACAACTTTACTCACAAAGGGACTAAACTTTACAC
AATGGAATAGATTGAGAAACAGGGTCAATATTCCACTGGCCTTCCAGATTCACAGTC
CTTAGTTTTTAATGAATGTACAGGGAGGTGTATTTCTGGATAAAAGCAAGATTCCAC
ACATCTAATGTTATTATTTAAATCCAGGGTAAGTGGACATGTTTTATTCTATGTGGT
CTGGCTCTGCAAAGTAGGAATGGACCTCTCCAAGTCTCTGATGGGCAAAGCGGATCT
GTTCTGGTAATATACT
>Olfr266 NZO/HlLtJ
TGTTTGACATCATTGGGGAGGTTACAACTTTACTCACAAAGGGACTAAACTTTACAC
AATGGAATAGATTGAGAAACAGGGTCAATATTCCACTGGCCTTCCAGATTCACAGTC
CTTAGTTTTTAATGAATGTACAGGGAGGTGTATTTCTGGATAAAAGCAAGATTCCAC
ACATCTAATGTTATTATTTAAATCCAGGGTAAGTGGACATGTTTTATTCTATGTGGT
CTGGCTCTGCAAAGTAGGAATGGACCTCTCCAAGTCTCTGATGGGCAAAGCGGATCT
GTTCTGGTAATATACT
>Olfr266 NZW/LacJ
TGTTTGACATCATTGGGGAGGTTACAACTTTACTCACAAAGGGACTAAACTTTACAC
AATGGAATAGATTGAGAAACAGGGTCAATATTCCACTGGCCTTCCAGATTCACAGTC
CTTAGTTTTTAATGAATGTACAGGGAGGTGTATTTCTGGATAAAAGCAAGATTCCAC
ACATCTAATGTTATTATTTAAATCCAGGGTAAGTGGACATGTTTTATTCTATGTGGT
CTGGCTCTGCAAAGTAGGAATGGACCTCTCCAAGTCTCTGATGGGCAAAGCGGATCT
GTTCTGGTAATATACT
>Olfr266 PWK/PhJ
TGTTTGACATCATTGGGGAGGTTACAACTTTACTCACAAAGGGACTAAACTTTACAC
AATGGAATAGATTGAGAAACAGGGTCAATATTCCACTGGCCTTCCAGATTCACAGTC
CTTAGTTTTTAATGAATGTACAGGGAGGTGTATTTCTGGATAAAAGCAAGATTCCAC
ACATCTAATGTTATTATTTAAATCCAGGGTAAGTGGACATGTTTTATTCTATGTGGT
CTGGCTCTGCAAAGTAGGAATGGACCTCTCCAAGTCTCTGATGGGCAAAGCGGATCT
GTTCTGGTAATATACT
>Olfr266 RF/J
TGTTTGACATCATTGGGGAGGTTACAACTTTACTCACAAAGGGACTAAACTTTACAC
AATGGAATAGATTGAGAAACAGGGTCAATATTCCACTGGCCTTCCAGATTCACAGTC
CTTAGTTTTTAATGAATGTACAGGGAGGTGTATTTCTGGATAAAAGCAAGATTCCAC
ACATCTAATGTTATTATTTAAATCCAGGGTAAGTGGACATGTTTTATTCTATGTGGT
CTGGCTCTGCAAATTAGGAATGGACCTCTCCAAGTCTCTGATGGGCAAAGCGGATCT
GTTCTGGTAATATACT
>Olfr266 SEA/GnJ
TGTTTGACATCATTGGGGAGGTTACAACTTTACTCACAAAGGGACTAAACTTTACAC
AATGGAATAGATTGAGAAACAGGGTCAATATTCCACTGGCCTTCCAGATTCACAGTC
CTTAGTTTTTAATGAATGTACAGGGAGGTGTATTTCTGGATAAAAGCAAGATTCCAC

ACATCTAATGTTATTATTTAAATCCAGGGTAAGTGGACATGTTTTATTCTATGTGGT
CTGGCTCTGCAAATTAGGAATGGACCTCTCCAAGTCTCTGATGGGCAAAGCGGATCT
GTTCTGGTAATATACT
>Olfr266 SPRET/EiJ
TGTTTGACATCATTGGGGAGGTTACAACTTTACTCACAAAGGGACTAAACTTTACAC
AATGGAATAGATTCAGAAACAGGGTCAATATTCCACTGGCCTTCCAGATTCACAGCC
CTTAGTTTTTAATGAATGTACAGGGAGGTGTATTTCTGGATAAAAGCAAGATTCCAC
ACATTTAATGTCATTATTTAAATCCAGGGTAAGTAGACATGTTTTATTCTATCTGGC
CTGGCTCTGCAAAGTAGGAATGGACCTCTCCAAGTCTCTGATGGGAAAAGCTGATCT
GTTCTGGTAATATACT
>Olfr266 ST/bJ
TGTTTGACATCATTGGGGAGGTTACAACTTTACTCACAAAGGGACTAAACTTTACAC
AATGGAATAGATTGAGAAACAGGGTCAATATTCCACTGGCCTTCCAGATTCACAGTC
CTTAGTTTTTAATGAATGTACAGGGAGGTGTATTTCTGGATAAAAGCAAGATTCCAC
ACATCTAATGTTATTATTTAAATCCAGGGTAAGTGGACATGTTTTATTCTATGTGGT
CTGGCTCTGCAAATTAGGAATGGACCTCTCCAAGTCTCTGATGGGCAAAGCGGATCT
GTTCTGGTAATATACT
>Olfr266 WSB/EiJ
TGTTTGACATCATTGGGGAGGTTACAACTTTACTCACAAAGGGACTAAACTTTACAC
AATGGAATAGATTGAGAAACAGGGTCAATATTCCACTGGCCTTCCAGATTCACAGTC
CTTAGTTTTTAATGAATGTACAGGGAGGTGTATTTCTGGATAAAAGCAAGATTCCAC
ACATCTAATGTTATTATTTAAATCCAGGGTAAGTGGACATGTTTTATTCTATGTGGT
CTGGCTCTGCAAATTAGGAATGGACCTCTCCAAGTCTCTGATGGGCAAAGCGGATCT
GTTCTGGTAATATACT
>Olfr266 ZALENDE/EiJ
TGTTTGACATCATTGGGGAGGTTACAACTTTACTCACAAAGGGACTAAACTTTACAC
AATGGAATAGATTGAGAAACAGGGTCAATATTCCACTGGCCTTCCAGATTCACAGTC
CTTAGTTTTTAATGAATGTACAGGGAGGTGTATTTCTGGATAAAAGCAAGATTCCAC
ACATCTAATGTTATTATTTAAATCCAGGGTAAGTGGACATGTTTTATTCTATGTGGT
CTGGCTCTGCAAATTAGGAATGGACCTCTCCAAGTCTCTGATGGGCAAAGCGGATCT
GTTCTGGTAATATACT

>Olfr267 C57BL/6J
GGATCTTTGGGGCATTATCCCTGAAGGATGAGAAGTTGCAAACCTCTGTGTAATGCA
GCTGCTAAACTTTAAATTGTTCAGGGACAATGAGTATTCCCCCAAAACAAGTGATTT
AAATGTATATAGTACTGCAGTTTGGGATCCTTTGGTAATTTTGGTTATGTTTGTAAC
ATCTGGTCATTTTGAAAGAAATGCATTGCATAATTGATTTTTCTATAGCATTTCAGA
TTTTAAATGTAGATATATTCATTCTTCTTTACATATAACCAACCATAACTAACTTGT
GGAACATATCATAAAA
>Olfr267 Wild_consensus
GGATCTTTGGGGCATTATCCCTGAAGGATGAGAAGTTGCAAACCTCTGTGTAATGCA
GCTGCTAAACTTTAAATTGTTCAGGGACAATGAGTATTCCCCCAAAACAAGTGATTT
AAATGTATATAGTACTGCAGTTTGGGATCCTTTGGTAATTTTGGTTATGTTTGTAAC
ATCTGGTCATTTTGAAAGAAATGCATTGCATAATTGATTTTTCTATAGCATTTCAGA
TTTTAAATGTAGATATATTCATTCTTCTTTACATATAACCAACCATAACTAACTTGT
GGAACATATCATAAAA
>Olfr267 France
GGATCTTTGGGGCATTATCCCTGAAGGATGAGAAGTTGCAAACCTCTGTGTAATGCA
GCTGCTAAACTTTAAATTGTTCAGGGACAATGAGTATTCCCCCAACAACAAGTGATT
TAAATGTATATAGTACTGCAGTTTGGGATCCTTTGGTAATTTTGGTTATGTTTGTAA
CATCTGGTCATTTTGAAAGAAATGCATTGCATAATTGATTTTTCTATAGCATTTCAG

ATTTTAAATGTAGATATATTCATTCTTCTTTACATATAACCAACCATAACTAACTTG
TGGAACATATCATAAAA
>Olfr267 Germany
GGATCTTTGGGGCATTATCCCTGAAGGATGAGAAGTTGCAAACCTCTGTGTAATGCA
GCTGCTAAACTTTAAATTGTTCAGGGACAATGAGTATTCCCCCAAAACAAGTGATTT
AAATGTATATAGTACTGCAGTTTGGGATCCTTTGGTAATTTTGGTTATGTTTGTAAC
ATCTGGTCATTTTGAAAGAAATGCATTGCATAATTGATTTTTCTATAGCATTTCAGA
TTTTAAATGTAGATATATTCATTCTTCTTTACATATAACCAACCATAACTAACTTGT
GGAACATATCATAAAA
>Olfr267 Iran
GGATCTTTGGGGCATTATCCCTGAAGGATGAGAAGTTGCAAACCTCTGTGTAATGCA
GCTGCTAAACTTTAAATTGTTCAGGGACAATGAGTATTCCCCCAAAACAAGTGATTT
AAATGTATATAGTACTGCAGTTTGGGATCCTTTGGTAATTTTGGTTATGTTTGTAAC
ATCTGGTCATTTTGAAAGAAATGCATTGCATAATTGATTTTTCTATAGCATTTCAGA
TTTTAAATGTAGATATATTCATTCTTCTTTACATATAACCAACCATAACTAACTTGT
GGAACATATCATAAAA
>Olfr267 129P2/OlaHsd
GGATCTTTGGGGCATTATCCCTGAAGGATGAGAAGTTGCAAACCTCTGTGTAATGCA
GCTGCTAAACTTTAAATTGTTCAGGGACAATGAGTATTCCCCCAAAACAAGTGATTT
AAATGTATATAGTACTGCAGTTTGGGATCCTTTGGTAATTTTGGTTATGTTTGTAAC
ATCTGGTCATTTTGAAAGAAATGCATTGCATAATTGATTTTTCTATAGCATTTCAGA
TTTTAAATGTAGATATATTCATTCTTCTTTACATATAACCAACCATAACTAACTTGT
GGAACATATCATAAAA
>Olfr267 129S1/SvImJ
GGATCTTTGGGGCATTATCCCTGAAGGATGAGAAGTTGCAAACCTCTGTGTAATGCA
GCTGCTAAACTTTAAATTGTTCAGGGACAATGAGTATTCCCCCAAAACAAGTGATTT
AAATGTATATAGTACTGCAGTTTGGGATCCTTTGGTAATTTTGGTTATGTTTGTAAC
ATCTGGTCATTTTGAAAGAAATGCATTGCATAATTGATTTTTCTATAGCATTTCAGA
TTTTAAATGTAGATATATTCATTCTTCTTTACATATAACCAACCATAACTAACTTGT
GGAACATATCATAAAA
>Olfr267 129S5SvEvBrd
GGATCTTTGGGGCATTATCCCTGAAGGATGAGAAGTTGCAAACCTCTGTGTAATGCA
GCTGCTAAACTTTAAATTGTTCAGGGACAATGAGTATTCCCCCAAAACAAGTGATTT
AAATGTATATAGTACTGCAGTTTGGGATCCTTTGGTAATTTTGGTTATGTTTGTAAC
ATCTGGTCATTTTGAAAGAAATGCATTGCATAATTGATTTTTCTATAGCATTTCAGA
TTTTAAATGTAGATATATTCATTCTTCTTTACATATAACCAACCATAACTAACTTGT
GGAACATATCATAAAA
>Olfr267 A/J
GGATCTTTGGGGCATTATCCCTGAAGGATGAGAAGTTGCAAACCTCTGTGTAATGCA
GCTGCTAAACTTTAAATTGTTCAGGGACAATGAGTATTCCCCCAAAACAAGTGATTT
AAATGTATATAGTATTGCAGTTTGGGATCCTTTGGTAATTTTGGTTATGTTTGTAAC
ATCTGGTCATTTTGAAAGAAATGCATTGCATAATTGATTTTTCTATAGCATTTCAGA
TTTTAAATGTAGATATATTCATTCTTCTTTACATATAACCAACCATAACTAACTTGT
GGAACATATCATAAAA
>Olfr267 AKR/J
GGATCTTTGGGGCATTATCCCTGAAGGATGAGAAGTTGCAAACCTCTGTGTAATGCA
GCTGCTAAACTTTAAATTGTTCAGGGACAATGAGTATTCCCCCAAAACAAGTGATTT
AAATGTATATAGTATTGCAGTTTGGGATCCTTTGGTAATTTTGGTTATGTTTGTAAC
ATCTGGTCATTTTGAAAGAAATGCATTGCATAATTGATTTTTCTATAGCATTTCAGA
TTTTAAATGTAGATATATTCATTCTTCTTTACATATAACCAACCATAACTAACTTGT
GGAACATATCATAAAA

>Olfr267 BALB/cJ
GGATCTTTGGGGCATTATCCCTGAAGGATGAGAAGTTGCAAACCTCTGTGTAATGCA
GCTGCTAAACTTTAAATTGTTCAGGGACAATGAGTATTCCCCCAAAACAAGTGATTT
AAATGTATATAGTATTGCAGTTTGGGATCCTTTGGTAATTTTGGTTATGTTTGTAAC
ATCTGGTCATTTTGAAAGAAATGCATTGCATAATTGATTTTTCTATAGCATTTCAGA
TTTTAAATGTAGATATATTCATTCTTCTTTACATATAACCAACCATAACTAACTTGT
GGAACATATCATAAAA
>Olfr267 BTBR/T_+_Itpr3tf/J
GGATCTTTGGGGCATTATCCCTGAAGGATGAGAAGTTGCAAACCTCTGTGTAATGCA
GCTGCTAAACTTTAAATTGTTCAGGGACAATGAGTATTCCCCCAAAACAAGTGATTT
AAATGTATATAGTATTGCAGTTTGGGATCCTTTGGTAATTTTGGTTATGTTTGTAAC
ATCTGGTCATTTTGAAAGAAATGCATTGCATAATTGATTTTTCTATAGCATTTCAGA
TTTTAAATGTAGATATATTCATTCTTCTTTACATATAACCAACCATAACTAACTTGT
GGAACATATCATAAAA
>Olfr267 BUB/BnJ
GGATCTTTGGGGCATTATCCCTGAAGGATGAGAAGTTGCAAACCTCTGTGTAATGCA
GCTGCTAAACTTTAAATTGTTCAGGGACAATGAGTATTCCCCCAAAACAAGTGATTT
AAATGTATATAGTATTGCAGTTTGGGATCCTTTGGTAATTTTGGTTATGTTTGTAAC
ATCTGGTCATTTTGAAAGAAATGCATTGCATAATTGATTTTTCTATAGCATTTCAGA
TTTTAAATGTAGATATATTCATTCTTCTTTACATATAACCAACCATAACTAACTTGT
GGAACATATCATAAAA
>Olfr267 C3H/HeH
GGATCTTTGGGGCATTATCCCTGAAGGATGAGAAGTTGCAAACCTCTGTGTAATGCA
GCTGCTAAACTTTAAATTGTTCAGGGACAATGAGTATTCCCCCAAAACAAGTGATTT
AAATGTATATAGTATTGCAGTTTGGGATCCTTTGGTAATTTTGGTTATGTTTGTAAC
ATCTGGTCATTTTGAAAGAAATGCATTGCATAATTGATTTTTCTATAGCATTTCAGA
TTTTAAATGTAGATATATTCATTCTTCTTTACATATAACCAACCATAACTAACTTGT
GGAACATATCATAAAA
>Olfr267 C3H/HeJ
GGATCTTTGGGGCATTATCCCTGAAGGATGAGAAGTTGCAAACCTCTGTGTAATGCA
GCTGCTAAACTTTAAATTGTTCAGGGACAATGAGTATTCCCCCAAAACAAGTGATTT
AAATGTATATAGTATTGCAGTTTGGGATCCTTTGGTAATTTTGGTTATGTTTGTAAC
ATCTGGTCATTTTGAAAGAAATGCATTGCATAATTGATTTTTCTATAGCATTTCAGA
TTTTAAATGTAGATATATTCATTCTTCTTTACATATAACCAACCATAACTAACTTGT
GGAACATATCATAAAA
>Olfr267 C57BL/10J
GGATCTTTGGGGCATTATCCCTGAAGGATGAGAAGTTGCAAACCTCTGTGTAATGCA
GCTGCTAAACTTTAAATTGTTCAGGGACAATGAGTATTCCCCCAAAACAAGTGATTT
AAATGTATATAGTACTGCAGTTTGGGATCCTTTGGTAATTTTGGTTATGTTTGTAAC
ATCTGGTCATTTTGAAAGAAATGCATTGCATAATTGATTTTTCTATAGCATTTCAGA
TTTTAAATGTAGATATATTCATTCTTCTTTACATATAACCAACCATAACTAACTTGT
GGAACATATCATAAAA
>Olfr267 C57BL/6NJ
GGATCTTTGGGGCATTATCCCTGAAGGATGAGAAGTTGCAAACCTCTGTGTAATGCA
GCTGCTAAACTTTAAATTGTTCAGGGACAATGAGTATTCCCCCAAAACAAGTGATTT
AAATGTATATAGTACTGCAGTTTGGGATCCTTTGGTAATTTTGGTTATGTTTGTAAC
ATCTGGTCATTTTGAAAGAAATGCATTGCATAATTGATTTTTCTATAGCATTTCAGA
TTTTAAATGTAGATATATTCATTCTTCTTTACATATAACCAACCATAACTAACTTGT
GGAACATATCATAAAA
>Olfr267 C57BR/cdJ
GGATCTTTGGGGCATTATCCCTGAAGGATGAGAAGTTGCAAACCTCTGTGTAATGCA

GCTGCTAAACTTTAAATTGTTCAGGGACAATGAGTATTCCCCCAAAACAAGTGATTT
AAATGTATATAGTATTGCAGTTTGGGATCCTTTGGTAATTTTGGTTATGTTTGTAAC
ATCTGGTCATTTTGAAAGAAATGCATTGCATAATTGATTTTTCTATAGCATTTCAGA
TTTTAAATGTAGATATATTCATTCTTCTTTACATATAACCAACCATAACTAACTTGT
GGAACATATCATAAAA
>Olfr267 C57L/J
GGATCTTTGGGGCATTATCCCTGAAGGATGAGAAGTTGCAAACCTCTGTGTAATGCA
GCTGCTAAACTTTAAATTGTTCAGGGACAATGAGTATTCCCCCAAAACAAGTGATTT
AAATGTATATAGTATTGCAGTTTGGGATCCTTTGGTAATTTTGGTTATGTTTGTAAC
ATCTGGTCATTTTGAAAGAAATGCATTGCATAATTGATTTTTCTATAGCATTTCAGA
TTTTAAATGTAGATATATTCATTCTTCTTTACATATAACCAACCATAACTAACTTGT
GGAACATATCATAAAA
>Olfr267 C58/J
GGATCTTTGGGGCATTATCCCTGAAGGATGAGAAGTTGCAAACCTCTGTGTAATGCA
GCTGCTAAACTTTAAATTGTTCAGGGACAATGAGTATTCCCCCAAAACAAGTGATTT
AAATGTATATAGTACTGCAGTTTGGGATCCTTTGGTAATTTTGGTTATGTTTGTAAC
ATCTGGTCATTTTGAAAGAAATGCATTGCATAATTGATTTTTCTATAGCATTTCAGA
TTTTAAATGTAGATATATTCATTCTTCTTTACATATAACCAACCATAACTAACTTGT
GGAACATATCATAAAA
>Olfr267 CAST/EiJ
GGATCTTTGGGGCATTATCCCTGAAGGATGAGAAGTTGCAAACCTCTGTATAATACA
GCTGCTAAACTTTAAATTGTTCAGGGACAATGAGTATTCCCCAAAACAACAAGTGAT
TTAAATGTATATAGTACTGCGGTTTGGGATCCTTTGGTAATTTTGGTTATGTTTGTA
ACATCTGGTCATTTTGAAAGAAATGCATTGCATAATTGATTTTTCTATAGCATTTCA
GATTTTAAATGTAGATATATTCATTCTTCTTTACATATAACCAACCATAACTAACTT
GTGGAACATATCATAAAA
>Olfr267 CBA/J
GGATCTTTGGGGCATTATCCCTGAAGGATGAGAAGTTGCAAACCTCTGTGTAATGCA
GCTGCTAAACTTTAAATTGTTCAGGGACAATGAGTATTCCCCCAAAACAAGTGATTT
AAATGTATATAGTATTGCAGTTTGGGATCCTTTGGTAATTTTGGTTATGTTTGTAAC
ATCTGGTCATTTTGAAAGAAATGCATTGCATAATTGATTTTTCTATAGCATTTCAGA
TTTTAAATGTAGATATATTCATTCTTCTTTACATATAACCAACCATAACTAACTTGT
GGAACATATCATAAAA
>Olfr267 DBA/1J
GGATCTTTGGGGCATTATCCCTGAAGGATGAGAAGTTGCAAACCTCTGTGTAATGCA
GCTGCTAAACTTTAAATTGTTCAGGGACAATGAGTATTCCCCCAAAACAAGTGATTT
AAATGTATATAGTATTGCAGTTTGGGATCCTTTGGTAATTTTGGTTATGTTTGTAAC
ATCTGGTCATTTTGAAAGAAATGCATTGCATAATTGATTTTTCTATAGCATTTCAGA
TTTTAAATGTAGATATATTCATTCTTCTTTACATATAACCAACCATAACTAACTTGT
GGAACATATCATAAAA
>Olfr267 DBA/2J
GGATCTTTGGGGCATTATCCCTGAAGGATGAGAAGTTGCAAACCTCTGTGTAATGCA
GCTGCTAAACTTTAAATTGTTCAGGGACAATGAGTATTCCCCCAAAACAAGTGATTT
AAATGTATATAGTATTGCAGTTTGGGATCCTTTGGTAATTTTGGTTATGTTTGTAAC
ATCTGGTCATTTTGAAAGAAATGCATTGCATAATTGATTTTTCTATAGCATTTCAGA
TTTTAAATGTAGATATATTCATTCTTCTTTACATATAACCAACCATAACTAACTTGT
GGAACATATCATAAAA
>Olfr267 FVB/NJ
GGATCTTTGGGGCATTATCCCTGAAGGATGAGAAGTTGCAAACCTCTGTGTAATGCA
GCTGCTAAACTTTAAATTGTTCAGGGACAATGAGTATTCCCCCAAAACAAGTGATTT
AAATGTATATAGTATTGCAGTTTGGGATCCTTTGGTAATTTTGGTTATGTTTGTAAC

ATCTGGTCATTTTGAAAGAAATGCATTGCATAATTGATTTTTCTATAGCATTTCAGA
TTTTAAATGTAGATATATTCATTCTTCTTTACATATAACCAACCATAACTAACTTGT
GGAACATATCATAAAA
>Olfr267 I/LnJ
GGATCTTTGGGGCATTATCCCTGAAGGATGAGAAGTTGCAAACCTCTGTGTAATGCA
GCTGCTAAACTTTAAATTGTTCAGGGACAATGAGTATTCCCCCAAAACAAGTGATTT
AAATGTATATAGTATTGCAGTTTGGGATCCTTTGGTAATTTTGGTTATGTTTGTAAC
ATCTGGTCATTTTGAAAGAAATGCATTGCATAATTGATTTTTCTATAGCATTTCAGA
TTTTAAATGTAGATATATTCATTCTTCTTTACATATAACCAACCATAACTAACTTGT
GGAACATATCATAAAA
>Olfr267 KK/HiJ
GGATCTTTGGGGCATTATCCCTGAAGGATGAGAAGTTGCAAACCTCTGTGTAATGCA
GCTGCTAAACTTTAAATTGTTCAGGGACAATGAGTATTCCCCCAAAACAAGTGATTT
AAATGTATATAGTACTGCAGTTTGGGATCCTTTGGTAATTTTGGTTATGTTTGTAAC
ATCTGGTCATTTTGAAAGAAATGCATTGCATAATTGATTTTTCTATAGCATTTCAGA
TTTTAAATGTAGATATATTCATTCTTCTTTACATATAACCAACCATAACTAACTTGT
GGAACATATCATAAAA
>Olfr267 LEWES/EiJ
GGATCTTTGGGGCATTATCCCTGAAGGATGAGAAGTTGCAAACCTCTGTGTAATGCA
GCTGCTAAACTTTAAATTGTTCAGGGACAATGAGTATTCCCCCAAAACAAGTGATTT
AAATGTATATAGTACTGCAGTTTGGGATCCTTTGGTAATTTTGGTTATGTTTGTAAC
ATCTGGTCATTTTGAAAGAAATGCATTGCATAATTGATTTTTCTATAGCATTTCAGA
TTTTAAATGTAGATATATTCATTCTTCTTTACATATAACCAACCATAACTAACTTGT
GGAACATATCATAAAA
>Olfr267 LP/J
GGATCTTTGGGGCATTATCCCTGAAGGATGAGAAGTTGCAAACCTCTGTGTAATGCA
GCTGCTAAACTTTAAATTGTTCAGGGACAATGAGTATTCCCCCAAAACAAGTGATTT
AAATGTATATAGTACTGCAGTTTGGGATCCTTTGGTAATTTTGGTTATGTTTGTAAC
ATCTGGTCATTTTGAAAGAAATGCATTGCATAATTGATTTTTCTATAGCATTTCAGA
TTTTAAATGTAGATATATTCATTCTTCTTTACATATAACCAACCATAACTAACTTGT
GGAACATATCATAAAA
>Olfr267 MOLF/EiJ
GGATCTTTGGGGCATTATCCCTGAAGGATGAGAAGTTGCAAACCTCTGTATAATACA
GCTGCTAAACTTTAAATTGTTCAGGGACAATGAGTATTCCCCCAAAACAACAAGTGG
TTTAAATGTATATAGTACTGCAGTTTGGGATCCTTTGGTAATTTTGGTTATGTTTGT
AACATCTGGTCATTTTGAAAGAAATGCATTGCATAATTGATTTTTCTATAGCATTTC
AGATTTTAAATGTAGATATATTCATTCTTCTTTACATATAACCAACCATAACTAACT
GTGGAACATATCATAAAA
>Olfr267 NOD/ShiLtJ
GGATCTTTGGGGCATTATCCCTGAAGGATGAGAAGTTGCAAACCTCTGTGTAATGCA
GCTGCTAAACTTTAAATTGTTCAGGGACAATGAGTATTCCCCCAAAACAAGTGATTT
AAATGTATATAGTATTGCAGTTTGGGATCCTTTGGTAATTTTGGTTATGTTTGTAAC
ATCTGGTCATTTTGAAAGAAATGCATTGCATAATTGATTTTTCTATAGCATTTCAGA
TTTTAAATGTAGATATATTCATTCTTCTTTACATATAACCAACCATAACTAACTTGT
GGAACATATCATAAAA
>Olfr267 NZB/B1NJ
GGATCTTTGGGGCATTATCCCTGAAGGATGAGAAGTTGCAAACCTCTGTGTAATGCA
GCTGCTAAACTTTAAATTGTTCAGGGACAATGAGTATTCCCCCAAAACAAGTGATTT
AAATGTATATAGTATTGCAGTTTGGGATCCTTTGGTAATTTTGGTTATGTTTGTAAC
ATCTGGTCATTTTGAAAGAAATGCATTGCATAATTGATTTTTCTATAGCATTTCAGA
TTTTAAATGTAGATATATTCATTCTTCTTTACATATAACCAACCATAACTAACTTGT

GGAACATATCATAAAA
>Olfr267 NZO/HlLtJ
GGATCTTTGGGGCATTATCCCTGAAGGATGAGAAGTTGCAAACCTCTGTGTAATGCA
GCTGCTAAACTTTAAATTGTTCAGGGACAATGAGTATTCCCCCAAAACAAGTGATTT
AAATGTATATAGTATTGCAGTTTGGGATCCTTTGGTAATTTTGGTTATGTTTGTAAC
ATCTGGTCATTTTGAAAGAAATGCATTGCATAATTGATTTTTCTATAGCATTTCAGA
TTTTAAATGTAGATATATTCATTCTTCTTTACATATAACCAACCATAACTAACTTGT
GGAACATATCATAAAA
>Olfr267 NZW/LacJ
GGATCTTTGGGGCATTATCCCTGAAGGATGAGAAGTTGCAAACCTCTGTGTAATGCA
GCTGCTAAACTTTAAATTGTTCAGGGACAATGAGTATTCCCCCAAAACAAGTGATTT
AAATGTATATAGTATTGCAGTTTGGGATCCTTTGGTAATTTTGGTTATGTTTGTAAC
ATCTGGTCATTTTGAAAGAAATGCATTGCATAATTGATTTTTCTATAGCATTTCAGA
TTTTAAATGTAGATATATTCATTCTTCTTTACATATAACCAACCATAACTAACTTGT
GGAACATATCATAAAA
>Olfr267 PWK/PhJ
GGATCTTTGGGGCATTATCCCTGAAGGATGAGAAGTTGCAAATCTCTGTATAATACA
GCTGCTAAACTTTAAATTGTTCAGGGACAATGAGTATTCCCCCAAAACAACAAGTGG
TTTAAATGTATATAGTACTGCAGTTTGGGATCCTTTGGTAATTTTGGTTATGTTTGT
AACATCTGGTCATTTTGAAAGAAATGCATTGCATAATTGATTTTTCTATAGCATTTC
AGATTTTAAATGTAGATATATTCATTCTTCTTTACATATAACCAACCATAACTAACT
GTGGAACATATCATAAAA
>Olfr267 RF/J
GGATCTTTGGGGCATTATCCCTGAAGGATGAGAAGTTGCAAACCTCTGTGTAATGCA
GCTGCTAAACTTTAAATTGTTCAGGGACAATGAGTATTCCCCCAAAACAAGTGATTT
AAATGTATATAGTATTGCAGTTTGGGATCCTTTGGTAATTTTGGTTATGTTTGTAAC
ATCTGGTCATTTTGAAAGAAATGCATTGCATAATTGATTTTTCTATAGCATTTCAGA
TTTTAAATGTAGATATATTCATTCTTCTTTACATATAACCAACCATAACTAACTTGT
GGAACATATCATAAAA
>Olfr267 SEA/GnJ
GGATCTTTGGGGCATTATCCCTGAAGGATGAGAAGTTGCAAACCTCTGTGTAATGCA
GCTGCTAAACTTTAAATTGTTCAGGGACAATGAGTATTCCCCCAAAACAAGTGATTT
AAATGTATATAGTATTGCAGTTTGGGATCCTTTGGTAATTTTGGTTATGTTTGTAAC
ATCTGGTCATTTTGAAAGAAATGCATTGCATAATTGATTTTTCTATAGCATTTCAGA
TTTTAAATGTAGATATATTCATTCTTCTTTACATATAACCAACCATAACTAACTTGT
GGAACATATCATAAAA
>Olfr267 SPRET/EiJ
GGATCTTTGGGGCATTATCCCTGAAGGATGAGAAGCTGCAAACCTCTGTATAATACA
GCTGCTAAACTTTAAATTGTTCAGGGACAATGAGTATTCCCCCAAAACAACAAGTGA
TTTAAATGTATATAGTACTGCAGTTTGGGATCCTTTGGTAATTTTGGTTATGTTTGT
AACATTTGGTCATTTTGAAAGAAATGCATTGCATAATTGATTTTTCTATAGCATTTC
AGATTTTAAATGTAGATATATTCATTCTTCTTTACATATAACCAGCCATAACTAACT
TGTGGAACATATCATAAAA
>Olfr267 ST/bJ
GGATCTTTGGGGCATTATCCCTGAAGGATGAGAAGTTGCAAACCTCTGTGTAATGCA
GCTGCTAAACTTTAAATTGTTCAGGGACAATGAGTATTCCCCCAAAACAAGTGATTT
AAATGTATATAGTATTGCAGTTTGGGATCCTTTGGTAATTTTGGTTATGTTTGTAAC
ATCTGGTCATTTTGAAAGAAATGCATTGCATAATTGATTTTTCTATAGCATTTCAGA
TTTTAAATGTAGATATATTCATTCTTCTTTACATATAACCAACCATAACTAACTTGT
GGAACATATCATAAAA
>Olfr267 WSB/EiJ

GGATCTTTGGGGCATTATCCCTGAAGGATGAGAAGTTGCAAACCTCTGTGTAATGCA
GCTGCTAAACTTTAAATTGTTCAGGGACAATGAGTATTCCCCCAAAACAAGTGATTT
AAATGTATATAGTACTGCAGTTTGGGATCCTTTGGTAATTTTGGTTATGTTTGTAAC
ATCTGGTCATTTTGAAAGAAATGCATTGCATAATTGATTTTTCTATAGCATTTCAGA
TTTTAAATGTAGATATATTCATTCTTCTTTACATATAACCAACCATAACTAACTTGT
GGAACATATCATAAAA
>Olfr267 ZALENDE/EiJ
GGATCTTTGGGGCATTATCCCTGAAGGATGAGAAGTTGCAAACCTCTGTGTAATGCA
GCTGCTAAACTTTAAATTGTTCAGGGACAATGAGTATTCCCCCAAAACAAGTGATTT
AAATGTATATAGTATTGCAGTTTGGGATCCTTTGGTAATTTTGGTTATGTTTGTAAC
ATCTGGTCATTTTGAAAGAAATGCATTGCATAATTGATTTTTCTATAGCATTTCAGA
TTTTAAATGTAGATATATTCATTCTTCTTTACATATAACCAACCATAACTAACTTGT
GGAACATATCATAAAA

>Olfr370 C57BL/6J
GTCTATTTTCTACAACCAACCAAGCTGTTAATTAAACCCAGTGCAGCATCTCCATGG
CAATGCCTTTGGCATCAGGAGACCCACTGAAGACCTTGTACTACCAAAAATGAAGCA
ACTCCTTTATGGTCACTATTAGCCATGACTGCGGTGCTTGGCTAATTAATATAAAAG
GGCACAAAAAGGCTGGTTACATTTGTTTCAGTCTGGGTCTCAGACCCCAGACTATGA
TTCCATACCTCCCTGAGTAGACAGCAGCAAACTTTAACTCACCTCGTACTTCCTCTT
CTTCTAGCAGTCTATG
>Olfr370 Wild_consensus
GTCTATTTTCTACAACCAAGCAAGCTGTTAATTAAACCCAGTGCAGCATCTCCATGG
CAATGCCTTTGGCATCAGGAGACCCACTGAAGACCTTGTACTACCAAAAATGAAGCA
ACTCCTTTATGGTCACTATTAGCCATGACATCGATGCTTGGCTAATTAATATAAAAG
GGCACAAAAAAGGCTGGTTACATTTGTTTCGGTCTGGGTCTCAGACCCCAGACTATG
ATTCCATACCTCCCTGAGTAGACAGCAGCAAACTTTAACTCACCTCGTACTTCCTCT
TCTTCTAGCAGTCTATG
>Olfr370 France
GTCTATTTTCTACAACCAAGCAAGCTGTTAATTAAACCCAGTGCAGCATCTCCATGG
CAATGCCTTTGGCATCAGGAGACCCACTGAAGACCTTGTACTACCAAAAATGAAGCA
ACTCCTTTATGGTCACTATTAGCCATGACATCGATGCTTGGCTAATTAATATAAAAG
GGCACAAAAAAGGCTGGTTACATTTGTTTCGGTCTGGGTCTCAGACCCCAGACTATG
ATTCCATACCTCCCTGAGTAGACAGCAGCAAACTTTAACTCACCTCGTACTTCCTCT
TCTTCTAGCAGTCTATG
>Olfr370 Germany
GTCTATTTTCTACAACCAAGCAAGCTGTTAATTAAACCCAGTGCAGCATCTCCATGG
CAATGCCTTTGGCATCAGGAGACCCACTGAAGACCTTGTACTACCAAAAATGAAGCA
ACTCCTTTATGGTCACTATTAGCCATGACATCGATGCTTGGCTAATTAATATAAAAG
GGCACAAAAAAGGCTGGTTACATTTGTTTCGGTCTGGGTCTCAGACCCCAGACTATG
ATTCCATACCTCCCTGAGTAGACAGCAGCAAACTTTAACTCACCTCGTACTTCCTCT
TCTTCTAGCAGTCTATG
>Olfr370 Iran
GTCTATTTTCTACAACCAAGCAAGCTGTTAATTAAACCCAGTGCAGCATCTCCATGG
CAATGCCTTTGGCATCAGGAGACCCACTGAAGACCTTGTACTACCAAAAATGAAGCA
ACTCCTTTATGGTCACTATTAGCCATGACATCGATGCTTGGCTAATTAATATAAAAG
GGCACAAAAAGGCTGGTTACATTTGTTTCGGTCTGGGTCTCAGACCCCAGACTATGA
TTCCATACCTCCCTGAGTAGACAGCAGCAAACTTTAACTCACCTCGTACTTCCTCTT
CTTCTAGCAGTCTATG
>Olfr370 129P2/OlaHsd
GTCTATTTTCTACAACCAAGCAAGCTGTTAATTAAACCCAGTGCAGCATCTCCATGG

CAATGCCTTTGGCATCAGGAGACCCACTGAAGACCTTGTACTACCAAAAATGAAGCA
ACTCCTTTATGGTCACTATTAGCCATGACATCGATGCTTGGCTAATTAATATAAAAG
GGCACAAAAAAGGCTGGTTACATTTGTTTCGGTCTGGGTCTCAGACCCCAGACTATG
ATTCCATACCTCCCTGAGTAGACAGCAGCAAACTTTAACTCACCTCGTACTTCCTCT
TCTTCTAGCAGTCTATG
>Olfr370 129S1/SvImJ
GTCTATTTTCTACAACCAAGCAAGCTGTTAATTAAACCCAGTGCAGCATCTCCATGG
CAATGCCTTTGGCATCAGGAGACCCACTGAAGACCTTGTACTACCAAAAATGAAGCA
ACTCCTTTATGGTCACTATTAGCCATGACATCGATGCTTGGCTAATTAATATAAAAG
GGCACAAAAAAGGCTGGTTACATTTGTTTCGGTCTGGGTCTCAGACCCCAGACTATG
ATTCCATACCTCCCTGAGTAGACAGCAGCAAACTTTAACTCACCTCGTACTTCCTCT
TCTTCTAGCAGTCTATG
>Olfr370 129S5SvEvBrd
GTCTATTTTCTACAACCAAGCAAGCTGTTAATTAAACCCAGTGCAGCATCTCCATGG
CAATGCCTTTGGCATCAGGAGACCCACTGAAGACCTTGTACTACCAAAAATGAAGCA
ACTCCTTTATGGTCACTATTAGCCATGACATCGATGCTTGGCTAATTAATATAAAAG
GGCACAAAAAAGGCTGGTTACATTTGTTTCGGTCTGGGTCTCAGACCCCAGACTATG
ATTCCATACCTCCCTGAGTAGACAGCAGCAAACTTTAACTCACCTCGTACTTCCTCT
TCTTCTAGCAGTCTATG
>Olfr370 A/J
GTCTATTTTCTACAACCAAGCAAGCTGTTAATTAAACCCAGTGCAGCATCTCCATGG
CAATGCCTTTGGCATCAGGAGACCCACTGAAGACCTTGTACTACCAAAAATGAAGCA
ACTCCTTTATGGTCACTATTAGCCATGACATCGATGCTTGGCTAATTAATATAAAAG
GGCACAAAAAAGGCTGGTTACATTTGTTTCGGTCTGGGTCTCAGACCCCAGACTATG
ATTCCATACCTCCCTGAGTAGACAGCAGCAAACTTTAACTCACCTCGTACTTCCTCT
TCTTCTAGCAGTCTATG
>Olfr370 AKR/J
GTCTATTTTCTACAACCAAGCAAGCTGTTAATTAAACCCAGTGCAGCATCTCCATGG
CAATGCCTTTGGCATCAGGAGACCCACTGAAGACCTTGTACTACCAAAAATGAAGCA
ACTCCTTTATGGTCACTATTAGCCATGACATCGATGCTTGGCTAATTAATATAAAAG
GGCACAAAAAAGGCTGGTTACATTTGTTTCGGTCTGGGTCTCAGACCCCAGACTATG
ATTCCATACCTCCCTGAGTAGACAGCAGCAAACTTTAACTCACCTCGTACTTCCTCT
TCTTCTAGCAGTCTATG
>Olfr370 BALB/cJ
GTCTATTTTCTACAACCAAGCAAGCTGTTAATTAAACCCAGTGCAGCATCTCCATGG
CAATGCCTTTGGCATCAGGAGACCCACTGAAGACCTTGTACTACCAAAAATGAAGCA
ACTCCTTTATGGTCACTATTAGCCATGACATCGATGCTTGGCTAATTAATATAAAAG
GGCACAAAAAAGGCTGGTTACATTTGTTTCGGTCTGGGTCTCAGACCCCAGACTATG
ATTCCATACCTCCCTGAGTAGACAGCAGCAAACTTTAACTCACCTCGTACTTCCTCT
TCTTCTAGCAGTCTATG
>Olfr370 BTBR/T_+_Itpr3tf/J
GTCTATTTTCTACAACCAAGCAAGCTGTTAATTAAACCCAGTGCAGCATCTCCATGG
CAATGCCTTTGGCATCAGGAGACCCACTGAAGACCTTGTACTACCAAAAATGAAGCA
ACTCCTTTATGGTCACTATTAGCCATGACATCGATGCTTGGCTAATTAATATAAAAG
GGCACAAAAAAGGCTGGTTACATTTGTTTCGGTCTGGGTCTCAGACCCCAGACTATG
ATTCCATACCTCCCTGAGTAGACAGCAGCAAACTTTAACTCACCTCGTACTTCCTCT
TCTTCTAGCAGTCTATG
>Olfr370 BUB/BnJ
GTCTATTTTCTACAACCAACCAAGCTGTTAATTAAACCCAGTGCAGCATCTCCATGG
CAATGCCTTTGGCATCAGGAGACCCACTGAAGACCTTGTACTACCAAAAATGAAGCA
ACTCCTTTATGGTCACTATTAGCCATGACTGCGGTGCTTGGCTAATTAATATAAAAG

GGCACAAAAAGGCTGGTTACATTTGTTTCAGTCTGGGTCTCAGACCCCAGACTATGA
TTCCATACCTCCCTGAGTAGACAGCAGCAAACTTTAACTCACCTCGTACTTCCTCTT
CTTCTAGCAGTCTATG
>Olfr370 C3H/HeH
GTCTATTTTCTACAACCAAGCAAGCTGTTAATTAAACCCAGTGCAGCATCTCCATGG
CAATGCCTTTGGCATCAGGAGACCCACTGAAGACCTTGTACTACCAAAAATGAAGCA
ACTCCTTTATGGTCACTATTAGCCATGACATCGATGCTTGGCTAATTAATATAAAAG
GGCACAAAAAAGGCTGGTTACATTTGTTTCGGTCTGGGTCTCAGACCCCAGACTATG
ATTCCATACCTCCCTGAGTAGACAGCAGCAAACTTTAACTCACCTCGTACTTCCTCT
TCTTCTAGCAGTCTATG
>Olfr370 C3H/HeJ
GTCTATTTTCTACAACCAAGCAAGCTGTTAATTAAACCCAGTGCAGCATCTCCATGG
CAATGCCTTTGGCATCAGGAGACCCACTGAAGACCTTGTACTACCAAAAATGAAGCA
ACTCCTTTATGGTCACTATTAGCCATGACATCGATGCTTGGCTAATTAATATAAAAG
GGCACAAAAAAGGCTGGTTACATTTGTTTCGGTCTGGGTCTCAGACCCCAGACTATG
ATTCCATACCTCCCTGAGTAGACAGCAGCAAACTTTAACTCACCTCGTACTTCCTCT
TCTTCTAGCAGTCTATG
>Olfr370 C57BL/10J
GTCTATTTTCTACAACCAACCAAGCTGTTAATTAAACCCAGTGCAGCATCTCCATGG
CAATGCCTTTGGCATCAGGAGACCCACTGAAGACCTTGTACTACCAAAAATGAAGCA
ACTCCTTTATGGTCACTATTAGCCATGACTGCGGTGCTTGGCTAATTAATATAAAAG
GGCACAAAAAGGCTGGTTACATTTGTTTCAGTCTGGGTCTCAGACCCCAGACTATGA
TTCCATACCTCCCTGAGTAGACAGCAGCAAACTTTAACTCACCTCGTACTTCCTCTT
CTTCTAGCAGTCTATG
>Olfr370 C57BL/6NJ
GTCTATTTTCTACAACCAACCAAGCTGTTAATTAAACCCAGTGCAGCATCTCCATGG
CAATGCCTTTGGCATCAGGAGACCCACTGAAGACCTTGTACTACCAAAAATGAAGCA
ACTCCTTTATGGTCACTATTAGCCATGACTGCGGTGCTTGGCTAATTAATATAAAAG
GGCACAAAAAGGCTGGTTACATTTGTTTCAGTCTGGGTCTCAGACCCCAGACTATGA
TTCCATACCTCCCTGAGTAGACAGCAGCAAACTTTAACTCACCTCGTACTTCCTCTT
CTTCTAGCAGTCTATG
>Olfr370 C57BR/cdJ
GTCTATTTTCTACAACCAACCAAGCTGTTAATTAAACCCAGTGCAGCATCTCCATGG
CAATGCCTTTGGCATCAGGAGACCCACTGAAGACCTTGTACTACCAAAAATGAAGCA
ACTCCTTTATGGTCACTATTAGCCATGACTGCGGTGCTTGGCTAATTAATATAAAAG
GGCACAAAAAGGCTGGTTACATTTGTTTCAGTCTGGGTCTCAGACCCCAGACTATGA
TTCCATACCTCCCTGAGTAGACAGCAGCAAACTTTAACTCACCTCGTACTTCCTCTT
CTTCTAGCAGTCTATG
>Olfr370 C57L/J
GTCTATTTTCTACAACCAACCAAGCTGTTAATTAAACCCAGTGCAGCATCTCCATGG
CAATGCCTTTGGCATCAGGAGACCCACTGAAGACCTTGTACTACCAAAAATGAAGCA
ACTCCTTTATGGTCACTATTAGCCATGACTGCGGTGCTTGGCTAATTAATATAAAAG
GGCACAAAAAGGCTGGTTACATTTGTTTCAGTCTGGGTCTCAGACCCCAGACTATGA
TTCCATACCTCCCTGAGTAGACAGCAGCAAACTTTAACTCACCTCGTACTTCCTCTT
CTTCTAGCAGTCTATG
>Olfr370 C58/J
GTCTATTTTCTACAACCAAGCAAGCTGTTAATTAAACCCAGTGCAGCATCTCCATGG
CAATGCCTTTGGCATCAGGAGACCCACTGAAGACCTTGTACTACCAAAAATGAAGCA
ACTCCTTTATGGTCACTATTAGCCATGACATCGATGCTTGGCTAATTAATATAAAAG
GGCACAAAAAAGGCTGGTTACATTTGTTTCGGTCTGGGTCTCAGACCCCAGACTATG
ATTCCATACCTCCCTGAGTAGACAGCAGCAAACTTTAACTCACCTCGTACTTCCTCT

TCTTCTAGCAGTCTATG
>Olfr370 CAST/EiJ
GTCTATTTTCTACAACCAACCAAGCTGTTAATTAAACCCAGTGCAGCATCTCCATGG
CAATGCCTTTGGCATCAGGAGACCCACTGAAGACCTTGTACTACCAAAAATGAAGCA
ACTCCTTTATGGTCACTATTAGCCATGACTGCGATGCTTGGCTAATTAATATAAAAG
GGCACAAAAAGGCTGGTTACATTTGTTTCAGTCTGGGTCTCAGACCCCAGACTATGA
TTCCATACCTCCCTGAGTAGACAGCAGCAAACTTTAACTCACCTCGTACTTCCTCTT
CTTCTAGCAGTCTATG
>Olfr370 CBA/J
GTCTATTTTCTACAACCAAGCAAGCTGTTAATTAAACCCAGTGCAGCATCTCCATGG
CAATGCCTTTGGCATCAGGAGACCCACTGAAGACCTTGTACTACCAAAAATGAAGCA
ACTCCTTTATGGTCACTATTAGCCATGACATCGATGCTTGGCTAATTAATATAAAAG
GGCACAAAAAAGGCTGGTTACATTTGTTTCGGTCTGGGTCTCAGACCCCAGACTATG
ATTCCATACCTCCCTGAGTAGACAGCAGCAAACTTTAACTCACCTCGTACTTCCTCT
TCTTCTAGCAGTCTATG
>Olfr370 DBA/1J
GTCTATTTTCTACAACCAAGCAAGCTGTTAATTAAACCCAGTGCAGCATCTCCATGG
CAATGCCTTTGGCATCAGGAGACCCACTGAAGACCTTGTACTACCAAAAATGAAGCA
ACTCCTTTATGGTCACTATTAGCCATGACATCGATGCTTGGCTAATTAATATAAAAG
GGCACAAAAAAGGCTGGTTACATTTGTTTCGGTCTGGGTCTCAGACCCCAGACTATG
ATTCCATACCTCCCTGAGTAGACAGCAGCAAACTTTAACTCACCTCGTACTTCCTCT
TCTTCTAGCAGTCTATG
>Olfr370 DBA/2J
GTCTATTTTCTACAACCAAGCAAGCTGTTAATTAAACCCAGTGCAGCATCTCCATGG
CAATGCCTTTGGCATCAGGAGACCCACTGAAGACCTTGTACTACCAAAAATGAAGCA
ACTCCTTTATGGTCACTATTAGCCATGACATCGATGCTTGGCTAATTAATATAAAAG
GGCACAAAAAAGGCTGGTTACATTTGTTTCGGTCTGGGTCTCAGACCCCAGACTATG
ATTCCATACCTCCCTGAGTAGACAGCAGCAAACTTTAACTCACCTCGTACTTCCTCT
TCTTCTAGCAGTCTATG
>Olfr370 FVB/NJ
GTCTATTTTCTACAACCAACCAAGCTGTTAATTAAACCCAGTGCAGCATCTCCATGG
CAATGCCTTTGGCATCAGGAGACCCACTGAAGACCTTGTACTACCAAAAATGAAGCA
ACTCCTTTATGGTCACTATTAGCCATGACTGCGGTGCTTGGCTAATTAATATAAAAG
GGCACAAAAAGGCTGGTTACATTTGTTTCAGTCTGGGTCTCAGACCCCAGACTATGA
TTCCATACCTCCCTGAGTAGACAGCAGCAAACTTTAACTCACCTCGTACTTCCTCTT
CTTCTAGCAGTCTATG
>Olfr370 I/LnJ
GTCTATTTTCTACAACCAAGCAAGCTGTTAATTAAACCCAGTGCAGCATCTCCATGG
CAATGCCTTTGGCATCAGGAGACCCACTGAAGACCTTGTACTACCAAAAATGAAGCA
ACTCCTTTATGGTCACTATTAGCCATGACATCGATGCTTGGCTAATTAATATAAAAG
GGCACAAAAAAGGCTGGTTACATTTGTTTCGGTCTGGGTCTCAGACCCCAGACTATG
ATTCCATACCTCCCTGAGTAGACAGCAGCAAACTTTAACTCACCTCGTACTTCCTCT
TCTTCTAGCAGTCTATG
>Olfr370 KK/HiJ
GTCTATTTTCTACAACCAAGCAAGCTGTTAATTAAACCCAGTGCAGCATCTCCATGG
CAATGCCTTTGGCATCAGGAGACCCACTGAAGACCTTGTACTACCAAAAATGAAGCA
ACTCCTTTATGGTCACTATTAGCCATGACATCGATGCTTGGCTAATTAATATAAAAG
GGCACAAAAAAGGCTGGTTACATTTGTTTCGGTCTGGGTCTCAGACCCCAGACTATG
ATTCCATACCTCCCTGAGTAGACAGCAGCAAACTTTAACTCACCTCGTACTTCCTCT
TCTTCTAGCAGTCTATG
>Olfr370 LEWES/EiJ

GTCTATTTTCTACAACCAAGCAAGCTGTTAATTAAACCCAGTGCAGCATCTCCATGG
CAATGCCTTTGGCATCAGGAGACCCACTGAAGACCTTGTACTACCAAAAATGAAGCA
ACTCCTTTATGGTCACTATTAGCCATGACATCGATGCTTGGCTAATTAATATAAAAG
GGCACAAAAAAGGCTGGTTACATTTGTTTCGGTCTGGGTCTCAGACCCCAGACTATG
ATTCCATACCTCCCTGAGTAGACAGCAGCAAACTTTAACTCACCTCGTACTTCCTCT
TCTTCTAGCAGTCTATG
>Olfr370 LP/J
GTCTATTTTCTACAACCAAGCAAGCTGTTAATTAAACCCAGTGCAGCATCTCCATGG
CAATGCCTTTGGCATCAGGAGACCCACTGAAGACCTTGTACTACCAAAAATGAAGCA
ACTCCTTTATGGTCACTATTAGCCATGACATCGATGCTTGGCTAATTAATATAAAAG
GGCACAAAAAAGGCTGGTTACATTTGTTTCGGTCTGGGTCTCAGACCCCAGACTATG
ATTCCATACCTCCCTGAGTAGACAGCAGCAAACTTTAACTCACCTCGTACTTCCTCT
TCTTCTAGCAGTCTATG
>Olfr370 MOLF/EiJ
GTCTATTTTCTACAACCAACCAAGCTGTTAATTAAACCCAGTGCAGCATCTCCATGG
CAATGCCTTTGGCATCAGGAGACCCACTGAAGACCTTGTACTACCAAAAATGAAGCA
ACTCCTTTATGGTCACTATTAGCCATGACTGCGGTGCTTGGCTAATTAATATAAAAG
GGCACAAAAAGGCTGGTTACATTTGTTTCAGTCTGGGTCTCAGACCCCAGACTATGA
TTCCATACCTCCCTGAGTAGACAGCAGCAAACTTTAACTCACCTCGTACTTCCTCTT
CTTCTAGCAGTCTATG
>Olfr370 NOD/ShiLtJ
GTCTATTTTCTACAACCAAGCAAGCTGTTAATTAAACCCAGTGCAGCATCTCCATGG
CAATGCCTTTGGCATCAGGAGACCCACTGAAGACCTTGTACTACCAAAAATGAAGCA
ACTCCTTTATGGTCACTATTAGCCATGACATCGATGCTTGGCTAATTAATATAAAAG
GGCACAAAAAAGGCTGGTTACATTTGTTTCGGTCTGGGTCTCAGACCCCAGACTATG
ATTCCATACCTCCCTGAGTAGACAGCAGCAAACTTTAACTCACCTCGTACTTCCTCT
TCTTCTAGCAGTCTATG
>Olfr370 NZB/B1NJ
GTCTATTTTCTACAACCAACCAAGCTGTTAATTAAACCCAGTGCAGCATCTCCATGG
CAATGCCTTTGGCATCAGGAGACCCACTGAAGACCTTGTACTACCAAAAATGAAGCA
ACTCCTTTATGGTCACTATTAGCCATGACTGCGGTGCTTGGCTAATTAATATAAAAG
GGCACAAAAAGGCTGGTTACATTTGTTTCAGTCTGGGTCTCAGACCCCAGACTATGA
TTCCATACCTCCCTGAGTAGACAGCAGCAAACTTTAACTCACCTCGTACTTCCTCTT
CTTCTAGCAGTCTATG
>Olfr370 NZO/HlLtJ
GTCTATTTTCTACAACCAACCAAGCTGTTAATTAAACCCAGTGCAGCATCTCCATGG
CAATGCCTTTGGCATCAGGAGACCCACTGAAGACCTTGTACTACCAAAAATGAAGCA
ACTCCTTTATGGTCACTATTAGCCATGACTGCGGTGCTTGGCTAATTAATATAAAAG
GGCACAAAAAGGCTGGTTACATTTGTTTCAGTCTGGGTCTCAGACCCCAGACTATGA
TTCCATACCTCCCTGAGTAGACAGCAGCAAACTTTAACTCACCTCGTACTTCCTCTT
CTTCTAGCAGTCTATG
>Olfr370 NZW/LacJ
GTCTATTTTCTACAACCAAGCAAGCTGTTAATTAAACCCAGTGCAGCATCTCCATGG
CAATGCCTTTGGCATCAGGAGACCCACTGAAGACCTTGTACTACCAAAAATGAAGCA
ACTCCTTTATGGTCACTATTAGCCATGACATCGATGCTTGGCTAATTAATATAAAAG
GGCACAAAAAAGGCTGGTTACATTTGTTTCGGTCTGGGTCTCAGACCCCAGACTATG
ATTCCATACCTCCCTGAGTAGACAGCAGCAAACTTTAACTCACCTCGTACTTCCTCT
TCTTCTAGCAGTCTATG
>Olfr370 PWK/PhJ
GTCTATTTTCTACAACCAACCAAGCTGTTAATTAAACCCAGTGCAGCATCTCCATGG
CAATGCCTTTGGCATCAGGAGACCCACTGAAGACCTTGTACTACCAAAAATGAAGCA

ACTCCTTTATGGTCACTATTAGCCATGACTGCGATGCTTGGCTAATTAATATAAAAG
GGCACAAAAAGGCTGGTTACATTTGTTTCAGTCTGGGTCTCAGACCCCAGACTATGA
TTCCATACCTCCCTGAGTAGACAGCAGCAAACTTTAACTCACCTCGTACTTCCTCTT
CTTCTAGCAGTCTATG
>Olfr370 RF/J
GTCTATTTTCTACAACCAAGCAAGCTGTTAATTAAACCCAGTGCAGCATCTCCATGG
CAATGCCTTTGGCATCAGGAGACCCACTGAAGACCTTGTACTACCAAAAATGAAGCA
ACTCCTTTATGGTCACTATTAGCCATGACATCGATGCTTGGCTAATTAATATAAAAG
GGCACAAAAAAGGCTGGTTACATTTGTTTCGGTCTGGGTCTCAGACCCCAGACTATG
ATTCCATACCTCCCTGAGTAGACAGCAGCAAACTTTAACTCACCTCGTACTTCCTCT
TCTTCTAGCAGTCTATG
>Olfr370 SEA/GnJ
GTCTATTTTCTACAACCAAGCAAGCTGTTAATTAAACCCAGTGCAGCATCTCCATGG
CAATGCCTTTGGCATCAGGAGACCCACTGAAGACCTTGTACTACCAAAAATGAAGCA
ACTCCTTTATGGTCACTATTAGCCATGACATCGATGCTTGGCTAATTAATATAAAAG
GGCACAAAAAAGGCTGGTTACATTTGTTTCGGTCTGGGTCTCAGACCCCAGACTATG
ATTCCATACCTCCCTGAGTAGACAGCAGCAAACTTTAACTCACCTCGTACTTCCTCT
TCTTCTAGCAGTCTATG
>Olfr370 SPRET/EiJ
GTCTATTTTCTACAACCAACCAAGCTGTTAATTAAACCCAGTGCAGCATCTCCATGG
CAATGCCTTTGGCATCAGGAGACCCACTGAAGACCTTGTACTACCAAAAATGAAGCA
ACTCCTTTATGGTCACTATTAGCCATGACAGTGATGCTTGGCTAATTAATATAAAAG
GGCACAAAAGGCTGGTTACATTTGTTTCGGTCTGGGTCTCAGACCCCAGACTATGAT
TGCATACCTCCCTGAGTAGACAGCAGCAAACTTTAACTCACCTTGTACTTCCTCTTC
TTCTAGCAGTCTATG
>Olfr370 ST/bJ
GTCTATTTTCTACAACCAAGCAAGCTGTTAATTAAACCCAGTGCAGCATCTCCATGG
CAATGCCTTTGGCATCAGGAGACCCACTGAAGACCTTGTACTACCAAAAATGAAGCA
ACTCCTTTATGGTCACTATTAGCCATGACATCGATGCTTGGCTAATTAATATAAAAG
GGCACAAAAAAGGCTGGTTACATTTGTTTCGGTCTGGGTCTCAGACCCCAGACTATG
ATTCCATACCTCCCTGAGTAGACAGCAGCAAACTTTAACTCACCTCGTACTTCCTCT
TCTTCTAGCAGTCTATG
>Olfr370 WSB/EiJ
GTCTATTTTCTACAACCAAGCAAGCTGTTAATTAAACCCAGTGCAGCATCTCCATGG
CAATGCCTTTGGCATCAGGAGACCCACTGAAGACCTTGTACTACCAAAAATGAAGCA
ACTCCTTTATGGTCACTATTAGCCATGACATCGATGCTTGGCTAATTAATATAAAAG
GGCACAAAAAAGGCTGGTTACATTTGTTTCGGTCTGGGTCTCAGACCCCAGACTATG
ATTCCATACCTCCCTGAGTAGACAGCAGCAAACTTTAACTCACCTCGTACTTCCTCT
TCTTCTAGCAGTCTATG
>Olfr370 ZALENDE/EiJ
GTCTATTTTCTACAACCAAGCAAGCTGTTAATTAAACCCAGTGCAGCATCTCCATGG
CAATGCCTTTGGCATCAGGAGACCCACTGAAGACCTTGTACTACCAAAAATGAAGCA
ACTCCTTTATGGTCACTATTAGCCATGACATCGATGCTTGGCTAATTAATATAAAAG
GGCACAAAAAAGGCTGGTTACATTTGTTTCGGTCTGGGTCTCAGACCCCAGACTATG
ATTCCATACCTCCCTGAGTAGACAGCAGCAAACTTTAACTCACCTCGTACTTCCTCT
TCTTCTAGCAGTCTATG

>Olfr371 C57BL/6J
CATTTTACAACATATTTCTCTATTTCCTTTTAAAGTGCTTAGTTTTGTAATGACCCT
TGGGGGAGGCAGCACAGTTTCTGAACTCAATAACCACAAGGGAACAAAACTGATTGG
AACTTTGCCATTAATGTTTTGGTCCCTAGGTTCCTAGCAGGTATATTGTCCAGAGAT

TATGAAATTATAAACTTTTATTTAATGAGAGGCTCTGAGAATTGAGTTCATGGAACC
AAATAAACAACACAGCTCCTGACATTGTCATTCACTAACCCAAGCATACATGAAAAA
ACAATCCCCCTGCTCC
>Olfr371 Wild_consensus
CATTTTACAACATATTTCTCTATTTCCTTTTAAAGTGCTTAGTTTTGTAATGACCCT
TGGGGGAGGCAGCACAGTTTCTGAACTCAATAACCACAAGGGAACAAAACTGATTGG
AACTTTGCCATTAATGTTTTGGTCCCTAGGTTCCTAGCAGGTATATTGTCCAGAGAT
TATGAAATTATAAACTTTTATTTAATGAGAGGCTCTGAGAATTGAGTTCATGGAACC
AAATAAACAACACAGCTCCTGACATTGTCATTCACTAACCCAAGCATACATGAAAAA
ACAATCCCCCTGCTCC
>Olfr371 France
CATTTTACAACATATTTCTCTATTTCCTTTTAAAGTGCTTAGTTTTGTAATGATCCT
TGGGGGAGGCAGCACAGTTTCTGAACTCAATAACCACAAGGGAACAAAACTGATTGG
AACTTTGCCATTAATGTTTTGGTCCCTAGGTTCCTAGCAGGTATATTGTCCAGAGAT
TATGAAATTATAAACTTTTATTTAATGAGAGGCTCTGAGAATTGAGTTCATGGAACC
AAATAAACAACACAGCTCCTGACATTGTCATTCACTAACCCAAGCATACATGAAAAA
ACAATCCCCCTGCTCC
>Olfr371 Germany
CATTTTACAACATATTTCTCTATTTCCTTTTAAAGTGCTTAGTTTTGTAATGACCCC
TGGGGGAGGCAGCACAGTTTCTGAACTCAATAACCACAAGGGAACAAAACTGATTGG
AACTTTGCCATTAATGTTTTGGTCCCTAGGTTCCTAGCAGGTATATTGTCCAGAGAT
TATGAAATTATAAACTTTTATTTAATGAGAGGCTCTGAGAATTGAGTTCATGGAACC
AAATAAACAACACAGCTCCTGACATTGTCATTCACTAACCCAAGCATACATGAAAAA
ACAATCCCCCTGCTCC
>Olfr371 Iran
CATTTTACAACATATTTCTCTATTTCCTTTTAAAGTGCTTAGTTTTGTAATGACCCT
TGGGGGAGGCAGCACAGTTTCTGAACTCAATAACCACAAGGGAACAAAACTGATTGG
AACTTTGCCATTAATGTTTTGGTCCCTAGGTTCCTAGCAGGTATATTGTCCAGAGAT
TATGAAATTATAAACTTTTATTTAATGAGAGGCTCTGAGAATTGAGTTCATGGAACC
AAATAAACAACACAGCTCCTGACATTGTCATTCACTAACCCAAGCATACATGAAAAA
ACAATCCCCCTGCTCC
>Olfr371 129P2/OlaHsd
CATTTTACAACATATTTCTCTATTTCCTTTTAAAGTGCTTAGTTTTGTAATGATCCT
TGGGGGAGGCAGCACAGTTTCTGAACTCAATAACCACAAGGGAACAAAACTGATTGG
AACTTTGCCATTAATGTTTTGGTCCCTAGGTTCCTAGCAGGTATATTGTCCAGAGAT
TATGAAATTATAAACTTTTATTTAATGAGAGGCTCTGAGAATTGAGTTCATGGAACC
AAATAAACAACACAGCTCCTGACATTGTCATTCACTAACCCAAGCATACATGAAAAA
ACAATCCCCCTGCTCC
>Olfr371 129S1/SvImJ
CATTTTACAACATATTTCTCTATTTCCTTTTAAAGTGCTTAGTTTTGTAATGATCCT
TGGGGGAGGCAGCACAGTTTCTGAACTCAATAACCACAAGGGAACAAAACTGATTGG
AACTTTGCCATTAATGTTTTGGTCCCTAGGTTCCTAGCAGGTATATTGTCCAGAGAT
TATGAAATTATAAACTTTTATTTAATGAGAGGCTCTGAGAATTGAGTTCATGGAACC
AAATAAACAACACAGCTCCTGACATTGTCATTCACTAACCCAAGCATACATGAAAAA
ACAATCCCCCTGCTCC
>Olfr371 129S5SvEvBrd
CATTTTACAACATATTTCTCTATTTCCTTTTAAAGTGCTTAGTTTTGTAATGATCCT
TGGGGGAGGCAGCACAGTTTCTGAACTCAATAACCACAAGGGAACAAAACTGATTGG
AACTTTGCCATTAATGTTTTGGTCCCTAGGTTCCTAGCAGGTATATTGTCCAGAGAT
TATGAAATTATAAACTTTTATTTAATGAGAGGCTCTGAGAATTGAGTTCATGGAACC
AAATAAACAACACAGCTCCTGACATTGTCATTCACTAACCCAAGCATACATGAAAAA

ACAATCCCCCTGCTCC
>Olfr371 A/J
CATTTTACAACATATTTCTCTATTTCCTTTTAAAGTGCTTAGTTTTGTAATGACCCT
TGGGGGAGGCAGCACAGTTTCTGAACTCAATAACCACAAGGGAACAAAACTGATTGG
AACTTTGCCATTAATGTTTTGGTCCCTAGGTTCCTAGCAGGTATATTGTCCAGAGAT
TATGAAATTATAAACTTTTATTTAATGAGAGGCTCTGAGAATTGAGTTCATGGAACC
AAATAAACAACACAGCTCCTGACATTGTCATTCACTAACCCAAGCATACATGAAAAA
ACAATCCCCCTGCTCC
>Olfr371 AKR/J
CATTTTACAACATATTTCTCTATTTCCTTTTAAAGTGCTTAGTTTTGTAATGATCCT
TGGGGGAGGCAGCACAGTTTCTGAACTCAATAACCACAAGGGAACAAAACTGATTGG
AACTTTGCCATTAATGTTTTGGTCCCTAGGTTCCTAGCAGGTATATTGTCCAGAGAT
TATGAAATTATAAACTTTTATTTAATGAGAGGCTCTGAGAATTGAGTTCATGGAACC
AAATAAACAACACAGCTCCTGACATTGTCATTCACTAACCCAAGCATACATGAAAAA
ACAATCCCCCTGCTCC
>Olfr371 BALB/cJ
CATTTTACAACATATTTCTCTATTTCCTTTTAAAGTGCTTAGTTTTGTAATGATCCT
TGGGGGAGGCAGCACAGTTTCTGAACTCAATAACCACAAGGGAACAAAACTGATTGG
AACTTTGCCATTAATGTTTTGGTCCCTAGGTTCCTAGCAGGTATATTGTCCAGAGAT
TATGAAATTATAAACTTTTATTTAATGAGAGGCTCTGAGAATTGAGTTCATGGAACC
AAATAAACAACACAGCTCCTGACATTGTCATTCACTAACCCAAGCATACATGAAAAA
ACAATCCCCCTGCTCC
>Olfr371 BTBR/T_+_Itpr3tf/J
CATTTTACAACATATTTCTCTATTTCCTTTTAAAGTGCTTAGTTTTGTAATGATCCT
TGGGGGAGGCAGCACAGTTTCTGAACTCAATAACCACAAGGGAACAAAACTGATTGG
AACTTTGCCATTAATGTTTTGGTCCCTAGGTTCCTAGCAGGTATATTGTCCAGAGAT
TATGAAATTATAAACTTTTATTTAATGAGAGGCTCTGAGAATTGAGTTCATGGAACC
AAATAAACAACACAGCTCCTGACATTGTCATTCACTAACCCAAGCATACATGAAAAA
ACAATCCCCCTGCTCC
>Olfr371 BUB/BnJ
CATTTTACAACATATTTCTCTATTTCCTTTTAAAGTGCTTAGTTTTGTAATGACCCT
TGGGGGAGGCAGCACAGTTTCTGAACTCAATAACCACAAGGGAACAAAACTGATTGG
AACTTTGCCATTAATGTTTTGGTCCCTAGGTTCCTAGCAGGTATATTGTCCAGAGAT
TATGAAATTATAAACTTTTATTTAATGAGAGGCTCTGAGAATTGAGTTCATGGAACC
AAATAAACAACACAGCTCCTGACATTGTCATTCACTAACCCAAGCATACATGAAAAA
ACAATCCCCCTGCTCC
>Olfr371 C3H/HeH
CATTTTACAACATATTTCTCTATTTCCTTTTAAAGTGCTTAGTTTTGTAATGACCCT
TGGGGGAGGCAGCACAGTTTCTGAACTCAATAACCACAAGGGAACAAAACTGATTGG
AACTTTGCCATTAATGTTTTGGTCCCTAGGTTCCTAGCAGGTATATTGTCCAGAGAT
TATGAAATTATAAACTTTTATTTAATGAGAGGCTCTGAGAATTGAGTTCATGGAACC
AAATAAACAACACAGCTCCTGACATTGTCATTCACTAACCCAAGCATACATGAAAAA
ACAATCCCCCTGCTCC
>Olfr371 C3H/HeJ
CATTTTACAACATATTTCTCTATTTCCTTTTAAAGTGCTTAGTTTTGTAATGACCCT
TGGGGGAGGCAGCACAGTTTCTGAACTCAATAACCACAAGGGAACAAAACTGATTGG
AACTTTGCCATTAATGTTTTGGTCCCTAGGTTCCTAGCAGGTATATTGTCCAGAGAT
TATGAAATTATAAACTTTTATTTAATGAGAGGCTCTGAGAATTGAGTTCATGGAACC
AAATAAACAACACAGCTCCTGACATTGTCATTCACTAACCCAAGCATACATGAAAAA
ACAATCCCCCTGCTCC
>Olfr371 C57BL/10J

CATTTTACAACATATTTCTCTATTTCCTTTTAAAGTGCTTAGTTTTGTAATGACCCT
TGGGGGAGGCAGCACAGTTTCTGAACTCAATAACCACAAGGGAACAAAACTGATTGG
AACTTTGCCATTAATGTTTTGGTCCCTAGGTTCCTAGCAGGTATATTGTCCAGAGAT
TATGAAATTATAAACTTTTATTTAATGAGAGGCTCTGAGAATTGAGTTCATGGAACC
AAATAAACAACACAGCTCCTGACATTGTCATTCACTAACCCAAGCATACATGAAAAA
ACAATCCCCCTGCTCC
>Olfr371 C57BL/6NJ
CATTTTACAACATATTTCTCTATTTCCTTTTAAAGTGCTTAGTTTTGTAATGACCCT
TGGGGGAGGCAGCACAGTTTCTGAACTCAATAACCACAAGGGAACAAAACTGATTGG
AACTTTGCCATTAATGTTTTGGTCCCTAGGTTCCTAGCAGGTATATTGTCCAGAGAT
TATGAAATTATAAACTTTTATTTAATGAGAGGCTCTGAGAATTGAGTTCATGGAACC
AAATAAACAACACAGCTCCTGACATTGTCATTCACTAACCCAAGCATACATGAAAAA
ACAATCCCCCTGCTCC
>Olfr371 C57BR/cdJ
CATTTTACAACATATTTCTCTATTTCCTTTTAAAGTGCTTAGTTTTGTAATGACCCT
TGGGGGAGGCAGCACAGTTTCTGAACTCAATAACCACAAGGGAACAAAACTGATTGG
AACTTTGCCATTAATGTTTTGGTCCCTAGGTTCCTAGCAGGTATATTGTCCAGAGAT
TATGAAATTATAAACTTTTATTTAATGAGAGGCTCTGAGAATTGAGTTCATGGAACC
AAATAAACAACACAGCTCCTGACATTGTCATTCACTAACCCAAGCATACATGAAAAA
ACAATCCCCCTGCTCC
>Olfr371 C57L/J
CATTTTACAACATATTTCTCTATTTCCTTTTAAAGTGCTTAGTTTTGTAATGACCCT
TGGGGGAGGCAGCACAGTTTCTGAACTCAATAACCACAAGGGAACAAAACTGATTGG
AACTTTGCCATTAATGTTTTGGTCCCTAGGTTCCTAGCAGGTATATTGTCCAGAGAT
TATGAAATTATAAACTTTTATTTAATGAGAGGCTCTGAGAATTGAGTTCATGGAACC
AAATAAACAACACAGCTCCTGACATTGTCATTCACTAACCCAAGCATACATGAAAAA
ACAATCCCCCTGCTCC
>Olfr371 C58/J
CATTTTACAACATATTTCTCTATTTCCTTTTAAAGTGCTTAGTTTTGTAATGACCCT
TGGGGGAGGCAGCACAGTTTCTGAACTCAATAACCACAAGGGAACAAAACTGATTGG
AACTTTGCCATTAATGTTTTGGTCCCTAGGTTCCTAGCAGGTATATTGTCCAGAGAT
TATGAAATTATAAACTTTTATTTAATGAGAGGCTCTGAGAATTGAGTTCATGGAACC
AAATAAACAACACAGCTCCTGACATTGTCATTCACTAACCCAAGCATACATGAAAAA
ACAATCCCCCTGCTCC
>Olfr371 CAST/EiJ
CATTTTACAACATATTTCTCTATTTCCTTTTAAAGTGTTTAGTTTTGTAATGACCCT
TGGGGGAGGCAGCACAGTTTCTGAACTCAATAACCACAAGGGAACAAAACTGATTGG
AACTTTGCCATTAATGTTTTGGTCCCTAGGTTCCTAGCAGGTATATTGTCCAGAGAT
TATGAAATTATAAACTTTTATTTAATGAGAGGCTCTGAGAATTGAGTTCATGGAACC
AAATAAACAACACAGCTCCTGACATTGTCATTCACTAACCCAAGCATACATGAAAAA
ACAATCCCCCTGCTCC
>Olfr371 CBA/J
CATTTTACAACATATTTCTCTATTTCCTTTTAAAGTGCTTAGTTTTGTAATGACCCT
TGGGGGAGGCAGCACAGTTTCTGAACTCAATAACCACAAGGGAACAAAACTGATTGG
AACTTTGCCATTAATGTTTTGGTCCCTAGGTTCCTAGCAGGTATATTGTCCAGAGAT
TATGAAATTATAAACTTTTATTTAATGAGAGGCTCTGAGAATTGAGTTCATGGAACC
AAATAAACAACACAGCTCCTGACATTGTCATTCACTAACCCAAGCATACATGAAAAA
ACAATCCCCCTGCTCC
>Olfr371 DBA/1J
CATTTTACAACATATTTCTCTATTTCCTTTTAAAGTGCTTAGTTTTGTAATGACCCT
TGGGGGAGGCAGCACAGTTTCTGAACTCAATAACCACAAGGGAACAAAACTGATTGG

AACTTTGCCATTAATGTTTTGGTCCCTAGGTTCCTAGCAGGTATATTGTCCAGAGAT
TATGAAATTATAAACTTTTATTTAATGAGAGGCTCTGAGAATTGAGTTCATGGAACC
AAATAAACAACACAGCTCCTGACATTGTCATTCACTAACCCAAGCATACATGAAAAA
ACAATCCCCCTGCTCC
>Olfr371 DBA/2J
CATTTTACAACATATTTCTCTATTTCCTTTTAAAGTGCTTAGTTTTGTAATGACCCT
TGGGGGAGGCAGCACAGTTTCTGAACTCAATAACCACAAGGGAACAAAACTGATTGG
AACTTTGCCATTAATGTTTTGGTCCCTAGGTTCCTAGCAGGTATATTGTCCAGAGAT
TATGAAATTATAAACTTTTATTTAATGAGAGGCTCTGAGAATTGAGTTCATGGAACC
AAATAAACAACACAGCTCCTGACATTGTCATTCACTAACCCAAGCATACATGAAAAA
ACAATCCCCCTGCTCC
>Olfr371 FVB/NJ
CATTTTACAACATATTTCTCTATTTCCTTTTAAAGTGCTTAGTTTTGTAATGACCCT
TGGGGGAGGCAGCACAGTTTCTGAACTCAATAACCACAAGGGAACAAAACTGATTGG
AACTTTGCCATTAATGTTTTGGTCCCTAGGTTCCTAGCAGGTATATTGTCCAGAGAT
TATGAAATTATAAACTTTTATTTAATGAGAGGCTCTGAGAATTGAGTTCATGGAACC
AAATAAACAACACAGCTCCTGACATTGTCATTCACTAACCCAAGCATACATGAAAAA
ACAATCCCCCTGCTCC
>Olfr371 I/LnJ
CATTTTACAACATATTTCTCTATTTCCTTTTAAAGTGCTTAGTTTTGTAATGACCCT
TGGGGGAGGCAGCACAGTTTCTGAACTCAATAACCACAAGGGAACAAAACTGATTGG
AACTTTGCCATTAATGTTTTGGTCCCTAGGTTCCTAGCAGGTATATTGTCCAGAGAT
TATGAAATTATAAACTTTTATTTAATGAGAGGCTCTGAGAATTGAGTTCATGGAACC
AAATAAACAACACAGCTCCTGACATTGTCATTCACTAACCCAAGCATACATGAAAAA
ACAATCCCCCTGCTCC
>Olfr371 KK/HiJ
CATTTTACAACATATTTCTCTATTTCCTTTTAAAGTGCTTAGTTTTGTAATGACCCT
TGGGGGAGGCAGCACAGTTTCTGAACTCAATAACCACAAGGGAACAAAACTGATTGG
AACTTTGCCATTAATGTTTTGGTCCCTAGGTTCCTAGCAGGTATATTGTCCAGAGAT
TATGAAATTATAAACTTTTATTTAATGAGAGGCTCTGAGAATTGAGTTCATGGAACC
AAATAAACAACACAGCTCCTGACATTGTCATTCACTAACCCAAGCATACATGAAAAA
ACAATCCCCCTGCTCC
>Olfr371 LEWES/EiJ
CATTTTACAACATATTTCTCTATTTCCTTTTAAAGTGCTTAGTTTTGTAATGACCCT
TGGGGGATGCAGCACAGTTTCTGAACTCAATAACCACAAGGGAACAAAACTGATTGG
AACTTTGCCATTAATGTTTTGGTCCCTAGGTTCCTAGCAGGTATATTGTCCAGAGAT
TATGAAATTATAAACTTTTATTTAATGAGAGGCTCTGAGAATTGAGTTCATGGAACC
AAATAAACAACACAGCTCCTGACATTGTCATTCACTAACCCAAGCATACATGAAAAA
ACAATCCCCCTGCTCC
>Olfr371 LP/J
CATTTTACAACATATTTCTCTATTTCCTTTTAAAGTGCTTAGTTTTGTAATGATCCT
TGGGGGAGGCAGCACAGTTTCTGAACTCAATAACCACAAGGGAACAAAACTGATTGG
AACTTTGCCATTAATGTTTTGGTCCCTAGGTTCCTAGCAGGTATATTGTCCAGAGAT
TATGAAATTATAAACTTTTATTTAATGAGAGGCTCTGAGAATTGAGTTCATGGAACC
AAATAAACAACACAGCTCCTGACATTGTCATTCACTAACCCAAGCATACATGAAAAA
ACAATCCCCCTGCTCC
>Olfr371 MOLF/EiJ
CATTTTACAACATATTTCTCTATTTCCTTTTAAAGTGTTTAGTTTTGTAATGACCCT
TGGGGGAGGCAGCACAGTTTCTGAACTCAATAACCACAAGGGAACAAAACTGATTGG
AACTTTGCCATTAATGTTTTGGTCCCTAGGTTCCTAGCAGGTATATTGTCCAGAGAT
TATGAAATTATAAACTTTTATTTAATGAGAGGCTCTGAGAATTGAGTTCATGGAACC

AAATAAACAACACAGCTCCTGACATTGTCATTCACTAACCCAAGCATACATGAAAAA
ACAATCCCCCTGCTCC
>Olfr371 NOD/ShiLtJ
CATTTTACAACATATTTCTCTATTTCCTTTTAAAGTGCTTAGTTTTGTAATGACCCT
TGGGGGAGGCAGCACAGTTTCTGAACTCAATAACCACAAGGGAACAAAACTGATTGG
AACTTTGCCATTAATGTTTTGGTCCCTAGGTTCCTAGCAGGTATATTGTCCAGAGAT
TATGAAATTATAAACTTTTATTTAATGAGAGGCTCTGAGAATTGAGTTCATGGAACC
AAATAAACAACACAGCTCCTGACATTGTCATTCACTAACCCAAGCATACATGAAAAA
ACAATCCCCCTGCTCC
>Olfr371 NZB/B1NJ
CATTTTACAACATATTTCTCTATTTCCTTTTAAAGTGCTTAGTTTTGTAATGACCCT
TGGGGGAGGCAGCACAGTTTCTGAACTCAATAACCACAAGGGAACAAAACTGATTGG
AACTTTGCCATTAATGTTTTGGTCCCTAGGTTCCTAGCAGGTATATTGTCCAGAGAT
TATGAAATTATAAACTTTTATTTAATGAGAGGCTCTGAGAATTGAGTTCATGGAACC
AAATAAACAACACAGCTCCTGACATTGTCATTCACTAACCCAAGCATACATGAAAAA
ACAATCCCCCTGCTCC
>Olfr371 NZO/HlLtJ
CATTTTACAACATATTTCTCTATTTCCTTTTAAAGTGCTTAGTTTTGTAATGACCCT
TGGGGGAGGCAGCACAGTTTCTGAACTCAATAACCACAAGGGAACAAAACTGATTGG
AACTTTGCCATTAATGTTTTGGTCCCTAGGTTCCTAGCAGGTATATTGTCCAGAGAT
TATGAAATTATAAACTTTTATTTAATGAGAGGCTCTGAGAATTGAGTTCATGGAACC
AAATAAACAACACAGCTCCTGACATTGTCATTCACTAACCCAAGCATACATGAAAAA
ACAATCCCCCTGCTCC
>Olfr371 NZW/LacJ
CATTTTACAACATATTTCTCTATTTCCTTTTAAAGTGCTTAGTTTTGTAATGACCCT
TGGGGGAGGCAGCACAGTTTCTGAACTCAATAACCACAAGGGAACAAAACTGATTGG
AACTTTGCCATTAATGTTTTGGTCCCTAGGTTCCTAGCAGGTATATTGTCCAGAGAT
TATGAAATTATAAACTTTTATTTAATGAGAGGCTCTGAGAATTGAGTTCATGGAACC
AAATAAACAACACAGCTCCTGACATTGTCATTCACTAACCCAAGCATACATGAAAAA
ACAATCCCCCTGCTCC
>Olfr371 PWK/PhJ
CATTTTACAACATATTTCTCTATTTCCTTTTAAAGTGCTTAGTTTTGTAATGACCCT
TGGGGGAGGCAGCACAGTTTCTGAACTCAATAACCACAAGGGAACAAAACTGATTGG
AACTTTGCCATTAATGTTTTGGTCCCTAGGTTCCTAGCAGGTATATTGTCCAGAGAT
TATGAAATTATAAACTTTTATTTAATGAGAGGCTCTGAGAATTGAGTTCATGGAACC
AAATAAACAACACAGCTCCTGACATTGTCATTCACTAACCCAAGCATACATGAAAAA
ACAATCCCCCTGCTCC
>Olfr371 RF/J
CATTTTACAACATATTTCTCTATTTCCTTTTAAAGTGCTTAGTTTTGTAATGATCCT
TGGGGGAGGCAGCACAGTTTCTGAACTCAATAACCACAAGGGAACAAAACTGATTGG
AACTTTGCCATTAATGTTTTGGTCCCTAGGTTCCTAGCAGGTATATTGTCCAGAGAT
TATGAAATTATAAACTTTTATTTAATGAGAGGCTCTGAGAATTGAGTTCATGGAACC
AAATAAACAACACAGCTCCTGACATTGTCATTCACTAACCCAAGCATACATGAAAAA
ACAATCCCCCTGCTCC
>Olfr371 SEA/GnJ
CATTTTACAACATATTTCTCTATTTCCTTTTAAAGTGCTTAGTTTTGTAATGATCCT
TGGGGGAGGCAGCACAGTTTCTGAACTCAATAACCACAAGGGAACAAAACTGATTGG
AACTTTGCCATTAATGTTTTGGTCCCTAGGTTCCTAGCAGGTATATTGTCCAGAGAT
TATGAAATTATAAACTTTTATTTAATGAGAGGCTCTGAGAATTGAGTTCATGGAACC
AAATAAACAACACAGCTCCTGACATTGTCATTCACTAACCCAAGCATACATGAAAAA
ACAATCCCCCTGCTCC

>Olfr371 SPRET/EiJ
CATTTTACAACATATTTCTCTATTTCATTTTAAAGTGCTTATGAATAGTTTTGTAAT
GACCCTTGGGGGAGGCAGCACAGTTTCTGAACTCAATAACCACAAGAGAACAAAACT
GATTGGAACTTTGCCATTAATGTTTTGGTCCCTAGGTTCCTAGCAGGTATATTGTCC
AGAGATTATGAAATTATAAACTTTTATTTAATGAGAGGCTCTGAGAATTGAGTTCAC
GGAACCAAATAAACAACACAGCACCTGGCATTGTCATTCACTAACCCAAGCATACAT
GAAAAAACAATCCCCCTGCTCC
>Olfr371 ST/bJ
CATTTTACAACATATTTCTCTATTTCCTTTTAAAGTGCTTAGTTTTGTAATGACCCT
TGGGGGAGGCAGCACAGTTTCTGAACTCAATAACCACAAGGGAACAAAACTGATTGG
AACTTTGCCATTAATGTTTTGGTCCCTAGGTTCCTAGCAGGTATATTGTCCAGAGAT
TATGAAATTATAAACTTTTATTTAATGAGAGGCTCTGAGAATTGAGTTCATGGAACC
AAATAAACAACACAGCTCCTGACATTGTCATTCACTAACCCAAGCATACATGAAAAA
ACAATCCCCCTGCTCC
>Olfr371 WSB/EiJ
CATTTTACAACATATTTCTCTATTTCCTTTTAAAGTGCTTAGTTTTGTAATGATCCT
TGGGGGAGGCAGCACAGTTTCTGAACTCAATAACCACAAGGGAACAAAACTGATTGG
AACTTTGCCATTAATGTTTTGGTCCCTAGGTTCCTAGCAGGTATATTGTCCAGAGAT
TATGAAATTATAAACTTTTATTTAATGAGAGGCTCTGAGAATTGAGTTCATGGAACC
AAATAAACAACACAGCTCCTGACATTGTCATTCACTAACCCAAGCATACATGAAAAA
ACAATCCCCCTGCTCC
>Olfr371 ZALENDE/EiJ
CATTTTACAACATATTTCTCTATTTCCTTTTAAAGTGCTTAGTTTTGTAATGATCCT
TGGGGGAGGCAGCACAGTTTCTGAACTCAATAACCACAAGGGAACAAAACTGATTGG
AACTTTGCCATTAATGTTTTGGTCCCTAGGTTCCTAGCAGGTATATTGTCCAGAGAT
TATGAAATTATAAACTTTTATTTAATGAGAGGCTCTGAGAATTGAGTTCATGGAACC
AAATAAACAACACAGCTCCTGACATTGTCATTCACTAACCCAAGCATACATGAAAAA
ACAATCCCCCTGCTCC

>Olfr466r C57BL/6J
TTCAAATCTTTACTCTAGAACTTCCTTGTGCCACTTTTCCAGTTGCCTGAGCGATCA
AACTCTTCTTGTTAAAACAGAACCTTAGGGAATAAATCTCTGATGACCAACACCATG
GGAGCTATATATTCTTTATTCCTATCTCCTGAAAAATTGGGCTGTGGAAATTTTGTG
TGTGCATAATTTACTGCAGAGTTGAAGAGGAACTCAAGGCTCAGCTAGAAAGGGAAG
GAAAGATACTACATATCTAGGTGAGGTCTATCTATTTTATAGAAAATATAACGTTTT
CTCAAAGACACTGTGA
>Olfr466r Wild_consensus
TTCAAATCTTTACTCTAGAACTTCCTTGTGCCACTTTTCCAGTTGCCTGAGCGATCA
AACTCTTCTTGTTAAAACAGAACCTTAGGGAATAAATCTCTGATGACCAACACCATG
GGAGCTATATATTCTTTATTCCTATCTCCTGAAAAATTGGGCTGTGGAAATTTTGTG
TGTGCATAATTTACTGCAGAGTTGAAGAGGAACTCAAGGCTCAGCTAGAAAGGGAAG
GAAAGATACTACATATCTAGGTGAGGTCTATCTATTTTATAGAAAATATAACGTTTT
CTCAAAGACACTGTGG
>Olfr466r France
TTCAAATCTTTACTCTAGAACTTCCTTGTGCCACTTTTCCAGTTGCCTGAGCGATCA
AACTCTTCTTGTTAAAACAGAACCTTAGGGAATAAATCTCTGATGACCAACACCATG
GGAGCTATATATTCTTTATTCCTATCTCCTGAAAAATTGGGCTGTGGAAATTTTGTG
TGTGCATAATTTACTGCAGAGTTGAAGAGGAACTCAAGGCTCAGCTAGAAAGGGAAG
GAAAGATACTACATATCTAGGTGAGGTCTATCTATTTTATAGAAAATATAACGTTTT
CTCAAAGACACTGTGG
>Olfr466r Germany

TTCAAATCTTTACTCTAGAACTTCCTTGTGCCACTTTTCCAGTTGCCTGAGCGATCA
AACTCTTCTTGTTAAAACAGAACCTTAGGGAATAAATCTCTGATGACCAACACCATG
GGAGCTATATATTCTTTATTCCTATCTCCTGAAAAATTGGGCTGTGGAAATTTTGTG
TGTGCATAATTTACTGCAGAGTTGAAGAGGAACTCAAGGCTCAGCTAGAAAGGGAAG
GAAAGATACTACATATCTAGGTGAGGTCTATCTATTTTATAGAAAATATAACGTTTT
CTCAAAGACACTGTGG
>Olfr466r Iran
TTCAAATCTTTACTCTAGAACTTCCTTGTGCCACTTTTCCAGTTGCCTGAGCGATCA
AACTCTTCTTGTTAAAACAGAACCTTAGGGAATAAATCTCTGATGACCAACACCATG
GGAGCTATATATTCTTTATTCCTATCTCCTGAAAAATTGGGCTGTGGAAATTTTGTG
TGTGCATAATTTACTGCAGAGTTGAAGAGGAACTCAAGGCTCAGCTAGAAAGGGAAG
GAAAGATACTACATATCTAGGTGAGGTCTATCTATTTTATAGAAAATATAACGTTTT
CTCAAAGACACTGTGA
>Olfr466r 129P2/OlaHsd
TTCAAATCTTTACTCTAGAACTTCCTTGTGCCACTTTTCCAGTTGCCTGAGCGATCA
AACTCTTCTTGTTAAAACAGAACCTTAGGGAATAAATCTCTGATGACCAACACCATG
GGAGCTATATATTCTTTATTCCTATCTCCTGAAAAATTGGGCTGTGGAAATTTTGTG
TGTGCATAATTTACTGCAGAGTTGAAGAGGAACTCAAGGCTCAGCTAGAAAGGGAAG
GAAAGATACTACATATCTAGGTGAGGTCTATTTTATAGAAAATATAACGTTTTCTCA
AAGACACTGTGG
>Olfr466r 129S1/SvImJ
TTCAAATCTTTACTCTAGAACTTCCTTGTGCCACTTTTCCAGTTGCCTGAGCGATCA
AACTCTTCTTGTTAAAACAGAACCTTAGGGAATAAATCTCTGATGACCAACACCATG
GGAGCTATATATTCTTTATTCCTATCTCCTGAAAAATTGGGCTGTGGAAATTTTGTG
TGTGCATAATTTACTGCAGAGTTGAAGAGGAACTCAAGGCTCAGCTAGAAAGGGAAG
GAAAGATACTACATATCTAGGTGAGGTCTATTTTATAGAAAATATAACGTTTTCTCA
AAGACACTGTGG
>Olfr466r 129S5SvEvBrd
TTCAAATCTTTACTCTAGAACTTCCTTGTGCCACTTTTCCAGTTGCCTGAGCGATCA
AACTCTTCTTGTTAAAACAGAACCTTAGGGAATAAATCTCTGATGACCAACACCATG
GGAGCTATATATTCTTTATTCCTATCTCCTGAAAAATTGGGCTGTGGAAATTTTGTG
TGTGCATAATTTACTGCAGAGTTGAAGAGGAACTCAAGGCTCAGCTAGAAAGGGAAG
GAAAGATACTACATATCTAGGTGAGGTCTATTTTATAGAAAATATAACGTTTTCTCA
AAGACACTGTGG
>Olfr466r A/J
TTCAAATCTTTACTCTAGAACTTCCTTGTGCCACTTTTCCAGTTGCCTGAGCGATCA
AACTCTTCTTGTTAAAACAGAACCTTAGGGAATAAATCTCTGATGACCAACACCATG
GGAGCTATATATTCTTTATTCCTATCTCCTGAAAAATTGGGCTGTGGAAATTTTGTG
TGTGCATAATTTACTGCAGAGTTGAAGAGGAACTCAAGGCTCAGCTAGAAAGGGAAG
GAAAGATACTACATATCTAGGTGAGGTCTATTTTATAGAAAATATAACGTTTTCTCA
AAGACACTGTGG
>Olfr466r AKR/J
TTCAAATCTTTACTCTAGAACTTCCTTGTGCCACTTTTCCAGTTGCCTGAGCGATCA
AACTCTTCTTGTTAAAACAGAACCTTAGGGAATAAATCTCTGATGACCAACACCATG
GGAGCTATATATTCTTTATTCCTATCTCCTGAAAAATTGGGCTGTGGAAATTTTGTG
TGTGCATAATTTACTGCAGAGTTGAAGAGGAACTCAAGGCTCAGCTAGAAAGGGAAG
GAAAGATACTACATATCTAGGTGAGGTCTATTTTATAGAAAATATAACGTTTTCTCA
AAGACACTGTGG
>Olfr466r BALB/cJ
TTCAAATCTTTACTCTAGAACTTCCTTGTGCCACTTTTCCAGTTGCCTGAGCGATCA
AACTCTTCTTGTTAAAACAGAACCTTAGGGAATAAATCTCTGATGACCAACACCATG

GGAGCTATATATTCTTTATTCCTATCTCCTGAAAAATTGGGCTGTGGAAATTTTGTG
TGTGCATAATTTACTGCAGAGTTGAAGAGGAACTCAAGGCTCAGCTAGAAAGGGAAG
GAAAGATACTACATATCTAGGTGAGGTCTATTTTATAGAAAATATAACGTTTTCTCA
AAGACACTGTGG
>Olfr466r BTBR/T_+_Itpr3tf/J
TTCAAATCTTTACTCTAGAACTTCCTTGTGCCACTTTTCCAGTTGCCTGAGCGATCA
AACTCTTCTTGTTAAAACAGAACCTTAGGGAATAAATCTCTGATGACCAACACCATG
GGAGCTATATATTCTTTATTCCTATCTCCTGAAAAATTGGGCTGTGGAAATTTTGTG
TGTGCATAATTTACTGCAGAGTTGAAGAGGAACTCAAGGCTCAGCTAGAAAGGGAAG
GAAAGATACTACATATCTAGGTGAGGTCTATTTTATAGAAAATATAACGTTTTCTCA
AAGACACTGTGG
>Olfr466r BUB/BnJ
TTCAAATCTTTACTCTAGAACTTCCTTGTGCCACTTTTCCAGTTGCCTGAGCGATCA
AACTCTTCTTGTTAAAACAGAACCTTAGGGAATAAATCTCTGATGACCAACACCATG
GGAGCTATATATTCTTTATTCCTATCTCCTGAAAAATTGGGCTGTGGAAATTTTGTG
TGTGCATAATTTACTGCAGAGTTGAAGAGGAACTCAAGGCTCAGCTAGAAAGGGAAG
GAAAGATACTACATATCTAGGTGAGGTCTATTTTATAGAAAATATAACGTTTTCTCA
AAGACACTGTGG
>Olfr466r C3H/HeH
TTCAAATCTTTACTCTAGAACTTCCTTGTGCCACTTTTCCAGTTGCCTGAGCGATCA
AACTCTTCTTGTTAAAACAGAACCTTAGGGAATAAATCTCTGATGACCAACACCATG
GGAGCTATATATTCTTTATTCCTATCTCCTGAAAAATTGGGCTGTGGAAATTTTGTG
TGTGCATAATTTACTGCAGAGTTGAAGAGGAACTCAAGGCTCAGCTAGAAAGGGAAG
GAAAGATACTACATATCTGGGTGAGGTCTATTTTATAGAAAATATAACGTTTTCTCA
AAGACACTGTGG
>Olfr466r C3H/HeJ
TTCAAATCTTTACTCTAGAACTTCCTTGTGCCACTTTTCCAGTTGCCTGAGCGATCA
AACTCTTCTTGTTAAAACAGAACCTTAGGGAATAAATCTCTGATGACCAACACCATG
GGAGCTATATATTCTTTATTCCTATCTCCTGAAAAATTGGGCTGTGGAAATTTTGTG
TGTGCATAATTTACTGCAGAGTTGAAGAGGAACTCAAGGCTCAGCTAGAAAGGGAAG
GAAAGATACTACATATCTGGGTGAGGTCTATTTTATAGAAAATATAACGTTTTCTCA
AAGACACTGTGG
>Olfr466r C57BL/10J
TTCAAATCTTTACTCTAGAACTTCCTTGTGCCACTTTTCCAGTTGCCTGAGCGATCA
AACTCTTCTTGTTAAAACAGAACCTTAGGGAATAAATCTCTGATGACCAACACCATG
GGAGCTATATATTCTTTATTCCTATCTCCTGAAAAATTGGGCTGTGGAAATTTTGTG
TGTGCATAATTTACTGCAGAGTTGAAGAGGAACTCAAGGCTCAGCTAGAAAGGGAAG
GAAAGATACTACATATCTAGGTGAGGTCTATCTATTTTATAGAAAATATAACGTTTT
CTCAAAGACACTGTGA
>Olfr466r C57BL/6NJ
TTCAAATCTTTACTCTAGAACTTCCTTGTGCCACTTTTCCAGTTGCCTGAGCGATCA
AACTCTTCTTGTTAAAACAGAACCTTAGGGAATAAATCTCTGATGACCAACACCATG
GGAGCTATATATTCTTTATTCCTATCTCCTGAAAAATTGGGCTGTGGAAATTTTGTG
TGTGCATAATTTACTGCAGAGTTGAAGAGGAACTCAAGGCTCAGCTAGAAAGGGAAG
GAAAGATACTACATATCTAGGTGAGGTCTATCTATTTTATAGAAAATATAACGTTTT
CTCAAAGACACTGTGA
>Olfr466r C57BR/cdJ
TTCAAATCTTTACTCTAGAACTTCCTTGTGCCACTTTTCCAGTTGCCTGAGCGATCA
AACTCTTCTTGTTAAAACAGAACCTTAGGGAATAAATCTCTGATGACCAACACCATG
GGAGCTATATATTCTTTATTCCTATCTCCTGAAAAATTGGGCTGTGGAAATTTTGTG
TGTGCATAATTTACTGCAGAGTTGAAGAGGAACTCAAGGCTCAGCTAGAAAGGGAAG

GAAAGATACTACATATCTAGGTGAGGTCTATCTATTTTATAGAAAATATAACGTTTT
CTCAAAGACACTGTGA
>Olfr466r C57L/J
TTCAAATCTTTACTCTAGAACTTCCTTGTGCCACTTTTCCAGTTGCCTGAGCGATCA
AACTCTTCTTGTTAAAACAGAACCTTAGGGAATAAATCTCTGATGACCAACACCATG
GGAGCTATATATTCTTTATTCCTATCTCCTGAAAAATTGGGCTGTGGAAATTTTGTG
TGTGCATAATTTACTGCAGAGTTGAAGAGGAACTCAAGGCTCAGCTAGAAAGGGAAG
GAAAGATACTACATATCTAGGTGAGGTCTATCTATTTTATAGAAAATATAACGTTTT
CTCAAAGACACTGTGA
>Olfr466r C58/J
TTCAAATCTTTACTCTAGAACTTCCTTGTGCCACTTTTCCAGTTGCCTGAGCGATCA
AACTCTTCTTGTTAAAACAGAACCTTAGGGAATAAATCTCTGATGACCAACACCATG
GGAGCTATATATTCTTTATTCCTATCTCCTGAAAAATTGGGCTGTGGAAATTTTGTG
TGTGCATAATTTACTGCAGAGTTGAAGAGGAACTCAAGGCTCAGCTAGAAAGGGAAG
GAAAGATACTACATATCTGGGTGAGGTCTATTTTATAGAAAATATAACGTTTTCTCA
AAGACACTGTGG
>Olfr466r CAST/EiJ
TTCAAATCTTTACTCTAGAACTTCCTTGTGCCACTTTTCCAGTTGCCTGAGCGATCA
AACTCTTCTTGTTAAAACAGAACCTTAGGGAATAAATCTCTGATGACCAACACCATG
GGAGCTATATATTCTTTATTCCTATCTCCTGAAAAATTGGGCTGTGGAAATTTTGTG
TGTGCATAATTTACTGCAGAGTTGAAGAGGAACTCAAGGCTCAGCTAGAAAGGGAAG
GAAAGATACTACATATCTAGGTGAGGTCTATCTATTTTATAGAAAATATAACGTTTT
CTCAAAGACACTGTGA
>Olfr466r CBA/J
TTCAAATCTTTACTCTAGAACTTCCTTGTGCCACTTTTCCAGTTGCCTGAGCGATCA
AACTCTTCTTGTTAAAACAGAACCTTAGGGAATAAATCTCTGATGACCAACACCATG
GGAGCTATATATTCTTTATTCCTATCTCCTGAAAAATTGGGCTGTGGAAATTTTGTG
TGTGCATAATTTACTGCAGAGTTGAAGAGGAACTCAAGGCTCAGCTAGAAAGGGAAG
GAAAGATACTACATATCTGGGTGAGGTCTATTTTATAGAAAATATAACGTTTTCTCA
AAGACACTGTGG
>Olfr466r DBA/1J
TTCAAATCTTTACTCTAGAACTTCCTTGTGCCACTTTTCCAGTTGCCTGAGCGATCA
AACTCTTCTTGTTAAAACAGAACCTTAGGGAATAAATCTCTGATGACCAACACCATG
GGAGCTATATATTCTTTATTCCTATCTCCTGAAAAATTGGGCTGTGGAAATTTTGTG
TGTGCATAATTTACTGCAGAGTTGAAGAGGAACTCAAGGCTCAGCTAGAAAGGGAAG
GAAAGATACTACATATCTAGGTGAGGTCTATTTTATAGAAAATATAACGTTTTCTCA
AAGACACTGTGG
>Olfr466r DBA/2J
TTCAAATCTTTACTCTAGAACTTCCTTGTGCCACTTTTCCAGTTGCCTGAGCGATCA
AACTCTTCTTGTTAAAACAGAACCTTAGGGAATAAATCTCTGATGACCAACACCATG
GGAGCTATATATTCTTTATTCCTATCTCCTGAAAAATTGGGCTGTGGAAATTTTGTG
TGTGCATAATTTACTGCAGAGTTGAAGAGGAACTCAAGGCTCAGCTAGAAAGGGAAG
GAAAGATACTACATATCTAGGTGAGGTCTATTTTATAGAAAATATAACGTTTTCTCA
AAGACACTGTGG
>Olfr466r FVB/NJ
TTCAAATCTTTACTCTAGAACTTCCTTGTGCCACTTTTCCAGTTGCCTGAGCGATCA
AACTCTTCTTGTTAAAACAGAACCTTAGGGAATAAATCTCTGATGACCAACACCATG
GGAGCTATATATTCTTTATTCCTATCTCCTGAAAAATTGGGCTGTGGAAATTTTGTG
TGTGCATAATTTACTGCAGAGTTGAAGAGGAACTCAAGGCTCAGCTAGAAAGGGAAG
GAAAGATACTACATATCTAGGTGAGGTCTATTTTATAGAAAATATAACGTTTTCTCA
AAGACACTGTGG

>Olfr466r I/LnJ
TTCAAATCTTTACTCTAGAACTTCCTTGTGCCACTTTTCCAGTTGCCTGAGCGATCA
AACTCTTCTTGTTAAAACAGAACCTTAGGGAATAAATCTCTGATGACCAACACCATG
GGAGCTATATATTCTTTATTCCTATCTCCTGAAAAATTGGGCTGTGGAAATTTTGTG
TGTGCATAATTTACTGCAGAGTTGAAGAGGAACTCAAGGCTCAGCTAGAAAGGGAAG
GAAAGATACTACATATCTAGGTGAGGTCTATTTTATAGAAAATATAACGTTTTCTCA
AAGACACTGTGG
>Olfr466r KK/HiJ
TTCAAATCTTTACTCTAGAACTTCCTTGTGCCACTTTTCCAGTTGCCTGAGCGATCA
AACTCTTCTTGTTAAAACAGAACCTTAGGGAATAAATCTCTGATGACCAACACCATG
GGAGCTATATATTCTTTATTCCTATCTCCTGAAAAATTGGGCTGTGGAAATTTTGTG
TGTGCATAATTTACTGCAGAGTTGAAGAGGAACTCAAGGCTCAGCTAGAAAGGGAAG
GAAAGATACTACATATCTGGGTGAGGTCTATTTTATAGAAAATATAACGTTTTCTCA
AAGACACTGTGG
>Olfr466r LEWES/EiJ
TTCAAATCTTTACTCTAGAACTTCCTTGTGCCACTTTTCCAGTTGCCTGAGCGATCA
AACTCTTCTTGTTAAAACAGAACCTTAGGGAATAAATCTCTGATGACCAACACCATG
GGAGCTATATATTCTTTATTCCTATCTCCTGAAAAATTGGGCTGTGGAAATTTTGTG
TGTGCATAATTTACTGCAGAGTTGAAGAGGAACTCAAGGCTCAGCTAGAAAGGGAAG
GAAAGATACTACATATCTAGGTGAGGTCTATTTTATAGAAAATATAACGTTTTCTCA
AAGACACTGTGG
>Olfr466r LP/J
TTCAAATCTTTACTCTAGAACTTCCTTGTGCCACTTTTCCAGTTGCCTGAGCGATCA
AACTCTTCTTGTTAAAACAGAACCTTAGGGAATAAATCTCTGATGACCAACACCATG
GGAGCTATATATTCTTTATTCCTATCTCCTGAAAAATTGGGCTGTGGAAATTTTGTG
TGTGCATAATTTACTGCAGAGTTGAAGAGGAACTCAAGGCTCAGCTAGAAAGGGAAG
GAAAGATACTACATATCTAGGTGAGGTCTATTTTATAGAAAATATAACGTTTTCTCA
AAGACACTGTGG
>Olfr466r MOLF/EiJ
TTCAAATCTTTACTCTAGAACTTCCTTGTGCCACTTTTCCAGTTGCCTGAGCGATCA
AACTCTTCTTGTTAAAACAGAACCTTAGGGAATAAATCTCTGATGACCAACACCATG
GGAACTATATATTCTTTATTCCTATCTCCTGAAAAATTGGGCTGTGGAAATTTTGTG
TGTGCATAATTTACTGCAGAGTTGAAGAGGAACTCAAGGCTCAGCTAGAAAGGGAAG
GAAAGATACTACATATCTAGGTGAGGTCTATTTTACAGAAAATATAATGTTTTCTCA
AAGACTCTGTGG
>Olfr466r NOD/ShiLtJ
TTCAAATCTTTACTCTAGAACTTCCTTGTGCCACTTTTCCAGTTGCCTGAGCGATCA
AACTCTTCTTGTTAAAACAGAACCTTAGGGAATAAATCTCTGATGACCAACACCATG
GGAGCTATATATTCTTTATTCCTATCTCCTGAAAAATTGGGCTGTGGAAATTTTGTG
TGTGCATAATTTACTGCAGAGTTGAAGAGGAACTCAAGGCTCAGCTAGAAAGGGAAG
GAAAGATACTACATATCTAGGTGAGGTCTATTTTATAGAAAATATAACGTTTTCTCA
AAGACACTGTGG
>Olfr466r NZB/B1NJ
TTCAAATCTTTACTCTAGAACTTCCTTGTGCCACTTTTCCAGTTGCCTGAGCGATCA
AACTCTTCTTGTTAAAACAGAACCTTAGGGAATAAATCTCTGATGACCAACACCATG
GGAGCTATATATTCTTTATTCCTATCTCCTGAAAAATTGGGCTGTGGAAATTTTGTG
TGTGCATAATTTACTGCAGAGTTGAAGAGGAACTCAAGGCTCAGCTAGAAAGGGAAG
GAAAGATACTACATATCTGGGTGAGGTCTATTTTATAGAAAATATAACGTTTTCTCA
AAGACACTGTGG
>Olfr466r NZO/HlLtJ
TTCAAATCTTTACTCTAGAACTTCCTTGTGCCACTTTTCCAGTTGCCTGAGCGATCA

AACTCTTCTTGTTAAAACAGAACCTTAGGGAATAAATCTCTGATGACCAACACCATG
GGAGCTATATATTCTTTATTCCTATCTCCTGAAAAATTGGGCTGTGGAAATTTTGTG
TGTGCATAATTTACTGCAGAGTTGAAGAGGAACTCAAGGCTCAGCTAGAAAGGGAAG
GAAAGATACTACATATCTGGGTGAGGTCTATTTTATAGAAAATATAACGTTTTCTCA
AAGACACTGTGG
>Olfr466r NZW/LacJ
TTCAAATCTTTACTCTAGAACTTCCTTGTGCCACTTTTCCAGTTGCCTGAGCGATCA
AACTCTTCTTGTTAAAACAGAACCTTAGGGAATAAATCTCTGATGACCAACACCATG
GGAGCTATATATTCTTTATTCCTATCTCCTGAAAAATTGGGCTGTGGAAATTTTGTG
TGTGCATAATTTACTGCAGAGTTGAAGAGGAACTCAAGGCTCAGCTAGAAAGGGAAG
GAAAGATACTACATATCTAGGTGAGGTCTATTTTATAGAAAATATAACGTTTTCTCA
AAGACACTGTGG
>Olfr466r PWK/PhJ
TTCAAATCTTTACTCTAGAACTTCCTTGTGCCACTTTTCCAGTTGCCTGAGCGATCA
AACTCTTCTTGTTAAAACAGAACCTTAGGGAATAAATCTCTGATGACCAACACCATG
GGAGCTATATATTCTTTATTCCTATCTCCTGAAAAATTGGGCTGTGGAAATTTTGTG
TGTGCATAATTTACTGCAGAGTTGAAGAGGAACTCAAGGCTCAGCTAGAAAGGGAAG
GAAAGATACTACATATCTAGGTGAGGTCTATTTTATAGAAAATATAACGTTTTCTCA
AAGACACTGTGG
>Olfr466r RF/J
TTCAAATCTTTACTCTAGAACTTCCTTGTGCCACTTTTCCAGTTGCCTGAGCGATCA
AACTCTTCTTGTTAAAACAGAACCTTAGGGAATAAATCTCTGATGACCAACACCATG
GGAGCTATATATTCTTTATTCCTATCTCCTGAAAAATTGGGCTGTGGAAATTTTGTG
TGTGCATAATTTACTGCAGAGTTGAAGAGGAACTCAAGGCTCAGCTAGAAAGGGAAG
GAAAGATACTACATATCTAGGTGAGGTCTATTTTATAGAAAATATAACGTTTTCTCA
AAGACACTGTGG
>Olfr466r SEA/GnJ
TTCAAATCTTTACTCTAGAACTTCCTTGTGCCACTTTTCCAGTTGCCTGAGCGATCA
AACTCTTCTTGTTAAAACAGAACCTTAGGGAATAAATCTCTGATGACCAACACCATG
GGAGCTATATATTCTTTATTCCTATCTCCTGAAAAATTGGGCTGTGGAAATTTTGTG
TGTGCATAATTTACTGCAGAGTTGAAGAGGAACTCAAGGCTCAGCTAGAAAGGGAAG
GAAAGATACTACATATCTAGGTGAGGTCTATTTTATAGAAAATATAACGTTTTCTCA
AAGACACTGTGG
>Olfr466r SPRET/EiJ
TTCAAATCTTTACTCTAGAACTTCCTTGTGCCACTTTTCCAGTTGCCTGAGTGATCA
AACTCTTCTTGTTAAAACAGAACCTTAGGGAATAAATCTCTGATGACCAACACCATG
GGAACTATATATTCTTTATTCCTATCTCCTGAAAAATTGGGCTGTGGAAATTTTGTG
TGTGCATAATTTACTGCAGAGTTGAAGAGGAACTCAAGGCTCAGCTAGAAAGGGAAG
GAAAGATACTACATATCTAGGTGAGGTCTATTTTATAGAAAATATAACGTTTTCTCA
AAGACACTGTGA
>Olfr466r ST/bJ
TTCAAATCTTTACTCTAGAACTTCCTTGTGCCACTTTTCCAGTTGCCTGAGCGATCA
AACTCTTCTTGTTAAAACAGAACCTTAGGGAATAAATCTCTGATGACCAACACCATG
GGAGCTATATATTCTTTATTCCTATCTCCTGAAAAATTGGGCTGTGGAAATTTTGTG
TGTGCATAATTTACTGCAGAGTTGAAGAGGAACTCAAGGCTCAGCTAGAAAGGGAAG
GAAAGATACTACATATCTAGGTGAGGTCTATTTTATAGAAAATATAACGTTTTCTCA
AAGACACTGTGG
>Olfr466r WSB/EiJ
TTCAAATCTTTACTCTAGAACTTCCTTGTGCCACTTTTCCAGTTGCCTGAGCGATCA
AACTCTTCTTGTTAAAACAGAACCTTAGGGAATAAATCTCTGATGACCAACACCATG
GGAGCTATATATTCTTTATTCCTATCTCCTGAAAAATTGGGCTGTGGAAATTTTGTG

TGTGCATAATTTACTGCAGAGTTGAAGAGGAACTCAAGGCTCAGCTAGAAAGGGAAG
GAAAGATACTACATATCTAGGTGAGGTCTATTTTATAGAAAATATAACGTTTTCTCA
AAGACACTGTGG
>Olfr466r ZALENDE/EiJ
TTCAAATCTTTACTCTAGAACTTCCTTGTGCCACTTTTCCAGTTGCCTGAGCGATCA
AACTCTTCTTGTTAAAACAGAACCTTAGGGAATAAATCTCTGATGACCAACACCATG
GGAGCTATATATTCTTTATTCCTATCTCCTGAAAAATTGGGCTGTGGAAATTTTGTG
TGTGCATAATTTACTGCAGAGTTGAAGAGGAACTCAAGGCTCAGCTAGAAAGGGAAG
GAAAGATACTACATATCTAGGTGAGGTCTATTTTATAGAAAATATAACGTTTTCTCA
AAGACACTGTGG

>Olfr466p C57BL/6J
TGGTTCAGTGCTTTTGTTAGTTTCACTGTGTCTCTCTTTTGTTTCTGTTTCAATGAC
TTGTCCATTAATGAGTGAGGTGCTGAAGTATCCCACTATTATTGTGTGAGGTTTAGT
GTGTGCTTTTTTAGCTTTAGTGACATTTCTTTTAAAAATGTAGGTGTCCTTGCATTT
GGGGCATAGATGTTCAGAATTGAGACTTACTCTTGGTGGATCTTTCCTTTGATGAAT
ATATGAAGTGTCCTTCGCCATCTCGTTTGATAAGTTTTGGTTGAAAGTCTATTTTAT
TGAATATTAGAATGGC
>Olfr466p Wild_consensus
TGGTTCAGTGCTTTTGTTAGTTTCACTGTGTCTCTCTTTTGTTTCTGTTTCAATGAC
TTGTCCATTAATGAGTGAGGTGCGGAAGTATCCCACTATTATTGTGTGAGGTTTAGT
GTGTGCTTTTTTAGCTTTAGTGACATTTCTTTTAAAAATGTAGGTGTCCTTGCATTT
GGGGCATAGATGTTCAGAATTGAGACTTACTCTTGGTGGATCTTTCCTTTGATGAAT
ATATGAAGTGTCCTTCGCCATCTCGTTTGATMAGTTTTGGTTGAAAGTCTATTTTAT
TGAATATTAGAATGGC
>Olfr466p France
TGGTTCAGTGCTTTTGTTAGTTTCACTGTGTCTCTCTTTTGTTTCTGTTTCAATGAC
TTGTCCATTAATGAGTGAGGTGCGGAAGTATCCCACTATTATTGTGTGAGGTTTAGT
GTGTGCTTTTTTAGCTTTAGTGACATTTCTTTTAAAAATGTAGGTGTCCTTGCATTT
GGGGCATAGATGTTCAGAATTGAGACTTACTCTTGGTGGATCTTTCCTTTGATGAAT
ATATGAAGTGTCCTTCGCCATCTCGTTTGATAAGTTTTGGTTGAAAGTCTATTTTAT
TGAATATTAGAATGGC
>Olfr466p Germany
TGGTTCAGTGCTTTTGTTAGTTTCACTGTGTCTCTCTTTTGTTTCTGTTTCAATGAC
TTGTCCATTAATGAGTGAGGTGCGGAAGTATCCCACTATTATTGTGTGAGGTTTAGT
GTGTGCTTTTTTAGCTTTAGTGACATTTCTTTTAAAAATGTAGGTGTCCTTGCATTT
GGGGCATAGATGTTCAGAATTGAGACTTACTCTTGGTGGATCTTTCCTTTGATGAAT
ATATGAAGTGTCCTTCGCCATCTCGTTTGATCAGTTTTGGTTGAAAGTCTATTTTAT
TGAATATTAGAATGGC
>Olfr466p Iran
TGGTTCAGTGCTTTTGTTAGTTTCACTGTGTCTCTCTTTTGTTTCTGTTTCAATGAC
TTGTCCATTAATGAGTGAGGTGCGGAAGTATCCCACTATTATTGTGTGAGGTTTAGT
GTGTGCTTTTTTAGCTTTAGTGACATTTCTTTTAAAAATGTAGGTGTCCTTGCATTT
GGGGCATAGATGTTCAGAATTGAGACTTACTCTTGGTGGATCTTTCCTTTGATGAAT
ATATGAAGTGTCCTTCGCCATCTCGTTTGATAAGTTTTGGTTGAAAGTCTATTTTAT
TGAATATTAGAATGGC
>Olfr466p 129P2/OlaHsd
TGGTTCAGTGCTTTTGTTAGTTTCACTGTGTCTCTCTTTTGTTTCTGTTTCAATGAC
TTGTCCATTAATGAGTGAGGTGCGGAAGTATCCCACTATTATTGTGTGAGGTTTAGT
GTGTGCTTTTTTAGCTTTAGTGACATTTCTTTTAAAAATGTAGGTGTCCTTGCATTT
GGGGCATAGATGTTCAGAATTGAGACTTACTCTTGGTGGATCTTTCCTTTGATGAAT

ATATGAAGTGTCCTTCGCCATCTCGTTTGATCAGTTTTGGTTGAAAGTCTATTTTAT
TGAATATTAGAATGGC
>Olfr466p 129S1/SvImJ
TGGTTCAGTGCTTTTGTTAGTTTCACTGTGTCTCTCTTTTGTTTCTGTTTCAATGAC
TTGTCCATTAATGAGTGAGGTGCGGAAGTATCCCACTATTATTGTGTGAGGTTTAGT
GTGTGCTTTTTTAGCTTTAGTGACATTTCTTTTAAAAATGTAGGTGTCCTTGCATTT
GGGGCATAGATGTTCAGAATTGAGACTTACTCTTGGTGGATCTTTCCTTTGATGAAT
ATATGAAGTGTCCTTCGCCATCTCGTTTGATCAGTTTTGGTTGAAAGTCTATTTTAT
TGAATATTAGAATGGC
>Olfr466p 129S5SvEvBrd
TGGTTCAGTGCTTTTGTTAGTTTCACTGTGTCTCTCTTTTGTTTCTGTTTCAATGAC
TTGTCCATTAATGAGTGAGGTGCGGAAGTATCCCACTATTATTGTGTGAGGTTTAGT
GTGTGCTTTTTTAGCTTTAGTGACATTTCTTTTAAAAATGTAGGTGTCCTTGCATTT
GGGGCATAGATGTTCAGAATTGAGACTTACTCTTGGTGGATCTTTCCTTTGATGAAT
ATATGAAGTGTCCTTCGCCATCTCGTTTGATCAGTTTTGGTTGAAAGTCTATTTTAT
TGAATATTAGAATGGC
>Olfr466p A/J
TGGTTCAGTGCTTTTGTTAGTTTCACTGTGTCTCTCTTTTGTTTCTGTTTCAATGAC
TTGTCCATTAATGAGTGAGGTGCGGAAGTATCCCACTATTATTGTGTGAGGTTTAGT
GTGTGCTTTTTTAGCTTTAGTGACATTTCTTTTAAAAATGTAGGTGTCCTTGCATTT
GGGGCATAGATGTTCAGAATTGAGACTTACTCTTGGTGGATCTTTCCTTTGATGAAT
ATATGAAGTGTCCTTCGCCATCTCGTTTGATCAGTTTTGGTTGAAAGTCTATTTTAT
TGAATATTAGAATGGC
>Olfr466p AKR/J
TGGTTCAGTGCTTTTGTTAGTTTCACTGTGTCTCTCTTTTGTTTCTGTTTCAATGAC
TTGTCCATTAATGAGTGAGGTGCGGAAGTATCCCACTATTATTGTGTGAGGTTTAGT
GTGTGCTTTTTTAGCTTTAGTGACATTTCTTTTAAAAATGTAGGTGTCCTTGCATTT
GGGGCATAGATGTTCAGAATTGAGACTTACTCTTGGTGGATCTTTCCTTTGATGAAT
ATATGAAGTGTCCTTCGCCATCTCGTTTGATCAGTTTTGGTTGAAAGTCTATTTTAT
TGAATATTAGAATGGC
>Olfr466p BALB/cJ
TGGTTCAGTGCTTTTGTTAGTTTCACTGTGTCTCTCTTTTGTTTCTGTTTCAATGAC
TTGTCCATTAATGAGTGAGGTGCGGAAGTATCCCACTATTATTGTGTGAGGTTTAGT
GTGTGCTTTTTTAGCTTTAGTGACATTTCTTTTAAAAATGTAGGTGTCCTTGCATTT
GGGGCATAGATGTTCAGAATTGAGACTTACTCTTGGTGGATCTTTCCTTTGATGAAT
ATATGAAGTGTCCTTCGCCATCTCGTTTGATCAGTTTTGGTTGAAAGTCTATTTTAT
TGAATATTAGAATGGC
>Olfr466p BTBR/T_+_Itpr3tf/J
TGGTTCAGTGCTTTTGTTAGTTTCACTGTGTCTCTCTTTTGTTTCTGTTTCAATGAC
TTGTCCATTAATGAGTGAGGTGCGGAAGTATCCCACTATTATTGTGTGAGGTTTAGT
GTGTGCTTTTTTAGCTTTAGTGACATTTCTTTTAAAAATGTAGGTGTCCTTGCATTT
GGGGCATAGATGTTCAGAATTGAGACTTACTCTTGGTGGATCTTTCCTTTGATGAAT
ATATGAAGTGTCCTTCGCCATCTCGTTTGATCAGTTTTGGTTGAAAGTCTATTTTAT
TGAATATTAGAATGGC
>Olfr466p BUB/BnJ
TGGTTCAGTGCTTTTGTTAGTTTCACTGTGTCTCTCTTTTGTTTCTGTTTCAATGAC
TTGTCCATTAATGAGTGAGGTGCGGAAGTATCCCACTATTATTGTGTGAGGTTTAGT
GTGTGCTTTTTTAGCTTTAGTGACATTTCTTTTAAAAATGTAGGTGTCCTTGCATTT
GGGGCATAGATGTTCAGAATTGAGACTTACTCTTGGTGGATCTTTCCTTTGATGAAT
ATATGAAGTGTCCTTCGCCATCTCGTTTGATCAGTTTTGGTTGAAAGTCTATTTTAT
TGAATATTAGAATGGC

>Olfr466p C3H/HeH
TGGTTCAGTGCTTTTGTTAGTTTCACTGTGTCTCTCTTTTGTTTCTGTTTCAATGAC
TTGTCCATTAATGAGTGAGGTGCGGAAGTATCCCACTATTATTGTGTGAGGTTTAGT
GTGTGCTTTTTTAGCTTTAGTGACATTTCTTTTAAAAATGTAGGTGTCCTTGCATTT
GGGGCATAGATGTTCAGAATTGAGACTTACTCTTGGTGGATCTTTCCTTTGATGAAT
ATATGAAGTGTCCTTCGCCATCTCGTTTGATCAGTTTTGGTTGAAAGTCTATTTTAT
TGAATATTAGAATGGC
>Olfr466p C3H/HeJ
TGGTTCAGTGCTTTTGTTAGTTTCACTGTGTCTCTCTTTTGTTTCTGTTTCAATGAC
TTGTCCATTAATGAGTGAGGTGCGGAAGTATCCCACTATTATTGTGTGAGGTTTAGT
GTGTGCTTTTTTAGCTTTAGTGACATTTCTTTTAAAAATGTAGGTGTCCTTGCATTT
GGGGCATAGATGTTCAGAATTGAGACTTACTCTTGGTGGATCTTTCCTTTGATGAAT
ATATGAAGTGTCCTTCGCCATCTCGTTTGATCAGTTTTGGTTGAAAGTCTATTTTAT
TGAATATTAGAATGGC
>Olfr466p C57BL/10J
TGGTTCAGTGCTTTTGTTAGTTTCACTGTGTCTCTCTTTTGTTTCTGTTTCAATGAC
TTGTCCATTAATGAGTGAGGTGCTGAAGTATCCCACTATTATTGTGTGAGGTTTAGT
GTGTGCTTTTTTAGCTTTAGTGACATTTCTTTTAAAAATGTAGGTGTCCTTGCATTT
GGGGCATAGATGTTCAGAATTGAGACTTACTCTTGGTGGATCTTTCCTTTGATGAAT
ATATGAAGTGTCCTTCGCCATCTCGTTTGATAAGTTTTGGTTGAAAGTCTATTTTAT
TGAATATTAGAATGGC
>Olfr466p C57BL/6NJ
TGGTTCAGTGCTTTTGTTAGTTTCACTGTGTCTCTCTTTTGTTTCTGTTTCAATGAC
TTGTCCATTAATGAGTGAGGTGCTGAAGTATCCCACTATTATTGTGTGAGGTTTAGT
GTGTGCTTTTTTAGCTTTAGTGACATTTCTTTTAAAAATGTAGGTGTCCTTGCATTT
GGGGCATAGATGTTCAGAATTGAGACTTACTCTTGGTGGATCTTTCCTTTGATGAAT
ATATGAAGTGTCCTTCGCCATCTCGTTTGATAAGTTTTGGTTGAAAGTCTATTTTAT
TGAATATTAGAATGGC
>Olfr466p C57BR/cdJ
TGGTTCAGTGCTTTTGTTAGTTTCACTGTGTCTCTCTTTTGTTTCTGTTTCAATGAC
TTGTCCATTAATGAGTGAGGTGCTGAAGTATCCCACTATTATTGTGTGAGGTTTAGT
GTGTGCTTTTTTAGCTTTAGTGACATTTCTTTTAAAAATGTAGGTGTCCTTGCATTT
GGGGCATAGATGTTCAGAATTGAGACTTACTCTTGGTGGATCTTTCCTTTGATGAAT
ATATGAAGTGTCCTTCGCCATCTCGTTTGATAAGTTTTGGTTGAAAGTCTATTTTAT
TGAATATTAGAATGGC
>Olfr466p C57L/J
TGGTTCAGTGCTTTTGTTAGTTTCACTGTGTCTCTCTTTTGTTTCTGTTTCAATGAC
TTGTCCATTAATGAGTGAGGTGCTGAAGTATCCCACTATTATTGTGTGAGGTTTAGT
GTGTGCTTTTTTAGCTTTAGTGACATTTCTTTTAAAAATGTAGGTGTCCTTGCATTT
GGGGCATAGATGTTCAGAATTGAGACTTACTCTTGGTGGATCTTTCCTTTGATGAAT
ATATGAAGTGTCCTTCGCCATCTCGTTTGATAAGTTTTGGTTGAAAGTCTATTTTAT
TGAATATTAGAATGGC
>Olfr466p C58/J
TGGTTCAGTGCTTTTGTTAGTTTCACTGTGTCTCTCTTTTGTTTCTGTTTCAATGAC
TTGTCCATTAATGAGTGAGGTGCGGAAGTATCCCACTATTATTGTGTGAGGTTTAGT
GTGTGCTTTTTTAGCTTTAGTGACATTTCTTTTAAAAATGTAGGTGTCCTTGCATTT
GGGGCATAGATGTTCAGAATTGAGACTTACTCTTGGTGGATCTTTCCTTTGATGAAT
ATATGAAGTGTCCTTCGCCATCTCGTTTGATCAGTTTTGGTTGAAAGTCTATTTTAT
TGAATATTAGAATGGC
>Olfr466p CAST/EiJ
TGGTTCAGTGCTTTTGTTAGTTTCACTGTGTCTCTCTTTTGTTTCTGTTTCAATGAC

TTGTCCATTAATGAGTGAGGTGCGGAAGTATCCCACTATTATTGTGTGAGGTTTAGT
GTGTGCTTTTTTAGCTTTAGTGACATTTCTTTTAAAAATGTAGGTATCCTTGCATTT
GGGGCATAGATGTTCAGAATTGAGACTTACTCTTGGTGGATCTTTCCTTTGATGAAT
ATATGAAGTGTCCTTCGCCATCTCGTTTGATCAGTTTTGGTTGAAAGTCTATTTTAT
TGAATATTAGAATGGC
>Olfr466p CBA/J
TGGTTCAGTGCTTTTGTTAGTTTCACTGTGTCTCTCTTTTGTTTCTGTTTCAATGAC
TTGTCCATTAATGAGTGAGGTGCGGAAGTATCCCACTATTATTGTGTGAGGTTTAGT
GTGTGCTTTTTTAGCTTTAGTGACATTTCTTTTAAAAATGTAGGTGTCCTTGCATTT
GGGGCATAGATGTTCAGAATTGAGACTTACTCTTGGTGGATCTTTCCTTTGATGAAT
ATATGAAGTGTCCTTCGCCATCTCGTTTGATCAGTTTTGGTTGAAAGTCTATTTTAT
TGAATATTAGAATGGC
>Olfr466p DBA/1J
TGGTTCAGTGCTTTTGTTAGTTTCACTGTGTCTCTCTTTTGTTTCTGTTTCAATGAC
TTGTCCATTAATGAGTGAGGTGCGGAAGTATCCCACTATTATTGTGTGAGGTTTAGT
GTGTGCTTTTTTAGCTTTAGTGACATTTCTTTTAAAAATGTAGGTGTCCTTGCATTT
GGGGCATAGATGTTCAGAATTGAGACTTACTCTTGGTGGATCTTTCCTTTGATGAAT
ATATGAAGTGTCCTTCGCCATCTCGTTTGATCAGTTTTGGTTGAAAGTCTATTTTAT
TGAATATTAGAATGGC
>Olfr466p DBA/2J
TGGTTCAGTGCTTTTGTTAGTTTCACTGTGTCTCTCTTTTGTTTCTGTTTCAATGAC
TTGTCCATTAATGAGTGAGGTGCGGAAGTATCCCACTATTATTGTGTGAGGTTTAGT
GTGTGCTTTTTTAGCTTTAGTGACATTTCTTTTAAAAATGTAGGTGTCCTTGCATTT
GGGGCATAGATGTTCAGAATTGAGACTTACTCTTGGTGGATCTTTCCTTTGATGAAT
ATATGAAGTGTCCTTCGCCATCTCGTTTGATCAGTTTTGGTTGAAAGTCTATTTTAT
TGAATATTAGAATGGC
>Olfr466p FVB/NJ
TGGTTCAGTGCTTTTGTTAGTTTCACTGTGTCTCTCTTTTGTTTCTGTTTCAATGAC
TTGTCCATTAATGAGTGAGGTGCGGAAGTATCCCACTATTATTGTGTGAGGTTTAGT
GTGTGCTTTTTTAGCTTTAGTGACATTTCTTTTAAAAATGTAGGTGTCCTTGCATTT
GGGGCATAGATGTTCAGAATTGAGACTTACTCTTGGTGGATCTTTCCTTTGATGAAT
ATATGAAGTGTCCTTCGCCATCTCGTTTGATCAGTTTTGGTTGAAAGTCTATTTTAT
TGAATATTAGAATGGC
>Olfr466p I/LnJ
TGGTTCAGTGCTTTTGTTAGTTTCACTGTGTCTCTCTTTTGTTTCTGTTTCAATGAC
TTGTCCATTAATGAGTGAGGTGCGGAAGTATCCCACTATTATTGTGTGAGGTTTAGT
GTGTGCTTTTTTAGCTTTAGTGACATTTCTTTTAAAAATGTAGGTGTCCTTGCATTT
GGGGCATAGATGTTCAGAATTGAGACTTACTCTTGGTGGATCTTTCCTTTGATGAAT
ATATGAAGTGTCCTTCGCCATCTCGTTTGATCAGTTTTGGTTGAAAGTCTATTTTAT
TGAATATTAGAATGGC
>Olfr466p KK/HiJ
TGGTTCAGTGCTTTTGTTAGTTTCACTGTGTCTCTCTTTTGTTTCTGTTTCAATGAC
TTGTCCATTAATGAGTGAGGTGCGGAAGTATCCCACTATTATTGTGTGAGGTTTAGT
GTGTGCTTTTTTAGCTTTAGTGACATTTCTTTTAAAAATGTAGGTGTCCTTGCATTT
GGGGCATAGATGTTCAGAATTGAGACTTACTCTTGGTGGATCTTTCCTTTGATGAAT
ATATGAAGTGTCCTTCGCCATCTCGTTTGATCAGTTTTGGTTGAAAGTCTATTTTAT
TGAATATTAGAATGGC
>Olfr466p LEWES/EiJ
TGGTTCAGTGCTTTTGTTAGTTTCACTGTGTCTCTCTTTTGTTTCTGTTTCAATGAC
TTGTCCATTAATGAGTGAGGTGCGGAAGTATCCCACTATTATTGTGTGAGGTTTAGT
GTGTGCTTTTTTAGCTTTAGTGACATTTCTTTTAAAAATGTAGGTGTCCTTGCATTT

GGGGCATAGATGTTCAGAATTGAGACTTACTCTTGGTGGATCTTTCCTTTGATGAAT
ATATGAAGTGTCCTTCGCCATCTCGTTTGATCAGTTTTGGTTGAAAGTCTATTTTAT
TGAATATTAGAATGGC
>Olfr466p LP/J
TGGTTCAGTGCTTTTGTTAGTTTCACTGTGTCTCTCTTTTGTTTCTGTTTCAATGAC
TTGTCCATTAATGAGTGAGGTGCGGAAGTATCCCACTATTATTGTGTGAGGTTTAGT
GTGTGCTTTTTTAGCTTTAGTGACATTTCTTTTAAAAATGTAGGTGTCCTTGCATTT
GGGGCATAGATGTTCAGAATTGAGACTTACTCTTGGTGGATCTTTCCTTTGATGAAT
ATATGAAGTGTCCTTCGCCATCTCGTTTGATCAGTTTTGGTTGAAAGTCTATTTTAT
TGAATATTAGAATGGC
>Olfr466p MOLF/EiJ
TGGTTCAGTGCTTTTGTTAGTTTCACTGTGTCTCTCTTTTGTTTCTGTTTCAATGAC
TTGTCCATTAATGAGTGAGGTGCTGAAGTATCCCACTATTATTGTGTGAGGTTTAGT
GTGTGCTTTTTTAGCTTTAGTGACATTTCTTTTAAAAATGTAGGTGTCCTTGCATTT
GGGGCATAGATGTTCAGAATTGAGACTTACTCTTGGTGGATCTTTCCTTTGATGAAT
ATATGAAGTGTCCTTCGCCATCTCATTTGATAAGTTTTGGTTGAAAGTCTATTTTAT
TGAATATTAGAATGGC
>Olfr466p NOD/ShiLtJ
TGGTTCAGTGCTTTTGTTAGTTTCACTGTGTCTCTCTTTTGTTTCTGTTTCAATGAC
TTGTCCATTAATGAGTGAGGTGCGGAAGTATCCCACTATTATTGTGTGAGGTTTAGT
GTGTGCTTTTTTAGCTTTAGTGACATTTCTTTTAAAAATGTAGGTGTCCTTGCATTT
GGGGCATAGATGTTCAGAATTGAGACTTACTCTTGGTGGATCTTTCCTTTGATGAAT
ATATGAAGTGTCCTTCGCCATCTCGTTTGATCAGTTTTGGTTGAAAGTCTATTTTAT
TGAATATTAGAATGGC
>Olfr466p NZB/B1NJ
TGGTTCAGTGCTTTTGTTAGTTTCACTGTGTCTCTCTTTTGTTTCTGTTTCAATGAC
TTGTCCATTAATGAGTGAGGTGCGGAAGTATCCCACTATTATTGTGTGAGGTTTAGT
GTGTGCTTTTTTAGCTTTAGTGACATTTCTTTTAAAAATGTAGGTGTCCTTGCATTT
GGGGCATAGATGTTCAGAATTGAGACTTACTCTTGGTGGATCTTTCCTTTGATGAAT
ATATGAAGTGTCCTTCGCCATCTCGTTTGATCAGTTTTGGTTGAAAGTCTATTTTAT
TGAATATTAGAATGGC
>Olfr466p NZO/HlLtJ
TGGTTCAGTGCTTTTGTTAGTTTCACTGTGTCTCTCTTTTGTTTCTGTTTCAATGAC
TTGTCCATTAATGAGTGAGGTGCGGAAGTATCCCACTATTATTGTGTGAGGTTTAGT
GTGTGCTTTTTTAGCTTTAGTGACATTTCTTTTAAAAATGTAGGTGTCCTTGCATTT
GGGGCATAGATGTTCAGAATTGAGACTTACTCTTGGTGGATCTTTCCTTTGATGAAT
ATATGAAGTGTCCTTCGCCATCTCGTTTGATCAGTTTTGGTTGAAAGTCTATTTTAT
TGAATATTAGAATGGC
>Olfr466p NZW/LacJ
TGGTTCAGTGCTTTTGTTAGTTTCACTGTGTCTCTCTTTTGTTTCTGTTTCAATGAC
TTGTCCATTAATGAGTGAGGTGCGGAAGTATCCCACTATTATTGTGTGAGGTTTAGT
GTGTGCTTTTTTAGCTTTAGTGACATTTCTTTTAAAAATGTAGGTGTCCTTGCATTT
GGGGCATAGATGTTCAGAATTGAGACTTACTCTTGGTGGATCTTTCCTTTGATGAAT
ATATGAAGTGTCCTTCGCCATCTCGTTTGATCAGTTTTGGTTGAAAGTCTATTTTAT
TGAATATTAGAATGGC
>Olfr466p PWK/PhJ
TGGTTCAGTGCTTTTGTTAGTTTCACTGTGTCTCTCTTTTGTTTCTGTTTCAATGAC
TTGTCCATTAATGAGTGAGGTGCTGAAGTATCCCACTATTATTGTGTGAGGTTTAGT
GTGTGCTTTTTTAGCTTTAGTGACATTTCTTTTAAAAATGTAGGTGTCCTTGCATTT
GGGGCATAGATGTTCAGAATTGAGACTTACTCTTGGTGGATCTTTCCTTTGATGAAT
ATATGAAGTGTCCTTCGCCATCTCGTTTGATAAGTTTTGGTTGAAAGTCTATTTTAT

TGAATATTAGAATGGC
>Olfr466p RF/J
TGGTTCAGTGCTTTTGTTAGTTTCACTGTGTCTCTCTTTTGTTTCTGTTTCAATGAC
TTGTCCATTAATGAGTGAGGTGCGGAAGTATCCCACTATTATTGTGTGAGGTTTAGT
GTGTGCTTTTTTAGCTTTAGTGACATTTCTTTTAAAAATGTAGGTGTCCTTGCATTT
GGGGCATAGATGTTCAGAATTGAGACTTACTCTTGGTGGATCTTTCCTTTGATGAAT
ATATGAAGTGTCCTTCGCCATCTCGTTTGATCAGTTTTGGTTGAAAGTCTATTTTAT
TGAATATTAGAATGGC
>Olfr466p SEA/GnJ
TGGTTCAGTGCTTTTGTTAGTTTCACTGTGTCTCTCTTTTGTTTCTGTTTCAATGAC
TTGTCCATTAATGAGTGAGGTGCGGAAGTATCCCACTATTATTGTGTGAGGTTTAGT
GTGTGCTTTTTTAGCTTTAGTGACATTTCTTTTAAAAATGTAGGTGTCCTTGCATTT
GGGGCATAGATGTTCAGAATTGAGACTTACTCTTGGTGGATCTTTCCTTTGATGAAT
ATATGAAGTGTCCTTCGCCATCTCGTTTGATCAGTTTTGGTTGAAAGTCTATTTTAT
TGAATATTAGAATGGC
>Olfr466p SPRET/EiJ
TGGTTCAGTGCTTTTGTTAGTTTCACTGTGTCTCTCTTTTGTTTCTGTTTCAATGAC
TTGTCCATTAATGAGTGAGGTGCTGAAGTATCCCACTATTATTGTGTGAGGTTTAGT
GTGTGCATTTTTAGCTTTAGTGACATTTCTTTTTAAAATGTAGGTGTCCTTGCATTT
GGGGCATAGATGTTCAGCATTGAGACTTACTCTTGGTGGATCTTTCCTTTGATGAAT
ATATGAAGTGTCCTTCGCCATCTCGTTTGATAAGTTTTGGTTGAAAGTCTATTTTAT
TGAATATTAGAATGGC
>Olfr466p ST/bJ
TGGTTCAGTGCTTTTGTTAGTTTCACTGTGTCTCTCTTTTGTTTCTGTTTCAATGAC
TTGTCCATTAATGAGTGAGGTGCGGAAGTATCCCACTATTATTGTGTGAGGTTTAGT
GTGTGCTTTTTTAGCTTTAGTGACATTTCTTTTAAAAATGTAGGTGTCCTTGCATTT
GGGGCATAGATGTTCAGAATTGAGACTTACTCTTGGTGGATCTTTCCTTTGATGAAT
ATATGAAGTGTCCTTCGCCATCTCGTTTGATCAGTTTTGGTTGAAAGTCTATTTTAT
TGAATATTAGAATGGC
>Olfr466p WSB/EiJ
TGGTTCAGTGCTTTTGTTAGTTTCACTGTGTCTCTCTTTTGTTTCTGTTTCAATGAC
TTGTCCATTAATGAGTGAGGTGCGGAAGTATCCCACTATTATTGTGTGAGGTTTAGT
GTGTGCTTTTTTAGCTTTAGTGACATTTCTTTTAAAAATGTAGGTGTCCTTGCATTT
GGGGCATAGATGTTCAGAATTGAGACTTACTCTTGGTGGATCTTTCCTTTGATGAAT
ATATGAAGTGTCCTTCGCCATCTCGTTTGATCAGTTTTGGTTGAAAGTCTATTTTAT
TGAATATTAGAATGGC
>Olfr466p ZALENDE/EiJ
TGGTTCAGTGCTTTTGTTAGTTTCACTGTGTCTCTCTTTTGTTTCTGTTTCAATGAC
TTGTCCATTAATGAGTGAGGTGCGGAAGTATCCCACTATTATTGTGTGAGGTTTAGT
GTGTGCTTTTTTAGCTTTAGTGACATTTCTTTTAAAAATGTAGGTGTCCTTGCATTT
GGGGCATAGATGTTCAGAATTGAGACTTACTCTTGGTGGATCTTTCCTTTGATGAAT
ATATGAAGTGTCCTTCGCCATCTCGTTTGATCAGTTTTGGTTGAAAGTCTATTTTAT
TGAATATTAGAATGGC

>Olfr1402 C57BL/6J
CACCAGGGAGGAGGATAGTCAGGCTTTCAGGCTTCAGCCACAGCAATTCCCAACAAT
ACATTTAGGACTGCAATTTACTAACAAATGTCCCAAAAGAGATAGGGAGCTGTTCTC
ATTGCATTTTGGGAAAAGATGATTTCTATGACCTTAATAAATACACAAGAGAGTTAA
TTGTTCTTCATTTCTACAGCTTCTGTTTTGGCTGTTGCCTTTGATTTGTATTTGTGT
GCTAAGGCGTTTCAGACAGCTGCTGAGAGAGAGATTACTTAGGCTACTCTCTAAAGG
TTAGTCCATGAACCTT

```
>Olfr1402 Wild_consensus
CACCAGGGAGGAGGATAGTCAGGCTTTCAGGCTTCAGCCACAGCAATTCCCAACAAT
ACATTTAGGACTGCAATTTACTAACAAATGTCCCAAAAGAGATAGGGAGCTGTTCTC
ATTGCATTTTGGGAAAAGATGATTTCTATGACCTTAATAAATACACAAGAGAGTTAA
TTGTTCTTCATTTCTACAGCTTCTGTTTTGGCTGTTGCCTTTGATTTGTATTTGTGT
GCTAAGGCGTTTCAGACAGCTGCTGAGAGAGAGATTACTTAGGCTACTCTCTAAAGG
TCAGTCCATGAACCTT
>Olfr1402 France
CACCAGGGAGGAGGATAGTCAGGCTTTCAGGCTTCAGCCACAGCAATTCCCAACAAT
ACATTTAGGACTGCAATTTACTAACAAATGTCCCAAAAGAGATAGGGAGCTGTTCTC
ATTGCATTTTGGGAAAAGATGATTTCTATGACCTTAATAAATACACAAGAGAGTTAA
TTGTTCTTCATTTCTACAGCTTCTGTTTTGGCTGTTGCCTTTGATTTGTATTTGTGT
GCTAAGGCGTTTCAGACAGCTGCTGAGAGAGAGATTACTTAGGCTACTCTCTAAAGG
TCAGTCCATGAACCTT
>Olfr1402 Germany
CACCAGGGAGGAGGATAGTCAGGCTTTCAGGCTTCAGCCACAGCAATTCCCAACAAT
ACATTTAGGACTGCAATTTACTAACAAATGTCCCAAAAGAGATAGGGAGCTGTTCTC
ATTGCATTTTGGGAAAAGATGATTTCTATGACCTTAATAAATACACAAGAGAGTTAA
TTGTTCTTCATTTCTACAGCTTCTGTTTTGGCTGTTGCCTTTGATTTGTATTTGTGT
GCTAAGGCGTTTCAGACAGCTGCTGAGAGAGAGATTACTTAGGCTACTCTCTAAAGG
TCAGTCCATGAACCTT
>Olfr1402 Iran
CACCAGGGAGGAGGATAGTCAGGCTTTCAGGCTTCAGCCACAGCAATTCCCAACAAT
ACATTTAGGACTGCAATTTACTAACAAATGTCCCAAAAGAGATAGGGAGCTGTTCTC
ATTGCATTTTGGGAAAAGATGATTTCTATGACCTTAATAAATACACAAGAGAGTTAA
TTGTTCTTCATTTCTACAGCTTCTGTTTTGGCTGTTGCCTTTGATTTGTATTTGTGT
GCTAAGGCGTTTCAGACAGCTGCTGAGAGAGAGATTACTTAGGCTACTCTCTAAAGG
TCAGTCCATGAACCTT
>Olfr1402 129P2/OlaHsd
CACCAGGGAGGAGGATAGTCAGGCTTTCAGGCTTCAGCCACAGCAATTCCCAACAAT
ACATTTAGGACTGCAATTTACTAACAAATGTCCCAAAAGAGATAGGGAGCTGTTCTC
ATTGCATTTTGGGAAAAGATGATTTCTATGACCTTAATAAATACACAAGAGAGTTAA
TTGTTCTTCATTTCTACAGCTTCTGTTTTGGCTGTTGCCTTTGATTTGTATTTGTGT
GCTAAGGCGTTTCAGACAGCTGCTGAGAGAGAGATTACTTAGGCTACTCTCTAAAGG
TTAGTCCATGAACCTT
>Olfr1402 129S1/SvImJ
CACCAGGGAGGAGGATAGTCAGGCTTTCAGGCTTCAGCCACAGCAATTCCCAACAAT
ACATTTAGGACTGCAATTTACTAACAAATGTCCCAAAAGAGATAGGGAGCTGTTCTC
ATTGCATTTTGGGAAAAGATGATTTCTATGACCTTAATAAATACACAAGAGAGTTAA
TTGTTCTTCATTTCTACAGCTTCTGTTTTGGCTGTTGCCTTTGATTTGTATTTGTGT
GCTAAGGCGTTTCAGACAGCTGCTGAGAGAGAGATTACTTAGGCTACTCTCTAAAGG
TTAGTCCATGAACCTT
>Olfr1402 129S5SvEvBrd
CACCAGGGAGGAGGATAGTCAGGCTTTCAGGCTTCAGCCACAGCAATTCCCAACAAT
ACATTTAGGACTGCAATTTACTAACAAATGTCCCAAAAGAGATAGGGAGCTGTTCTC
ATTGCATTTTGGGAAAAGATGATTTCTATGACCTTAATAAATACACAAGAGAGTTAA
TTGTTCTTCATTTCTACAGCTTCTGTTTTGGCTGTTGCCTTTGATTTGTATTTGTGT
GCTAAGGCGTTTCAGACAGCTGCTGAGAGAGAGATTACTTAGGCTACTCTCTAAAGG
TTAGTCCATGAACCTT
>Olfr1402 A/J
CACCAGGGAGGAGGATAGTCAGGCTTTCAGGCTTCAGCCACAGCAATTCCCAACAAT
```

ACATTTAGGACTGCAATTTACTAACAAATGTCCCAAAAGAGATAGGGAGCTGTTCTC
ATTGCATTTTGGGAAAAGATGATTTCTATGACCTTAATAAATACACAAGAGAGTTAA
TTGTTCTTCATTTCTACAGCTTCTGTTTTGGCTGTTGCCTTTGATTTGTATTTGTGT
GCTAAGGCGTTTCAGACAGCTGCTGAGAGAGAGATTACTTAGGCTACTCTCTAAAGG
TTAGTCCATGAACCTT
>Olfr1402 AKR/J
CACCAGGGAGGAGGATAGTCAGGCTTTCAGGCTTCAGCCACAGCAATTCCCAACAAT
ACATTTAGGACTGCAATTTACTAACAAATGTCCCAAAAGAGATAGGGAGCTGTTCTC
ATTGCATTTTGGGAAAAGATGATTTCTATGACCTTAATAAATACACAAGAGAGTTAA
TTGTTCTTCATTTCTACAGCTTCTGTTTTGGCTGTTGCCTTTGATTTGTATTTGTGT
GCTAAGGCGTTTCAGACAGCTGCTGAGAGAGAGATTACTTAGGCTACTCTCTAAAGG
TTAGTCCATGAACCTT
>Olfr1402 BALB/cJ
CACCAGGGAGGAGGATAGTCAGGCTTTCAGGCTTCAGCCACAGCAATTCCCAACAAT
ACATTTAGGACTGCAATTTACTAACAAATGTCCCAAAAGAGATAGGGAGCTGTTCTC
ATTGCATTTTGGGAAAAGATGATTTCTATGACCTTAATAAATACACAAGAGAGTTAA
TTGTTCTTCATTTCTACAGCTTCTGTTTTGGCTGTTGCCTTTGATTTGTATTTGTGT
GCTAAGGCGTTTCAGACAGCTGCTGAGAGAGAGATTACTTAGGCTACTCTCTAAAGG
TTAGTCCATGAACCTT
>Olfr1402 BTBR/T_+_Itpr3tf/J
CACCAGGGAGGAGGATAGTCAGGCTTTCAGGCTTCAGCCACAGCAATTCCCAACAAT
ACATTTAGGACTGCAATTTACTAACAAATGTCCCAAAAGAGATAGGGAGCTGTTCTC
ATTGCATTTTGGGAAAAGATGATTTCTATGACCTTAATAAATACACAAGAGAGTTAA
TTGTTCTTCATTTCTACAGCTTCTGTTTTGGCTGTTGCCTTTGATTTGTATTTGTGT
GCTAAGGCGTTTCAGACAGCTGCTGAGAGAGAGATTACTTAGGCTACTCTCTAAAGG
TTAGTCCATGAACCTT
>Olfr1402 BUB/BnJ
CACCAGGGAGGAGGATAGTCAGGCTTTCAGGCTTCAGCCACAGCAATTCCCAACAAT
ACATTTAGGACTGCAATTTACTAACAAATGTCCCAAAAGAGATAGGGAGCTGTTCTC
ATTGCATTTTGGGAAAAGATGATTTCTATGACCTTAATAAATACACAAGAGAGTTAA
TTGTTCTTCATTTCTACAGCTTCTGTTTTGGCTGTTGCCTTTGATTTGTATTTGTGT
GCTAAGGCGTTTCAGACAGCTGCTGAGAGAGAGATTACTTAGGCTACTCTCTAAAGG
TTAGTCCATGAACCTT
>Olfr1402 C3H/HeH
CACCAGGGAGGAGGATAGTCAGGCTTTCAGGCTTCAGCCACAGCAATTCCCAACAAT
ACATTTAGGACTGCAATTTACTAACAAATGTCCCAAAAGAGATAGGGAGCTGTTCTC
ATTGCATTTTGGGAAAAGATGATTTCTATGACCTTAATAAATACACAAGAGAGTTAA
TTGTTCTTCATTTCTACAGCTTCTGTTTTGGCTGTTGCCTTTGATTTGTATTTGTGT
GCTAAGGCGTTTCAGACAGCTGCTGAGAGAGAGATTACTTAGGCTACTCTCTAAAGG
TTAGTCCATGAACCTT
>Olfr1402 C3H/HeJ
CACCAGGGAGGAGGATAGTCAGGCTTTCAGGCTTCAGCCACAGCAATTCCCAACAAT
ACATTTAGGACTGCAATTTACTAACAAATGTCCCAAAAGAGATAGGGAGCTGTTCTC
ATTGCATTTTGGGAAAAGATGATTTCTATGACCTTAATAAATACACAAGAGAGTTAA
TTGTTCTTCATTTCTACAGCTTCTGTTTTGGCTGTTGCCTTTGATTTGTATTTGTGT
GCTAAGGCGTTTCAGACAGCTGCTGAGAGAGAGATTACTTAGGCTACTCTCTAAAGG
TTAGTCCATGAACCTT
>Olfr1402 C57BL/10J
CACCAGGGAGGAGGATAGTCAGGCTTTCAGGCTTCAGCCACAGCAATTCCCAACAAT
ACATTTAGGACTGCAATTTACTAACAAATGTCCCAAAAGAGATAGGGAGCTGTTCTC
ATTGCATTTTGGGAAAAGATGATTTCTATGACCTTAATAAATACACAAGAGAGTTAA

TTGTTCTTCATTTCTACAGCTTCTGTTTTGGCTGTTGCCTTTGATTTGTATTTGTGT
GCTAAGGCGTTTCAGACAGCTGCTGAGAGAGAGATTACTTAGGCTACTCTCTAAAGG
TTAGTCCATGAACCTT
>Olfr1402 C57BL/6NJ
CACCAGGGAGGAGGATAGTCAGGCTTTCAGGCTTCAGCCACAGCAATTCCCAACAAT
ACATTTAGGACTGCAATTTACTAACAAATGTCCCAAAAGAGATAGGGAGCTGTTCTC
ATTGCATTTTGGGAAAAGATGATTTCTATGACCTTAATAAATACACAAGAGAGTTAA
TTGTTCTTCATTTCTACAGCTTCTGTTTTGGCTGTTGCCTTTGATTTGTATTTGTGT
GCTAAGGCGTTTCAGACAGCTGCTGAGAGAGAGATTACTTAGGCTACTCTCTAAAGG
TTAGTCCATGAACCTT
>Olfr1402 C57BR/cdJ
CACCAGGGAGGAGGATAGTCAGGCTTTCAGGCTTCAGCCACAGCAATTCCCAACAAT
ACATTTAGGACTGCAATTTACTAACAAATGTCCCAAAAGAGATAGGGAGCTGTTCTC
ATTGCATTTTGGGAAAAGATGATTTCTATGACCTTAATAAATACACAAGAGAGTTAA
TTGTTCTTCATTTCTACAGCTTCTGTTTTGGCTGTTGCCTTTGATTTGTATTTGTGT
GCTAAGGCGTTTCAGACAGCTGCTGAGAGAGAGATTACTTAGGCTACTCTCTAAAGG
TTAGTCCATGAACCTT
>Olfr1402 C57L/J
CACCAGGGAGGAGGATAGTCAGGCTTTCAGGCTTCAGCCACAGCAATTCCCAACAAT
ACATTTAGGACTGCAATTTACTAACAAATGTCCCAAAAGAGATAGGGAGCTGTTCTC
ATTGCATTTTGGGAAAAGATGATTTCTATGACCTTAATAAATACACAAGAGAGTTAA
TTGTTCTTCATTTCTACAGCTTCTGTTTTGGCTGTTGCCTTTGATTTGTATTTGTGT
GCTAAGGCGTTTCAGACAGCTGCTGAGAGAGAGATTACTTAGGCTACTCTCTAAAGG
TTAGTCCATGAACCTT
>Olfr1402 C58/J
CACCAGGGAGGAGGATAGTCAGGCTTTCAGGCTTCAGCCACAGCAATTCCCAACAAT
ACATTTAGGACTGCAATTTACTAACAAATGTCCCAAAAGAGATAGGGAGCTGTTCTC
ATTGCATTTTGGGAAAAGATGATTTCTATGACCTTAATAAATACACAAGAGAGTTAA
TTGTTCTTCATTTCTACAGCTTCTGTTTTGGCTGTTGCCTTTGATTTGTATTTGTGT
GCTAAGGCGTTTCAGACAGCTGCTGAGAGAGAGATTACTTAGGCTACTCTCTAAAGG
TTAGTCCATGAACCTT
>Olfr1402 CAST/EiJ
CACCAGGGAGGAGGATAGTCAGGCTTTCAGGCTTCAGCCACAGCAATTCTCAACAAT
ACATTTAGGACTGCAATTTACTAACAAATGTCCCAAAAGAGATAGGGAGCTGTTCTC
ATTGCATTTTGGGAAAACATGATTTCTATGACCTTAATAAATACCCAAGAGAGTTAA
TTGTTCTTCATTTCTACAGCTTCTGTTTTGGCTGTTGCCTTTGATTTGTATTTGTGT
GCTAAGGCATTTCAGACAGCTGCTGAGAGAGAGATTACTTAGGCTACTCTCTAAAGG
TCAGTCCATGAACCTT
>Olfr1402 CBA/J
CACCAGGGAGGAGGATAGTCAGGCTTTCAGGCTTCAGCCACAGCAATTCCCAACAAT
ACATTTAGGACTGCAATTTACTAACAAATGTCCCAAAAGAGATAGGGAGCTGTTCTC
ATTGCATTTTGGGAAAAGATGATTTCTATGACCTTAATAAATACACAAGAGAGTTAA
TTGTTCTTCATTTCTACAGCTTCTGTTTTGGCTGTTGCCTTTGATTTGTATTTGTGT
GCTAAGGCGTTTCAGACAGCTGCTGAGAGAGAGATTACTTAGGCTACTCTCTAAAGG
TTAGTCCATGAACCTT
>Olfr1402 DBA/1J
CACCAGGGAGGAGGATAGTCAGGCTTTCAGGCTTCAGCCACAGCAATTCCCAACAAT
ACATTTAGGACTGCAATTTACTAACAAATGTCCCAAAAGAGATAGGGAGCTGTTCTC
ATTGCATTTTGGGAAAAGATGATTTCTATGACCTTAATAAATACACAAGAGAGTTAA
TTGTTCTTCATTTCTACAGCTTCTGTTTTGGCTGTTGCCTTTGATTTGTATTTGTGT
GCTAAGGCGTTTCAGACAGCTGCTGAGAGAGAGATTACTTAGGCTACTCTCTAAAGG

TTAGTCCATGAACCTT
>Olfr1402 DBA/2J
CACCAGGGAGGAGGATAGTCAGGCTTTCAGGCTTCAGCCACAGCAATTCCCAACAAT
ACATTTAGGACTGCAATTTACTAACAAATGTCCCAAAAGAGATAGGGAGCTGTTCTC
ATTGCATTTTGGGAAAAGATGATTTCTATGACCTTAATAAATACACAAGAGAGTTAA
TTGTTCTTCATTTCTACAGCTTCTGTTTTGGCTGTTGCCTTTGATTTGTATTTGTGT
GCTAAGGCGTTTCAGACAGCTGCTGAGAGAGAGATTACTTAGGCTACTCTCTAAAGG
TTAGTCCATGAACCTT
>Olfr1402 FVB/NJ
CACCAGGGAGGAGGATAGTCAGGCTTTCAGGCTTCAGCCACAGCAATTCCCAACAAT
ACATTTAGGACTGCAATTTACTAACAAATGTCCCAAAAGAGATAGGGAGCTGTTCTC
ATTGCATTTTGGGAAAAGATGATTTCTATGACCTTAATAAATACACAAGAGAGTTAA
TTGTTCTTCATTTCTACAGCTTCTGTTTTGGCTGTTGCCTTTGATTTGTATTTGTGT
GCTAAGGCGTTTCAGACAGCTGCTGAGAGAGAGATTACTTAGGCTACTCTCTAAAGG
TTAGTCCATGAACCTT
>Olfr1402 I/LnJ
CACCAGGGAGGAGGATAGTCAGGCTTTCAGGCTTCAGCCACAGCAATTCCCAACAAT
ACATTTAGGACTGCAATTTACTAACAAATGTCCCAAAAGAGATAGGGAGCTGTTCTC
ATTGCATTTTGGGAAAAGATGATTTCTATGACCTTAATAAATACACAAGAGAGTTAA
TTGTTCTTCATTTCTACAGCTTCTGTTTTGGCTGTTGCCTTTGATTTGTATTTGTGT
GCTAAGGCGTTTCAGACAGCTGCTGAGAGAGAGATTACTTAGGCTACTCTCTAAAGG
TTAGTCCATGAACCTT
>Olfr1402 KK/HiJ
CACCAGGGAGGAGGATAGTCAGGCTTTCAGGCTTCAGCCACAGCAATTCTCAACAAT
ACATTTAGGACTGCAATTTACTAACAAATGTCCCAAAAGAGATAGGGAGCTGTTCTC
ATTGCATTTTGGGAAAACATGATTTCTATGACCTTAATAAATACACAAGAGAGTTAA
TTGTTCTTCATTTCTACAGCTTCTGTTTTGGCTGTTGCCTTTGATTTGTATTTGTGT
GCTAAGGCATTTCAGACAGCTGCTGAGAGAGAGATTACTTAGGCTACTCTCTAAAGG
TCAGTCCATGAACCTT
>Olfr1402 LEWES/EiJ
CACCAGGGAGGAGGATAGTCAGGCTTTCAGGCTTCAGCCACAGCAATTCCCAACAAT
ACATTTAGGACTGCAATTTACTAACAAATGTCCCAAAAGAGATAGGGAGCTGTTCTC
ATTGCATTTTGGGAAAAGATGATTTCTATGACCTTAATAAATACACAAGAGAGTTAA
TTGTTCTTCATTTCTACAGCTTCTGTTTTGGCTGTTGCCTTTGATTTGTATTTGTGT
GCTAAGGCGTTTCAGACAGCTGCTGAGAGAGAGATTACTTAGGCTACTCTCTAAAGG
TCAGTCCATGAACCTT
>Olfr1402 LP/J
CACCAGGGAGGAGGATAGTCAGGCTTTCAGGCTTCAGCCACAGCAATTCCCAACAAT
ACATTTAGGACTGCAATTTACTAACAAATGTCCCAAAAGAGATAGGGAGCTGTTCTC
ATTGCATTTTGGGAAAAGATGATTTCTATGACCTTAATAAATACACAAGAGAGTTAA
TTGTTCTTCATTTCTACAGCTTCTGTTTTGGCTGTTGCCTTTGATTTGTATTTGTGT
GCTAAGGCGTTTCAGACAGCTGCTGAGAGAGAGATTACTTAGGCTACTCTCTAAAGG
TTAGTCCATGAACCTT
>Olfr1402 MOLF/EiJ
CACCAGGGAGGAGGATAGTCAGGCTTTCAGGCTTCAGCCACAGCAATTCTCAACAAT
ACATTTAGGACTGCAATTTACTAACAAATGTCCCAAAAGAGATAGGGAGCTGTTCTC
ATTGCATTTTGGGAAAACATGATTTCTATGACCTTAATAAATACACAAGAGAGTTAA
TTGTTCTTCATTTCTACAGCTTCTGTTTTGGCTGTTGCCTTTGATTTGTATTTGTGT
GCTAAGGCATTTCAGACAGCTGCTGAGAGAGAGATTACTTAGGCTACTCTCTAAAGG
TCAGTCCATGAACCTT
>Olfr1402 NOD/ShiLtJ

CACCAGGGAGGAGGATAGTCAGGCTTTCAGGCTTCAGCCACAGCAATTCCCAACAAT
ACATTTAGGACTGCAATTTACTAACAAATGTCCCAAAAGAGATAGGGAGCTGTTCTC
ATTGCATTTTGGGAAAAGATGATTTCTATGACCTTAATAAATACACAAGAGAGTTAA
TTGTTCTTCATTTCTACAGCTTCTGTTTTGGCTGTTGCCTTTGATTTGTATTTGTGT
GCTAAGGCGTTTCAGACAGCTGCTGAGAGAGAGATTACTTAGGCTACTCTCTAAAGG
TCAGTCCATGAACCTT
>Olfr1402 NZB/B1NJ
CACCAGGGAGGAGGATAGTCAGGCTTTCAGGCTTCAGCCACAGCAATTCCCAACAAT
ACATTTAGGACTGCAATTTACTAACAAATGTCCCAAAAGAGATAGGGAGCTGTTCTC
ATTGCATTTTGGGAAAAGATGATTTCTATGACCTTAATAAATACACAAGAGAGTTAA
TTGTTCTTCATTTCTACAGCTTCTGTTTTGGCTGTTGCCTTTGATTTGTATTTGTGT
GCTAAGGCGTTTCAGACAGCTGCTGAGAGAGAGATTACTTAGGCTACTCTCTAAAGG
TCAGTCCATGAACCTT
>Olfr1402 NZO/HlLtJ
CACCAGGGAGGAGGATAGTCAGGCTTTCAGGCTTCAGCCACAGCAATTCCCAACAAT
ACATTTAGGACTGCAATTTACTAACAAATGTCCCAAAAGAGATAGGGAGCTGTTCTC
ATTGCATTTTGGGAAAAGATGATTTCTATGACCTTAATAAATACACAAGAGAGTTAA
TTGTTCTTCATTTCTACAGCTTCTGTTTTGGCTGTTGCCTTTGATTTGTATTTGTGT
GCTAAGGCGTTTCAGACAGCTGCTGAGAGAGAGATTACTTAGGCTACTCTCTAAAGG
TCAGTCCATGAACCTT
>Olfr1402 NZW/LacJ
CACCAGGGAGGAGGATAGTCAGGCTTTCAGGCTTCAGCCACAGCAATTCCCAACAAT
ACATTTAGGACTGCAATTTACTAACAAATGTCCCAAAAGAGATAGGGAGCTGTTCTC
ATTGCATTTTGGGAAAAGATGATTTCTATGACCTTAATAAATACACAAGAGAGTTAA
TTGTTCTTCATTTCTACAGCTTCTGTTTTGGCTGTTGCCTTTGATTTGTATTTGTGT
GCTAAGGCGTTTCAGACAGCTGCTGAGAGAGAGATTACTTAGGCTACTCTCTAAAGG
TCAGTCCATGAACCTT
>Olfr1402 PWK/PhJ
CACCAGGGAGGAGGATAGTCAGGCTTTCAGGCTTCAGCCACAGCAATTCTCAACAAT
ACATTTAGGACTGCAATTTACTAACAAATGTCCCAAAAGAGATAGGGAGCTGTTCTC
ATTGCATTTTGGGAAAACATGATTTCTATGACCTTAATAAATACACAAGAGAGTTAA
TTGTTCTTCATTTCTACAGCTTCTGTTTTGGCTGTTGCCTTTGATTTGTATTTGTGT
GCTAAGGCATTTCAGACAGCTGCTGAGAGAGAGATTACTTAGGCTACTCTCTAAAGG
TCAGTCCATGAACCTT
>Olfr1402 RF/J
CACCAGGGAGGAGGATAGTCAGGCTTTCAGGCTTCAGCCACAGCAATTCCCAACAAT
ACATTTAGGACTGCAATTTACTAACAAATGTCCCAAAAGAGATAGGGAGCTGTTCTC
ATTGCATTTTGGGAAAAGATGATTTCTATGACCTTAATAAATACACAAGAGAGTTAA
TTGTTCTTCATTTCTACAGCTTCTGTTTTGGCTGTTGCCTTTGATTTGTATTTGTGT
GCTAAGGCGTTTCAGACAGCTGCTGAGAGAGAGATTACTTAGGCTACTCTCTAAAGG
TTAGTCCATGAACCTT
>Olfr1402 SEA/GnJ
CACCAGGGAGGAGGATAGTCAGGCTTTCAGGCTTCAGCCACAGCAATTCCCAACAAT
ACATTTAGGACTGCAATTTACTAACAAATGTCCCAAAAGAGATAGGGAGCTGTTCTC
ATTGCATTTTGGGAAAAGATGATTTCTATGACCTTAATAAATACACAAGAGAGTTAA
TTGTTCTTCATTTCTACAGCTTCTGTTTTGGCTGTTGCCTTTGATTTGTATTTGTGT
GCTAAGGCGTTTCAGACAGCTGCTGAGAGAGAGATTACTTAGGCTACTCTCTAAAGG
TTAGTCCATGAACCTT
>Olfr1402 SPRET/EiJ
CACCAGGGAGGAGGATAGTCAGGCTTTCAGGCTTCAGCCACAGCAATTCCCAACAAT
ACATTTAGGACTGCAATTTACTAACTAATGTCCCAAAAGAGATAGGGAGCTGTTCTC

ATTGCATTTTGGGAAAAGATGATTTCTATGACCTTAATAAATACCCAAGAGAGTTAA
TTGTTCTTCATTTCTACAGCTTCTGTTTTGGCTGTTGCCTTTGATTTGTATTTGTGT
GCTAAGGCATTTCAGACAGCTGCTGAGAGAGAGATTACTTAGGCTACTCTCTAATGG
TTAGTCCATGAACCTT
>Olfr1402 ST/bJ
CACCAGGGAGGAGGATAGTCAGGCTTTCAGGCTTCAGCCACAGCAATTCCCAACAAT
ACATTTAGGACTGCAATTTACTAACAAATGTCCCAAAAGAGATAGGGAGCTGTTCTC
ATTGCATTTTGGGAAAAGATGATTTCTATGACCTTAATAAATACACAAGAGAGTTAA
TTGTTCTTCATTTCTACAGCTTCTGTTTTGGCTGTTGCCTTTGATTTGTATTTGTGT
GCTAAGGCGTTTCAGACAGCTGCTGAGAGAGAGATTACTTAGGCTACTCTCTAAAGG
TTAGTCCATGAACCTT
>Olfr1402 WSB/EiJ
CACCAGGGAGGAGGATAGTCAGGCTTTCAGGCTTCAGCCACAGCAATTCCCAACAAT
ACATTTAGGACTGCAATTTACTAACAAATGTCCCAAAAGAGATAGGGAGCTGTTCTC
ATTGCATTTTGGGAAAAGATGATTTCTATGACCTTAATAAATACACAAGAGAGTTAA
TTGTTCTTCATTTCTACAGCTTCTGTTTTGGCTGTTGCCTTTGATTTGTATTTGTGT
GCTAAGGCGTTTCAGACAGCTGCTGAGAGAGAGATTACTTAGGCTACTCTCTAAAGG
TCAGTCCATGAACCTT
>Olfr1402 ZALENDE/EiJ
CACCAGGGAGGAGGATAGTCAGGCTTTCAGGCTTCAGCCACAGCAATTCCCAACAAT
ACATTTAGGACTGCAATTTACTAACAAATGTCCCAAAAGAGATAGGGAGCTGTTCTC
ATTGCATTTTGGGAAAAGATGATTTCTATGACCTTAATAAATACACAAGAGAGTTAA
TTGTTCTTCATTTCTACAGCTTCTGTTTTGGCTGTTGCCTTTGATTTGTATTTGTGT
GCTAAGGCGTTTCAGACAGCTGCTGAGAGAGAGATTACTTAGGCTACTCTCTAAAGG
TCAGTCCATGAACCTT
